# The *Lancet Global Health* Commission on Global Eye Health: vision beyond 2020

**DOI:** 10.1016/S2214-109X(20)30488-5

**Published:** 2021-02-16

**Authors:** Matthew J Burton, Jacqueline Ramke, Ana Patricia Marques, Rupert R A Bourne, Nathan Congdon, Iain Jones, Brandon A M Ah Tong, Simon Arunga, Damodar Bachani, Covadonga Bascaran, Andrew Bastawrous, Karl Blanchet, Tasanee Braithwaite, John C Buchan, John Cairns, Anasaini Cama, Margarida Chagunda, Chimgee Chuluunkhuu, Andrew Cooper, Jessica Crofts-Lawrence, William H Dean, Alastair K Denniston, Joshua R Ehrlich, Paul M Emerson, Jennifer R Evans, Kevin D Frick, David S Friedman, João M Furtado, Michael M Gichangi, Stephen Gichuhi, Suzanne S Gilbert, Reeta Gurung, Esmael Habtamu, Peter Holland, Jost B Jonas, Pearse A Keane, Lisa Keay, Rohit C Khanna, Peng Tee Khaw, Hannah Kuper, Fatima Kyari, Van C Lansingh, Islay Mactaggart, Milka M Mafwiri, Wanjiku Mathenge, Ian McCormick, Priya Morjaria, Lizette Mowatt, Debbie Muirhead, Gudlavalleti V S Murthy, Nyawira Mwangi, Daksha B Patel, Tunde Peto, Babar M Qureshi, Solange R Salomão, Virginia Sarah, Bernadetha R Shilio, Anthony W Solomon, Bonnielin K Swenor, Hugh R Taylor, Ningli Wang, Aubrey Webson, Sheila K West, Tien Yin Wong, Richard Wormald, Sumrana Yasmin, Mayinuer Yusufu, Juan Carlos Silva, Serge Resnikoff, Thulasiraj Ravilla, Clare E Gilbert, Allen Foster, Hannah B Faal

**Affiliations:** a International Centre for Eye Health, London School of Hygiene & Tropical Medicine, London, UK; b Department of Health Services Research and Policy, London School of Hygiene & Tropical Medicine, London, UK; c International Centre for Evidence in Disability, London School of Hygiene & Tropical Medicine, London, UK; d National Institute for Health Research Biomedical Research Centre for Ophthalmology at Moorfields Eye Hospital NHS Foundation Trust and UCL Institute of Ophthalmology, London, UK; e School of Optometry and Vision Science, University of Auckland, Auckland, New Zealand; f Vision and Eye Research Institute, Anglia Ruskin University, Cambridge, UK; g Department of Ophthalmology, Cambridge University Hospitals, Cambridge, UK; h Centre for Public Health, Queen's University Belfast, Belfast, UK; i Zhongshan Ophthalmic Center, Sun Yat-sen University, Guangzhou, China; j Sightsavers, Haywards Heath, UK; k The Fred Hollows Foundation, Melbourne, Australia; l Department of Ophthalmology, Mbarara University of Science and Technology, Mbarara, Uganda; m John Snow India, New Delhi, India; n Ministry of Health and Family Welfare, New Delhi, India; o Peek Vision, London, UK; p Geneva Centre of Humanitarian Studies, University of Geneva, Geneva, Switzerland; q The Medical Eye Unit, St Thomas' Hospital, London, UK; r Leeds Teaching Hospitals NHS Trust, Leeds, UK; s Pacific Eye Care Society, Suva, Fiji; t Ministry of Health, Maputo, Mozambique; u Orbis International, Ulaanbaatar, Mongolia; v Mongolian Ophthalmology Society, Ulaanbaatar, Mongolia; w Vision Catalyst Fund, London, UK; x International Agency for the Prevention of Blindness, London, UK; y Division of Ophthalmology, University of Cape Town, Cape Town, South Africa; z Ophthalmology Department, University Hospital Birmingham NHS Foundation Trust, Queen Elizabeth Hospital Birmingham, Birmingham, UK; aa Health Data Research UK, London, UK; ab Department of Ophthalmology and Visual Sciences, University of Michigan, Ann Arbor, MI, USA; ac Institute for Healthcare Policy and Innovation, University of Michigan, Ann Arbor, MI, USA; ad International Trachoma Initiative and Rollins School of Public Health, Emory University, Atlanta, GA, USA; ae Carey Business School, Johns Hopkins University, Baltimore, MD, USA; af Dana Center for Preventive Ophthalmology, Wilmer Eye Institute, Johns Hopkins University, Baltimore, MD, USA; ag Department of Epidemiology, Johns Hopkins Bloomberg School of Public Health, Johns Hopkins University, Baltimore, MD, USA; ah Massachusetts Eye and Ear, Harvard Ophthalmology, Harvard Medical School, Boston, MA, USA; ai Ribeirão Preto Medical School, University of São Paulo, São Paulo, Brazil; aj Ophthalmic Services Unit, Ministry of Health, Nairobi, Kenya; ak Department of Ophthalmology, University of Nairobi, Nairobi, Kenya; al Research and Strategy, Seva Foundation, Berkeley, CA, USA; am Tilganga Institute of Ophthalmology, Kathmandu, Nepal; an Eyu-Ethiopia Eye Health Research, Training, and Service Centre, Bahirdar, Ethiopia; ao Institute of Clinical and Scientific Ophthalmology and Acupuncture Jonas and Panda, Heidelberg, Germany; ap Department of Ophthalmology, Medical Faculty Mannheim, Heidelberg University, Mannheim, Germany; aq Institute of Molecular and Clinical Ophthalmology Basel, Basel, Switzerland; ar School of Optometry and Vision Science, University of New South Wales, Sydney, Australia; as George Institute for Global Health, University of New South Wales, Sydney, Australia; at Gullapalli Pratibha Rao International Centre for Advancement of Rural Eye Care, LV Prasad Eye Institute, Hyderabad, India; au Brien Holden Eye Research Centre, LV Prasad Eye Institute, Hyderabad, India; av College of Health Sciences, University of Abuja, Abuja, Nigeria; aw Instituto Mexicano de Oftalmologia, Queretaro, Mexico; ax Centro Mexicano de Salud Visual Preventiva, Mexico City, Mexico; ay Help Me See, New York, NY, USA; az Department of Ophthalmology, Muhimbili University of Health and Allied Sciences, Dar es Salaam, Tanzania; ba Rwanda International Institute of Ophthalmology, Kigali, Rwanda; bb University Hospital of the West Indies, Kingston, Jamaica; bc Nossal Institute for Global Health, University of Melbourne, Melbourne, VIC, Australia; bd Melbourne School of Population and Global Health, University of Melbourne, Melbourne, VIC, Australia; be Indian Institute of Public Health, Hyderabad, India; bf Kenya Medical Training College, Nairobi, Kenya; bg Christian Blind Mission, Cambridge, UK; bh Departamento de Oftalmologia e Ciências Visuais, Escola Paulista de Medicina, Universidade Federal de São Paulo, São Paulo, Brazil; bi The Fred Hollows Foundation, London, UK; bj Department of Curative Services, Ministry of Health Community Development, Gender, Elderly, and Children, Dodoma, Tanzania; bk Department of Control of Neglected Tropical Diseases, WHO, Geneva, Switzerland; bl Beijing Institute of Ophthalmology, Beijing Tongren Eye Center, Beijing Tongren Hospital, Capital Medical University, Beijing, China; bm Beijing Ophthalmology and Visual Sciences Key Laboratory, Beijing, China; bn Permanent Mission of Antigua and Barbuda to the United Nation, New York, NY, USA; bo Singapore Eye Research Institute, Singapore National Eye Center, Singapore; bp Duke-NUS Medical School, Singapore; bq Sightsavers, Islamabad, Pakistan; br Pan American Health Organization, Bogotá, Colombia; bs Brien Holden Vision Institute, University of New South of Wales, Sydney, Australia; bt LAICO-Aravind Eye Care System, Madurai, India; bu Department of Ophthalmology, University of Calabar, Calabar, Nigeria; bv Africa Vision Research Institute, Durban, South Africa

## Executive Summary

Eye health and vision have widespread and profound implications for many aspects of life, health, sustainable development, and the economy. Yet nowadays, many people, families, and populations continue to suffer the consequences of poor access to high-quality, affordable eye care, leading to vision impairment and blindness.

In 2020, an estimated 596 million people had distance vision impairment worldwide, of whom 43 million were blind. Another 510 million people had uncorrected near vision impairment, simply because of not having reading spectacles. A large proportion of those affected (90%), live in low-income and middle-income countries (LMICs). However, encouragingly, more than 90% of people with vision impairment have a preventable or treatable cause with existing highly cost-effective interventions. Eye conditions affect all stages of life, with young children and older people being particularly affected. Crucially, women, rural populations, and ethnic minority groups are more likely to have vision impairment, and this pervasive inequality needs to be addressed. By 2050, population ageing, growth, and urbanisation might lead to an estimated 895 million people with distance vision impairment, of whom 61 million will be blind. Action to prioritise eye health is needed now.

This Commission defines eye health as maximised vision, ocular health, and functional ability, thereby contributing to overall health and wellbeing, social inclusion, and quality of life. Eye health is essential to achieve many of the Sustainable Development Goals (SDGs). Poor eye health and impaired vision have a negative effect on quality of life and restrict equitable access to and achievement in education and the workplace. Vision loss has substantial financial implications for affected individuals, families, and communities. Although high-quality data for global economic estimates are scarce, particularly for LMICs, conservative assessments based on the latest prevalence figures for 2020 suggest that annual global productivity loss from vision impairment is approximately US$410·7 billion purchasing power parity. Vision impairment reduces mobility, affects mental wellbeing, exacerbates risk of dementia, increases likelihood of falls and road traffic crashes, increases the need for social care, and ultimately leads to higher mortality rates.

By contrast, vision facilitates many daily life activities, enables better educational outcomes, and increases work productivity, reducing inequality. An increasing amount of evidence shows the potential for vision to advance the SDGs, by contributing towards poverty reduction, zero hunger, good health and wellbeing, quality education, gender equality, and decent work. Eye health is a global public priority, transforming lives in both poor and wealthy communities. Therefore, eye health needs to be reframed as a development as well as a health issue and given greater prominence within the global development and health agendas.

Vision loss has many causes that require promotional, preventive, treatment, and rehabilitative interventions. Cataract, uncorrected refractive error, glaucoma, age-related macular degeneration, and diabetic retinopathy are responsible for most global vision impairment. Research has identified treatments to reduce or eliminate blindness from all these conditions; the priority is to deliver treatments where they are most needed. Proven eye care interventions, such as cataract surgery and spectacle provision, are among the most cost-effective in all of health care. Greater financial investment is needed so that millions of people living with unnecessary vision impairment and blindness can benefit from these interventions.

Lessons from the past three decades give hope that this challenge can be met. Between 1990 and 2020, the age-standardised global prevalence of blindness fell by 28·5%. Since the 1990s, prevalence of major infectious causes of blindness—onchocerciasis and trachoma—have declined substantially. Hope remains that by 2030, the transmission of onchocerciasis will be interrupted, and trachoma will be eliminated as a public health problem in every country worldwide. However, the ageing population has led to a higher crude prevalence of age-related causes of blindness, and thus an increased total number of people with blindness in some regions.

**Key messages**

**Eye health is essential to achieve the Sustainable Development Goals; vision needs to be reframed as a development issue**

There is extensive evidence showing that improving eye health contributes directly and indirectly to achieving many Sustainable Development Goals, including reducing poverty and improving work productivity, general and mental health, and education and equity. Improving eye health is a practical and cost-effective way of unlocking human potential. Eye health needs to be reframed as an enabling, cross-cutting issue within the sustainable development framework.

**Almost everyone will experience impaired vision or an eye condition during their lifetime and require eye care services; urgent action is necessary to meet the rapidly growing eye health need**

In 2020, 1·1 billion people had distance vision impairment or uncorrected presbyopia. By 2050, this figure is expected to rise to 1·8 billion. Most affected people live in low-income and middle-income countries (LMICs) with avoidable causes of vision impairment. During the life course, most people will experience vision impairment, even if just the need for reading glasses. Because of unmet needs and an ageing global population, eye health is a major public health and sustainable development concern which warrants urgent political action.

**Eye health is an essential component of universal health coverage; it must be included in planning, resourcing, and delivery of health care**

Universal health coverage is not universal without affordable, high quality, equitable eye care. In line with the WHO *World report on vision*, we urge countries to consider eye care as an essential service within universal health coverage. To deliver comprehensive services including promotion, prevention, treatment, and rehabilitation, eye care needs to be included in national strategic health plans and development policies, health financing structures, and health workforce planning. Coordinated intersectoral action is needed to systematically improve population eye health, also within healthy ageing initiatives, schools, and the workplace. Integration of eye health services with multiple relevant components of health service delivery and at all levels of the health system is of central importance.

**High quality eye health services are not universally delivered; concerted action is needed to improve quality and outcomes, providing effective, efficient, safe, timely, equitable, and people-centred care**

Use of effective service coverage indicators for cataract and refractive error highlight the delivery gap between population eye health needs and the delivery of good outcomes. We urge eye health providers to take a holistic view to emphasise quality and design service delivery based on individual and population needs: a people-centred approach. Services need to be characterised by inclusiveness and equity in design and delivery, proactively addressing the needs of marginalised and vulnerable groups through targeted interventions. To encourage improved quality in cataract surgery, we support redefining a good vision outcome threshold as 6/12 or better.

**Highly cost-effective vision-restoring interventions offer enormous potential to improve the economic outlook of individuals and nations; a major scale up of financial investment in eye health is required**

For 2020, we estimate that vision impairment resulted in $410·7 billion lost economic productivity; the full cost is most likely higher. Treatments for cataract and refractive error would meet more than 90% of unmet needs and are highly cost-effective. The case for countries to invest in improving population eye health is compelling and more financial resources are urgently required.

**Financial barriers to accessing eye care leave many people behind; eye health needs to be included in national health financing to pool the risk**

Health-care costs prevent many people from accessing essential eye health services. Eye care needs to be integrated into general health system financing to remove cost barriers. To improve access for the whole population and mitigate eye care expenditure, mechanisms that pool risk are highly desirable.

**Technology and treatment developments offer new tools to improve eye health; thoughtful application is needed to maximise the potential to improve coverage, accessibility, quality, efficiency, and affordability**

Technological developments such as telemedicine, mHealth, and artificial intelligence offer the potential to revolutionise eye health care in the next decade by delivering affordable, high-quality services to remote areas. However, caution is needed to ensure all populations benefit from these developments.

**The eye health workforce is unable to meet population needs in many countries; major expansion in service capacity is required through increased numbers, sharing tasks, strengthened training, enabling work environments, and effective leadership**

Many areas have major shortages of personnel working in eye health. The available workforce needs to be distributed according to population need. Quality of training for the workforce needs to be updated, with renewed emphasis on competency. Enabling working environments need to be created, including appropriate support, supervision, and equipment. Long-standing issues of low productivity need to be systematically resolved. Mentoring and other programmes to cultivate an emerging generation of eye health leaders are needed.

**Reliable survey and service data are key to progress in eye health; robust indicator data are needed to shape change and drive action**

To monitor progress in delivering improved eye health within universal health coverage, a balanced set of robust indicators are needed, which we outlined in this Commission. Service data should be available and used by implementers and policy makers to drive change. We highlight the scarcity of epidemiological data in several regions, which should be addressed as a priority.

**Research has been crucial to advances in understanding and treating eye disease; solution-focused, contextually relevant research is urgently needed to deliver innovative prevention and treatment strategies and inform implementation of eye health within universal health coverage**

Implementation research is needed, particularly in LMICs, to guide effective delivery of services within universal health coverage. Discovery research is needed for specific areas that remain without efficacious interventions. The economic impact of vision impairment, and the costs and benefits of interventions are only partly understood; a coordinated global effort to systematically collect data is needed. A step-change in the capacity of LMICs to do contextually relevant eye health research and a greater commitment are needed to improve diversity and inclusion in the research community.

Despite this progress, business as usual will not keep pace with the demographic trends of an ageing global population or address the inequities that persist in each country. New threats to eye health are emerging, including the worldwide increase in diabetic retinopathy, high myopia, retinopathy of prematurity, and chronic eye diseases of ageing such as glaucoma and age-related macular degeneration. With the projected increase in such conditions and their associated vision loss over the coming decades, urgent action is needed to develop innovative treatments and deliver services at a greater scale than previously achieved.

Good eye health at the community and national level has been marginalised as a luxury available to only wealthy or urban areas. Eye health needs to be urgently brought into the mainstream of national health and development policy, planning, financing, and action.

The challenge is to develop and deliver comprehensive eye health services (promotion, prevention, treatment, rehabilitation) that address the full range of eye conditions within the context of universal health coverage. Accessing services should not bring the risk of falling into poverty and services should be of high quality, as envisaged by the WHO framework for health-care quality: effective, safe, people-centred, timely, equitable, integrated, and efficient. To this framework we add the need for services to be environmentally sustainable. Universal health coverage is not universal without eye care.

Multiple obstacles need to be overcome to achieve universal coverage for eye health. Important issues include complex barriers to availability and access to quality services, cost, major shortages and maldistribution of well-trained personnel, and lack of suitable, well maintained equipment and consumables. These issues are particularly widespread in LMICs, but also occur in underserved communities in high-income countries. Strong partnerships need to be formed with natural allies working in areas affected by eye health, such as non-communicable diseases, neglected tropical diseases, healthy ageing, children's services, education, disability, and rehabilitation. The eye health sector has traditionally focused on treatment and rehabilitation, and underused health promotion and prevention strategies to lessen the impact of eye disease and reduce inequality.

Solving these problems will depend on solutions established from high quality evidence that can guide more effective implementation at scale. Evidence-based approaches will need to address existing deficiencies in the supply and demand. Strategic investments in discovery research, harnessing new findings from diverse fields, and implementation research to guide effective scale up are needed globally. Encouragingly, developments in telemedicine, mobile health, artificial intelligence, and distance learning could potentially enable eye care professionals to deliver higher quality care that is more plentiful, equitable, and cost-effective.

This Commission did a Grand Challenges in Global Eye Health prioritisation exercise to highlight key areas for concerted research and action. This exercise has identified a broad set of challenges spanning the fields of epidemiology, health systems, diagnostics, therapeutics, and implementation. The most compelling of these issues, picked from among 3400 suggestions proposed by 336 people from 118 countries, can help to frame the future research agenda for global eye health.

In this Commission, we harness lessons learned from over two decades, present the growing evidence for the life-transforming impact of eye care, and provide a thorough understanding of rapid developments in the field. This report was created through a broad consultation involving experts within and outside the eye care sector to help inform governments and other stakeholders about the path forward for eye health beyond 2020, to further the SDGs (including universal health coverage), and work towards a world without avoidable vision loss.

The next few years are a crucial time for the global eye health community and its partners in health care, government, and other sectors to consider the successes and challenges encountered in the past two decades, and at the same time to chart a way forward for the upcoming decades. Moving forward requires building on the strong foundation laid by WHO and partners in VISION 2020 with renewed impetus to ultimately deliver high quality universal eye health care for all.

## Introduction

In 2020, an estimated 596 million people worldwide had distance vision impairment and a further 510 million had uncorrected near vision impairment.^1^ Most of these people live in low-income and middle-income countries (LMIC). Eye health is also affected by conditions that do not, at least initially, impair vision. Although these conditions are not currently included in global prevalence estimates, they contribute substantially to the unmet need for eye health services. Vision is important for many aspects of life, and vision impairment can profoundly affect individuals, families, and society. Eye health touches all lives, either directly or indirectly, through its impact on those close to us.

The year 2020 marks the culmination of the global initiative to eliminate avoidable blindness, VISION 2020: The Right to Sight (appendix 1 p 7). This initiative provided the framework for national programmes to address eye health over the past 20 years. In 2019, WHO published the *World report on vision*,^2^ which was endorsed by the 73rd World Health Assembly in 2020. The report and resolution call for the advancing of eye health as an integral part of universal health coverage, by implementation of integrated people-centred eye care, following the approach outlined in a broader health services framework.^3^

*The Lancet Global Health* Commission on Global Eye Health contends that eye health should be part of the mainstream agenda to achieve universal health coverage and sustainable development. We define eye health as the state in which vision, ocular health, and functional ability are maximised, thereby contributing to overall health and wellbeing, social inclusion, and quality of life. Eye health can be considered both a process and an outcome. We define eye care services as those that contribute to any of vision, ocular health, or functional ability being maximised.

This report broadly divides into two halves. First, we present evidence for the importance of eye health, supporting the case for urgent action. Second, looking beyond 2020, we examine approaches to enable delivery of eye health services within universal health coverage. In section 1 we summarise the visual system, vision impairment, and common conditions. In section 2, we synthesise several reviews done by the Commission on the relevance of eye health to the Sustainable Development Goals (SDGs), as well as its impact on quality of life, general health, and mortality. In section 3, we describe the magnitude and causes of vision impairment in 2020 and projected global and regional trends. We explore service needs of people with non-vision impairing eye conditions. We propose a more standardised approach to reporting population-based eye health surveys and examine the disability weights applied to vision impairment. In section 4, we summarise findings from a systematic review of eye health economics, identifying important areas for future work. We present a new estimate of global lost productivity associated with vision impairment for 2020, and an analysis of the cost-effectiveness ratios for cataract surgery and refractive error services. In section 5, we outline a bibliometric analysis of eye health research since 2000, and report a global Grand Challenges project, highlighting crucial issues for concerted research and action. Lastly, we address the question of how health systems can practically advance towards delivering high quality integrated people-centred eye care within universal health coverage.^2^ We argue that business as usual will be insufficient, as evidenced by new analysis of effective cataract surgical coverage data. We examine service delivery components: primary eye care and integration with general health services, workforce strengthening, financing, health information systems, indicators, advocacy, and approaches to increase quality and equity.

### The development of global eye health

**Figure 1:**
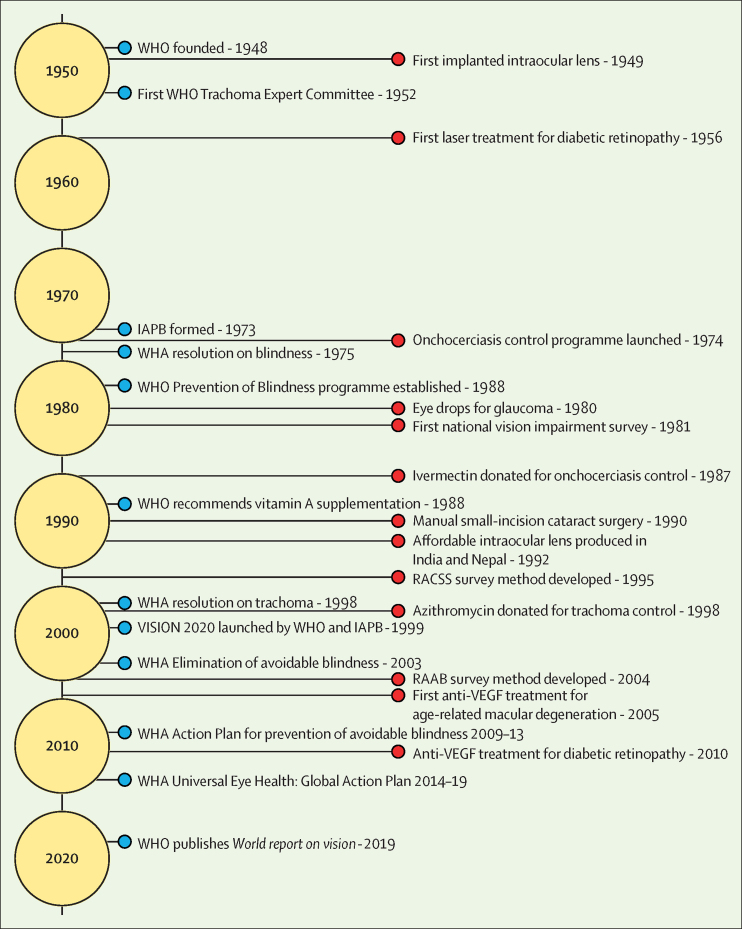
The development of global eye health Blue circles indicate major global developments. Red circles indicate major treatments and programmatic developments. WHA=World Health Assembly. IAPB=International Agency for the Prevention of Blindness. RAAB=Rapid Assessment of Avoidable Blindness. RACSS=Rapid Assessment of Cataract Surgical Services.

This Commission views global eye health through the global health framework articulated by Koplan and colleagues.^4^ Eye health started with an understanding of the anatomy, physiology, diseases of the eye, and the development of clinical ophthalmology, the medical and surgical discipline for diagnosis and treatment of eye diseases. From the mid-20th century onwards (figure 1), there have been major technological advances in microsurgical techniques for cataract and other conditions, and equipment for diagnosis and treatment of major non-communicable eye diseases, resulting in more effective interventions. There has been an enormous demographic transition, with ageing populations and epidemiological changes from infectious diseases and towards non-communicable diseases, requiring accessible and affordable eye services with long-term follow-up. The increase in demand, emphasis on better quality, and higher cost of more sophisticated diagnostic and treatment services is requiring an increase in resources, which presents enormous public health challenges.

There are many lessons from the past 70 years that are instructive for the future of global eye health (appendix 1 p 8). First, the importance of advocacy in creating global platforms to address a public health issue. Second, the value of common definitions, high quality data and research to develop global, regional, and national health programmes. Third, the importance of identifying and addressing specific eye diseases of public health importance that can be eliminated through public–private partnerships. Fourth, that the VISION 2020 initiative created an easily understood message for advocacy and planning services, and a global partnership involving different stakeholders in public health, including the private sector and non-governmental organisations, which resulted in extra resources and a common goal and focus. Fifth, that in promoting a global programme, inadequate attention was given to the engagement and partnership with ministries of health to ensure national ownership. There remains a need to integrate eye care planning and resource allocation into national health systems and share the achievements and successes. Sixth, that the transition from elimination of focal eye diseases with regional programmes and international funding to the development of comprehensive services to achieve universal eye health requires engagement, commitment, and leadership by the ministries of health and the willingness of all stakeholders (including the private sector) to support eye care services integrated within national health-care plans.

## Section 1: The eyes, vision impairment, and eye conditions

### The visual system

**Figure 2:**
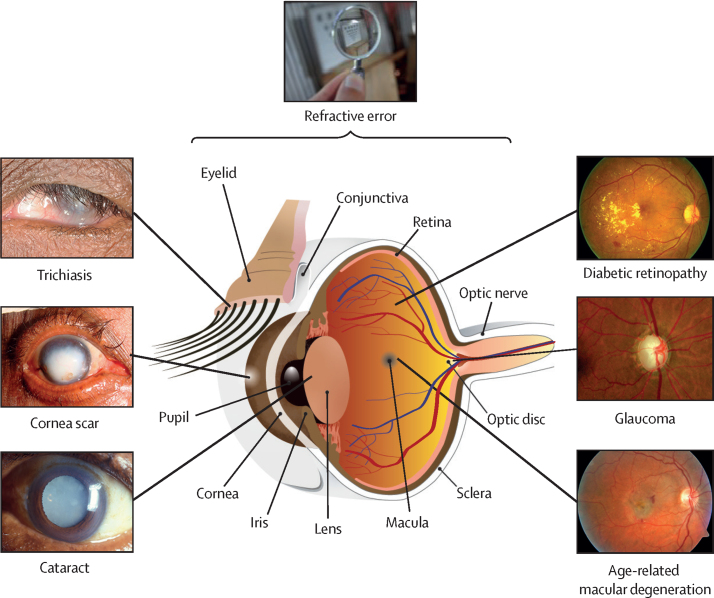
The human eye and common conditions The location and clinical appearance of common causes of vision impairment.

Vision is the most dominant of human senses. The eye, its associated adnexal tissues, and visual pathways within the brain are very intricate (figure 2). All these elements need to function well together to achieve clear vision. The transparent optical elements at the front of the eye (cornea and lens) focus light onto retinal photoreceptors. These transduce light stimuli into neuronal impulses with which the brain creates a three-dimensional image. Vision requires structural and physiological integrity of the eyes, brain, and their connections. Disruption of any part of this pathway causes vision impairment.

### Measuring visual function

Our eyesight has several distinct components that require specific types of testing, including visual acuity (distance and near), contrast sensitivity, colour vision, and visual fields (appendix 1 p 9). Tests of cerebral visual function include interpreting the meaning of a picture or recognising faces. Vision-driven activities of daily living can be captured using quality of life tools and vision function-related tasks. The most common measure of visual function is distance visual acuity, which tests the ability to discern letters or characters of high contrast at decreasing size using the central retina.

### Defining vision impairment

There have been progressive developments in WHO recommendations on how vision impairment is measured, defined, and categorised, particularly in population-based epidemiological surveys (appendix 1 p 10). *International Classification of Diseases 11th Revision* (ICD-11) definitions of distance vision impairment were updated in 2019 with additional subdivisions (table 1).^5^ In the *World report on vision*,^2^ WHO has signalled an intention to change the way vision impairment is reported, moving away from only presenting visual acuity (with spectacles or contact lenses if available), to also reporting uncorrected visual acuity (without spectacles or contact lenses if worn). Including the measurement of uncorrected acuity allows for better estimation of the ongoing service need and effective coverage of refractive error correction. In prevalence surveys, vision impairment is generally reported as visual acuity in the better seeing eye. This Commission uses visual acuity categories defined on Snellen charts in metres when presenting data. Moderate and severe vision impairment (MSVI) is defined as visual acuity worse than 6/18, but equal to or better than 3/60, and blindness is defined as worse than 3/60 (table 1).

**Table 1:** WHO definitions for vision impairment

	**Distance visual acuity worse than**	**Distance visual acuity equal to or better than**
**0 - no vision impairment**		
Snellen, metres	NA	6/12
Snellen, feet	NA	20/40
LogMAR	NA	0·30
Decimal	NA	0·5
**1 - mild vision impairment**		
Snellen, metres	6/12	6/18
Snellen, feet	20/40	20/60
LogMAR	0·30	0·50
Decimal	5/10 (0·5)	0·3
**2 - moderate vision impairment**		
Snellen, metres	6/18	6/60
Snellen, feet	20/60	20/200
LogMAR	0·50	1·00
Decimal	3/10 (0·3)	0·1
**3 - severe vision impairment**		
Snellen, metres	6/60	3/60
Snellen, feet	20/200	20/400
LogMAR	1·00	1·30
Decimal	1/10 (0·1)	0·05
**4 - blindness**		
Snellen, metres	3/60	1/60*
Snellen, feet	20/400	5/300
LogMAR	1·30	1·80
Decimal	1/20 (0·05)	0·02
**5 - blindness**		
Snellen, metres	1/60*	Light perception
Snellen, feet	5/300	Light perception
LogMAR	1·80	Light perception
Decimal	1/50 (0·02)	Light perception
**6 - blindness**		
Test for light perception	Light perception	No light perception

Sourced from WHO, 2019.^5^ There are several visual acuity chart types, which differ in the number and type of characters (optotypes), spacing, and reporting formats. The participant is asked to read down the chart, which has multiple rows with progressively smaller characters, at a specific distance. The visual acuity is the line with the smallest characters correctly identified. Snellen is expressed as a fraction: the numerator is the test distance and the denominator is the smallest line size correctly read. For each vision impairment category, the equivalent visual acuity thresholds are presented. NA=not applicable. LogMAR=Logarithm of the Minimum Angle of Resolution.

* Or counting fingers at 1 metre.

### Common eye conditions

Many conditions can affect eye health, and even those that do not cause vision impairment can produce pronounced morbidity. Common eye conditions covering key clinical features, epidemiology, and management are summarised in appendix 1 (pp 11–14). The most common causes of vision impairment in adults are uncorrected refractive error, cataract, glaucoma, age-related macular degeneration, diabetic retinopathy, corneal scarring, and trachoma (figure 2). Among children the leading causes of blindness and MSVI include uncorrected refractive error, cataract, retinopathy of prematurity, congenital ocular anomalies, corneal scarring, and cerebral visual impairment. There are also many conditions causing pronounced symptoms (eg, pain, itching, discharge) and affecting a large number of people without vision impairment. These include infectious and allergic conjunctivitis, blepharitis, and dry eyes. The resulting morbidity needs to be addressed, forming a substantial proportion of ophthalmic service work.^6^

### Social determinants of eye health

Public health approaches can prevent or treat most common eye diseases. Vision loss and access to eye care is greatly affected by social determinants.^7^, ^8^ These encompass many issues: social exclusion, gender inequity, racism, early childhood development, educational opportunities, employment conditions, design and implementation of health systems and public health programmes, urbanisation, globalisation, and commercial determinants.^7^, ^9^ Inequity in health, between and within countries, is mostly attributable to social determinants of health.^9^ We will explore questions on access, equity, and health financing in the wider context of universal health coverage in section 6.^10^

### Eye health during the life course

**Figure 3:**
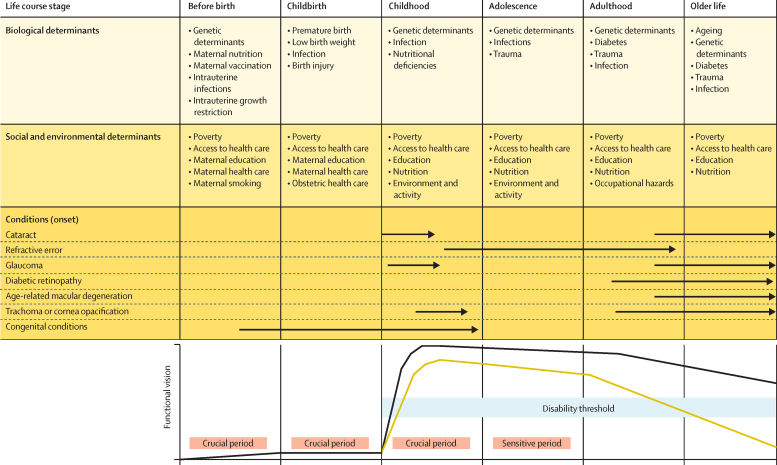
Life course perspective on eye health Arrows indicate the period in the life course in which different conditions typically present. The yellow line indicates a hypothetical functional vision trajectory of someone with a condition leading to increased vision impairment. The black line represents the functional vision trajectory of someone who does not have a condition leading to vision impairment. This figure is partly based on the concept of functional trajectories illustrated in WHO, 2001.^12^ The disability threshold represents the level of functional vision below which there is functional vision impairment.

Life course perspectives focus on health trajectories during key developmental periods and across the whole lifespan.^11^ At each stage of life, multiple biological, socioeconomic, and environmental factors interact to determine the development and course of eye health (figure 3).^13^ Lifelong accumulation of risk factors, particularly during crucial periods of visual development, influence visual function trajectories and underlie marked regional differences in vision impairment.^12^

The socioeconomic environment into which a child is born has profound effects on eye health over the individual's life course. Maternal nutritional and vaccination status, and development of intrauterine infections (rubella, toxoplasmosis, syphilis, Zika) are important determinants.^14^, ^15^ Preterm birth can lead to retinopathy of prematurity and cerebral visual impairment. Low birthweight, fetal growth restriction, antenatal maternal smoking and alcohol misuse, and social deprivation in childhood can also cause vision impairment.^16^, ^17^ Congenital eye conditions frequently have strong genetic components. Over the life course, biological and social determinants interact to determine visual function. For example, retinoblastoma, a mostly genetically determined childhood eye cancer, is not expected to vary by socioeconomic status. However, socioeconomic and cultural factors that influence timely access and adherence to treatment are responsible for important differences in vision and survival outcomes.^18^

Visual acuity develops rapidly after birth and reaches full development at around age 8 years. Early childhood is a crucial period because visual cortex plasticity progressively diminishes after age 2 years. Since vision is important for early child development, early onset vision impairment can lead to psychomotor and cognitive developmental delay.^19^ Visual stimulus deprivation between birth and age 8 years can lead to permanent vision impairment (amblyopia) if not managed in a timely manner.^11^ As the eyes grow and change shape there is a further sensitive period from childhood to adolescence when a combination of genetic and environmental factors, such as light exposure and time spent outdoors, can lead to myopia.^20^ Several infectious diseases (trachoma, toxoplasmosis, onchocerciasis) begin in childhood and lead to vision impairment later in life from accumulated pathology.^21^, ^22^

Many conditions are age-related. Presbyopia starts developing from age 35 years, as the lens ages. Some conditions (eg, glaucoma and age-related macular degeneration) have a complex polygenetic background, which can interact with nutrition and other biological factors.^23^, ^24^ Diabetes and diabetic retinopathy are influenced by multiple social and environmental determinants (diet, activity, obesity).^25^ Cataract arises from multiple factors across the life course that promote lens ageing: ultraviolet light exposure, smoking, poor nutrition, diabetes, and severe dehydration.^26^

The life course trajectory of visual function is not fixed. Many conditions and risk factors are amenable to interventions, including social determinants, along the spectrum of promotion, prevention, treatment, and rehabilitation. These are complex issues, requiring multisectoral approaches (nutrition, housing, social security, education), long-term policies, and health system investment for greater health equity.^13^, ^27^

## Section 2: The importance of eye health

### Eye health and the SDGs

**Figure 4:**
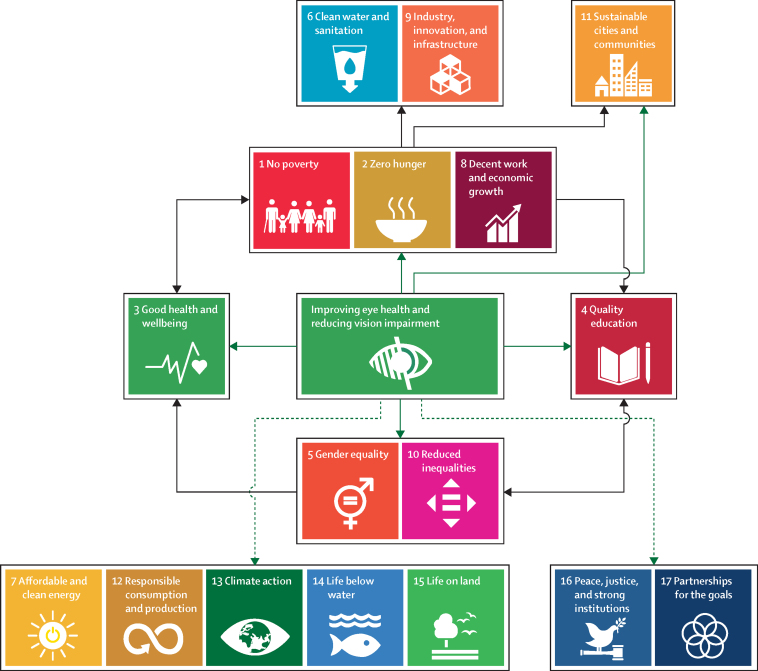
Improving eye health and Sustainable Development Goals Green arrows indicate relationships with direct evidence of a beneficial effect from improving eye health on Sustainable Development Goals. Dashed green arrows represent hypothesised direct beneficial effects. Black arrows represent possible indirect beneficial effects.

The UN SDGs are a group of broad target-driven goals for 2030, designed as a “blueprint to achieve a better and more sustainable future for all”.^28^ We did a series of systematic and scoping reviews to examine the relationship between eye health and the SDGs. Together, these reviews provide compelling evidence that improving access to eye health services will contribute to achieving many SDGs, including the goals to reduce poverty and increased work productivity, health, education, and equity (figure 4). Furthermore, progress towards many SDGs will benefit vision and eye health. Therefore, we believe improving eye health should be viewed principally as a human development issue.

This Commission explored the relationship between eye health and general health and wellbeing (SDG3) in six separate reviews, as well as doing a further review on the relation between eye health and the 16 other SDGs (appendix 1 p 15; table 2).^57^ In terms of these 16 SDGs, we showed that the provision of eye care services is associated with improvements in workplace productivity,^29^ household consumption,^30^, ^31^ household income,^32^, ^33^, ^34^, ^35^ employment prospects,^36^ and economic productivity.^37^, ^38^, ^39^, ^40^ Economic benefits, particularly in resource-limited communities, contribute to achieving SDGs such as poverty reduction (SDG1), food security (SDG2), and decent work (SDG8). An example of benefits resulting from provision of eye care services is shown by the Cataract Impact Study^30^, ^31^ done in Kenya, Bangladesh, and the Philippines (appendix 2 p 3).

**Table 2:** Eye health and Sustainable Development Goals

	**Number and type of study**	**Summary of study findings**
**Poverty-related (SDGs 1, 2, 8)**		
Relative productivity in the workplace	One (randomised controlled trial)	Provision of free spectacles to tea workers with presbyopia in India improved workplace relative productivity by 22% (p<0·0001)^29^
Household per-capita expenditure	Two (prospective cohort studies)	Increase in household per-capita expenditure in people with vision impairment who underwent cataract surgery—eg, in the Philippines, increase by 88% over 1 year (p<0·001)^30^, ^31^
Household income	Four (prospective cohort studies and one retrospective cohort study)	Household income increased after cataract surgery—eg, 1 year after provision of surgery for marginalised communities in rural India,^32^, ^33^, ^34^ the proportion of households with a monthly income (<1000 Rupees) decreased from 51% to 21% (p=0·05); in the USA,^35^ children who became blind by age of 6 years and attended vision impairment schools had a lower salary than those who attended public schools (possibly confounded by other determinants)
Employment rates	One (retrospective cohort study)	Vocational rehabilitation services for vision impairment in the USA were positively associated with paid employment—eg, training and support services increased odds of obtaining paid employment (odds ratio 1·10, p=0·001)^36^
Economic productivity	Four (cost-effectiveness and evaluation studies)	Benefits to economic productivity from cataract surgery^37^, ^38^ and trichiasis surgery^39^, ^40^—eg, there was a net 13-year US$123·4-billion return on investment from a 1-year cohort after cataract surgery, including an increase in US national productivity of US$25·4 billion^37^
**Quality education (SDG 4)**		
Academic test scores	Seven (randomised controlled trials and prospective cohort studies)	Providing children with spectacles improved academic test scores^41^, ^42^, ^43^, ^44^, ^45^, ^46^, ^47^—eg, in China, vision correction reduced the odds of failing a class by 44% (p<0·01)^43^
Reading or word identification	Two (cohort studies)	Improved reading and word identification with spectacle wear and attendance at specialised schools^35^, ^48^
**Inequalities (SDGs 5, 10)**		
Gender inequality	Three (systematic review with meta-analysis, and pair of cross-sectional surveys)	Reduced gender inequality in all-cause blindness, clinic attendance, cataract surgery coverage, and trachoma treatment coverage following interventions to promote eye services by trained rural community volunteers in low-income and middle-income countries;^49^ free cataract screening and low-cost quality cataract surgery in China reduced gender disparity in willingness to pay at 5-year follow-up (88% men, 91% women) compared with baseline (67% men, 50% women)^50^
Equity (per-capita expenditure)	One (cohort study)	People who had cataract surgery in Kenya, the Philippines, and Bangladesh were poorer than non-visually impaired people before surgery (p≤0·02), but after surgery, there was no difference in household per-capita expenditure between the groups (p≥0·2), showing equity improvement^33^
Inequalities in use of eye care services	One (series of repeat cross-sectional studies)	Free eye examinations in Scotland increased use of eye care services at the aggregate level but widened inequalities by income (p<0·001) and education (p<0·001)
**Sustainable cities and communities (SDG 11)**		
Driving-related difficulties	One (meta-analysis)	Reduced driving-related difficulties after cataract surgery (pooled odds ratio 0·12, 95% CI 0·10–0·16)^51^
Motor vehicle crashes	Five (observational studies)	Cataract surgery reduced motor vehicle crashes (all studies statistically significant)^52^, ^53^, ^54^, ^55^, ^56^

Changes in eye health following an intervention are directly linked to one or more Sustainable Development Goals.

Educational performance is linked to vision. Children with vision impairment have poorer educational outcomes and are more likely to be excluded from schools (less likely to attend). We found evidence that providing spectacles to children improves educational performance, supporting quality education (SDG4), with effect sizes at least as large as other health interventions.^41^, ^42^, ^43^, ^44^, ^45^ Improved education is crucial to development, reducing poverty and hunger, and enabling work (SDGs 1, 2, and 8).^58^

Improving eye health contributes to increased gender equity (SDG5) and reduced inequalities (SDG10).^49^ Cataract surgery can improve equity, measured by expenditure.^31^ Women have poorer access to eye health services and more vision impairment; addressing gender inequality will help advance eye health.^59^ Specific interventions such as community outreach vision screening services increase equity for conditions such as cataract and glaucoma among women, marginalised communities, and older people.^51^, ^60^

Improved eye care can contribute to improving the sustainability of cities and communities (SDG11). Studies^52^, ^53^, ^54^, ^55^, ^56^ show that improved vision enhances road safety, thereby contributing to safer cities. Although no studies met our inclusion criteria for the remaining SDGs, we contend that environmentally responsible eye health services would reduce carbon emissions and plastic waste, thereby contributing to progress towards the SDGs for affordable clean energy (SDG7), responsible consumption (SDG12), climate action (SDG13), life below water (SDG14), and life on land (SDG15).^61^, ^62^ Unaddressed eye care needs in displaced populations underscore the potential for such interventions to contribute to the resilience of these disadvantaged communities (SDG16).^63^

Underlying all SDGs is SDG17, strengthening partnerships to achieve the goals. The global eye health community provides examples of effective health-care partnerships, including the GET2020 Alliance^64^ and the African Programme for Onchocerciasis Control,^65^ which have yielded major reductions in the disease burden.

### Vision impairment and health and wellbeing

Vision impairment affects multiple functional domains (physical, cognitive, psychological, social), and overall quality of life and wellbeing.^66^, ^67^ Here, we reflect on vision impairment and inclusive development before summarising each review done by this Commission.

#### Vision impairment and inclusive development

The UN places great emphasis on “realization of the SDGs by, for and with persons with disabilities”, including people with vision loss.^68^ This emphasis recognises that people with vision-related disability play an important part in achieving the SDGs, and their exclusion from schooling and employment is a violation of their rights, as set out in the UN Convention on the Rights of Persons with Disabilities. For example, children with vision impairment in multiple LMICs are up to five times less likely to be in formal education than children without disabilities.^69^ In high-income countries, where school attendance is usually mandatory, children with vision impairment often achieve poorer outcomes (appendix 1 p 16)^70^ and might face social exclusion and violence in schools, impacting their education.^71^, ^72^ People with vision impairment also experience reduced employment prospects and are more likely to have low paid work rather than professional jobs.^73^, ^74^ Beyond education and employment, vision impairment is linked to social exclusion including the experience of negative attitudes,^75^ violence and bullying,^76^ sexual assault,^77^ and loneliness.

The key to promoting the rights of people with vision impairment is to improve functional ability by increasing access to vision rehabilitation services and creating more inclusive environments through strengthening inclusive policies and laws, providing assistive technology, inclusive education and vocational training, advocacy, and creating accessible spaces. To ensure vision rehabilitation is considered as an integral component of eye care services in pursuit of universal health coverage, this Commission calls for adoption of a new definition of eye health, which involves maximised vision, ocular health, and functional ability, thereby contributing to overall health, wellbeing, social inclusion, and quality of life.

The International Classification of Functioning, Disability and Health model by WHO^67^ can help to contextualise the impact of vision impairment on a person's life, including walking, eating, education, employment, and social participation (appendix 1 p 16). This model illustrates the importance of environmental factors to maximise participation of people with vision impairment in society, including the opportunity to access vision rehabilitation.

Studies have shown^78^, ^79^, ^80^ that vision screening and provision of glasses helps to improve educational outcomes for children with vision impairment, and the provision of other types of assistive devices and reading aids is also likely to be effective. However, spectacles are not universally available and the ability to read and write using assistive technology, such as screen reading software or an electronic braille display, requires skills; the means to procure, purchase, and power the technology; and access to information in a compatible format. Compensatory skills learned in vision rehabilitation, such as orientation and mobility using a white cane, presupposes the necessary infrastructure of predictable paths and spaces in which the person can confidently move. These skills might be useful in one context but are not transferable to another—eg, in an urban setting with requisite infrastructure versus a rural setting without appropriate infrastructure. Clearly, much needs to be done to maximise the function and societal participation for people living with vision impairment. This is explored further in section 6.

The Commission calls for a more holistic approach to the health of people with vision impairment, who might have great difficulties with other health conditions and some might be perceived to no longer require eye care services.^81^, ^82^ We see integrated people-centred eye care as an opportunity to ensure that any reorientation of care promotes the rights of people to access eye care that they require during the life course, which is not limited to the condition causing their vision loss.

#### Vision impairment and quality of life

Assessment of quality of life describes an overall state of wellbeing from the individual's perspective. Health-related quality of life describes the degree to which health affects wellbeing, whereas vision-related quality of life reflects the degree to which vision affects wellbeing.^83^, ^84^ Health-related quality of life and vision-related quality of life are used to understand how vision impairment affects wellbeing from a person-centred perspective, and how this perspective is influenced by personal, social, and environmental contexts.

To summarise the extensive research on the impact of vision impairment, eye conditions, and ophthalmic interventions on the quality of life, we conducted an umbrella review of systematic reviews (appendix 1 p 17).^85^ In total, 69 systematic reviews were identified. Nine of those reviews evaluated the relationship between quality of life and vision impairment or specific eye conditions, such as age-related macular degeneration, glaucoma, or diabetic retinopathy, and all concluded that vision loss and eye disease were associated with poor quality of life outcomes. The remaining 60 reviews evaluated quality of life between groups receiving alternative ophthalmic interventions, active treatment (appendix 1 p 18), and controls who did not receive an ophthalmic intervention. 75% of ophthalmic interventions showed a positive impact on quality of life. Most notably, cataract surgery and anti-vascular endothelial growth factor treatment for age-related macular degeneration, diabetic macular oedema, and macular oedema secondary to other causes resulted in improved quality of life (appendix 1 p 18).

Our umbrella review reinforces the close relationship between good vision and enhanced quality of life and provides a strong argument for increased investment in eye health. The umbrella review also highlights that despite quality of life being commonly assessed in ophthalmic studies, definitions varied widely. This Commission calls for the development and the adoption of standardised and culturally sensitive measures of quality of life for eye health research, to better understand the effect of vision impairment and ophthalmic interventions from the patient's perspective.

#### Vision impairment and other health conditions

**Figure 5:**
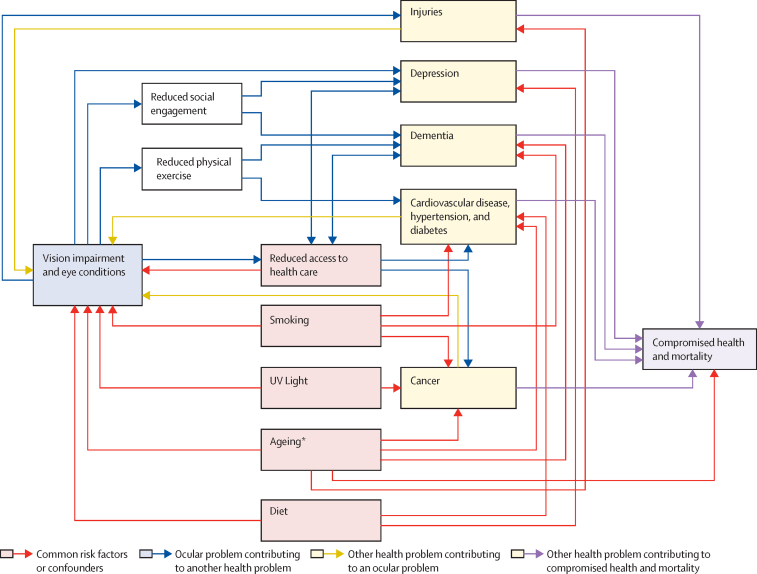
Relationships between vision impairment and general health These associations are derived from multiple literature reviews done by this Commission. *Hypothetical common degenerative pathways.

People with self-reported vision impairment have increased risk of some health conditions such as depression, dementia, cardiovascular disease, and lung cancer.^86^, ^87^, ^88^, ^89^ The causal relationship between vision impairment and other conditions is complex, but can be broadly summarised by three different pathways (figure 5); (1) vision impairment causes or exacerbates other conditions either directly, through injuries, or indirectly—eg, through reduced access to health care, limitations in physical activity, or increased social isolation; (2) vision impairment and other conditions share common risk factors—eg, smoking, poverty, reduced health-care access, ageing, or poor diet; and (3) systemic health problems can lead to vision impairment—eg, diabetes and diabetic retinopathy, cancer and ocular metastases, and dementia—limiting access to eye health services.

More than one pathway seems to underlie the association between vision impairment and other health conditions, making the pathways difficult to elucidate. Here, we summarise a rapid review led by one of the commissioners to investigate the relationship between vision impairment and mental health; and cardiovascular disease, respiratory disease, and cancer (appendix 1 p 19).

The rapid review found that vision impairment is likely to be linked to depressive illness. A meta-analysis of data from high-income and middle-income countries found that 25% of people with eye conditions also have pronounced depressive symptoms.^87^ Depressive symptoms have been shown to increase with more severe eye disease.^90^, ^91^ Another meta-analysis found a significant reduction in depression after cataract surgery.^92^ A review done by members of this Commission, in addition to the rapid review, found an association between vision impairment and dementia, and suggested that interventions to improve vision could possibly slow down cognitive decline (panel 1).

Panel 1 **Vision impairment and dementia**

In 2016, 43·8 million adults aged 60 years and older had dementia, with numbers doubling every 20 years (GBD Dementia Collaborators, 2019; Prince et al, 2013). The estimated global economic cost of dementia in 2018 was US$1 trillion (Wimo et al, 2017). Although there are few effective treatments (Mukaden et al, 2019), in the past decade vision impairment has emerged as a potential modifiable risk factor (Ehrlich et al, 2019; Swenor et al, 2019; Zheng et al, 2018; Brenowitz et al, 2019; Fischer et al, 2016; Schubert et al, 2017; Rogers et al, 2010; Nael et al, 2019; Maharani et al, 2018).

Longitudinal studies indicate that vision impairment might be a risk factor for dementia and accelerated cognitive decline (Swenor et al, 2019; Zheng et al, 2018; Fischer et al, 2016; Rogers et al, 2010; Tran et al, 2020). In Singapore, adults with vision impairment had an accelerated cognitive decline (Lim et al, 2020). In the USA, vision impairment was associated with 55% increased hazard of incident cognitive impairment and the effect of declining vision on future declines in Mini-Mental State Exam scores was significantly stronger than the reverse association (Swenor et al, 2019). Among more than 1000 women in the USA, incident dementia increased with vision impairment severity (Tran et al, 2020). In France, near vision impairment was associated with increased dementia risk at 4 years, although this association waned with longer follow-up (Nael et al, 2019). Preliminary data suggest that cataract surgery might decrease risk of cognitive decline. In an English longitudinal study of ageing, cognitive decline slowed down significantly (by 50%) following cataract surgery (Maharani et al, 2018). Together, these data provide strong evidence of an association between vision impairment and dementia.

Most research on vision impairment with cognitive function and dementia has been done in high-income countries, with few ongoing studies from LMICs (Lee et al, 2019; Kowal et al, 2012). However, there is a pressing need in all settings to test causal pathways that might underlie the association of vision impairment with cognitive decline and dementia. Although several hypotheses have been proposed to account for this relationship (Whitson et al, 2018), none have been rigorously tested. The postulated reason that dementia might be more prevalent in those with poor vision could be because of a single common underlying cause, such as neurodegenerative or microvascular disease. This reason is supported by the possible use of retinal imaging as a biomarker for dementia (Chan et al, 2019). Several indirect pathways have also been proposed. Poor vision is known to increase cognitive load (Pigeon et al, 2019), a stressor that might increase dementia risk. Another possibility is that decreased visual input could result in direct alteration of brain structure. Finally, vision impairment might exert its effect on dementia risk by limiting social and physical activity, factors that have separately been shown to elevate dementia risk (Tan et al, 2017; Reas et al, 2019; Rafnsson et al, 2020).

Vision impairment is possibly a modifiable risk factor for dementia because most of vision loss is preventable or treatable. However, vision impairment has not been widely recognised as such outside of the vision research community (Livingstone et al, 2020). Additional work is needed to ensure that various stakeholders invest in the importance of characterising cognitive trajectories of older adults with vision and multisensory impairments and in the testing of interventions to slow cognitive decline across diverse cultural and geographic contexts. References for this panel can be found in appendix 1 (p 105).

Indirect pathways might also link vision impairment with systemic conditions—eg, as a barrier to accessing health care. A UK study^93^ found that women with vision impairment were less likely to participate in breast and bowel cancer screening than women with no disabilities, after adjusting for confounding factors. Reduced physical activity might be another important factor; older adults in the USA with vision impairment took 26% fewer steps each day and spent 48% less time in moderate or vigorous physical activity than those with healthy vision.^94^, ^95^ Reduced physical activity is a leading risk factor for non-communicable eye diseases.

Ocular and general health conditions might share common risk factors. Smoking is associated with lung cancer, chronic obstructive pulmonary disease, stroke, coronary artery disease, dementia, and numerous eye conditions including cataract, age-related macular degeneration, diabetic retinopathy, and possibly glaucoma.^96^, ^97^, ^98^, ^99^ Sun exposure is associated with cataract and skin cancer.^100^ Poor diet, obesity, and low physical activity are common to systemic diseases and eye diseases. Consumption of vegetables, fruit, and micronutrients are protective for some cancers, cardiovascular disease, and depression. Similarly, a Mediterranean diet and some micronutrients might protect against cardiovascular disease and cataract, diabetic retinopathy, glaucoma, and age-related macular degeneration.^97^, ^101^, ^102^, ^103^

For cases in which systemic diseases directly cause vision impairment, the pathophysiology is often better understood. However, causative effects of general illness on vision impairment can also be mediated indirectly through the reverse of some of the indirect pathways. For example, poor systemic health might limit physical activity, which might increase risk of diabetic retinopathy^104^ and glaucoma progression.^105^ Preventive initiatives that reduce smoking, improve diet, and promote physical activity are likely to have shared benefits for general and ocular health. The question of whether interventions to ameliorate vision impairment can also improve general health is an important area for future investigation.

#### Dual sensory impairment

In a similar way to vision impairment, hearing impairment is also associated with age. Therefore, dual sensory impairment—ie, when these two conditions occur concurrently—is an important consideration for healthy ageing.^106^ This Commission did a scoping review to summarise dual sensory impairment definitions, prevalence, and the effect on people's lives (appendix 1 p 21). We found striking heterogeneity in the definitions of dual sensory impairment (67 variations in 151 studies), the age groups included, and prevalence estimates. Despite this heterogeneity, dual sensory impairment appears prevalent in older adults (increasing prevalence with age), and many studies reported that people with dual sensory impairment had worse physical and psychosocial health outcomes and reduced social participation compared with those who had only vision impairment or hearing impairment. In the context of an ageing population, this Commission calls for greater emphasis on dual sensory impairment, including a consensus on definitions and reporting, and collaborative efforts to advance the research, clinical care, and social inclusion for this population.

#### Vision and driving

Driving is a complex vision-dependent task with a risk of road traffic injury. SDG3 (good health and wellbeing) and SDG11 (improving the sustainability of cities and communities) include targets to reduce road traffic injury, which is the leading cause of death for children and young adults.^107^ This Commission did a systematic review of the relationship between vision impairment and driving, outlined in appendix 1 (pp 22–23). Findings from 115 studies showed that some causes of vision impairment, such as glaucoma and cataract, are associated with motor vehicle collisions and unsafe driving practices.^108^, ^109^ Interventions such as cataract surgery can reduce the risk of motor vehicle collisions,^56^, ^110^ whereas anti-vascular endothelial growth factor (for age-related macular degeneration or diabetic macular oedema) can enable continued participation in driving.^111^, ^112^ Most of these data were from high-income countries (88%). With the increasing reliance on motor vehicle transport, maintaining vision is essential for drivers to prevent road traffic injuries and promote independent mobility. This Commission calls for ready access to eye care services for drivers and evidence-based legislation to mitigate the risks associated with vision impairment and driving, particularly in LMICs.

#### Vision impairment and falls

Globally, a third of people aged over 65 years fall each year and falls are the leading cause of injury-related death among adults over 70 years.^113^, ^114^ This Commission did a systematic review to assess the relationship between vision impairment, ophthalmic interventions, and falls (appendix 1 pp 23–24). The main findings from 129 studies showed that vision impairment is an independent risk factor for falls among older adults and that timely access to ophthalmic interventions such as cataract surgery can reduce the risk of falls.^115^, ^116^, ^117^ On the basis of these findings, we call for vision to be included in risk assessment tools for falls and for eye care services to be better integrated with fall prevention efforts.

#### Vision impairment and mortality

Vision impairment seems to be associated with an increased risk of all-cause mortality.^118^, ^119^, ^120^, ^121^ Several explanations are possible for this association in relation to non-communicable eye diseases, mental health, and injuries (figure 5). This Commission did a systematic review and meta-analysis to contribute an updated appraisal of the literature, assessment of bias, and overall grading of the quality of evidence (appendix 1 p 24).^122^ We included studies that measured visual acuity and contained at least 1-year follow-up to assess all-cause mortality. A total of 28 studies representing 30 cohorts were included. Studies came from 12 countries in Africa, Asia, Australia, Europe, and North America and included 451 001 participants.

**Figure 6:**
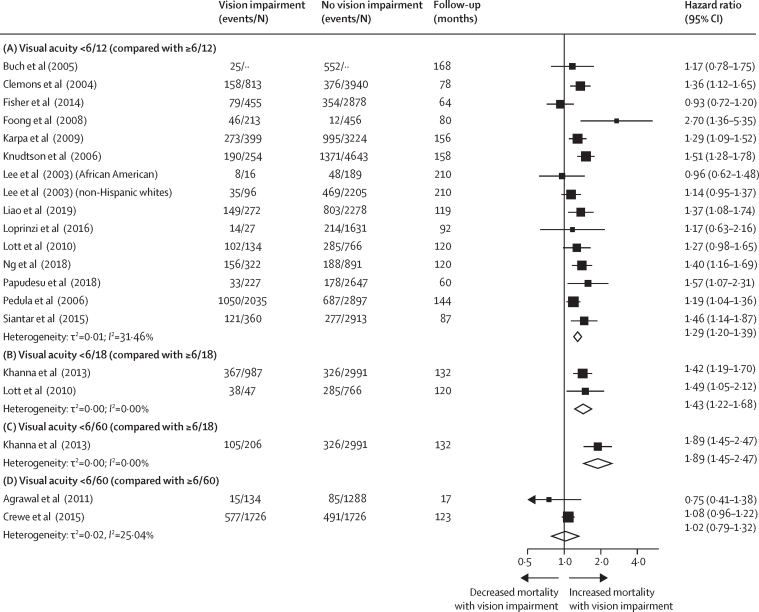
Vision impairment and mortality Random-effects meta-analysis results showing the maximally adjusted pooled hazard of mortality in adults with (A) mild vision impairment or worse (<6/12 compared with ≥6/12); (B) moderate vision impairment or worse (<6/18 compared with ≥6/18); (C) severe vision impairment or worse (<6/60 compared with ≥6/18); and (D) severe vision impairment or worse (<6/60 compared with ≥6/60). Events are defined as the number of participants in the study who died, and N is the total number of participants in the study. 12 cohorts that were included in the systematic review are not depicted in this figure for the following reasons: they used other vision impairment thresholds that could not be aggregated with these studies; they reported results per unit difference in visual acuity; they reported odds ratios or risk ratios that could not be pooled with HRs; or they compared a reference category of participants with good vision to participants with various vision impairment categories. References can be found in appendix 1 (p 100).

The primary meta-analysis included studies comparing mortality among participants with vision better or worse than the prespecified thresholds of 6/12, 6/18, and 6/60. Since age is a common risk factor for vision impairment and mortality, all measures of association in this meta-analysis were age-adjusted. Where available, we also selected estimates adjusted for other possible confounders such as smoking, diabetes, access to health care, and socioeconomic status. Figure 6 presents the results of this analysis. We found that the hazard of mortality was higher among those with visual acuity <6/12 (hazard ratio [HR] 1·29, 95% CI 1·20–1·39) and <6/18 (1·43, 1·22–1·68) compared to those with better vision. At the 6/60 threshold, the hazard of mortality was higher than for those with visual acuity of ≥6/18 (1·89, 1·45–2·47). However, no significant association was detected when comparing those with visual acuity better and worse than 6/60 (1·02, 0·79–1·32), probably because the reference group contained participants with a substantial degree of vision impairment (≥6/60). We evaluated the certainty of evidence using the Grades of Recommendation, Assessment, Development, and Evaluation (GRADE) framework and judged it to be of moderate certainty.^123^ Additional research is needed to better understand factors that modulate mortality risk among adults with vision impairment, and to more fully characterise risk in LMICs, where data remain scarce. Given the prevailing finding of an association between vision impairment and mortality, future calculations of disability-adjusted life-years might include years of life lost due to vision impairment, which could provide a more complete estimate of the overall global burden of vision impairment. The impact of vision impairment on mortality should drive action to address avoidable sight loss and reinforces the relevance of eye health to SDG3 (good health and wellbeing) and the SDGs in general.

## Section 3: Magnitude of eye disease

### Burden of global vision impairment in 2020

World Health Assembly Resolution 66/11 *Universal eye health: a global action plan 2014–19*, opened up a new opportunity for WHO member states to progress with their efforts to prevent vision impairment and strengthen vision loss rehabilitation in their countries.^124^ Central to this process is an understanding of the prevalence, magnitude, and causes of vision loss.

The Vision Loss Expert Group (VLEG) has worked with the Global Burden of Disease (GBD) collaborators since 2007 to produce global vision loss metrics. The VLEG published global estimates for vision loss and modelled temporal change for the first time in 2010,^125^, ^126^ and subsequently in 2015.^127^, ^128^ The results from 2015 were also published in the *World report on vision* by WHO.^2^ These analyses use definitions from the ICD-11 for distance and near vision impairment. The VLEG–GBD group collaborators have jointly published estimates for 2020, which we summarised here.^1^

Globally, of 7·79 billion people living in 2020, it is estimated that 43·28 million (95% uncertainty interval [UI] 37·57–48·36) are blind with presenting visual acuity of worse than 3/60 in the better eye and a crude all-age prevalence of 0·55% (0·48–0·61; table 3). Of these, 55% or 23·88 million (20·83–26·82) are women. The prevalence of blindness increases with age; most (77·7% or 33·61 million, 28·58–38·54) people with blindness are aged 50 years or older.

**Table 3:** Global number, crude prevalence, and age-adjusted prevalence of vision impairment in 2020

	**Blindness**			**Moderate and severe vision impairment**			**Mild vision impairment**		
	Number, millions	Crude prevalence	Age-standardised prevalence	Number, millions	Crude prevalence	Age-standardised prevalence	Number, millions	Crude prevalence	Age-standardised prevalence
**All**									
All ages	43·28 (37·57–48·36)	0·55% (0·48–0·61)	0·52% (0·46–0·59)	295·09 (267·32–324·60)	3·74% (3·39–4·12)	3·58% (3·24–3·92)	257·83 (232·66–285·34)	3·27% (2·95–3·62)	3·20% (2·89–3·54)
≥50 years	33·61 (28·58–38·54)	1·77% (1·51–2·03)	1·85% (1·57–2·11)	206·42 (182·37–233·16)	10·87% (9·61–12·28)	11·18% (9·90–12·61)	142·88 (122·12–163·00)	7·53% (6·43–8·59)	7·73% (6·62–8·82)
**Men**									
All ages	19·40 (16·95–21·70)	0·49% (0·43–0·55)	0·50% (0·44–0·56)	132·12 (119·77–145·68)	3·34% (3·03–3·68)	3·37% (3·05–3·70)	115·54 (104·37–127·95)	2·92% (2·64–3·23)	2·97% (2·68–3·28)
≥50 years	14·56 (12·38–16·73)	1·61% (1·37–1·85)	1·76% (1·49–2·01)	89·44 (78·70–101·43)	9·87% (8·68–11·19)	10·49% (9·30–11·83)	60·56 (51·60–69·25)	6·68% (5·69–7·64)	7·11% (6·07–8·11)
**Women**									
All ages	23·88 (20·83–26·82)	0·61% (0·53–0·68)	0·54% (0·47–0·61)	162·97 (147·43–179·21)	4·15% (3·75–4·56)	3·77% (3·42–4·13)	142·29 (128·45–157·36)	3·62% (3·27–4·00)	3·42% (3·10–3·78)
≥50 years	19·05 (16·22–21·82)	1·92% (1·64–2·20)	1·92% (1·63–2·20)	116·98 (103·72–131·93)	11·79% (10·46–13·30)	11·78% (10·44–13·30)	82·32 (70·52–93·83)	8·30% (7·11–9·46)	8·29% (7·10–9·45)

Data are n (95% uncertainty interval) or % (95% uncertainty interval). Data taken from VLEG–GBD, 2020.^1^

Moderate or severe vision impairment (MSVI) is defined as presenting visual acuity of worse than 6/18 to 3/60 in the better eye. MSVI is estimated to affect 295·09 million (95% UI 267·32–324·60) people, which is 3·74% (3·39–4·12) of the global population (table 3). A further 257·83 million (232·66–285·34) or 3·27% (2·95–3·62) have mild vision impairment, defined as presenting visual acuity of worse than 6/12 to 6/18 in the better eye. Globally, an estimated 509·69 million (371·11–666·66) people globally have near vision impairment from uncorrected presbyopia, representing 22·06% (15·52–29·62) of people aged 50 years and older. Similar to the gender imbalance in blindness, 55% or 162·97 million (147·43–179·21) of individuals who have MSVI and 55% or 142·29 million (128·45–157·36) of people with mild vision impairment are women.

**Figure 7:**
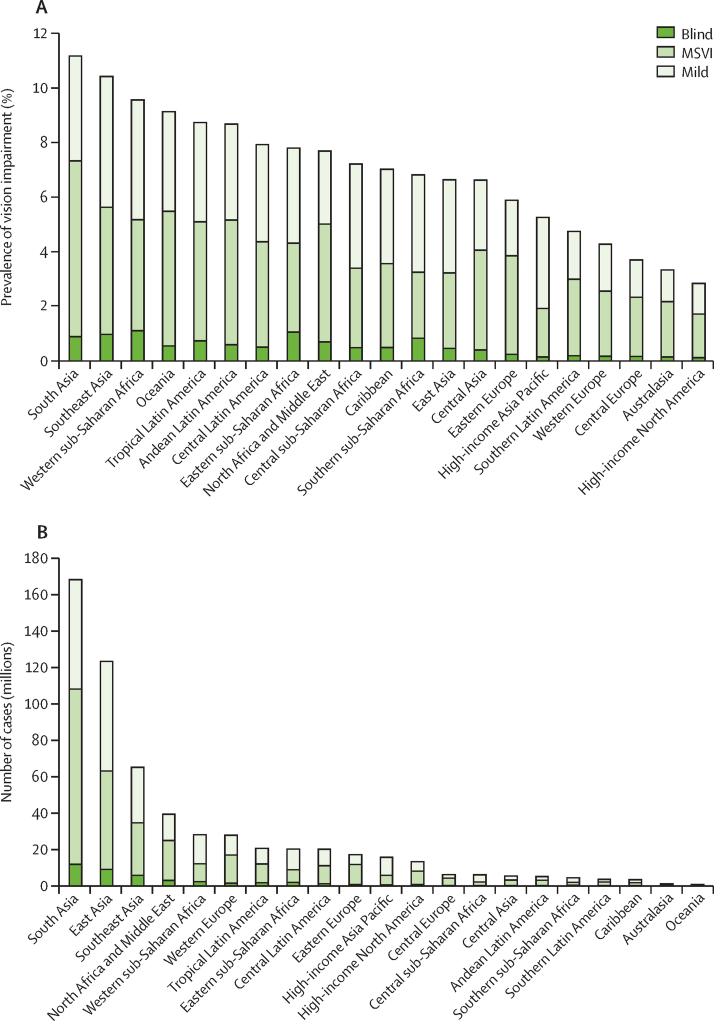
Vision impairment by Global Burden of Disease region (A) Age-standardised prevalence of vision impairment and (B) number of people with vision impairment. Data from VLEG–GBD.^1^ MSVI=moderate and severe vision impairment.

Globally, 91·75% of people who are blind (39·62 million, 95% UI 34·64–44·79) and 87·68% of those with MSVI (257·90 million, 231·87–285·81) live in LMICs. There are large interregional differences in crude and age-standardised prevalence of blindness and MSVI for 2020 (figure 7; appendix 1 p 26). Western sub-Saharan Africa has the highest age-standardised prevalence of blindness (1·11%, 0·95–1·26) and high-income North America has the lowest prevalence (0·12%, 0·11–0·14). South Asia has the highest age-standardised overall prevalence of MSVI (6·44%, 5·79–7·13). The largest number of blind people live in South Asia (11·9 million, 10·4–13·4), followed by east Asia (9·1 million, 7·9–10·3), and southeast Asia (5·9 million, 5·2–6·7), because of the large regional populations (figure 7; appendix 1 p 26). MSVI follows a similar pattern.

The *World report on vision*^2^ by WHO used estimates of people with distance vision impairment for 2015, provided by the VLEG, and combined these with an estimated 1·8 billion people with addressed (974 million) and unaddressed (826 million) near vision impairment due to presbyopia, derived from a different model, to reach an overall figure of 2·2 billion people with vision impairment.^127^, ^128^, ^129^ These 2015 estimates have now been superseded by 2020 estimates from the VLEG–GBD group.

In summary, for 2020, there are an estimated 596 million people with distance vision impairment and a further 510 million with uncorrected presbyopia. These estimates do not include people who have already received spectacles or contact lenses to correct distance refractive error or presbyopia, because reliable population-based data are scarce. However, this group probably represents a very large number of people who require ongoing services to meet their eye health needs.^129^, ^130^

### Causes of global vision impairment in 2020

The leading causes of blindness globally are cataract (17·01 million, 95% UI 14·40–19·93), uncorrected refractive error (3·70 million, 3·10–4·29), glaucoma (3·61 million, 2·81–4·42), age-related macular degeneration (1·85 million, 1·35–2·43), and diabetic retinopathy (1·07 million, 0·76–1·51; appendix 1 p 27).^131^ Notably, 37% (16·04 million, 14·00–18·06) of all blindness is attributable to a variety of other conditions. This group cannot be overlooked when focusing on the five leading causes of blindness. A person can have more than one cause of vision impairment; however, epidemiological studies tend to report only the primary cause.

In 2020, the leading causes of MSVI globally are uncorrected refractive error (157·49 million, 140·30–175·54), followed by cataract (83·48 million, 71·76–95·98), age-related macular degeneration (6·23 million, 5·04–7·59), glaucoma (4·14 million, 3·24–5·18), and diabetic retinopathy (3·28 million, 2·41–4·34; appendix 1 p 28).^1^

**Figure 8:**
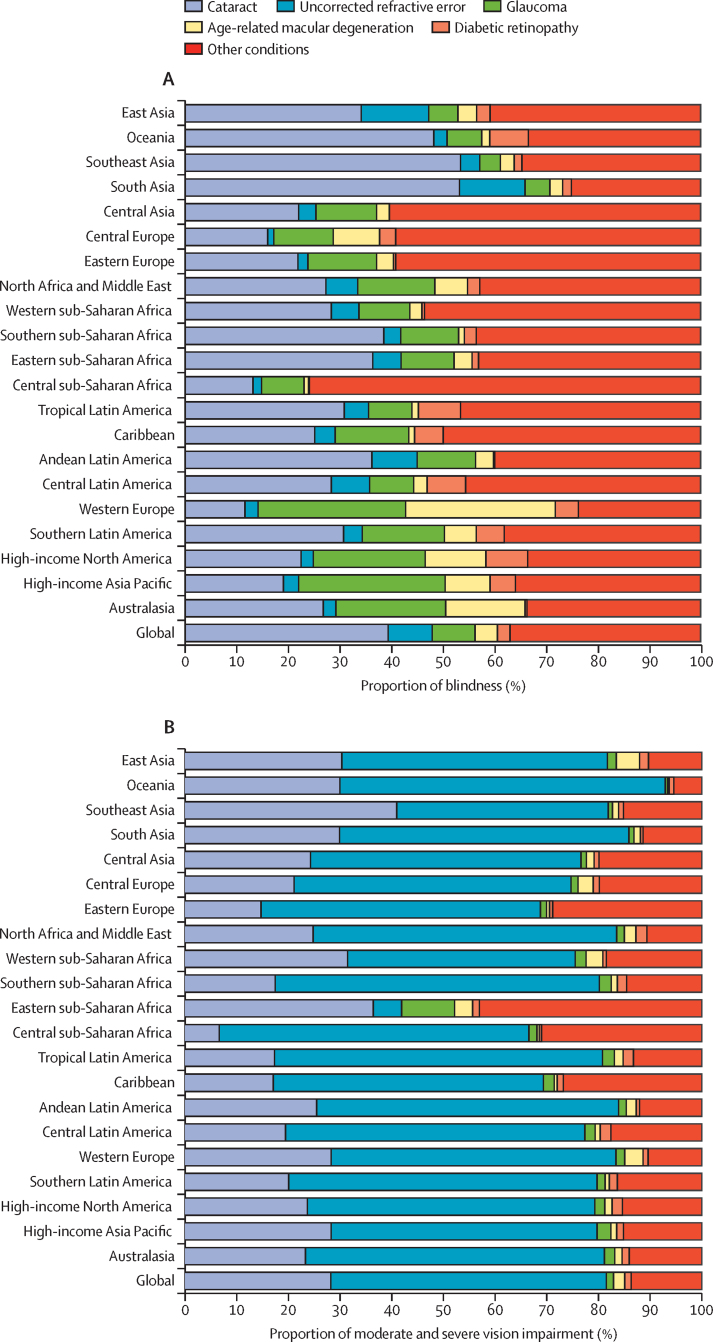
Causes of vision impairment by Global Burden of Disease region (A) Blindness and (B) moderate and severe vision impairment attributable to the five leading causes of vision impairment and other conditions (combined), all ages. Data are presented for the 21 Global Burden of Disease regions. Data from VLEG–GBD.^131^

Regions with particularly high prevalence of cataract blindness (as a proportion of all-cause blindness) include south Asia, Oceania, and southeast Asia, where cataract is responsible for around half of all blindness in 2020 (figure 8).^131^ In high-income regions, glaucoma and age-related macular degeneration account for a greater proportion of blindness than other regions. In all regions, uncorrected refractive error is responsible for most MSVI. In 2020, 77·3% of people with blindness and MSVI have an avoidable cause, defined as cataract or uncorrected refractive error; increasing to 90·9% if near vision impairment is included.

### Vision impairment in children

Data on vision impairment in children and adults younger than 40 years are scarce. Surveys need to be larger because blindness prevalence is lower in this age group (3 per 10 000 children in high-income countries, 10 per 10 000 in low-income countries) and some conditions appear as clusters. Measuring visual acuity of young children is challenging. As a result, population-based data are rare and mostly consist of school surveys. Mindful of these data limitations, the VLEG–GBD group has estimated that for 2020, 1·44 million children aged 0–14 years are blind (including uncorrected refractive error), 22·16 million have MSVI, and 46·60 million have mild vision impairment.

In 1990, WHO estimated that 1·5 million children (aged 0–15 years) worldwide were blind, excluding those with refractive errors.^132^ In 1999, WHO updated this estimate using an alternative method based on under-5 mortality rate as a proxy indicator for prevalence of blindness in children aged 0–15 years; reporting 1·4 million children.^133^ The rationale was that many causes of blindness in children also cause mortality, such as measles infection, vitamin A deficiency, meningitis, malaria, birth hypoxia, and prematurity. This prevalence was re-estimated at 1·14 million for 2015.^134^ Using the same method, this Commission re-estimated prevalence of blindness to be 1·02 million for 2020; representing a global prevalence of 4·8 per 10 000 children (appendix 1 pp 29–31). Further decline reflects a fall in under-5 mortality rate and stabilisation of the global population of children at 2·1 billion. South Asia and Western sub-Saharan Africa account for almost half (45·6%) of all children who are blind (appendix 1 p 30).

Overall, these two different estimation approaches have produced similar values. However, there is a pressing need for new methods to obtain more extensive survey data that would be representative of the population and would improve the estimates of vision impairment in children.

### Trends in vision impairment

**Figure 9:**
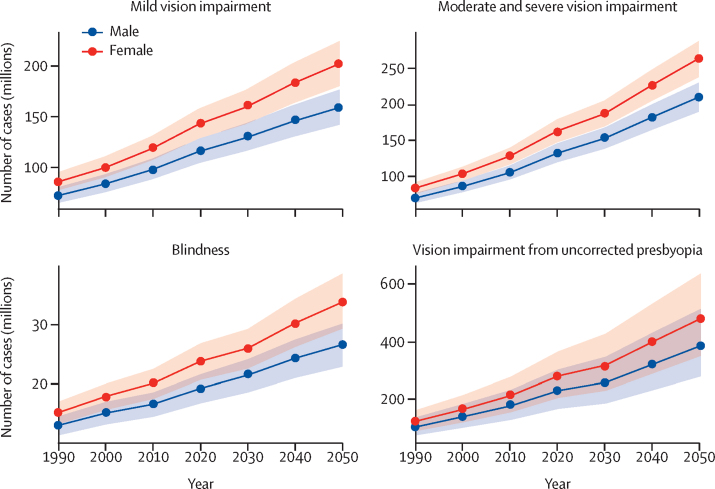
Forecast to 2050 of global cases of blindness and vision impairment by sex Reproduced from VLEG–GBD, 2020.^1^

To model temporal trends, the VLEG–GBD group^1^ generated forecasts of vision impairment prevalence for 1990–2019 (in 5-year increments), using age-specific prevalence as input into a regression model with year, region, and age as predictors. In the past 30 years, age-standardised prevalence of blindness has reduced by approximately 28·5%. By 2050, the number of people who are blind is predicted to increase to 61·05 million globally (95% UI 52·85–69·27). For MSVI, the number affected is expected to rise to 474·12 million (428·43–518·23), followed by 360·35 million (321·96–399·96) for mild vision impairment, and 865·67 million (628·79–1154·14) for vision impairment from uncorrected presbyopia (figure 9).

These trends are continuing—largely driven by population ageing and changes in disease patterns. UN projections^135^ indicate a substantial increase in the number of people aged 65 years and older over the next 30 years, from 700 million to 1·5 billion, with the largest increase in LMICs. Many conditions causing vision impairment become more prevalent with age.

**Figure 10:**
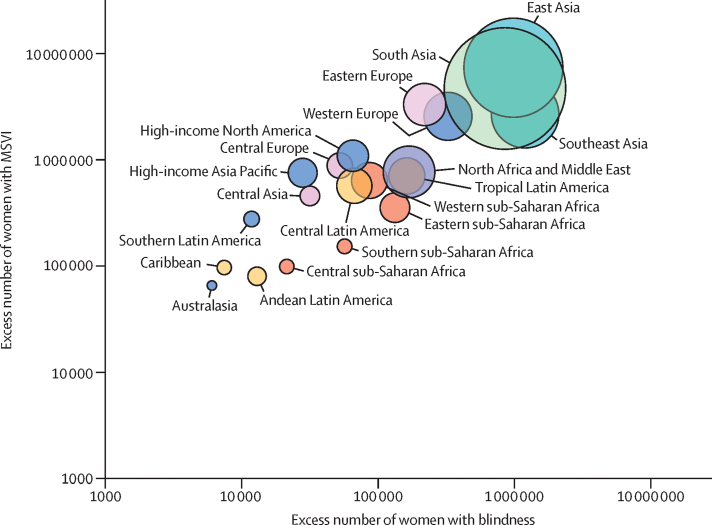
Women with blindness and MSVI Excess number of women over men, in adults (≥50 years). Data are plotted for the 21 Global Burden of Disease regions using a log scale. Size of the circle represents the total number of adults with blindness and MSVI in that region. Oceania has less than 1000 excess women with MSVI and was not plotted on this figure. Data available in appendix 1 (p 33), from VLEG–GBD.^1^ MSVI=moderate and severe vision impairment.

Projections suggest that the proportion of people with vision loss who are women will increase (figure 9).^1^ When actual numbers are assessed, there are more women than men living with blindness and MSVI in all regions of the world (figure 10). This gender imbalance can be attributed to demographic factors (women living longer than men) and social factors (women having reduced access to care). To adjust for demographic differences, age-standardised prevalence can be compared to provide a better estimate of gender inequity. Even after this adjustment, MSVI prevalence is higher in women in all regions of the world (except in two regions: central and southern sub-Saharan Africa; appendix 1 p 32). Men have lower age-standardised prevalence of blindness in less than half of world regions. Globally, for every 100 men with blindness or MSVI there are 108 women with blindness and 112 women with MSVI. The persistent gender differences after age-standardisation suggest that, in some settings, greater vision loss in women is socially determined. Some groups of women have difficulty accessing eye care, particularly in southeast Asia. These demographic and social factors have major implications for the pursuit of gender equity within universal health coverage.

There are several important risk factor and disease-specific trends that are changing the epidemiology of eye disease in many populations (panel 2). In the past three decades there has been substantial success in controlling trachoma and onchocerciasis. Environmental factors and educational practices, particularly in Asian populations, are believed to explain the pronounced increase in myopia in schoolchildren and young adults. Increasing urbanisation, less active lifestyles, and altered diets are contributing to a marked increase in diabetes, resulting in more people affected by diabetic retinopathy (appendix 1 p 33). Myopia and diabetic retinopathy require specific prevention and management strategies. New treatments for wet age-related macular degeneration have reduced the progression to blindness from this cause.

Panel 2 **The changing epidemiology of eye disease**

**Changing epidemiology of eye disease**

References for this panel can be found in appendix 1 (pp 106–08).

*Global increase of myopia*

Myopia is a major growing public health challenge. More than 2 billion people worldwide have a degree of myopia (≥–0·5 dioptres), 15% of whom have high myopia (≥–5 dioptres; Holden et al, 2016; WHO, 2015). In 2020, an estimated 161 million people worldwide were blind or had moderate to severe vision impairment from uncorrected refractive error, the leading cause of vision impairment (Bourne et al, 2020). By 2050, myopia is expected to affect 5 billion people, more than half of the projected global population (Holden et al, 2016), which will place an enormous burden on health services to provide spectacles and detect and manage serious eye diseases caused by high myopia (appendix 1 p 34). Uncorrected myopia and myopic macular degeneration, a major complication of high myopia, were responsible for approximately US$250 billion lost global productivity in 2015 (Naidoo et al, 2019).

Myopia is rapidly increasing worldwide (Holden et al, 2016; Naidoo et al, 2019; Koh et al, 2014; Vitale et al, 2008; Morgan et al, 2019). Myopia is considered a genetic condition with more than 200 associated genes, individually and in combination these genes contribute to only a small proportion of myopia (Tedja et al, 2019). Furthermore, the rapid global increase in school myopia cannot be explained by genetics alone, given the prevalence rise in a short timeframe (Morgan et al, 2019). Environmental factors and gene–environment interactions are thought to play a large role.

The large increase in myopia prevalence in east and southeast Asia since 1960 have mirrored rapid economic development and the associated educational and lifestyle changes in societies such as Hong Kong, Taiwan, Singapore, and South Korea (Siddiqi et al, 2001). The prevalence of myopia in people aged 20 years has risen from 20–30% in the mid-20th century to more than 80% nowadays (Morgan et al, 2019). China has also shown a rapid increase in myopia in the past few decades (Morgan et al, 2019; Sun et al, 2015). High myopia is becoming more prevalent and developing at a younger age (WHO, 2015), resulting in increased vision impairment and blindness from its complications (myopic macular degeneration, retinal detachment, and glaucoma; Liu et al, 2020; Fricke et al, 2018).

Educational pressures are substantially higher in east and southeast Asia than in other world regions (Morgan et al, 2019), which creates two interrelated environmental risk factors for myopia: a combination of increased near work activity (including screen time with increasing use of smart devices) and reduced outdoor activity (Dirani et al, 2019). With long periods of near work (Huang et al, 2015), children from east and southeast Asia spend less time outdoors than their peers in countries such as Australia (Rose et al, 2016; Wen et al, 2020). Clinical trials of increased time outdoors report a 25–50% reduction in incident myopia, although the precise mechanisms for these effects are not yet understood (Wu et al, 2018).

Finally, optical interventions such as spectacles and contact lenses reduce retinal defocus and slow myopia progression (Wildsoet et al, 2019). Pharmacological therapies such as low-dose atropine, have also been shown to slow myopia progression, although the underlying mechanism is unclear (Wildsoet et al, 2019; Walline et al, 2020). There are no good treatment options for myopic macular degeneration, although the treatment of one of its major complications, myopic choroidal neovascularisation, has improved with anti-vascular endothelial growth factor therapy (Cheung et al, 2017).

Reducing the growing societal burdens of uncorrected myopia will require complex strategies—ie, combining prevention methods with enhanced capacity to deliver high quality, affordable, and equitable refractive services (Ang et al, 2020). Investments will be substantial but are likely to be outweighed by the cost of inaction.

*Diabetic retinopathy*

Diabetic retinopathy is the most common microvascular complication of diabetes (Wong et al, 2016). The global prevalence of diabetes has tripled in the past 20 years. In 2019, diabetes prevalence was estimated at 9·3% (95% CI 7·4–12·1) of the global population aged 20–79 years, which is 463 million people, and is projected to reach 700 million by 2045 (Saeedi et al, 2019). The increase, mainly in type 2 diabetes, is attributed to dietary and lifestyle changes related to urbanisation, population growth, and increasing life expectancy (Saeedi et al, 2019; WHO, 2016). There are large regional differences in age-standardised prevalence of diabetes, with the highest in north Africa and the Middle East (12·2%, 8·3–16·1) and the lowest in sub-Saharan Africa (4·7%, 3·2–8·1). The greatest number of people with diabetes are in China, India, and the USA (Saeedi et al, 2019). In the next 25 years, sub-Saharan Africa is projected to have the largest percentage increase in diabetes (appendix 1 p 33). People with diabetes in LMICs are predominantly younger than 65 years and face a large unmet need for diabetes treatment, leading to inadequate glycaemic control and subsequent complications of diabetes (Manne-Goehler et al, 2019).

As the global prevalence of diabetes increases, the prevalence of vision loss from diabetic retinopathy will also increase. Although the crude prevalence of other causes of blindness decreased between 1990 and 2020, diabetic retinopathy-related blindness increased by 68%, mainly in LMICs (Flaxman et al, 2017; Leasher et al, 2016). A meta-analysis of population-based studies estimated that globally 34·6% (34·5–34·8) of people with diabetes have some degree of diabetic retinopathy and 10·2% (10·1–10·3) have vision-threatening diabetic retinopathy (Yau et al, 2012). Some findings show that the risk of developing diabetic retinopathy varies between populations although these data are scarce (Yau et al, 2012). Multiplying the estimated number of people with diabetes (Saeedi et al, 2019) by the global diabetic retinopathy prevalence provides an estimate of the magnitude of these conditions (Yau et al, 2012). In 2019, an estimated 160 million people had some form of diabetic retinopathy, of whom 47 million had vision-threatening diabetic retinopathy; by 2045 this number is projected to increase to 242 million (for diabetic retinopathy) and 71 million (for vision-threatening diabetic retinopathy; appendix 1 p 33).

In northern USA with good access to care, cumulative lifetime incidence of diabetic retinopathy for a person with type 1 diabetes was estimated at 90% in 1984 and for type 2 diabetes, approximately 50% (Klein et al, 1984 and 1984). The risk seems to be decreasing over time, particularly in high-income countries, probably because of improved risk factor control and advances in diagnostics over the past 30 years (Sabanayagam et al, 2019 and 2016).

Several factors influence the risk of developing diabetic retinopathy. Glycaemic control is a key factor, particularly in type 1 diabetes, and improved glycaemic control reduces the incident risk of diabetic retinopathy, progression, and sight loss (Diabetes Control and Complications Trial Research Group, 1993; UK Prospective Diabetes Study [UKPDS] Group, 1998). Increasing duration of diabetes is another major determinant for diabetic retinopathy, with incident risk rising to more than 75% in those living with diabetes for more than 15 years (Klein et al, 1984 and 1984). Hypertension is the third risk factor and might be more important in people with type 2 diabetes. Tight blood pressure control is thought to prevent the development of diabetic retinopathy, although its effect on diabetic retinopathy progression is less clear (UKPDS, 1998; Do et al, 2015). There appears to be genetic variation in diabetic retinopathy susceptibility, particularly among people with type 1 diabetes (Arar et al, 2008; Huang et al, 2011). Epigenetic factors might also be important in the pathophysiology (Kowluru et al, 2015).

Early detection and timely treatment of vision-threatening diabetic retinopathy can prevent 95% of blindness from this cause (Vujosevic et al, 2020). Strong links between general medical services and eye care are needed to ensure effective well-coordinated care. Treatment of vision-threatening diabetic retinopathy includes laser photocoagulation for retinopathy and maculopathy and careful consideration of an intravitreal injection with anti-vascular endothelial growth factor (Wong et al, 2018). Diabetic retinopathy screening and treatment programmes are considered by WHO as other recommended effective interventions in health care for non-communicable diseases (WHO, 2017). Low resources in many low-income and middle-income countries, to implement screening programmes and current cost-effectiveness ratios (outside the <$100 per disability-adjusted life-year range), means that reducing costs and increasing efficiency (through targeted screening, decreasing cost of treatment, and increasing completion rates) should be a high priority (Poore et al, 2015; Burgess et al, 2013). Additionally, a high proportion of people with diabetes are undiagnosed, with marked inequity seen within populations. In high-income countries, populations from disadvantaged groups (eg, highly deprived, ethnic minorities, and Indigenous people) are unable to equitably access services, leading to differential outcomes (Denniston et al, 2019; Foreman et al, 2017). New technologies, including teleophthalmology and artificial intelligence, offer potential new solutions (Vujosevic et al, 2020).

**Successful disease control programmes**

*Onchocerciasis control efforts*

Onchocerciasis, also known as river blindness, is a neglected tropical disease resulting in parasitic filarial infection caused by *Onchocerca volvulus*, which is transmitted by the *Simulium* blackfly. After an infected blackfly bite, adult worms (macrofilariae) develop in nodules under the skin. These worms release large numbers of microfilariae, which disperse around the body. The Global Burden of Disease study (Disease and Injury Incidence and Prevalence Collaborators, 2018) estimated that onchocerciasis infected 20·9 million people worldwide and caused vision loss in 1·15 million people from inflammatory damage to the cornea, retina, and optic nerve. The *Simulium* blackfly requires fast-flowing well-oxygenated water for its lifecycle, so larvicide treatment of fly breeding sites can interrupt transmission. Ivermectin can reduce symptoms and break transmission by killing microfilariae and stopping their release from adult female worms.

During the past five decades, four regionally focused control programmes have been developed. The Onchocerciasis Control Programme between 1974 and 2002, was launched by the World Bank, WHO, UNDP, and the Food and Agriculture Organization to control onchocerciasis transmission by larvicide spraying of rivers in seven (later expanded to 11) west African countries. The African Programme for Onchocerciasis Control between 1994 and 2015 was launched by the World Bank and WHO in 19 (other) African endemic countries and to assist the original 11 countries, using community directed treatment with ivermectin to control (and later eliminate) the disease. The Onchocerciasis Elimination Programme for the Americas was launched by WHO in 1992 to eliminate transmission of onchocerciasis in six endemic countries in central and south America using twice a year distribution of ivermectin. The Expanded Special Project for the Elimination of Neglected Tropical Diseases was established in 2016 by WHO Regional Office for Africa, member states, and neglected tropical disease partners to mobilise political, technical, and financial resources to accelerate the elimination of the five most prevalent neglected tropical diseases.

These programmes led to several key achievements. Infection has been prevented or treated and vision loss was prevented in more than 80 million people in 27 African countries and six countries of the Americas. Transmission of *O volvulus* has been eliminated in four of six endemic countries in the Americas and some areas in Africa. Programmes have supported health system capacity building for control of neglected tropical diseases. Arable land in Africa has been reclaimed for agriculture and economic development (WHO, 2008).

A key component of success of the African programme was implementation research, which identified that community-directed drug administration was an effective delivery strategy for ivermectin. A further benefit was the provision of extensive capacity building opportunities for African researchers, who are now global leaders in their field.

*Lessons from onchocerciasis control*

Economic impact research and advocacy led to the donation of ivermectin by Merck in 1988—“as much as is needed for as long as it is needed”. This donation was crucial in creating an international public–private partnership to improve the health of affected communities.

Regional programmes enabled efficient use of technical resources and cross-border treatment of endemic communities.

The community-directed distribution of ivermectin increased coverage, while keeping the cost of distribution low and improving coverage and sustainability.

The partnership between endemic communities, ministries of health, UN agencies, international and local non-governmental organisations, agricultural agencies, and the pharmaceutical industry has become a model for control of onchocerciasis and other neglected tropical diseases.

Implementation research built into these programmes to address issues of disease transmission, resistance, coverage, cost-effectiveness, adverse effects, and disability, has been crucial to their successful implementation (WHO, 2008).

*Eliminating trachoma as a public health problem*

Trachoma is the leading infectious cause of blindness, recognised as a clinical entity for many years, and responsible for vision impairment or blindness in 1·9 million people (Bourne et al, 2013). The pathogen, *Chlamydia trachomatis*, was first isolated in China in 1955 (Tang et al, 1957). The discovery that one oral dose of the antibiotic azithromycin was effective against *C trachomatis* reinvigorated efforts to control and eliminate the disease (Bailey et al, 1993). The WHO Alliance for the Global Elimination of Trachoma by 2020 was established in 1996 (WHO, 1997). Elimination of blindness from trachoma was included in VISION 2020 (WHO, 2000 and 2003). Since then, as a result of a well-coordinated effort between health ministries, donors, and implementing partners, the number of people at risk of blindness from trachoma has decreased by 91% from an estimated 1·5 billion in 2002 to under 137 million in 2020, and the number of people requiring surgery for trichiasis has decreased by 74% from 7·6 million in 2002 to 2 million in 2020 (WHO, 2020). Nine countries have been validated by WHO as having eliminated trachoma as a public health problem (WHO, 2020). Many other disease communities have monitored the trachoma community.

*Four crucial factors might underlie success*

First, normative guidance from WHO translated into preferred practices by the International Coalition for Trachoma Control, based on the SAFE strategy (surgery for trichiasis, antibiotic treatment, facial cleanliness, and environmental improvement).The SAFE strategy was formally adopted by WHO member states in 1998 in the World Health Assembly Resolution 51.11 (WHO, 1998). Conclusions and recommendations from the annual meeting of the Alliance for the Global Elimination of Trachoma are translated into complementary action plans by health ministries, academics, donors, and implementing partners.

Second, partnerships fostered within the Alliance for the Global Elimination of Trachoma and International Coalition for Trachoma Control, supporting government-led implementation of the SAFE strategy in endemic countries. Partners come together to strengthen national capacity; to coordinate the efforts of governments with those of funders, implementing partners, and researchers; and to mobilise resources from various donors to fill gaps in the global programme (Courtright et al, 2018).

Third, committed donors and a data-driven medicine donation programme with a strong country-led accountability framework. Pfizer's commitment to supply azithromycin for trachoma elimination is coupled with robust stewardship of the donation by the International Trachoma Initiative. Many other committed and motivated donors also support these programmes.

Fourth, credible national plans to achieve elimination. Through trachoma action plans and neglected tropical disease plans, health ministries have crafted ambitious but attainable targets, facilitating the political decision making that underlies progress in public health. Crucial decisions include commitments to scale up interventions, standardise approaches, allocate domestic resources, and recruit new stakeholders.

Now, in the last mile of trachoma elimination, the trachoma community has built on its strong foundations and strengthened global collaboration with four key enabling factors.

(1) A shared passion and ambition to target gaps and achieve a world free of trachoma.
(2) Use of high-quality prevalence data generated by the Global Trachoma Mapping Project and Tropical Data, coupled with rigorous research overseen by the WHO Network of Collaborating Centres for Trachoma to underpin evidence for interventions and facilitate refinement in programme delivery (Solomon et al, 2015 and 2018; WHO, 2017).
(3) Innovation for continuous improvement and accelerating the global programme, including new tools for active trachoma diagnosis and trichiasis surgeon training and practice (Solomon et al, 2018; Gower et al, 2014; Merbs et al, 2012); open online courses for programme managers (London School of Hygiene & Tropical Medicine, 2020); streamlining of reporting for endemic countries through the Trachoma Elimination Monitoring Form jointly managed by WHO and the International Trachoma Initiative; and transparent updates about the progress of antibiotic shipments through the International Trachoma Initiative azithromycin tracker.
(4) Integration by pursuing mutually advantageous opportunities to engage with interdependent sectors (water, sanitation, and hygiene, education, vision, and neglected tropical diseases).

WHO noted in an analysis of progress towards trachoma elimination, the 2021–30 Neglected Tropical Disease Roadmap, that key components (technical progress, strategy, service delivery) were in place, with enabling factors producing amplification of collective efforts (WHO, 2020). The essential actions required to achieve global elimination of trachoma as a public health problem are: continued investment in implementation, additional research to better understand transmission and how to limit transmission in different settings, improvements in surgical quality, strengthening surveillance capacity to monitor possible recrudescence, and ongoing advocacy for domestic financing (Habtamu et al, 2016; Last et al, 2020). These actions for trachoma overlap substantially with recommendations for the vision sector in the WHO *World report on vision*, reminding communities that integrated people-centred eye care is a cornerstone to end trachoma.

### Non-visually impairing ocular conditions

Population-based eye health surveys typically quantify vision impairment at an individual level. Surveys usually do not report conditions that have the potential to cause vision loss later in life, such as early glaucoma or diabetic retinopathy, or conditions that typically do not cause vision impairment. Surveys report data for the better seeing eye, overlooking individuals with monocular problems, which greatly underestimates the magnitude of eye conditions and service needs.

Broadly there are three groups of people that need eye care services: the first group are people with manifest or corrected vision impairment who need ongoing care, including rehabilitative services; the second group are people with early stage disease or at high risk of eye conditions that might cause vision impairment in later life, who need ongoing care to prevent the disease or its progression; and the third group are people with symptomatic conditions that typically do not cause vision impairment but require services.

The first group is partly quantified in population-based surveys measuring vision loss and summarised by the 2020 VLEG–GBD data.^1^ There is also a large but poorly quantified group of people, mostly with corrected refractive error or presbyopia, who need ongoing intermittent eye checks. Recording uncorrected visual acuity, in addition to visual acuity, in future population-based surveys will help to quantify this group.^2^ Data on the younger age groups are particularly scarce.

For the second group, vision-impairment-based surveys and analyses underestimate common diseases because they do not report conditions in people without impaired vision.^136^, ^137^, ^138^ Using population-based comprehensive survey data, it has been estimated that 76 million people are living with glaucoma worldwide in 2020.^136^ However, the latest 2020 global estimates for blindness (3·61 million) and MSVI (4·13 million) attributed to glaucoma are substantially lower (appendix 1 pp 27–28). Although these estimates are derived using different approaches, clearly, most people with glaucoma are not included in GBD estimates because their central visual acuity is preserved. Glaucoma constricts the visual field and even mild forms cause problems with reading, walking, and increase the risk of falling. Most people living with glaucoma have not been diagnosed and are not receiving treatment.^139^

In 2020, 4·4 million people were estimated to be blind or have MSVI from diabetic retinopathy (appendix 1 pp 27–28). However, this number is small compared with the 160 million people who have any diabetic retinopathy, or the estimated 463 million people living with diabetes in 2019, all of whom require regular access to eye care services to reduce long-term risk of vision loss (appendix 1 p 33).^138^, ^140^ People with vision impairment represent only a small proportion of all people who need these services. The second group also includes high-risk populations that require screening services for early disease detection. For example, premature infants at risk of retinopathy or older (≥40 years) African Americans, at risk of glaucoma.

The third group includes conditions that rarely affect vision, such as conjunctivitis and dry eyes. Existing data suggest that the number of people affected are considerable. For example, a large UK database^141^ of 3 million people showed that ocular problems without vision impairment account for 88·1% of all general practitioner consultations related to eye health and 68·9% of referrals. Data from secondary eye units, which are part of the Aravind Eye Care System in India, show a similar pattern; only 41% had visual acuity of worse than 6/12 in the worse eye and 28% in the better eye (appendix 1 p 35). Similarly, data from a secondary eye unit in Kenya found that less than half of presentations were for conditions associated with vision impairment.^6^

The implications of these findings are important and require action. There is a need for standard terminology and robust definitions to measure the magnitude of eye disease that does not impair vision; data need to be collected and analysed to assess this magnitude; the impact of non-visually impairing eye disease on the quality of life and its economic consequences need to be assessed; and the full magnitude and impact of all eye disease needs to inform health-care planning to serve the population and improve eye health.

### Quantifying the magnitude of eye disease

Population-based surveys provide estimates of disease prevalence, service coverage, and outcomes. These data are needed to support service planning, resourcing, and monitoring, and can be aggregated to provide regional or global estimates.^1^, ^127^, ^128^, ^131^, ^142^ Broadly, there are two approaches to eye health surveys, comprehensive and rapid (appendix 1 pp 36–37). Comprehensive eye health surveys typically include an in-depth ophthalmic examination with imaging for independent retinal grading and data on risk factors. Several rapid assessment methods are available.^143^ The most used method is the Rapid Assessment of Avoidable Blindness (RAAB), for people aged 50 years and older. The most applicable approach balances the required epidemiological detail and the available resources.

**Figure 11:**
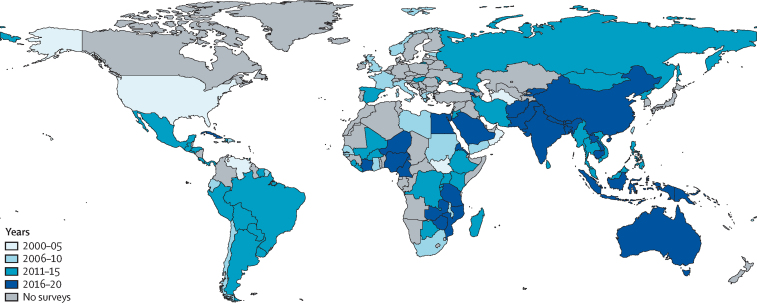
Most recent population-based eye health surveys globally All surveys (rapid and comprehensive, national or subnational) done since 2000. Not applicable indicates that no surveys were done. Data from VLEG–GBD,^1^ and the Rapid Assessment of Avoidable Blindness Repository.

Developing reliable estimates of eye disease depends on good quality survey data. This Commission has analysed the distribution of surveys since 2000 (appendix 1 pp 38–42). Most were RAAB surveys. Some regions have scarce or old data (eg, western and central sub-Saharan Africa, central Asia, central Europe, North America; figure 11). Where there are few data for a region, modelling approaches are sometimes used to fill in the gaps.

Rapid and comprehensive techniques have predominantly reported presenting distance visual acuity (ie, with available correction). However, following the WHO *World report on vision*, future eye health surveys are strongly encouraged to measure uncorrected visual acuity as well, and acknowledge met and unmet need for refractive services in addition to presenting vision impairment prevalence.^2^ With new focus on effective refractive error coverage as a core indicator, accurate diagnosis of refractive error needs to be ensured. The accuracy of pinhole correction to identify uncorrected refractive error (a common survey approach), might be variable as opposed to subjective refraction, on account of the quality, the type of pinhole occluder used, or other pathology.^144^ Standardisation of procedures and equipment is required.

### Reporting eye health surveys

The *World report on vision* by WHO^2^ highlighted the need for greater methodological and reporting standardisation of eye health surveys. Although generic tools such as the Strengthening The Reporting of Observational Studies in Epidemiology (STROBE) checklist and the risk of bias assessment exist, none are specific to eye health surveys,^145^ which is a missed opportunity to promote robust study design, reduce risk of bias, and emphasise complete reporting. In response, we did a two-round Delphi survey to identify key methodological issues and develop a checklist to improve the design, conduct, and reporting of vision impairment surveys, including minimising bias (appendix 1 p 43). Generic STROBE checklist items were endorsed as essential for high quality reporting in comprehensive and rapid surveys. We identified several STROBE items that were frequently poorly reported, several vision-specific extensions, and a need for better guidance to enhance the standardisation and quality of future eye health surveys (panel 3).

Panel 3 **Key recommendations for improving population-based vision impairment surveys**

**Improving survey design and conduct**

• Standardise equipment, participant recruitment strategy, and team training
• Measure vision at a constant distance using a vision chart with high contrast, crowded, standardised optotypes
• Assess quality assurance (survey staff accuracy of measuring vision) using Bland-Altman Limits of Agreement to test visual acuity or kappa, allowing to compare vision impairment categories
• Address declining response rates globally by updating sample size calculations, sampling, and analytical approaches (eg, weighting by cluster non-response)
• Incorporate standard socioeconomic position indices (eg, Equity Tool) and other known associated demographic or equity factors (eg, disability, distance from services)

**Improving completeness of reporting**

*Improve application of Strengthening The Reporting of Observational Studies in Epidemiology (STROBE) items*

• Item 6 (participant recruitment): report participant recruitment strategy in full
• Item 12 and 16 (statistical methods): analyse and report crude and adjusted prevalence estimates with measures of uncertainty (eg, 95% CI), to account for the sampling design
• Item 13 (results): report the number of units (eg, villages, households, people) sampled at each stage (assessed for eligibility, recruited, examined) with numbers of not contactable participants and reasons for non-participation
• Item 14 (results): report missing data for each variable of interest

*Extensions of STROBE items for vision impairment surveys*

• Items 7 and 11 (variables): define and report vision categories and unit of analysis (eg, best eye or binocular), eye disease case definitions, avoidable vision loss, and any risk exposures of interest
• Item 8 (details of measurement): for vision testing, report chart type (scale, optotype), approach to maintaining constant test distance, location of testing, and whether the examination protocol differed for different subgroups (eg, children or adults with cognitive impairment); if collecting clinical images, report details of reading centre personnel, training and grading or agreement criteria

**Major risks of bias to minimise and report**

• Wrong sample size bias—sample too small to yield precise prevalence estimates or too large and resource inefficient—eg, if expected prevalence of blindness is lower than sample prevalence
• Selection bias—the sampled population not accurately representing the target population, resulting in erroneous inferences about the population's magnitude of vision impairment—eg, if the random sampling and enumeration procedures are not rigorously observed, a convenience sample might be recruited, yielding an apparently good response rate, who differ in important but unmeasurable ways from the true target population
• Non-responder bias—non-responders might differ substantially from responders—eg, older people (in whom vision impairment is more prevalent) or people not working on account of vision impairment, are more likely to be available for examination than younger employed people
• Diagnostic purity bias—narrow case definitions—eg, exclusion of ocular comorbidities might lead to misleading cause attribution, a tendency to over-report causes most easily identified from simple examination protocols and to underreport posterior segment disease
• Missing clinical data bias—missing data might differentially impact reliability of results in different subgroups—eg, likelihood of agreeing to pupil dilation might vary between different clinical subgroups, affecting the ability to collect data

### Disability weights for vision impairment

How important are blindness and vision loss to an individual, compared with other health morbidities? Disability weights (where 0 equals a state of full health and 1 equals death) and disability-adjusted life-years (DALYs) provide an answer. Nine studies (appendix 1 p 44), published between 1994 and 2015, used various approaches to estimate the disability weight associated with blindness, which ranged widely from 0·600 (in 1994) to 0·173 (in 2015).

There are multiple explanations for this variation.^146^ First, the construct being measured shifted over time from loss of wellbeing (disability) to loss of health. Second, studies described the impact on health states differently.^147^ Third, studies framed their questions differently. Fourth, some studies used expert panels exclusively from high-income countries, while others sought responses from large samples of the general public internationally. There are likely to be considerable regional differences in the impact of vision loss on quality of life. Finally, studies used different valuation methods, which included person trade-off, paired comparison, population health equivalence, and the visual analogue scale.

The GBD 2010 study disability weights sparked a lively debate.^148^, ^149^ By reporting a blindness disability weight reduction from 0·600 to 0·195, the apparent global importance of cataracts fell substantially.^150^, ^151^ WHO subsequently incorporated health state data to calculate another blindness disability weight of 0·338.^152^ In view of the wide range and major implications that this figure has on how vision loss is valued, further empirical research is urgently needed to understand societal valuations of vision impairment and reach a broad, evidence-based consensus of weights that should be applied, possibly allowing for the use of different weights in different settings.^146^

## Section 4: The economics of vision

As a society, what value do we place on vision? How much are we willing to invest, relative to the other demands on finite health resources, to ensure that people can access services? These questions are especially complex in 2021 as the world continues to battle the COVID-19 pandemic. In response, this Commission did a systematic literature review of the economic costs of vision impairment and its major causes to identify and summarise what is currently known. Using studies identified in this review, we then determined a new estimate for global and regional productivity losses from vision impairment, and summarised studies reporting cost-effectiveness ratios of cataract surgery and refractive error correction.

### Systematic review of eye health economics

We searched the literature from Jan 1, 2000, to Dec 31, 2019, for partial economic studies (eg, cost of illness and economic burden of disease) and full economic studies (eg, cost-effectiveness and cost-benefit) reporting the economic cost of vision impairment or the cost of evaluating interventions for seven leading causes: cataract, refractive error, glaucoma, diabetic retinopathy, age-related macular degeneration, corneal opacity, and trachoma (appendix 1 pp 45–49).^153^ We excluded studies reporting only incremental costs, benefits, or cost-effectiveness ratios.

In total, 138 publications met these criteria, with scarce information about many regions and conditions. The geographical distribution and focus of each publication are shown in appendix 1 (p 48). We identified fourteen studies that reported global estimates related to eye health economics. Most (72%) regional estimates were from high-income countries. Study types were heterogeneous (appendix 1 p 49). The main economic study perspectives of vision impairment were societal (35%), health system (18%), or third-party payer (17%). Most studies (90%) used a prevalence-based approach for estimation.

Economic literature on eye health has multiple limitations and great uncertainty. First, many studies were not comprehensive in approach; most (70%) considered only one cost category (direct health-care costs, direct non-health-care costs, productivity loss, informal care, or intangible costs). Heterogeneity limits comparability and the few cost items included in many studies probably underestimate the full cost of treatment and rehabilitation. Second, vision impairment severity or disease stage are not standardised. Third, studies tended to be small and unrepresentative. Fourth, productivity loss estimates were limited in scope and generally made major, largely unsupported assumptions about the productivity and proportion of people with vision impairment who work. Finally, few studies did sensitivity analysis or addressed uncertainty. These limitations mean that previous estimates might have substantially underestimated or overestimated the economic impact of vision impairment, which limits the usefulness of cost-of-illness estimates and possibly led to flawed policy prioritisation decisions.

### Global productivity losses from vision impairment

We have already explored the limitations that vision impairment can place on an individual's ability to engage in the workplace, ensuing loss of productivity and income. Addressing avoidable vision impairment and enabling people with permanent impairment to access and function well in the workplace, increases productivity. For example, a trial^29^ in India found that provision of near vision spectacles resulted in significantly increased productivity of people with presbyopia in harvesting tea (appendix 2 p 4).^29^

Our systematic review identified 37 articles on productivity loss (appendix 1 p 49). Studies reported different combinations of components (absenteeism, presenteeism, wage reduction, reduction in employment, and premature mortality), although reduction in employment was the most frequently reported component. Assumptions around reduced employment attributable to vision impairment were variable, predominantly based on scarce data and diverse methods. Most studies focused on a single condition or a few regions. There are only three previous global productivity loss estimates for vision impairment and blindness, and they are compared in appendix 1 (p 50).^154^, ^155^, ^156^

This Commission has established a new estimate for global and regional productivity losses from unaddressed vision impairment (appendix 1 pp 51–53). We systematically searched for employment gap data for people with vision impairment, identifying 11 reports, with limited geographical distribution (appendix 1 p 52). Productivity loss calculations included the number of people with blindness or MSVI of working-age (15–64 years) in 2020, national employment rates for 2018, per-capita gross domestic product for 2018, and employment gap data published between 2004 and 2018. Values are presented for 2018 in US$, adjusted for purchasing power parity.

**Figure 12:**
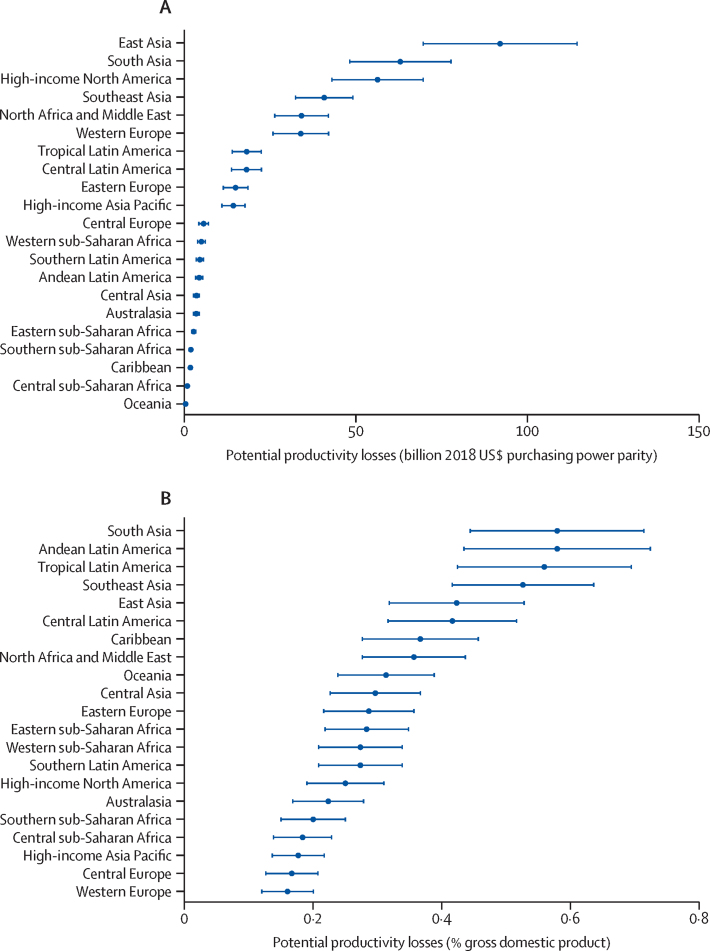
Productivity losses resulting from vision impairment Estimates made for 2020 using the number of people who were blind or had moderate and severe vision impairment, an employment gap of 30·2%, 2018 employment rates, and gross domestic product for (A) productivity loss, and (B) as a percentage of gross domestic product.

In 2020, it was estimated that globally, 18·1 million (95% UI 14·4–22·6) people are blind and 142·6 million (112·5–179·6) are living with MSVI in the working-age group. The overall relative reduction in employment of people with blindness or MSVI was estimated to be 30·2%. We estimated that the global annual productivity loss was $410·7 billion (95% uncertainty interval $322·1–518·7 billion), which represents 0·3% of the gross domestic product of the 21 GBD regions in 2018. Potential productivity losses were estimated at $43·6 billion, ($34·4–54·5 billion) attributable to blindness, and $367·1 billion ($287·7–464·2 billion) attributable to MSVI. Figure 12 shows the productivity losses for each GBD region. Productivity losses were highest in east Asia ($90·4 billion, $70·5–115·3 billion). Productivity losses as a proportion of gross domestic product was highest in south Asia (0·6%).

Our new analysis provides a robust estimate of the global annual productivity loss attributable to vision impairment. For the first time to our knowledge, regional VLEG–GBD estimates were used to estimate the number of people with vision impairment in the working-age group and regional employment reduction variables derived from a literature review. However, additional productivity loss components were not included in our analysis because reliable data at country and regional level remain scarce. The components that were not included are absenteeism and presenteeism (reduced productivity in the working place), premature mortality, people older than 64 years, productivity losses of caregivers, and value of time lost from unpaid or informal labour activities. Data do not sufficiently differentiate between reduction in employment for blindness and MSVI. Therefore, we only included components for which sufficient evidence was available, and consequently the magnitude of productivity loss could have been underestimated.

Overall, this analysis indicates that blindness and MSVI have substantial economic impact worldwide and highlights the opportunity to unlock human potential by addressing avoidable vision impairment and providing rehabilitation services to enable people to work.

### Cost-effectiveness of eye health interventions

As an adjunct to the systematic review, we examined cost-effectiveness ratios of eye health services and interventions (appendix 1 pp 54–57). We identified 182 reports for 16 ophthalmic conditions. The most frequently studied interventions addressed age-related macular degeneration,^53^ glaucoma,^32^ cataract,^29^ and diabetic retinopathy.^27^ We focused on cataract and uncorrected refractive error interventions and only included studies reporting health benefits using DALYs or quality-adjusted life-years (QALYs). Costs were adjusted to 2018 values expressed in US$ purchasing power parity.

**Figure 13:**
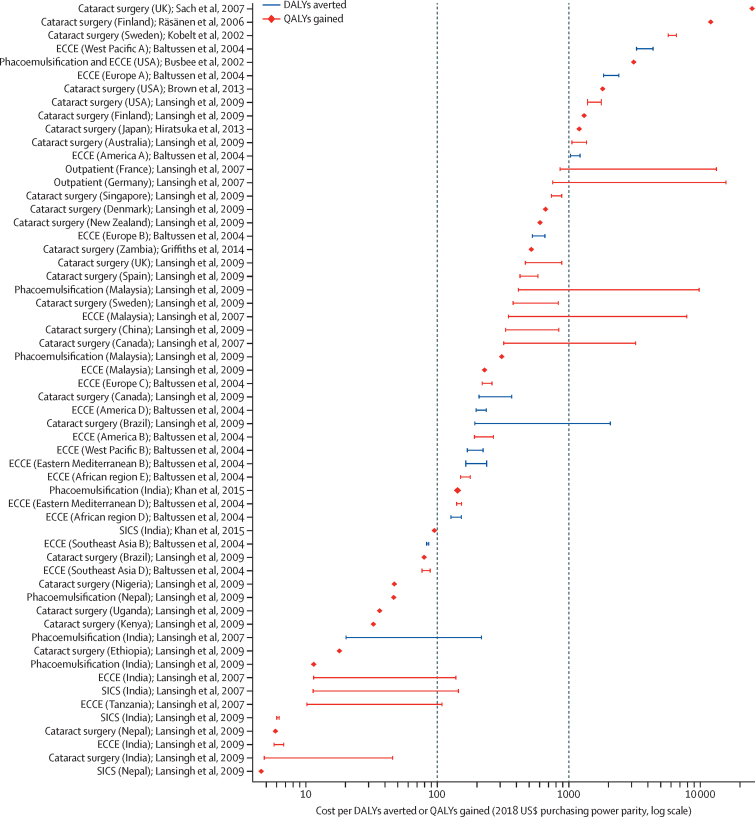
Cost-effectiveness ratios for cataract surgery Studies reporting either DALYs averted or QALYs gained. Costs have been inflated to 2018 levels and converted to US$ purchasing power parity. Studies which provide only a single estimate are plotted as a diamond. For studies reporting more than one estimation the highest and lowest values are plotted as a range (appendix 1 pp 56–57). Letters shown next to some regions are related to the WHO regional coding labels. References for this figure can be found in appendix 1 (p 101). DALYs=disability-adjusted life-years. ECCE=extracapsular cataract surgery. QALYs=quality-adjusted life-years. SICS=small-incision cataract surgery.

For cataract, we identified 11 publications providing 58 separate national or regional cost-effectiveness ratio estimates.^37^, ^157^, ^158^, ^159^, ^160^, ^161^, ^162^, ^163^, ^164^, ^165^ The average cost-effectiveness ratios are shown in figure 13. The studies are heterogeneous in perspective, costs included, surgical procedure, and assumed duration of the health benefit, which limited our comparison. The cost-effectiveness ratio ranged from $5 per QALY gained in India to $24 783 per QALY gained in the UK. However, most ratios were less than $1000 per DALY averted or QALY gained, and in 19 (33%) countries the cost-effectiveness ratio was less than $100 per DALY averted or QALY gained. In general, LMICs reported more favourable cost-effectiveness ratios for cataract surgery than did high-income countries. Two studies from India^158^ and Nepal^160^ showed that the cost-effectiveness ratio of a manual small-incision cataract surgery ranged from $5 to $95 per QALY gained and phacoemulsification surgery ranged from $47 to $142 per QALY gained. The highest estimate, from the UK, used a wider perspective, including personal social-care costs.^163^ The higher cost-effectiveness ratios in some studies, mostly from high-income countries, might be partly because of a higher proportion of mild vision impairment in those receiving surgery and possibly higher remuneration to eye care professionals in high-income countries than in LMICs.

**Figure 14:**
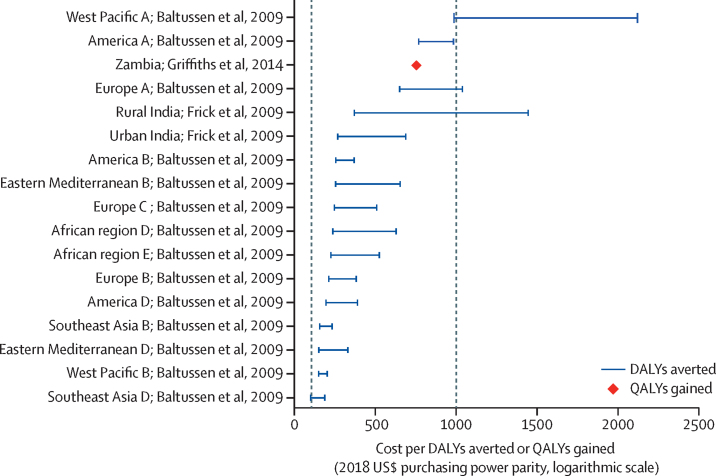
Cost-effectiveness ratios for refractive error services Cost-effectiveness of screening and treating refractive error in schoolchildren^166^, ^167^ and for facility-based refractive error services for all ages.^165^ Studies reported either DALYs averted or QALYs gained. Costs have been inflated to 2018 levels and converted to US$ purchasing power parity. For studies reporting more than one estimation, the highest and lowest values are plotted as a range (appendix 1 p 57). References for this figure can be found in appendix 1 (p 101).

There were two studies on screening and treatment of uncorrected refractive error in schoolchildren, providing 16 separate national or regional cost-effectiveness ratio estimates (WHO subregions), and an additional cost-effectiveness ratio analysis for a facility-based refractive error service in Zambia.^165^, ^166^, ^167^ The average cost-effectiveness ratios, shown in figure 14, ranged from $95–184 per DALY averted in southeast Asia D, to $987–2127 per DALY averted in Western Pacific A. The most cost-effective strategy ($95 per DALY averted) in all 14 regions involved screening children in the age group 11–15 years.^166^ The cost-effectiveness ratio exceeded $1000 in only three regions: Europe A, West Pacific A, and rural India. Reports in India showed that cost-effectiveness ratios for screening and treating schoolchildren were lower in urban areas ($264) than rural areas ($1448).^167^ Both studies concluded that screening and treating schoolchildren for uncorrected refractive error seems to be economically attractive in many settings and world regions.

The wide range of cost-effectiveness ratios suggest regional variations in resource use, costs, and patient characteristics, and heterogeneous study methods such as the cost component, measurement of health benefits, duration of health benefits, and use of different discount rates. This heterogeneity is common in health economics, but limits comparability. The low costs per QALY gained or DALY averted indicate that cataract surgery and refractive services are cost-effective in many settings, encouraging countries to prioritise eye care services. Cataract surgery seems favourable compared with other essential surgical procedures and non-surgical public health interventions in LMICs.^168^, ^169^ However, cost-effectiveness ratios are only one consideration when making resource allocation decisions; others include the locally relevant cost-effectiveness ratio threshold, budget, feasibility, and other specific context factors.^170^

### Strengthening research on eye health economics

Our systematic review and analyses highlighted marked methodological heterogeneity, limitations, and data gaps in eye health economics literature. To address these several actions are needed.

First, standardised methodological approaches and reporting need to be adopted, following international guidelines for health economic evaluations.^171^, ^172^, ^173^ This improvement would increase reliability and enable the comparison of findings between settings and over time. This Commission calls for an international consensus on the process to develop guidance for eye health economic studies and standardise approaches for core components and analyses, particularly for the cost of illness.

Second, more standardised data need to be collected from diverse settings and repeated over time. Our review revealed that data are particularly scarce from LMICs, a gap that needs to be urgently addressed to better inform policy and planning decisions. This step will require financial investment and development of capacity for local data collection and analysis. Opportunities to integrate data collection into routine health system data processes should be investigated. Few long-term studies have revisited estimates over time; these would be invaluable to monitor trends and the impact of interventions.

Third, robust, comprehensive economic impact analyses should be developed to better understand the true impact of vision impairment. Our productivity loss analysis provides only a partial perspective and is limited by the availability of data. A systematic approach in a representative set of countries is needed to collect data to inform more comprehensive models. For example, to improve the estimation of productivity losses, more data are needed in relation to vision impairment and its impact on employment status, wages, absenteeism, and presenteeism.

Fourth, better analyses are needed on cost-effectiveness, budgetary impact, and feasibility in a broader range of settings to better inform national decision making. Cost and cost-effectiveness analyses evaluating alternative service delivery approaches should be done—eg, moving to greater primary-care-based and community-based delivery platforms, integration with other services, and task sharing. Extended cost-effectiveness analyses that would include health system objectives, such as improved financial protection and equity to inform policy and planning decisions, are needed.

## Section 5: Global eye health research

Research is crucial to advancing global eye health; specifically, to understand population eye health needs, identify effective interventions, optimise delivery, and support effective advocacy. This Commission analysed vision and eye health research done in the past 20 years. We investigated the diversity of the research community and explored issues around equitable partnerships and growing capacity. Finally, we did a study (unpublished) to collectively identify the grand challenges in global eye health, highlighting key issues that would benefit from focused research. We outline crucial actions necessary to advance research and to improve eye care delivery within universal health coverage.

### 20 years of eye health research

To investigate how research relates to the distribution and causes of vision impairment, we examined all primary peer-reviewed research on eye health published between 2000 and 2019 using a systematic search of online databases and a semi-automated bibliometric analysis (appendix 1 p 58). After excluding editorials, comments, reviews, and case reports, 156 954 articles were analysed. There was a 50% increase in research output across two decades (62 868 publications for 2000–09 *vs* 94 086 for 2010–19). Only 4% of publications were trials. One notable finding was the three-times increase in the number of publications from China (3602 *vs* 10 594). Almost half (42%) of publications were on one of the five leading conditions, glaucoma being the most frequent (11·0%), followed by cataract (9·3%), and refractive error (8·8%; appendix 1 p 59). Overall, these findings appear in line with the need for research to focus on leading causes of vision impairment.

**Figure 15:**
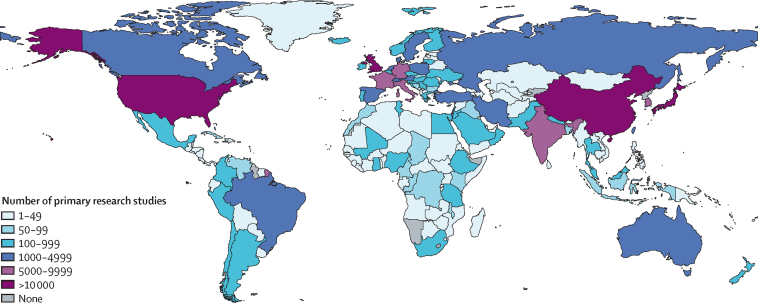
Global distribution of research Number of primary research studies on vision and eye health, by country, done between 2000 and 2019 (studies identified by “explode eye disease” on MEDLINE, July 10, 2020; n=156 954; appendix 1 pp 58–59).

However, there is substantial maldistribution in the geographic focus of eye health research (figure 15). Almost three-quarters of published reports are from high-income countries. Several regions such as southeast Asia, Latin America and the Caribbean, and sub-Saharan Africa, had particularly low research output per person. We anticipate high-income countries will continue to produce a large proportion of basic science, therapeutic, and translational research, although this distribution is changing with the emergence of India and China as major pharmaceutical centres. It is important that all regions have a solid local evidence base on the epidemiology of eye disease and an equally strong understanding of which treatments and service delivery approaches are most effective in their settings. A major gap is the lack of research that explores solutions to eye health problems, particularly in LMICs, where decision makers do not have sufficient and contextually relevant evidence. This gap was highlighted in two systematic reviews.^174^, ^175^ For the first review, of interventions that aimed to increase attendance at diabetic retinopathy screening, 66 randomised controlled trials were identified, none of which were done in LMICs.^174^ The second review of interventions to improve access to cataract services in LMICs identified only two studies.^175^

Given the magnitude of vision impairment in sub-Saharan Africa, we did an in-depth review of randomised controlled trials done in this region since 2000 (appendix 1 pp 60–61). There were four key findings. First, geographic spread of the 86 trials was limited; 16 countries had at least one trial and more than half were done in Nigeria, South Africa, Ghana, or Ethiopia (appendix 1 p 61). Second, trachoma (28 trials) and onchocerciasis (17 trials) were most the commonly assessed. High-quality trials have been crucial for shaping disease control and programme success. Third, trachoma and onchocerciasis research communities are well coordinated in identifying key questions and minimising unnecessary duplication; they engage in extensive collaboration with government-led programmes on neglected tropical disease and their funders recognise that research is needed to develop elimination strategies. Finally, 16 trials were about glaucoma. Given that glaucoma has a high prevalence in many African populations and the uncertainty around how to address this issue, we believe that concerted research action is urgently needed to develop contextually relevant management strategies for glaucoma.

### Equity, diversity, and inclusion in eye health research

Inequality is pervasive in medical science; eye health research is no exception.^176^ Equity and diversity in research teams produces higher quality science that better meets the needs of society. This Commission explored equity, diversity, and inclusion in eye health research in three ways.

First, we assessed female authorship in eye health research since the beginning of 2000 to the end of 2019 (appendix 1 p 58). There were almost 880 000 authorships; gender could be assigned to 780 000. Around 33% were female across the whole timespan. Since 2000, female authorship increased from 28% to 37%. Women held 36% of first authorships and 24% of last authorships.

Second, we assessed diversity of 112 ophthalmology journal editorial boards listed in Scopus (appendix 1 p 62). Gender and country of affiliation were available for 5061 editorships. Women held 23% of editorships and 11% editor-in-chief positions. 72 countries had at least one editor; 1643 (32%) of 5061 editorships were held by researchers in the USA (25% women), compared with only 15 (0·2%) across all of sub-Saharan Africa (20% women). Among 47 countries with at least five editorships, the proportion of editorships held by women within each country ranged from 0% to 60% (median 22%).

Third, we assessed authorship of 89 reports from 86 randomised controlled trials in sub-Saharan Africa to evaluate the extent to which researchers from the continent itself and female researchers were involved. All but one of the 89 reports included authors who originated from the country of the study. Researchers from the country of the study held 51% of all authorships, 74% of first authorships, and 43% of last authorships. However, female researchers from the country of the study held only 8% of all authorships, 12% of first authorships, and 8% of last authorships.

Change is required to improve equity, diversity, and inclusion for women, and other under-represented groups. Strategies for organisational change have been outlined and the need for measurable targets has been reinforced.^177^ *The Lancet* has shown how editors and publishers can address gender gaps and set diversity targets, leading to increased inclusion of women and people from LMICs among its authors and reviewers.^178^ We commend this pledge, and in preparing this Commission we aimed for balance in gender and LMIC representation. Despite our efforts, the proportion of commissioners who are women (44%) or originate from LMIC (38%) falls short of parity. Among our subsidiary papers, more than 50% of authors were female (more than two-thirds held first authorship).

### Increasing capacity for eye health research

Delivery of eye health within universal health coverage requires substantial expansion in capacity to deliver high-quality research in LMICs. We call for investment in institutions in LMICs so that eye health research is primarily executed by researchers living and working in the region. The analysis of randomised controlled trials in sub-Saharan Africa highlights that most research in this region appears to have substantial academic involvement from high-income countries. A structural shift is required so that LMIC institutions increasingly take the lead and are supported by equitable partnerships that address priorities identified within the region. Such partnerships need to maximise meaningful capacity building for local researchers. Previously, some externally driven research projects have failed to sufficiently consider this issue, with local eye health personnel limited to data collection roles as opposed to scientific design and leadership. Subsequently, personnel might be included as authors without being empowered to engage in a meaningful way that develops their research skills. Some lessons can be drawn from the Commonwealth Eye Health Consortium, which built eye health research capacity in LMICs (particularly in sub-Saharan Africa) by providing Masters degrees, doctoral scholarships, and postdoctoral fellowships, with a focus on mentoring and empowering independent researchers. More regionally focused peer-reviewed journals are needed. Some regions might need capacity development support for longer. Increasingly this involves South-South collaboration, with the development of research community networks. This improvement is important considering that there are few, widely dispersed eye health researchers. Funding arrangements are very important for equitable partnerships, with a need for LMIC institutions to take the lead role in obtaining and managing funding.

### Grand challenges in global eye health

Global eye health research needs to focus resources on key questions to maximise benefit. Previous research prioritisation processes were done in the UK, the USA, and for LMICs; these included expert panels with or without open calls for contributions from clinicians and the public, including people who live with vision loss.^179^, ^180^ To collectively identify and focus on key areas that need attention in the upcoming decade, this Commission did a global prioritisation exercise. We drew on the Grand Challenges method, which involves a three-round modified Delphi process (appendix 1 p 63).^181^ We recruited 336 people from 118 countries, working in clinical practice, eye health system management, research and policymaking, and people who live with vision and eye health problems. In round one, participants were asked the question “What are the grand challenges in global eye health?” and could nominate up to five challenges and five corresponding solutions. All 3400 responses were thematically analysed and consolidated into 85 unique challenges. Ranking was done across two rounds to arrive at 16 priority challenges (panel 4).

Panel 4 **Prioritised Grand Challenges in global eye health**

**Improve treatment (condition-specific)**

• Develop models to encourage population demand and ensure access to accurate refraction and affordable, good quality spectacles.
• Identify and implement strategies to improve the quality, productivity, equity, and access of cataract services.
• Improve child eye health by integrating evidenced-based primary eye care services for children into general health services and ensure strong connections to secondary eye care services. Develop and implement sustainable school eye health programmes, including screening and management for refractive error or amblyopia, which are well integrated within education services.
• Develop and implement effective, accessible, and inexpensive pathway approaches for screening, diagnosing, monitoring, and managing glaucoma.
• Develop and implement one-stop services for people with diabetes by integrating diabetic retinopathy screening services with general diabetes care and develop robust systems to ensure ongoing follow-up and referral for assessment and treatment.
• Develop and implement evidenced-based, effective, sustainable, and context-relevant screening and early detection strategies for eye conditions.

**Health system**

• Encourage governments to prioritise integrated people-centred eye care services for universal health coverage.
• Develop and implement evidence-based strategies for the effective integration of eye health services between the primary, secondary, and tertiary level to improve referral pathways, ensuring recognition of those who need secondary care and a timely, reliable, accessible, and affordable care mechanism.
• Develop and implement evidence-based strategies for the effective integration of eye care at the primary care level and with other medical services (eg, child health, diabetes or other NCD services), ensuring that services are widely accessible, affordable, of high quality, and meet the primary eye care needs of the population.
• Strengthen the information system for eye health within health facilities, integrating them into national systems.
• Ensure financing for eye health exists within national budgets and financing structures and increase the investment.

**Access and equity**

• Develop and implement services that prioritise, and by design, reach marginalised or vulnerable groups (women, poor communities, Indigenous people, ethnic minorities, people with disabilities, people in residential care, prisoners, and refugee camps) and people living in rural communities with quality affordable eye services.
• Develop and implement strategies that reduce out-of-pocket costs for those requiring eye care who are unable to afford full-cost services—eg, subsidy, tiered pricing, or insurance.
• Develop and implement responsive programmes to increase the access to and use of eye health services and treatment—eg, reduce barriers to accessing services and increase demand through greater awareness of need and confidence in health-care provision.

**Build resource capacity**

• Increase support from international bodies, professional bodies, colleges, and non-governmental organisations, for geographical regions with severe eye health resource shortages.
• Strengthen leadership and public health expertise across all levels of eye care and ensure that national leadership can influence policy and resource allocation. Additionally, strengthen regional and national professional bodies for eye health practitioners.

Six of the 16 challenges focused on improving specific services and treatment for cataract, refractive error, glaucoma, diabetic retinopathy, and children's eye diseases. The available evidence for each condition warrant different research approaches. For example, cataract and refractive error can be treated by well-established and efficacious interventions, but these do not reach all who could benefit with sufficient quality to be effective. Implementation research is needed to fill this gap; there are few examples, perhaps the most well known is onchocerciasis, for which implementation research was done to determine the best way to distribute ivermectin. Optimal treatment and implementation approaches also need to be determined, in different populations, for glaucoma and diabetic retinopathy to improve acceptance and sustained uptake of lifelong care (panel 5).

Panel 5 **The challenge of glaucoma**

Glaucoma is the second leading cause of blindness (age-standardised prevalence), which results in substantial disability before blindness, yet remains undertreated globally (Bourne et al, 2020). In most prevalence surveys from high-income countries, less than half of all detected glaucoma was previously diagnosed, and in low-income and middle-income countries (LMICs) over 90% of people with glaucoma are not in care (Tham et al, 2014; Vijaya et al, 2008; Kyari et al, 2015). This high percentage is because glaucoma is mostly asymptomatic until relatively late in the disease. As many as 35% of people are blind at diagnosis in LMICs, precluding effective interventions that would have prevented vision loss (Ramakrishnan et al, 2003; Buhrmann et al, 2000; Kyari et al, 2013; Abdull et al, 2015). Glaucoma lacks a one-stop solution such as cataract surgery, because of its chronic nature and complexity of management. In the absence of simple and affordable diagnostic and treatment solutions, the global eye health community has not prioritised glaucoma—eg, when VISION 2020 was being developed (WHO, 2000). There are several crucial issues.

First, there is a need to provide effective treatments that prevent glaucoma progression, and maybe someday, restore function to those with glaucoma damage. Several high-quality clinical trials have shown that lowering intraocular pressure slows, and in some cases stops, glaucoma progression (Garway-Heath et al, 2015; Collaborative Normal-Tension Glaucoma Study Group, 1998; Heijl et al, 2002). To do this intervention safely and effectively remains a challenge in all resource settings. The current treatment is long-term topical ocular hypotensive drops, but poor compliance and ongoing costs are major challenges in LMICs (Newman-Casey et al, 2020). Laser trabeculoplasty, which can be administered in a single session, is one effective strategy that has shown effectiveness in locations with scarce resources (Gazzard et al, 2019). Unfortunately, trabeculoplasty rarely provides lifetime control of intraocular pressure. Future hope is that more effective surgical or laser approaches will provide safe and sustained pressure lowering. Many novel approaches have been developed in the past decade, but none are able to overcome these challenges (Poitras et al, 2019).

Second, individuals need to be monitored to determine whether their glaucoma is progressing so that treatment can be changed or escalated when needed. Such monitoring presents challenges for more remote and resource-limited populations. However, glaucoma monitoring is undergoing a rapid evolution and home-based monitoring using off-the-shelf technology might become available in the near future (Che Hamzah et al, 2020). The growth of vision centres in India and elsewhere, staffed by mid-level ophthalmic personnel and supported remotely by ophthalmologists, is an example of how to provide ongoing monitoring and shared care for people living in remote settings.

Third, affordable and effective screening approaches are needed to enable identification of individuals at risk of sight loss. Major advances in automated grading of optic disc photographs has led to highly accurate glaucoma diagnosis on the basis of a single photo (Li et al, 2018). Widespread use of screening using fundus imaging with artificial intelligence-assisted grading could allow glaucoma to be diagnosed alongside the other major causes of blindness at low cost. Implementation studies are needed to determine how and where to apply these new tools.

Innovation in glaucoma detection and management, which will probably occur soon given the rapidly improving technology, could catalyse a new care model in which earlier detection and effective long-term intraocular pressure lowering combined with remote monitoring can prevent unnecessary blindness globally. To reach this goal, the global eye care community needs to include glaucoma in eye care planning, recognising the centrality of the patient as a partner in management. Many important research questions remain unresolved and require substantial investment and a concerted global effort to answer.

References for this panel can be found in appendix 1 (p 109).

Five of the 16 challenges related to health system factors including: advocating for and establishing the policy framework to implement people-centred eye care for universal health coverage; strengthening integration between primary and secondary levels of care, and between eye care and other health services; and strengthening the health information system and ensuring better budget allocation for eye care. Two challenges reflected the need to address shortages in human resources for eye health in parts of the world—eg, sub-Saharan Africa, and to increase capacity of eye health personnel in public health and leadership. These challenges could benefit from the application of health systems and policy research to answer questions such as to what extent improving integration between primary and secondary eye care services improve coverage, quality, and equity, and reduce out-of-pocket costs.

Equity is a crucial issue for universal health coverage and is of relevance to any service delivery-related research. We identified three challenges concerned with improving access and promoting equity, reaching vulnerable and marginalised groups, and developing and testing strategies to reduce out-of-pocket costs. Disadvantaged groups might have worse outcomes that are obscured by aggregate data. A simple step is to disaggregate data by the minimum set of social variables that are relevant in each context. In addition, equity-relevant reporting guidelines and frameworks are available and can be used in eye health research to consider equity in the design, analysis, and reporting of research.^182^, ^183^ These challenges highlight the need for researchers to include people with vision impairment and communities that the research is targeting in all aspects of the process, from identifying the research question to co-designing and implementing the study, data collection, analysis, and dissemination.

We believe this list of challenges serves as a starting point for immediate action that needs to be taken by researchers and funders. We call for funders to use the grand challenges to guide their research investments. Further, the list provides an opportunity for consortia and networks, advocacy organisations, universities, and governments to organise their activities around these challenges. In 2021, the authors of this Commission will seek to cohost a workshop to generate a research agenda and establish collaboration opportunities. The outputs of this workshop will include a strategy for periodic monitoring of eye health progress.

### Delivering research for universal eye health

We have identified six crucial actions to generate and use evidence to promote eye health within universal health coverage. First, to develop a research agenda based on the grand challenges. Second, to increase solution-focused research including more contextually relevant implementation and health systems research, in partnership with patients, communities, service implementers, and policy makers, with an emphasis on ensuring that services address the leading causes of vision impairment in terms of coverage, quality, equity, and financial protection. Third, to ensure more emphasis is put on translating research findings into policy and practice, including better partnership with policy makers and integrating eye health research into general research priorities. Fourth, to avoid research waste by ensuring relevant questions are answered by adequately powered, robustly designed studies that are informed by systematic reviews of all the available evidence. Fifth, to support capacity building through equitable North-South and South-South partnerships between researchers and research institutions. Sixth, to ensure inclusion by monitoring diversity of research teams with structural change toward better inclusion, and by including communities and patients throughout the research development process.

## Section 6: Beyond 2020—delivering high-quality universal eye care

### The case for action

The UN has set ambitious SDG targets for 2030.^28^ This Commission argues that eye health is integral to advancing sustainable development. Extensive evidence shows that improving eye health contributes directly and indirectly to several SDGs. Vision impairment profoundly impacts education and work, with substantial implications for poverty and the economy. Improving eye health benefits quality of life, general health, and wellbeing.

Universal health coverage is central to delivering SDG3 (good health and wellbeing). We reason that universal health coverage is not universal without affordable, accessible, high quality comprehensive eye care. One of the leading recommendations in the WHO *World report on vision*^2^ is “making eye care an integral part of universal health coverage” through implementation of integrated people-centred eye care. However, this ambition is far from being realised. In many regions, the scale and approach of existing service delivery are insufficient to meet current population needs, let alone projected increases in the magnitude of chronic eye conditions and vision impairment by 2050. In view of likely benefits to sustainable development, increasing need, and availability of cost-effective scalable interventions for many common eye conditions, the case for urgent action on eye health is compelling. Global eye health needs to take its rightful place within global development and the health agenda.

### Conceptualising eye health within universal health coverage

WHO defines universal health coverage as follows: *“Universal health coverage means that all people and communities can use the promotive, preventive, curative, rehabilitative and palliative health services they need, of sufficient quality to be effective, while also ensuring that the use of these services does not expose the user to financial hardship”.*^184^

**Figure 16:**
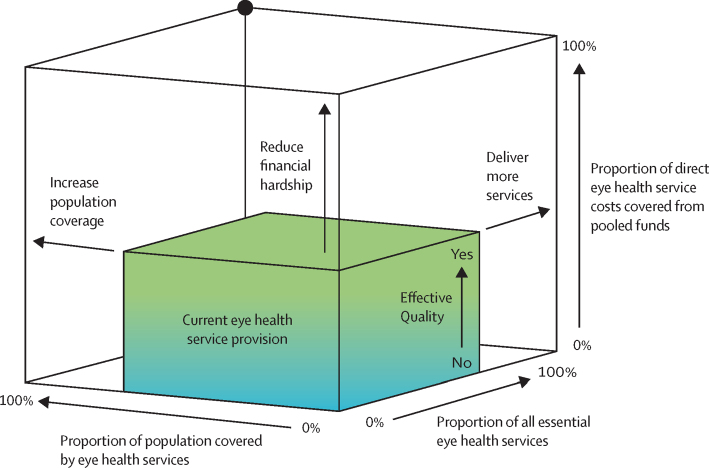
Considerations for universal eye health coverage Adapted from WHO 2015,^185^ to contextualise to eye health services and include the quality component. The inner cube has a colour gradation representing a range in the quality of delivered services; green represents effective quality services and blue represents ineffective services.

The core components of universal health coverage are often illustrated using a cube (appendix 1 p 64) and are intrinsically linked to health financing; the inner cube reflects budget constraints.^185^ To conceptualise eye health within universal health coverage, we have adapted the cube (figure 16). Universal health coverage is not a fixed final goal; population health needs change constantly and new treatment options are regularly developed, which presents challenges for defining interventions and measuring impact.

Here, we explore what the delivery of high-quality eye care involves within universal health coverage, and the actions needed to move towards this goal by 2030. We propose a framework for how eye health services could be integrated within the broader health system and in non-health sectors. We highlight promising integration strategies across levels of care and examine key enabling factors: workforce, financing, and technology. We illustrate important issues by focusing on cataract surgery and refractive error services, drawing together the available data to understand how these services are currently doing in relation to universal health coverage (coverage, quality, equity, and financial protection). Finally, we discuss cross-cutting issues integral to delivering eye care within universal health coverage (monitoring progress, quality, access, equity, and political prioritisation).

### Selecting eye services for universal health coverage

National policy makers face complex, context-specific decisions in prioritising services that maximise public health benefit with finite resources.^186^ Cost-effectiveness is often an influential consideration, alongside need, sustainability, affordability, and feasibility. Additionally, countries might also consider equity—giving higher priority to services that benefit the poorest communities or those offering greater protection from financial risk.^186^ Ideally, coverage of high-priority services is extended to all before adding medium-priority or low-priority services.

Our eye health economic literature review (section 4) highlighted the scarcity of cost-effectiveness data, particularly for LMICs, covering few interventions. Substantially more economic data are needed to inform countries that are deciding which eye health services to offer within universal health coverage. Considering current data limitations, how should eye care prioritisation choices be made? The epidemiology of eye disease is key, with an emphasis on the leading causes of vision impairment. However, many people presenting for eye health services have non-vision-impairing conditions and an eye health need that requires these services.

WHO is currently developing a package of eye care interventions for multiple eye conditions, informed by epidemiology and field experts.^187^ The inclusion of interventions is guided by high quality clinical practice guidelines and systematic reviews. Countries will be able to use these tools to inform service inclusion decisions, method of delivery, and resource implications. However, the need for more cost-effectiveness data to inform decisions relative to other health priorities remains.

Although countries vary widely in terms of population needs and health system capacity, core components necessary to meet general population eye health needs are similar in different settings. Ideally, a minimum package of eye care within universal health coverage would include primary eye care (promotion, prevention, and refractive services), eye care integrated within other services (neonatal care, school eye health, non-communicable eye disease services, care of older people), specialist ophthalmic services (to restore—eg, cataract surgery and preserve vision—eg, glaucoma, diabetic retinopathy and age-related macular degeneration management), and vision rehabilitation services.

### Delivering integrated people-centred eye care

Delivering eye health services within universal health coverage requires multidimensional integration, throughout and beyond the health system. Eye health needs to be included within national health plans, policies, and financing mechanisms. It also needs to be considered in policies and planning by other government ministries such as education, labour, and finance.

**Figure 17:**
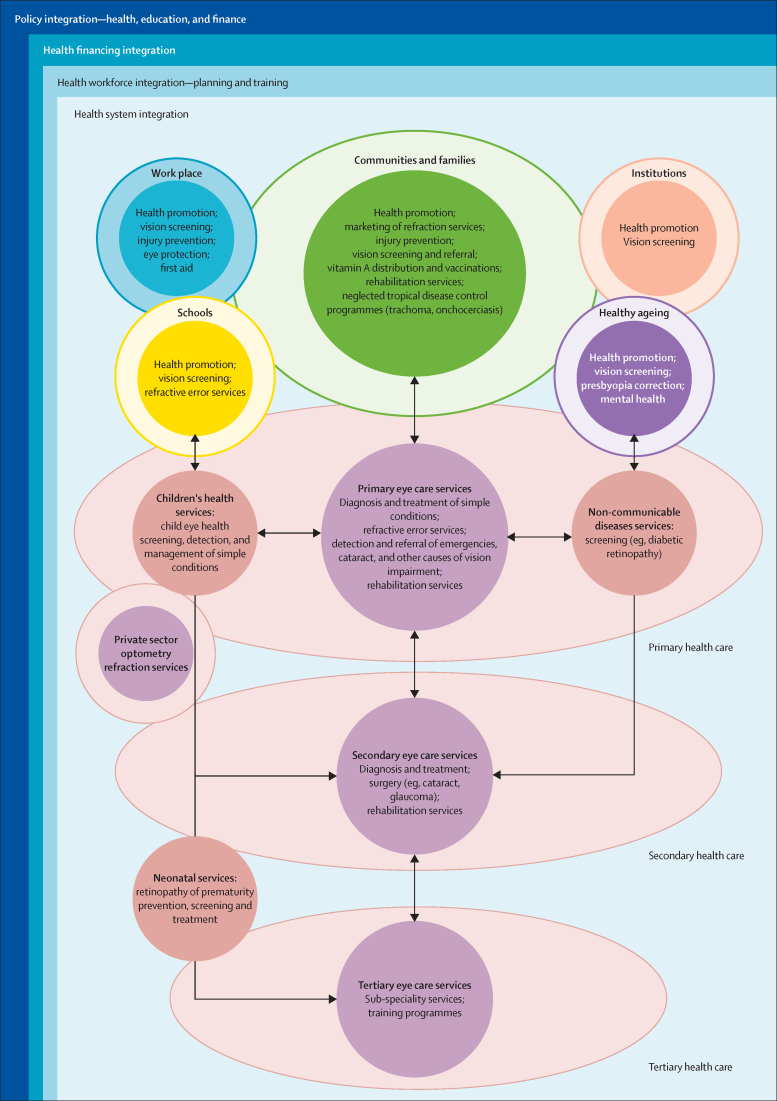
Integration of eye health services within the health-care system

Although we recognise that health systems vary substantially between and within regions in terms of capacity and the stage of development, there are some broad organisational similarities. Here, we present how eye care can be integrated into different aspects of society and health systems including community, primary, secondary, and tertiary levels (figure 17). Appendix 1 (pp 65–67) summarises services that could be provided in different settings in low-resource, middle-resource, and high-resource settings.

The *World report on vision*^2^ by WHO has placed renewed and welcome emphasis on a people-centred approach to eye care delivery. Individuals and communities need to be empowered through increased health literacy to actively engage in shaping and using services to drive demand. Effective, timely services need to be easily accessible to the population in convenient locations, with easy navigation through the system in cases for which more specialised services are required. Effective health systems are characterised by strong connections within and between levels.

A crucial component of delivering eye health within universal health coverage is effective primary eye care, needed in all resource settings, which connects with the wider primary health system and secondary eye health services. This Commission takes a broad view of primary eye care, encompassing activities and interventions within community settings and primary health facilities (general care and eye care). Primary eye care can include promotive, preventive, diagnostic, treatment, and rehabilitative services.

#### Eye care in community settings

Delivering services within or close to where people live and work results in increased access and use.^188^, ^189^ There are many effective eye health interventions that can be delivered at community level.

Eye health education and promotion within communities can lead to improved knowledge and service uptake.^190^, ^191^, ^192^ Promoting community-based facial cleanliness is integral to trachoma elimination programmes.^21^ Health promotion messages about meticulous contact lens hygiene are important in reducing corneal infections.^193^ To deliver education and promotion within integrated eye care, providers need to use more people-centred or community-centred design approaches, recognising that people are co-producers of health, not just users, beneficiaries, or choosers.^194^ More behaviour change and co-design research is needed. Previously, many interventions intended to improve facial cleanliness for trachoma ignored accepted theories and findings about behaviour change.^195^ A co-design process is underway in Ethiopia that seeks an in-depth understanding of social and cultural determinants of behaviour, working with community groups to propose and test contextually relevant strategies to promote facial cleanliness.^196^ Digital communication offers new routes to share and amplify such messages.^192^

Several community-level interventions have shown effectiveness. Vitamin A supplementation and measles vaccination markedly reduced childhood cornea scarring.^197^ Rubella vaccination has rendered ophthalmic complications of congenital rubella rare.^198^ Mass drug administration for trachoma and onchocerciasis are delivered in community settings and frequently involve community members in distribution (community-directed).^21^ Community volunteers distributing azithromycin were integral to trachoma elimination in Ghana.^199^

Some countries train community health workers in primary eye care, including health promotion, vision testing, and referrals. Clinical trials are few; however, they indicate promise in identification of people with eye health needs. In Nepal, community health workers were trained to recognise corneal abrasions and infections using a torch, and to provide early treatment and referral, which led to improved outcomes.^200^ In Kenya, community health workers have been trained to use a smartphone-guided vision test and algorithm to identify and refer people with vision impairment and other eye problems.^201^ This approach has been tested in a cluster randomised controlled trial in Kenya,^202^ and preliminary findings (unpublished) have shown that an increased number of people with vision impairment and eye problems are attending eye clinics.

However, sustaining programmes that engage general community health workers is hard.^203^ Although studies show efficacy of community health worker programmes under controlled research conditions, long term programmatic implementation is less certain.^204^ Implementation research is needed to establish approaches to sustainably embed basic eye health in training and activities of community health workers.

Traditional eye remedies are widely used in south Asia and sub-Saharan Africa to treat many eye conditions.^205^, ^206^, ^207^, ^208^ These remedies, frequently plant-based, are either home-made or sourced from traditional healers.^205^ They can worsen clinical outcomes through direct toxic effects, secondary infection, or delay in seeking appropriate treatment.^206^ Several studies have found that careful engagement and training programmes for traditional healers can improve practice, reduce the use of traditional eye remedies, and promote more rapid referral.^208^, ^209^ Such programmes are not widespread, but have potential for impact.

Pharmacies and informal drug sellers (eg, patent medicine vendors in Nigeria), can be the first source of health care accessed by people with eye conditions.^207^ They can be an important source of health advice and simple treatments. However, in some countries drug stores are poorly regulated, which leads to inappropriate advice or treatments such as sale of steroid eye drops to people with corneal infections.^210^ There is an opportunity in many settings to engage with pharmacists and drug stores to promote eye health messages, safe prescribing, and appropriate referral.

#### Eye care in schools

Many countries have developed school eye health programmes (appendix 2 p 5).^44^, ^45^, ^211^ Screening approaches to identify children with vision impairment—eg, training teachers to test vision, appear effective.^78^, ^212^, ^213^ Most vision impairment in school-aged children is due to uncorrected refractive error.^214^ Providing services in schools (refraction and dispensing spectacles) increases access and uptake; low cost, high quality, ready-made spectacles can meet the needs of more than 80% of children with uncorrected refractive error.^215^ However, subsequent spectacle wear can be low; contextual interventions are required to increase spectacle use.^216^ An important consideration is how to reach children who are not in school.^217^ Comprehensive school eye health programmes also include health education and promotion, and support inclusive education for children with irreversible vision impairment.

Well-coordinated action is crucial in Asia to address myopia, with strong collaboration between departments of health and education. This issue requires major changes in education delivery and preventive approaches to slow down the onset of myopia.^218^ For example, China is currently developing a national myopia strategy, with strong links between health and education (appendix 2 p 6).

#### Preventing eye injuries

Workplace injuries are an important cause of serious ocular morbidity.^219^ Minor abrasive corneal trauma during agricultural work is a frequent risk factor for corneal infection in south Asia.^200^ Using safety goggles while performing some tasks can prevent such injuries.^220^ The introduction of seat belts and firework regulations reduced ocular trauma; these should be promoted in areas where eye-related injuries remain common.^221^, ^222^

#### Eye care delivery in general primary health care

WHO calls for strengthening eye care delivery within primary health care to complement work in secondary and tertiary eye care.^2^ In many countries, general primary health-care workers assess, treat, and refer people with eye problems. Primary health-care staff might also support community-based activities. Services vary substantially between contexts; here we outline some similarities and integration opportunities.

Longstanding work has enabled primary health-care workers to deliver primary eye care and several training manuals have been developed.^223^ However, experience is mixed; establishing primary eye care within primary health care is not simple and requires substantial training investment and enabling environments. General health-care workers usually receive little primary eye care training, leading to important knowledge and skill gaps.^210^, ^224^, ^225^, ^226^, ^227^, ^228^ For example, training primary health-care nurses to test visual acuity did not increase referral rates in Malawi.^229^ Short supply of basic equipment and insufficient ongoing support to deliver primary eye care are frequent issues.^203^, ^210^, ^224^, ^230^, ^231^ Programme sustainability is challenging in areas that depend on external non-governmental funding.^232^

There are some promising strategies such as the Lady Health Worker programme^233^ in Pakistan, which includes primary eye care, increasing detection, and referral from the community; however, connecting to secondary care still remains challenging (appendix 2 p 7). In China, village doctors are trained by secondary eye care teams to identify and refer people with vision impairment.^234^ There is strong policy backing, whereby all people younger than 6 years and older than 65 years are offered a vision check in primary health-care facilities. This policy increases the number of people attending secondary units, which are more invested in supporting primary eye care services. An intensive programme in Rwanda, training health-care nurses in primary eye care, is now embedded in the general nursing curriculum which substantially improves access to eye care services throughout the country.^235^

Integration of primary eye care into primary health care services for younger children is a major opportunity. WHO identified ten key activities to promote healthy eyes in children.^236^ These activities were tested in Africa.^237^ A modification was included in the curriculum of WHO's Integrated Management of Newborn and Childhood Illness (IMNCI)^238^ in Tanzania (appendix 2 p 8). IMNCI is used in more than 100 countries to guide facility-based management of illnesses in children younger than 5 years, in the primary care setting, and is possibly a scalable and sustainable way to deliver interventions that address primary eye care needs of young children.

Screening for diabetic retinopathy depends on close partnerships with general medical services. The delivery of screening is being transformed by the increasing availability of low cost retinal cameras operated by non-specialists, acquiring images for remote grading. In high-income countries, established diabetic retinopathy screening and treatment programmes deploy technicians to community settings to collect images for remote grading and decide on the referral. In the UK, the proportion of blindness in the working-age population, caused by diabetic retinopathy, has declined because of improved diabetes control and the national screening programme (appendix 2 p 9).^239^ Programmes are also being developed in LMIC settings. For example, in India, multiple public pilot programmes have been developed by the Ministry of Health, showing a substantial increase in the number of people screened at clinics for non-communicable diseases and community health centres.^240^ In sub-Saharan Africa and the Caribbean, multiple countries have developed regional or national diabetic retinopathy screening programmes that also embed diabetic retinopathy services within general non-communicable disease services.^241^

The delivery of primary eye care by general health workers is an area that requires systematic high-quality implementation research that analyses policies and systems to assess strategies, which would fully embed primary eye care within primary health care. Realistic expectations are needed in terms of the tasks that general primary health-care workers can do alongside other duties. In this context, technological developments hold promise in enabling task sharing.

#### Specialist eye health services within primary care settings

Some LMICs have permanently based mid-level ophthalmic personnel (non-physician specialised practitioners) in larger primary health-care facilities such as community ophthalmic nurses stationed in large district health centres in The Gambia.^242^ These nurses serve approximately 30 000 people and provide a bridge between primary health care and secondary services, supervising community-based primary eye care activities. Similar examples are seen in Ethiopia (integrated eye care workers) or in Tanzania (assistant medical officers in ophthalmology).

Outreach services are delivered in more remote regions by eye care teams visiting periodically, and providing outpatient clinics and surgery.^204^ In some countries these services are widely used to increase access to surgery (eg, cataract).^243^, ^244^ Outreach services provide an opportunity for refresher training and supervision of primary health-care staff involved in primary eye care. However, communities can be left without access to services for extended periods, and this method might discourage the development of local eye care services.

Primary eye care facilities or vision centres, pioneered by several large non-governmental eye health organisations in south Asia, are satellite units operated by mid-level ophthalmic technicians (appendix 2 p 10).^245^ These units are separate from the public health system and are well integrated into the networks of secondary and tertiary hospitals, with teleophthalmology support and integrated electronic record systems.^246^ Approximately 80–90% of people presenting to vision centres can be managed at that level, and the remainder are referred on. However, the degree of integration with general public health structures is suboptimal.

In high-resource and middle-resource settings, primary eye care is mainly delivered by specialised personnel. In many high-income countries within community settings, primary eye care and refraction services are usually provided by eye health specialists, optometrists, or ophthalmologists in the private sector, with access to sophisticated diagnostic equipment, particularly in urban areas.

Overall, populations are well served by easy access to a dedicated eye health workforce, closely connected to the general primary health-care system. This Commission recommends that, where possible, countries should move towards developing a dedicated eye health workforce to strengthen primary eye care in the public and private sectors. Cadres vary between countries. How these personnel are managed and integrated is an important area for implementation research.

#### Refractive error services and public–private collaboration

There is an increasing need for refractive error services for adults and children worldwide. Access to refractive error and optical services in primary care settings is crucial. Services vary between countries and include optometrists, refraction technicians, and ophthalmologists. A competent workforce is important, in addition to good governance and equitable delivery, to ensure appropriate and quality spectacles for all.

In many regions, refractive error and optical services are provided by the private sector and therefore, are largely market-driven to an extent that other eye care services might not be. Market forces have been a major incentive for service development at scale in some settings, providing well for population needs. However, if the distribution of refractive error services is influenced by what providers consider a viable return on investment, populations in areas of high deprivation could remain without access to services.^247^ The cost of spectacles can vary greatly between settings, and can involve large out-of-pocket expenditure and be unaffordable to many people. In some countries there is a large unregulated market of optical shops that might provide poor quality services.^248^ The private sector usually does not share data on the number of refractions done and spectacles dispensed—an information gap that needs to be addressed for countries to be able to monitor refractive error services.

However, the private sector represents a huge opportunity to bring refractive error services (and primary eye care more broadly) closer to communities. Indeed, given the magnitude of uncorrected refractive error globally, eye health cannot be addressed as part of universal health coverage without a major contribution from the private sector. To contribute to delivering eye health within universal health coverage, more consideration needs to be given to developing the right regulatory and market conditions to promote high quality, affordable, and equitable services.

A strong regulatory framework for prescribing and dispensing refractive error corrections allows for coherent health promotion messages. Users' trust in the clinical competency and decision making of eye health workers is important for generating and sustaining service demand. By contrast, the sale of unregulated, variable quality optical devices (including via the expanding online market) might increase access, but at the possible expense of quality and sustainability.

Public–private partnerships are promising ways of increasing spectacle coverage while ensuring a safety net for those who are unable to afford private sector services. Examples include the national Jaminan Kesehatan Nasional (JKN) scheme in Indonesia, in which the vision test is provided by the public sector and a voucher is given to obtain spectacles from accredited private entities. The national school eye health programme in Trinidad and Tobago provides a government voucher for spectacles to reimburse the private sector provider. In Rwanda, the national insurance programme includes a sight test and a standard pair of spectacles, and more expensive spectacles will also be subsidised. In the UK, the Scottish Government made primary eye care free for all, at the point of use, and increased the fees paid to providers for their services. This change increased the viability of service providers in deprived areas, despite a lower demand for spectacles that generate more profit. Given the large and growing problem of uncorrected refractive error, other strategies and better evidence, with a focus on equity, are needed to improve effective coverage.

#### Connecting people to secondary eye care

A study^249^ from Uganda illustrates several challenges in integrating eye health services into primary health care and connecting people to secondary services (appendix 2 p 11). This study documented the journey of people with severe corneal infections seeking health care. Severe corneal infection is an acute painful condition requiring urgent specialist treatment within a few days of onset to prevent permanent sight loss. Although many people presented to a primary health-care facility within 2 days of symptom onset, the seriousness of their condition was frequently not recognised, appropriate treatment was not initiated, urgent referral to the hospital eye clinic was not given or followed, and the opportunity to prevent irreversible vision loss was missed. Many people visited multiple health facilities before reaching the eye unit for assessment and definitive management. Other studies^250^, ^251^ have also reported that attending additional intermediate health facilities for emergency and non-emergency conditions increases delays in accessing treatment and the direct and indirect costs for patients. Effective referral decisions and clearly defined pathways, ongoing supervision, and refresher training, would strengthen primary eye care. Referrals made using an electronic system have shown notable success in increasing the reliability and timeliness of attendance at secondary care facilities.^212^

#### Eye care within secondary and tertiary health care

Secondary eye health services are important for diagnosing and managing the leading causes of vision impairment, beyond uncorrected refractive error. These services include surgery (for cataract and glaucoma), and laser and injection therapies (for diabetic retinopathy and age-related macular degeneration). Access to rehabilitation services remains inadequate in many regions and requires concerted action. In many countries, secondary units support the development and supervision of primary eye care. Tertiary facilities usually have a larger number of specialists, offering a range of subspecialty services with more sophisticated equipment than in secondary units to manage complex problems. Typically, ophthalmology and ophthalmic nurse training programmes are based in tertiary facilities. In many LMICs, secondary and tertiary eye health services have had long-term underinvestment in eye health staff, equipment, and infrastructure. These issues are high priority and need to be addressed to deliver efficient and effective services.

At secondary and tertiary levels, good communication and integration within the health service is needed—eg, prevention, screening, and treatment of retinopathy of prematurity requires close collaboration with neonatal unit teams. With the increasing availability of low cost retinal cameras, task sharing is becoming a reality in some locations, including a remote assessment by an ophthalmologist (appendix 2 p 12).^252^ Similarly, diabetic retinopathy needs to be well integrated into primary health care, from screening to clinical assessment, and treatment in secondary and tertiary units.

#### Vision rehabilitation services

Vision rehabilitation is a set of services that assists individuals who experience disability to achieve and maintain optimal functioning. Services assist people with activities of daily living, accident prevention, and general physical and psychological wellbeing. People seeking vision rehabilitation face a range of challenges including, but not limited to, scarce services, physical barriers, inadequate skills and knowledge of the health workforce, and prohibitive costs. These barriers contribute to the global estimate of only 15% of people who could likely benefit from these services currently accessing them.^253^ Insufficient systematic data remains a persistent issue in raising the profile of vision rehabilitation and understanding the extent of the problem.

A nuanced tension exists between identity and recognising disability being part of natural human diversity, the interaction of impairment with the physical and social environment causing disability and a perception of medical care narrowly construed as being to treat and cure disease. This tension becomes acute in determining who ought to have responsibility for driving progress in vision related rehabilitative services.

Concerned by the general challenges in rehabilitation, WHO convened a meeting in 2017, *Rehabilitation 2030: A call for action*.^254^ Participants agreed to improve the integration of rehabilitation within health systems and strengthen intersectoral links. This shift recognised the gap produced by different government departments who have responsibility for rehabilitation and placed an emphasis on health to drive change as part of a continuum of care. The WHO *World report on vision* calls for vision rehabilitation services to be included within eye care interventions. Intersectoral partnerships with education, social services, and labour, are essential to provide person-centred support with complimentary and additional support for social, economic, and cultural participation.

Although improvements in the external environment are important for inclusion and development, vision-related rehabilitative services are intrinsic to person-centred health care for people living with blindness or vision impairment. Because the availability and access to services is poor, this Commission urges a greater endeavour on the part of eye health policy makers, practitioners, administrators, providers, and donors to advance vision rehabilitation as an essential part of integrated people-centred eye care. This shift will require action by WHO, governments, and eye health leadership bodies such as the International Agency for the Prevention of Blindness to raise awareness and build policies in collaboration with organisations for people with disabilities, rehabilitation professionals, and other sectors.

### Human resources for eye health

#### Aligning the health workforce and eye health needs

Delivering eye care within universal health coverage relies on an appropriately trained, connected, and enabled workforce who are available, acceptable, and accessible.^2^ Workforce competencies and configuration need to be matched to population eye health needs. Services vary substantially in the complexity, type of equipment, facilities, and competencies required. Workforce plans for eye health need to be included in the overall national health workforce planning. Tools, such as the WHO Workload Indicators of Staffing Need, can be used to systematically analyse workforce requirements and personnel deployment to meet population needs.

An integrated eye care team within the public and private sector is needed and would encompass community-based volunteers, delivering health messages and preventive interventions, through to subspecialists in secondary and tertiary ophthalmology centres, providing complex medical and surgical treatment. Workforce structures and responsibilities differ between health systems; contributory roles include ophthalmologists, optometrists, allied ophthalmic personnel, general health workers, counsellors, equipment technicians, vision rehabilitation workers, managers, administrators (IT, finance, procurement, maintenance), and community volunteers (appendix 1 p 68). Team composition varies with population characteristics, disease patterns, and health system maturity. The workforce needs to be well connected horizontally, within the local health system, ensuring appropriate access points and localised care and vertically for effective referral of more complex problems (figure 17).

WHO is calling for a change in eye health care delivery towards a more person-centred approach, with increased emphasis on service delivery initiated in primary care.^2^ This WHO vision needs a broader workforce view, involving populations and civil society alongside the formal health sector, in contextually specific community-based activities. To expand access to primary eye care in areas with few community-based allied ophthalmic personnel, sharing tasks with non-eye-care health workers is necessary. This expansion requires rigorous, competency-based training, reliable provision of essential equipment and consumables, supportive supervision from specialist services, quality assurance, and ongoing learning opportunities. The widespread introduction of core competency-based skills training for eye health into general medical and nursing training is a good starting point.

Primary eye care is preferably delivered by appropriately trained allied ophthalmic personnel, working closely with primary health care teams, located in easily accessible settings. Populations benefit from their expanded competencies and focus on eye health activities. For example, community-based vision centres in India. In high-resource and middle-resource settings, optometrists and ophthalmologists frequently provide the first point of contact for those with symptomatic eye conditions and can potentially provide opportunistic screening to detect asymptomatic conditions. Many LMICs need to develop training capacity for allied ophthalmic personnel and optometrists, and strengthen regulatory frameworks and ensure good governance.

For strong secondary and tertiary eye care services, planned investment in training and providing equipment for ophthalmologists and the associated team, is needed. The secondary and tertiary teams also support primary eye care, providing training and supportive supervision. Shaping the eye health workforce requires responsive planning and training for context specific needs.

#### Enabling environments to increase efficiency

Optimising productivity or efficiency of services requires personnel with the relevant competencies and resources to be present at each level. Workforce productivity can be a major challenge, with underuse of specialist eye health capacity, evidenced by wide variation in the number of cataract operations done each year by ophthalmologists.^255^, ^256^ Simply increasing the workforce size is insufficient. An enabling environment is crucial for workforce productivity, as the shortage of eye health workers is compounded by limited access to equipment and consumables.^230^, ^257^ Maximising the effectiveness of each team member is key to increasing overall productivity, and needs to be addressed within a local and health system context.

There are good examples from different resource settings showing that service efficiency can be increased using careful process analysis and well-coordinated teamwork. With outreach teams supporting primary eye care and ensuring a steady flow of patients for cataract surgery and hospital support teams ensuring sufficient surgical supplies, sterile instruments, and regular equipment maintenance, the surgeon can be more productive and average around 1500 cataract surgeries a year, which is routinely achieved in settings such as Nepal and India.^258^ These principles can be applied in other LMICs.^259^

Efficiency also varies considerably between the public and private settings, with variation in output attributed to training, personnel oversight, enabling workplace, incentives, and good management.^230^, ^260^ More research is needed on enabling and motivation factors that influence service volume and quality.^261^ Motivation and retention are driven by many factors such as the work, responsibility, achievement, organisational purpose, recognition and growth, compensation, security, status, work conditions, and the relationship with the supervisor. Understanding these factors in local contexts will help in building empowered, self-led teams. Retention, motivation, and performance depend to a large extent on how well this improvement is done.

#### Strengthening training to build quality

In many regions, education is based on outdated curricula taught in a traditional, professional, siloed approach focused on knowledge of diseases rather than competencies. The patient-centred approach outlined in the WHO *World report on vision*^2^ has not always been adopted by the health education system.^124^ There has been considerable development; updated and educationally sound competency-based training curricula for ophthalmology are now available, with several international collaborations leading to extensive exchange of knowledge.^262^ There needs to be a further shift, with increasing emphasis on competency focused learning tuned to meet population eye health needs.

Surgical training of ophthalmologists is an area of focus worldwide, with concern about insufficient training opportunities.^263^ To address these shortcomings, several initiatives are ongoing to strengthen surgical competency training programmes. The quality of these programmes and subsequent service provision depend on the training faculty, equipment, and opportunities to practise under supervision. There is increased emphasis on training the trainers to empower their approach. Programmes that are being cascaded in east Africa have transformed training practices.^264^ To improve the safety and efficiency of surgical skill acquisition, training has moved away from the apprentice model towards the use of simulation surgery. Simulation-based surgical education uses eye models and has been shown to rapidly and effectively increase surgical competence.^265^ This method increases confidence of the trainee surgeon and improves patient safety by reducing complications and surgical errors. Relying on the outdated apprentice model of surgical training is no longer appropriate. When underpinned by the principles of adult learning and encompassing key facets of educational theory, surgical simulation training is an important approach to bring novice eye surgeons to a competent level before operating on patients under supervision.

Lifelong learning is essential for the workforce to maintain skills in the context of rapid growth in medical knowledge and ever-changing health systems. In high-resource settings, formal and informal opportunities build professionalism and competencies in areas such as research skills, leadership, policy, and management. Equitable transferability of these learning opportunities to a global scale is becoming increasingly recognised through platforms such as the Orbis Cybersight programme and the International Centre for Eye Health open education courses. Shared learning has grown with the increase of a global online audience, particularly during the first months of the COVID-19 pandemic. These resources enable self-directed learning and lifelong educational opportunities for eye health professionals, particularly beyond ophthalmologists.

#### Workforce size and distribution

Universal access to eye health can only be achieved if an appropriately skilled and equipped eye health workforce is available and accessible to all those in need. National and subnational analyses of workforce needs in relation to population eye health are crucial, but currently not readily available. The International Council of Ophthalmology (ICO) periodically assesses the global ophthalmology workforce. For 2015, they estimated 233 000 ophthalmologists in 194 countries (appendix 1 p 69).^266^ Similarly, the World Council of Optometry assessed the number and distribution of human resources for refractive services (2017–20), and reported 478 000 personnel in 126 countries. Recognition of optometry has developed in many countries and is either fully regulated, partly regulated, or legal recognition is underway. In some countries, the scope of optometry has expanded from primarily refractive services to some diagnostic and treatment services.^267^ These data are challenging to collect, but data on allied ophthalmic personnel have proven particularly difficult to collect because of the wide variability in nomenclature.^268^

**Figure 18:**
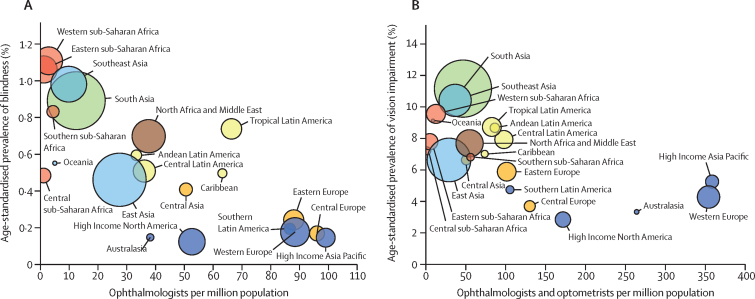
Vision impairment and eye health workforce Shown for the Global Burden of Disease regions. (A) Age-standardised prevalence of blindness (all ages) by the number of ophthalmologists per million population. The circle area is proportional to the number of people who are blind. (B) Age-standardised prevalence of vision impairment (mild, moderate, severe, and blind; all ages) by the number of ophthalmologists and optometrists per million population. The circle area is proportional to the number of people who have vision impairment. Data from Resnikoff et al,^266^ VLEG–GBD,^1^ and World Council of Optometry.^257^

The overall regional prevalence of blindness appears inversely correlated with the density of ophthalmologists per million population (figure 18). We recognise that these data are confounded by socioeconomic factors and not all causes of blindness are amenable to interventions by ophthalmologists. Several regions have clear shortages of ophthalmologists who are able to do surgery for cataract and other conditions, particularly in sub-Saharan Africa, which ranges between 1·1 and 4·4 ophthalmologists per million population compared with a mean of 76·2 ophthalmologists per million in high-income countries (appendix 1 p 70). Similarly, the overall prevalence of all vision impairment appears inversely correlated to the combined density of ophthalmologists and optometrists (figure 18). Several regions have shortages of optometrists; interregional variation is stark, with a median of 1 per million population in low-income countries compared with 221 per million population in high-income countries.

National data on human resource density alone do not indicate access to eye care. There are several challenges that need to be addressed to reach universal access to eye health. First is the maldistribution of the workforce within countries. A study^268^ mapping eye health workforce, in 21 sub-Saharan African countries, reported the mean density of practitioners inside versus outside capital cities (ophthalmic surgeons 12·9 per million *vs* 1·7 per million, ophthalmic nurses 20·5 per million *vs* 7·7 per million, refractionists [including optometrists] 16·7 per million *vs* 2·5 per million, respectively). Similarly, in Latin America there is strong evidence of inequality in the distribution of ophthalmologists, with higher density in more socially advantaged urban geographic areas.^261^ Small island states, such as those in Oceania and the Caribbean, face extreme challenges in developing and retaining an eye health workforce (appendix 2 p 13). Second, by only monitoring numbers, we have no information on workforce competency or scope of service. The ICO survey^266^ explored whether ophthalmologists were surgically active, finding 0·9 surgically active ophthalmologists per million in low-income countries and 32 per million in high-income countries.

Unmet needs, particularly in sub-Saharan Africa and parts of Asia, and future projections indicate that the eye care workforce needs to be urgently expanded, especially because training an ophthalmologist can take more than a decade. In the ICO survey, most countries (60·3%) reported growing workforces; however, the annual global growth rate (2·6%) lags behind the annual growth in the global population aged 65 years and older (2·9%), which is set to double by 2050. How the workforce is trained, deployed, and empowered to deliver services is equally as important as the numbers. Maximising health workforce capacity by developing enabling environments, practices, and increasing motivation is crucial for ophthalmic public health.

### Innovating delivery: technology to support eye health

Advancing eye health services within universal health coverage, particularly in LMICs, will require substantial expansion of service capacity. Many conditions already have effective interventions. However, systems are failing to deliver and there needs to be innovation in delivery. In primary health care settings, appropriate tools are needed to enable non-specialist health workers to identify and refer people that need services. Technological advances offer promise in enabling the larger health workforce to support eye care delivery through task sharing.

#### Teleophthalmology

Teleophthalmology has been used for more than 20 years—eg, to enable consultations for remote communities in Australia.^269^ Eye health technicians in vision centres in India have been linked to ophthalmology hub hospitals.^246^ Teleophthalmology can be broadly classified as asynchronous—ie, when images are collected by technicians for later review—or synchronous in live consultations. Asynchronous teleophthalmology screening for retinopathy (diabetic and of prematurity) substantially increases service coverage in a population, and reduces travel and opportunity costs with a high degree of acceptance and satisfaction.^270^ Synchronous teleophthalmology aids highly trained personnel in diagnosis and decision making and immediate initiation of appropriate treatment, while reducing the burden of patients travelling.

#### Mobile health

Rapid evolution of mobile telecommunications is profoundly impacting life, even in remote locations. Many mobile health (mHealth) applications for eye care have been developed, although few have achieved widespread uptake.^271^ Further, insufficient regulation is a concern, with most publicly available eye care applications being untested or unvalidated.^272^, ^273^ Several visual function testing applications have been validated and show good performance compared with the conventional charts used in rural settings in Kenya and Ethiopia.^274^, ^275^

By linking smartphone applications to a wider system, the effectiveness, efficiency, and knowledge gains can be considerable. For example, a cluster randomised controlled trial of an mHealth system for school vision screening in Kenya found that teachers can reliably identify pupils with vision impairment using a smartphone-based visual acuity application, which then initiates an electronic referral for full assessment.^212^ This method more than doubled the uptake of secondary eye care services among those referred, with active tracing of referred individuals, and SMS messaging to nudge attendance. This system is being scaled up by the Kenyan Ministry of Health in several regions. An integrated mHealth system is also being scaled up in Pakistan, enabling community health workers to identify and refer people with eye health needs and increase vision testing and decision support in the community (appendix 2 p 14).

#### Artificial intelligence

There is considerable interest in the potential of artificial intelligence and particularly deep learning in ophthalmology.^276^ This interest is driven in high-income countries by challenges in managing high volume chronic conditions (age-related macular degeneration, diabetic retinopathy, and glaucoma). Deep learning is particularly suited to these conditions because their diagnosis and management is largely dependent on retinal photography (appendix 2 p 15) and optical coherence tomography (appendix 2 p 16). Artificial intelligence has many potential applications in eye health that could enhance delivery, optimise health system functioning, and lead to improved patient outcomes. Examples of potential uses that are in development include supporting point-of-care diagnostics,^277^, ^278^ surgical decision making (risk stratification),^279^ or patient management and treatment.^278^ The most likely early application in public health programmes will be in screening for diabetic retinopathy and retinopathy of prematurity.^280^, ^281^ Artificial intelligence solutions can possibly be integrated with electronic medical records to support administrative tasks such as identifying patients with a specific eye condition or at-risk patients.^282^ Artificial intelligence can be used in clinical, epidemiological, and health system research to incorporate data on social determinants of health, identify at-risk patient populations, and help to prioritise scarce clinical resources.^283^ This area presents an opportunity to address the link between poverty and blindness, and the need to promote equity in eye health.^10^ Finally, artificial intelligence coupled with telemedicine and mHealth could help to reach populations with poor health system access, either because of geographical isolation or scarcity of human resources for eye health.^280^, ^284^

There is little information on implementing artificial intelligence in eye health delivery and even less on whether it could improve care or outcomes. Before deployment into routine clinical practice, artificial intelligence applications require external validation on data that were not previously used in algorithm development and should undergo rigorous safety and efficacy testing in prospective clinical studies. In 2018, IDx-DR was the first artificial intelligence screening product for diabetic retinopathy to obtain approval from the FDA, after demonstrating good diagnostic accuracy in a prospective trial^281^ done in a real-world setting.

To assess the extent of translation-ready artificial intelligence (in use or soon to be in use), this Commission did a scoping review for publications related to artificial intelligence in eye health since 2015 to 2020 (appendix 1 p 71). We identified 1256 primary data reports, most (60%) focused on retinal imaging (20% diabetic retinopathy, 16% glaucoma, and 6% age-related macular degeneration). Only 12% of reports addressed conditions that affect the anterior segment and 1·5% were on childhood eye conditions. Most (90%), reported on artificial intelligence model development and internal validation. Of 113 reports on external validation or successful deployment in clinical settings, most were for retinal conditions (58%), facilitated by greater availability of images. Arguably, the greatest potential for artificial intelligence to contribute to advancing eye health would be to assist in case identification and health system efficiency, helping people with uncorrected refractive error and cataract access services, neither of which are currently well represented in artificial intelligence development platforms.

A substantial obstacle to artificial intelligence use in global eye health is the availability of large well curated datasets from multiple distinct populations for the development and validation of algorithms. Performance characteristics depend on the data on which algorithms are trained. This Commission did a global review to identify publicly available datasets of ophthalmic images.^285^ We identified 94 (including six with data from two world regions) open access datasets with more than 500 000 images from 23 countries; there were 13 datasets for which the country was not specified. 34 datasets originated from populations in Europe, 21 from southeast or east Asia, 16 from North America, nine from north Africa and the Middle East, four from south Asia, two from Latin America, and one from sub-Saharan Africa. The most common conditions were diabetic eye disease (35 datasets), glaucoma (19 datasets), and age-related macular degeneration (15 datasets). This under-representation of populations in LMICs is a new manifestation of the digital divide occurring in health, which we term health data poverty. The scarcity of representative datasets (public and other) limits the extent to which populations can benefit from digital health solutions and artificial intelligence systems. This limitation might lead to pronounced bias and failure of generalisability, with a risk of underperformance or even failure when transferred between settings and populations. We recommend that the visibility, accessibility, and use of existing public datasets is improved and that investment is made in developing new public datasets to support research, innovation, and validation in regions with insufficient health data.

### Sustainable financing for eye health

Better health financing is crucial to make progress towards universal health coverage. To expand effective coverage for all, countries need to raise sufficient funds for capital investment and ongoing service delivery costs; pool funds to spread the financial risks and protect the most vulnerable; allocate and use funds efficiently and equitably; and define the benefit package and rationing mechanisms.^286^ Mobilising financial resources is key for eye health in LMICs, and determines the scale, scope, and depth of coverage, quality, sustainability, and equity of eye health programmes.^287^

In many LMICs, eye health appears very underfunded. Data on eye health financing at a global and national scale are scarce. However, the national eye health system assessments done in the past decade indicate that in LMICs national health plans and budgets tend not to include eye care; national eye health plans, if developed, are often insufficiently funded, with eye health rarely considered in the allocation of resources; there is often little or no social insurance coverage for many eye health interventions; in many countries eye health programmes receive significant financial support from international non-governmental organisations and other development partners; and out-of-pocket payments for eye health are widespread.

#### Public spending

Public spending to increase coverage and financial protection is an important source of eye health funding, particularly for the poorest people. However, analysis of government expenditure on eye health is difficult as only a few countries make the data publicly available (appendix 2 p 17), partly because national budgets and health accounts tend not to delineate eye health explicitly and rather spread it across several categories.^288^ Increased and better allocated public funding is central to making progress towards universal health coverage by promoting coverage, equity, financial protection, and sustainability.

#### Prepaid financing schemes

The inclusion of eye health services in compulsory prepaid financing (such as social health insurance) could be an effective means to promote scale up and reach those most in need. In countries where eye care services are included in benefit packages under social health insurance, providers respond strongly to payment incentives, with positive and negative consequences. For example, in Thailand, higher payment for cataract surgery increased surgical rates.^289^ However, in other settings such as the Philippines and Indonesia, payment structures can contribute to cost escalation for schemes that have resulted in efforts to limit surgical volumes, sometimes with potential negative consequences for equity. When considering eye health services within social health insurance, who is covered and from where services can be purchased are crucial to access, efficiency, coverage, and financial protection. Specific measures might be required to ensure the most disadvantaged are reached, as illustrated in Rwanda (appendix 2 p 18).

#### Out-of-pocket payments and private care

Regardless of the financing in place, out-of-pocket exemptions need to exist for the poorest and most vulnerable communities and health insurance schemes need to provide for poor individuals. In many LMICs, user fees are applied across the eye health sector by all health-care providers (public, private, faith-based, non-governmental organisations) to partly or fully cover the costs. Such out-of-pocket payments can be a barrier to health care use, often for those needing it the most, and can push people into poverty.^290^, ^291^ Studies in sub-Saharan Africa and east Asia have found that patient costs for cataract surgery can be as high as half of the average annual household income.^292^, ^293^, ^294^ Even when cataract services are included in insurance schemes, out-of-pocket costs can remain high because of complex and often opaque arrangements on whether or how particular items are covered.^295^, ^296^ Understanding how these costs reduce access and contribute to financial hardship is important for eye health in universal health coverage. Private financing (out-of-pocket, employer schemes, and private health insurance) will continue to be a substantial source of eye health funding in many contexts in the foreseeable future; therefore, more evidence is needed on financing arrangements and interventions that can make eye care affordable to all.

#### External sources

Eye health is an important component of universal health coverage, largely neglected by bilateral and multilateral donors. This Commission reviewed data on official development assistance from bilateral, multilateral, and private philanthropy organisations (appendix 1 p 72). Between 2014 and 2018, the annual average external assistance for all eye health was estimated to be approximately US$102 million, amounting to less than 0·06% of the total global official development assistance. Around 66% of the US$102 million was spent on supporting the elimination of neglected tropical diseases that cause blindness, which reflects the great commitment by the US Agency for International Development, UK Aid, and several philanthropic organisations to tackle neglected tropical diseases. In view of the benefits to sustainable development, health, and wellbeing, we believe that the case for increased investment in eye health is strong, taking into consideration the crucial issues highlighted in our Grand Challenges exercise.

Several international non-governmental organisations provide substantial technical and financial support to LMICs. There is no comprehensive database that records all non-governmental organisation funding for eye health; therefore, estimating the total international funding is difficult. However, an analysis^297^ of annual reports suggested that 12 major non-governmental eye health organisations provided almost US$430 million in 2013, which included grants from bilateral and multilateral donors, and philanthropic organisations. Increased international support for eye health, in partnership with public and private actors, and vision-related international non-governmental organisations, would accelerate country-led efforts to move towards universal health coverage and fulfil the promise to leave no one behind. However, external funding needs to be considered as a supplement and not a replacement for government and other domestic expenditures on eye health.

#### Innovative financing

Although alternative financing mechanisms and private finance are unlikely to address all the unmet resource needs in eye health, they could provide substantial additional funding to progress eye health services rapidly within universal health coverage. The development of new alternative financing arrangements such as public–private partnerships (eg, Onesight), development impact bonds and loans, social enterprises, and results-based financing could help to fund the growing demand for eye care services (appendix 2 p 19). These initiatives will need to be evaluated on their ability to access new financial resources, their costs compared with other funding schemes, the predictability and sustainability of the financing, the effectiveness in delivering quality eye health outcomes, the financial protection they provide, and their ability to address inequities in access and use of eye health services.

#### More eye health for the money

More resources need to be raised to maximise impact, but optimising funding allocation on eye health services is also crucial. Evidence on how to allocate funding is scarce, so a data revolution is required to ensure “more eye health for the money”.^298^ This revolution would include more and better research and programme data to inform priority-setting and strategic purchasing. Topics for future research include costs and cost-effectiveness of interventions, relative efficiency of various delivery models, affordability (for governments and individuals), and financial barriers to accessing services (including direct and indirect costs). We also need to better understand how much funding is required and how much is being provided from different sources, which interventions are being supported, who is benefitting from them and who is being excluded.

#### Delivering financing for eye health

To improve eye health: (1) more resources from all sources are urgently required, particularly in LMICs; (2) eye care needs to be integrated into general health financing for universal health coverage; (3) financial resources must be used wisely and a commitment is needed from all partners to ensure more eye health for the money; (4) countries need to improve social health insurance schemes as eye health needs change; (5) strategic purchasing arrangements need to be made to provide an incentive for efficiency, quality, equity, and financial protection; (6) step-change in data and evidence is needed on the financing of eye care, the value for money of interventions, and financial barriers to access and use of services; and (7) alternative financing arrangements need to be investigated.

### Measuring progress in eye health

Strengthening eye health within universal health coverage requires clearly defined, scientifically robust indicators that capture key health system inputs, outputs, outcomes, and impacts. Indicators should provide insights to shape change and stimulate action. In this way, the indicator framework can be intrinsically linked to priorities, design, and continual improvement of services.

There are many potential indicators that could be used to monitor eye health services. For example, WHO has published several lists of eye health indicators in the past two decades: the 2002 framework for VISION 2020 included 35 indicators,^299^ the 2006–11 action plan had 29 core indicators and multiple additional indicators,^300^ the 2014–19 action plan included 19 indicators,^124^ and in 2017 WHO produced a list of 32 eye health indicators for the African region.^301^ The uptake and use of these indicators has been variable, probably because collecting such data is challenging and expensive.

To contribute to thinking on indicators, this Commission convened an international panel of 72 eye health system experts to participate in an indicator prioritisation exercise (appendix 1 p 73). We developed a menu of 22 indicators covering each stage of the results chain: inputs, outputs, outcomes, and impact (appendix 1 p 74–75). From these, seven core indicators were selected (table 4). These indicators were considered suitable to monitor universal access to quality affordable eye care services, including proxy measures for accessibility and affordability, and two effective service coverage indicators. We anticipate countries might wish to prioritise data collection for core indicators, facilitating regional and global comparisons of eye health progress within universal health coverage. Additional work is required to develop detailed indicator metadata, address gaps around specific conditions (eg, glaucoma), and consider how to measure the integration of eye health into the broader health system.

**Table 4:** Core indicators to monitor universal access to quality, affordable eye care services

	**Definition**	**Rationale**	**Data sources**	**Responsible entity**	**Comments**
**Accessibility of eye health services**					
Eye health facility density and distribution	Total numbers (public and private) of primary, secondary, tertiary, and low vision services per million population, by place of residence (urban or rural); additional subnational administrative or geographic divisions as relevant to setting	Place of residence should not be a barrier to accessing eye health services	Facility records, population data	Health ministry	Informs policy and planning about location of eye health services in relation to population density; outreach programmes might be planned according to gaps in geographic access to static services
Eye health worker density and distribution	Total numbers of ophthalmologist, optometrist, ophthalmic nurses, and other ophthalmic personnel per million population, by place of residence (urban or rural); additional subnational administrative or geographic divisions as relevant to setting	Availability and accessibility of eye health workers dictates access to care	Facility records, data from professional or regulatory bodies, population data	Health ministry	Informs policy and planning on recruitment and distribution of resources for eye health; known disparities exist in the number and distribution of trained eye care personnel between countries and by urban and rural settings within countries
**Affordability of eye health services**					
Coverage of national health finance pooling mechanisms that include eye care services	Proportion of population covered with health finance pooling mechanisms that include eye care services (considered individually): out-patient care, cataract, refractive error services, glaucoma treatment, and diabetic retinopathy treatment	Cost should not be a barrier to accessing eye care; proxy for WHO, World Bank, UHC financial risk protection indicators; catastrophic and impoverishing out-of-pocket payments unlikely to be discriminatory for monitoring affordability of elective eye care services	Health finance scheme reports and questionnaires	Health ministry	Informs policy about eye health financing and affordability; coverage within the lowest wealth quintile should be reported alongside the total population to monitor equitable coverage of eye health financing
Out-of-pocket payments for cataract surgery	Median (and range) of out-of-pocket payments made for cataract surgery as a proportion of median monthly household (or individual) income	Cost should not be a barrier to accessing eye care. Proxy for WHO, World Bank, UHC financial risk protection indicators; catastrophic and impoverishing out-of-pocket payments unlikely to be discriminatory for monitoring affordability of elective eye care services	Population-based surveys	Health ministry (surveys might be commissioned in collaboration with other stakeholders)	Informs policy about eye health financing and affordability; additional services could be monitored in the same way
**Effective coverage of cataract and refractive error services**					
Effective cataract surgical coverage	Among the population aged 50 years and older, people with operated cataract and good postoperative presenting visual acuity as a proportion of all people with operated cataract or operable cataract (disaggregated by sex or gender)	Sex-disaggregated or gender-disaggregated effective coverage measures UHC dimensions of access, quality, and equity for the leading cause of blindness globally	Population-based surveys	Health ministry (surveys might be commissioned in collaboration with other stakeholders)	Informs policy and planning about the met and unmet need for cataract surgical services; candidate WHO UHC tracer indicator.
Effective refractive error coverage	Adults with refractive error corrected to a predefined visual acuity threshold with habitual correction as a proportion of all people with corrected and uncorrected refractive error (disaggregated by sex or gender)	Sex-disaggregated or gender-disaggregated effective coverage measures UHC dimensions of access, quality, and equity for the leading cause of vision impairment globally	Population-based surveys	Health ministry (surveys might be commissioned in collaboration with other stakeholders)	Informs policy and planning about the met and unmet need for refractive error services; candidate WHO UHC tracer indicator
**Prevalence of vision impairment**					
Prevalence of vision impairment	The prevalence of all cause distance and near vision impairment (WHO definitions); disaggregation by key equity measures and by avoidable versus non-avoidable vision impairment	Proxy measure of eye health; a measure of programmatic success. Journey towards eye health as part of UHC	Population-based surveys	Health ministry (surveys might be commissioned in collaboration with other stakeholders)	Disaggregated vision impairment prevalence estimates inform policy makers about the impact of eye health systems on eye health among population subgroups

Seven core indicators identified through the indicator prioritisation exercise, done by this Commission. UHC=universal health coverage.

Ideal indicators to track outcome progress within universal health coverage capture coverage and quality of an intervention, with disaggregation to assess equity. These are referred to as effective service coverage indicators. WHO and the World Bank have selected a panel of 16 tracer indicators to monitor progress towards universal health coverage, including several effective coverage indicators.^302^

A useful framework for conceptualising effective coverage and indicators in general is the health-service coverage cascade model, first proposed by Tanahashi, and later revised by the Effective Coverage Think Tank Group and others.^303^, ^304^ This framework illustrates key dependencies and health system bottlenecks that prevent effective delivery of services (appendix 1 p 76). With each step down the cascade, additional members of the population in need do not progress towards the desired health outcome. Ideally, effective coverage measures are outcome-adjusted, reflecting the desired health impact. However, measuring the health outcome of interest is not always possible, particularly in chronic diseases, for logistical or cost reasons, or because of the delay between the intervention and realisation of the full health benefit. Therefore, earlier steps in the cascade are sometimes necessary to consider.

Intervention or service coverage indicators measure the proportion of the population that needs a service and receives it. This measure depends on those in need attending a service provider that has all the necessary inputs available (staff, equipment, consumables) to deliver the service. Considering service quality, how closely the delivery follows guidelines indicates how likely the service is to result in the desired health outcome. These are quality-adjusted coverage indicators. For interventions that require ongoing acceptance and use, adherence-adjusted or acceptance-adjusted coverage indicators can be applied. Proxy indicators are sometimes used, if service coverage indicators are not available, and these provide a measure of health service provision.^302^, ^305^ Measuring equity in service provision by disaggregating data and comparing subpopulations—eg, by wealth quintiles, education, sex or gender, age, ethnicity, and geographical location—is very important.^302^

Eye health outcomes are currently not reflected by the 16 tracer indicators outlined by WHO.^302^ However, two indicators, effective cataract surgical coverage and effective refractive error coverage, have been proposed for inclusion in the *Thirteenth General Programme Of Work* 2019–23 framework^306^ by WHO and endorsed in the *World report on vision*.^2^ Both conditions are common and account for around 77% of vision impairment and these two indicators also have a broader relevance to the health system. Cataract surgery, one of the most frequent operations in many settings, is a marker for the provision of surgical services and spectacle correction by refractive error services is a marker for the provision of assistive devices.

**Figure 19:**
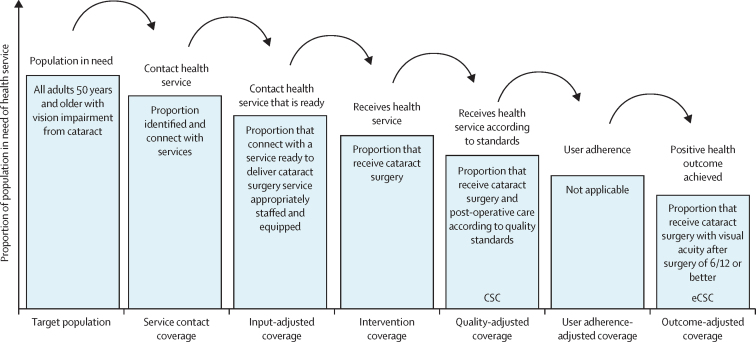
Effective coverage cascade for cataract surgical services CSC=cataract surgical coverage. eCSC=effective cataract surgical coverage.

Several indicators have been developed for cataract surgical services, which are defined in appendix 1 (p 76).^307^ Cataract surgical rate (the number of cataract operations per million population per year) has been widely used for many years as a measure of service output. Cataract surgical rate is used to set and track a desirable target to address the backlog and ongoing incidence of cataract. Cataract surgical outcome is a measure of visual acuity outcome and reflects the quality of surgery. It can be reported by the individual surgeon, health facility, or by a programme. Cataract surgical coverage is measured in cross-sectional population-based surveys, such as the RAAB survey. Effective cataract surgical coverage combines the proportion of population covered and the visual acuity outcome (figure 19).^308^ The term operable cataract defines the threshold of vision impairment required for an individual to be included in the population denominator of people with cataract, which is used for estimating cataract surgical coverage or effective cataract surgical coverage. RAAB surveys estimate these coverages using an operable cataract threshold of worse than 3/60, 6/60, or 6/18. Each country determines which threshold is most relevant to their context.

Effective refractive error coverage was proposed and described as a method in 2019.^2^, ^309^ Effective refractive error coverage assesses the proportion of people with refractive error who have received and use refractive error correction who achieve a specified visual acuity threshold (eg, 6/12); it takes into consideration the met, under-met, and unmet refractive error needs in a population (appendix 1 p 77). This method represents a major shift in the way refractive error is reported. Surveys previously focused only on counting unmet need (uncorrected refractive error) and excluded those who already had access to refractive error correction. An important consideration is whether spectacles are routinely used after being received, as reflected in the effective coverage cascade for refractive error services (appendix 1 p 77). This issue is particularly relevant for schoolchildren in many settings.^216^ When data for effective refractive error coverage are gathered using a population-based survey, the estimate considers people not adhering to spectacle use at that time as having an unmet need.

Using coverage cascade models provides helpful insights into appropriate coverage indicators and helps to identify key system blockages. Beyond effective cataract and refractive error coverage, examining service delivery effectiveness for other leading causes of vision impairment, such as glaucoma, diabetic retinopathy, and age-related macular degeneration is possibly more complex. We have outlined potential approaches for these three conditions in appendix 1 (p 78–79). Identifying the population in need can be challenging because these conditions might be asymptomatic in the early stages. Also, a successful outcome for each condition is to stabilise vision and prevent further vision or functional loss, which can only be confirmed after long follow-up between starting the service delivery and future observations. Therefore, an effective coverage indicator might be unrealistic for these conditions, possibly necessitating quality-adjusted service coverage indicators, at least in the medium-term.

### Progress towards universal eye health coverage

Despite the reduction in age-standardised prevalence of vision impairment observed in the past 20 years, the estimated number of people with vision impairment has risen with the continuously expanding and ageing global population. To explore progress towards improved eye health within universal health coverage in more depth, we focused on cataract, a key tracer condition with more population-level data than refractive error or other conditions.

We looked at peer-reviewed and grey literature since 2000 to 2020 for evidence on the delivery of cataract surgery in relation to the dimensions of universal health coverage: access, quality, financial protection, and equity (appendix 1 p 80).^310^ We also analysed all available RAAB datasets from the past 20 years to calculate the effective cataract surgical coverage, disaggregated by gender. We summarise the scope of reported literature for key cataract markers in appendix 1 (p 81).

**Figure 20:**
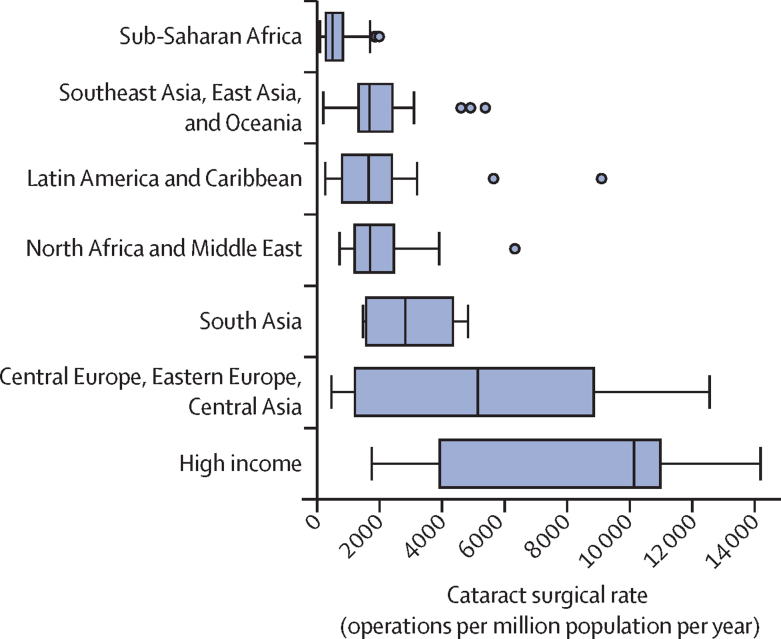
Cataract surgical rate by Global Burden of Disease super region Box and whisker plots; midlines are the median, boxes represent IQR, whiskers are upper and lower adjacent values. Outliers are plotted as individual dots.

Cataract surgical rate was the most frequently reported indicator with data from the past 10 years available for numerous regions. Cataract surgical rate data were available for 175 countries since 2000. Over the same period, the median cataract surgical rate varied considerably by GBD super region, from 494 in sub-Saharan Africa to 10 136 in high-income countries (figure 20). The global median cataract surgical rate was 1700 per million population per year from reports during the last decade (total range 95–14 188, IQR 720–3906). A desirable cataract surgical rate is context specific and depends on the unmet need, quality of service, population structure, population cataract incidence, and other factors.

There were 203 national or subnational survey reports of cataract surgical coverage, including 104 in the past decade. However, around a third of RAAB surveys done between 2000 and 2018 were not reported in the public domain, raising concerns over the selective non-reporting of less favourable coverage. Analysis of these additional datasets would contribute to a more complete epidemiological picture.

Cataract surgical outcome—a clinical marker of quality—was reported for 197 studies, which were mostly cross-sectional population-based surveys with variable time intervals between surgery and observation. We only considered surveys that reported presenting visual acuity (vision tested using spectacles if available). During the past 10 years most regions had two or fewer reports on cataract surgical outcome (appendix 1 p 81). The longstanding WHO benchmark for a good outcome, following cataract surgery, is a presenting visual acuity of 6/18 or better. This threshold was set more than 20 years ago with the expectation that it can be achieved in 80% or more surgeries.^311^ Among 82 reports on presenting visual acuity since 2010, the median proportion of people achieving 6/18 or better in the operated eye after surgery was 60% (total range 28–82%; IQR 50–68%). The median proportion of people with vision of 6/60 or worse in the operated eye after surgery was 18% (total range 3–51%; IQR 13–25%). The distribution of outcomes by GBD super region is shown in appendix 1 (p 83). Data from high-income countries are reported in many ways, which prevents comparisons. However, population-based data from Australia indicate that presenting visual acuity of 6/12 or better was achieved in around 80% of people, and clinic-based data from the UK indicate this number is closer to 90%.^312^, ^313^

Many surgeons audit their surgical outcomes. Collecting point-of-care outcome data is important to drive quality improvement. However, clinical outcome data collection is particularly challenging in LMICs, due to low postoperative follow-up rates. To investigate the validity of early postoperative data, which is more practical to collect, a large prospective study^314^ was done in 40 centres across ten countries in Asia, Africa, and Latin America. This study found that visual acuity measured during the first 3 days after cataract surgery was highly correlated with vision measured at 40 days or more after surgery. Consistent with previous findings, the final uncorrected visual acuity reached the WHO benchmark of 6/18 or better in only 64% of cases. Outcomes improved slightly with refraction, after which 85% had a best corrected visual acuity of 6/18 or better. Inadequate reporting of outcomes and frequency of poor results suggest that cataract surgical quality is not optimal and requires concerted action, with an emphasis on better integration with refraction services and strengthening of monitoring and reporting outcomes (appendix 1 p 84). Tools such as the Better Operative Outcomes Software Tool (BOOST), a mobile surgical outcome application, can facilitate the monitoring process.^315^

**Figure 21:**
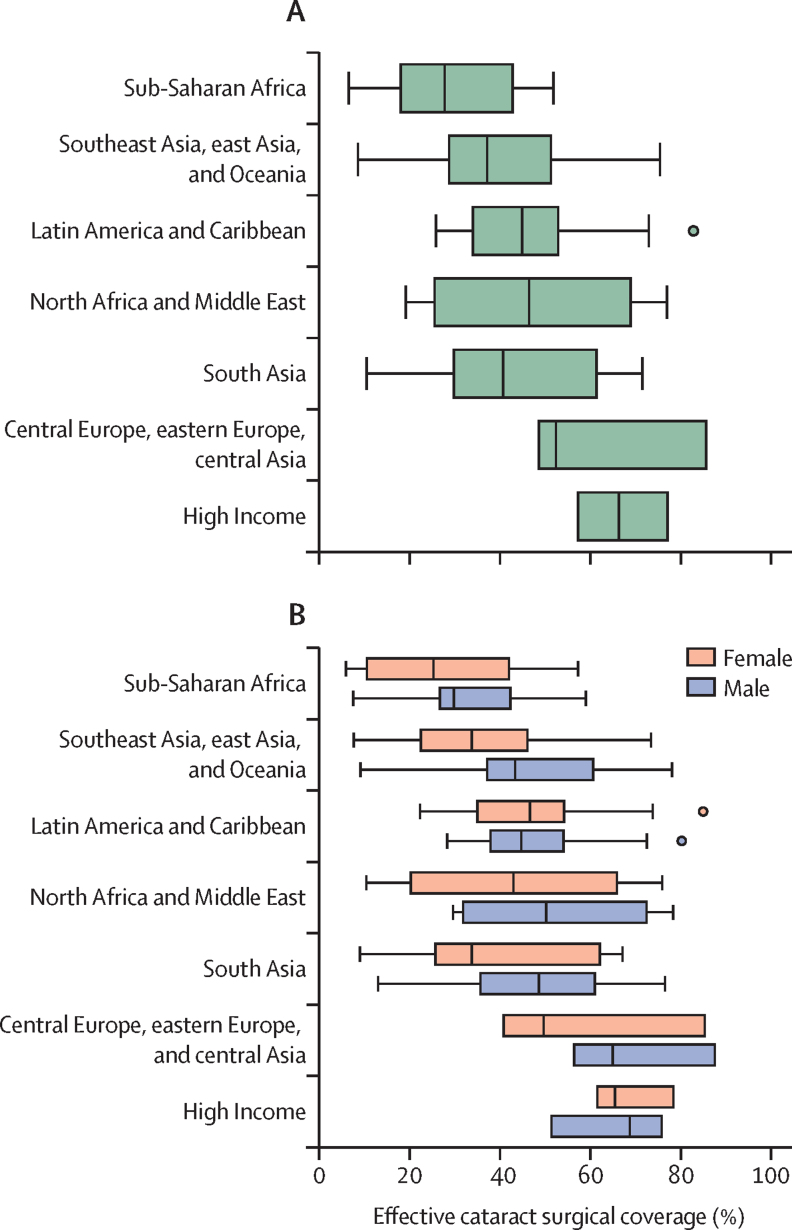
Effective cataract surgical coverage by Global Burden of Disease super region (A) Cluster-adjusted, age-adjusted, and sex-adjusted estimates. (B) Cluster-adjusted, age-adjusted, and sex-disaggregated estimates. Box and whisker plots; midlines are the median, boxes represent IQR, whiskers are upper and lower adjacent values. Outliers are plotted as individual dots. Countries within regions are represented by the most recent national or subnational Rapid Assessment of Avoidable Blindness survey and if two or more assessments occurred within 2 years, the median of estimates was used (appendix 1 pp 84–85).

The use of effective cataract surgical coverage (proportion of people aged 50 years or older with operated cataract or operable cataract who have a good postoperative presenting visual acuity) has only begun since 2017, with a total of 28 available reports representing few global regions (appendix 1 p 81). To supplement these reports and assess progress in the past two decades, we have reanalysed the data from 149 RAAB surveys from 48 countries (appendix 1 pp 84–85). We then selected the most appropriate estimate available for each country. Some caution needs to be exercised in the interpretation of these data as many are subnational surveys and have been extrapolated to represent coverage in the whole country for the purposes of this analysis. Between 2000 and 2019, the median effective cataract surgical coverage for 48 countries was 43·4% (total range 6·5–85·7%; IQR 29·4–59·2%), for an outcome of 6/18 or better and an operable cataract threshold of worse than 6/60. Some inter-regional variation is present, although the data from some areas are scarce (figure 21).

Systematic information on financial risk and protection in relation to eye health is rare, with only six reports worldwide from the past 20 years (appendix 1 p 81). For many people who are least able to pay, out-of-pocket payment for cataract surgery and presumably other procedures remains common.

Equitable access to eye health services has been assessed by disaggregating data, most frequently by gender, followed by place of residence, and socioeconomic status (appendix 1 p 81). These variables need to be routinely included in population-based surveys and analysed, to identify and inform the design and delivery of services for marginalised groups. Disaggregation of larger datasets, particularly for cataract surgical coverage, has highlighted disparities between men and women.^316^, ^317^, ^318^ We have disaggregated the results by gender (figure 21), showing that women consistently have lower effective cataract surgical coverage rates. For example, data from the Nigerian and Sri Lankan National Blindness Surveys^8^ have been disaggregated by gender, marital status, and area of residence (urban or rural), for the prevalence of cataract blindness and effective cataract surgical coverage (appendix 1 p 85). In both countries, the effective cataract surgical coverage was particularly low for women who were widowed living in rural areas. Further work in the design and delivery of services is needed to ensure more equitable outcomes among women.

There are fewer population-based data on effective refractive error coverage, which have mostly been collected through national eye health surveys or subnational surveys using the Rapid Assessment of Refractive Error (RARE). Several of these studies are summarised in table 5. Only one study was from a high-income country (Australia). The median effective refractive error coverage was 22% and the range was wide at 0–94%. Despite few data, clearly, at least in LMICs, the effective refractive error coverage is often low and represents a substantial unmet need. Existing data gaps need to be urgently addressed to understand the scale of needs, to inform programme implementation, and track progress. An updated RAAB survey protocol will be released in 2021, which will support the collection of additional visual acuity data outlined in appendix 1 (p 77), to enable estimation of effective refractive error coverage.^319^

**Table 5:** Population-based studies reporting effective refractive error coverage

	**Method**	**Age group, years**	Effective refractive error coverage (95% CI)*
Eritrea (Chan et al, 2013)	Subnational; RARE	15–50	22·2% (16·7–28·5)
Nigeria (Ezelum et al, 2011)	National eye health survey	≥40	3·4% (2·3– 4·4)
Tanzania (Mashayo et al, 2015)	Subnational; RARE	≥15	1·7% (0–3·3)
South Africa (Naidoo et al, 2016)	Subnational; RARE	15–35	51·4% (28·1–74·7)
Uganda (Nsubuga et al, 2016)	Subnational; RARE	15–50	6·0% (1·7–10·2)
Mozambique (Lougham et al, 2015)	Subnational; RARE	15–50	0·0%
Colombia (Casas Luque et al, 2019)	Subnational; RARE	≥15	50·9%
Iran (Fotouhi et al, 2006)	Subnational eye health survey	≥5	66·0%
Bangladesh (Bourne et al, 2004)	National eye health survey†	≥30	25·2%
Timor-Leste (Ramke et al, 2007)	Subnational; Modified RACSS‡	≥40	15·7%
Pakistan (Shah et al, 2008)	National eye health survey	≥30	15·1%
Australia (Foreman et al, 2017)	National eye health survey†	≥40	93·5% (92·0–94·8) for non-Indigenous; 82·2% (78·6–85·3) for Indigenous Australians

References can be found in appendix 1 (p 104). RACSS=Rapid Assessment of Cataract Surgical Services. RARE=Rapid Assessment of Refractive Error.

* 95% CI given if reported.

† An assumption of need, because the uncorrected visual acuity measurement was not given.

‡ Used a threshold of 6/18 (not 6/12) to define refractive error.

### Improving quality of eye care

Delivery of high quality services leading to good health outcomes is central to universal health coverage.^320^, ^321^ Unfortunately, high quality services are far from universal.^320^ Typically, clinical outcome measures such as visual acuity are used to quantify service effectiveness, and are tracked using population-based surveys. However, these provide a narrow understanding of the overall quality of care; a more holistic perspective is needed. A quality framework favoured by WHO^320^ considers seven components: effectiveness, efficiency, people centredness, safety, timeliness, equity, and integration. For the purpose of this Commission, in alignment with the SDGs, we have added planetary health as a component. Using cataract as an example, we examine approaches to improve the quality of eye care services.

Annually, an estimated 25 million cataract operations are done globally; making it the second most common surgical procedure after caesarean section.^322^, ^323^ However, from the available data on cataract surgical outcome and effective cataract surgical coverage, there is a major challenge in delivering effective and high quality services in many regions (figure 21). Moreover, these data present an overly optimistic view of the outcome because they define a good outcome following cataract surgery as 6/18 or better.^311^ However, 6/18 is still mild vision impairment. Since the WHO benchmark was set, cataract surgery has developed substantially with widespread adoption of small incision procedures and intraocular lens implantation. For these reasons, we recommend an update to the benchmark threshold of effectiveness for a good outcome, which should be a presenting visual acuity of 6/12 or better. This threshold would be used for reporting of surgeon-specific cataract surgical outcome and in the analysis of effective cataract surgical coverage in population-based surveys and would be expected to speed up efforts to improve quality and effectiveness.

**Figure 22:**
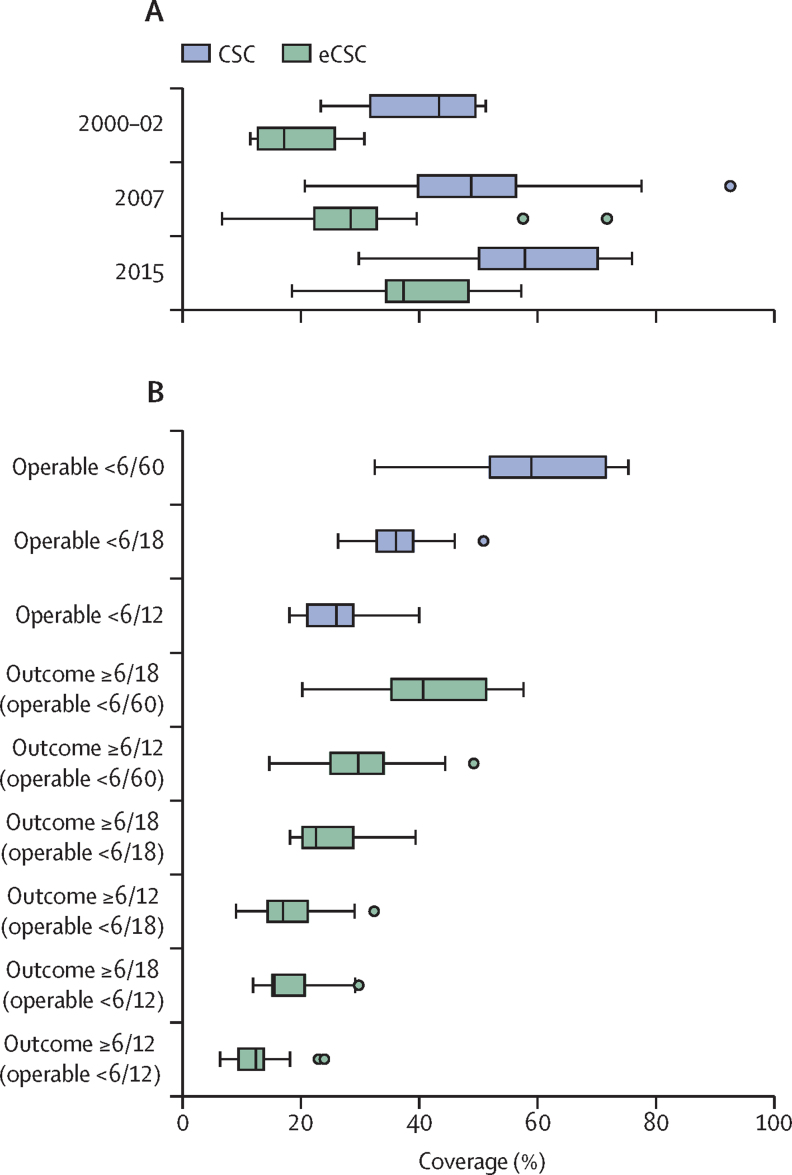
Cataract surgical coverage in Vietnam (A) Cluster-adjusted, age-adjusted, and gender-adjusted data from subnational Rapid Assessment of Avoidable Blindness surveys done in Vietnam in 2000–02 (n=8), 2007 (n=16), and 2015 (n=14), estimated for an operable cataract threshold of worse than 6/60 and a good clinical outcome of 6/18 or greater. (B) Data from 14 subnational surveys done in Vietnam in 2015. Data are plotted for three thresholds of operable cataract and two different levels of vision outcome: 6/18 or better and 6/12 or better. Box and whisker plots; midlines are the median, boxes represent IQR, whiskers are upper and lower adjacent values. Outliers are plotted as individual dots. CSC=cataract surgical coverage. eCSC=effective cataract surgical coverage.

The country with the largest number of RAAB or Rapid Assessment of Cataract Surgical Services surveys (38 between 2000 and 2015) is Vietnam. These surveys show rising cataract surgical coverage and effective cataract surgical coverage rates over the 15-year period (figure 22). Improvement in effective cataract surgical coverage seems partly attributable to increased use of intraocular lenses from 48% in 2000 to 98% in 2015.^324^ Notably, the effective cataract surgical coverage rates were substantially lower than the cataract surgical coverage rates, which indicates that the quality of surgical outcomes was not optimal. We have examined the effect of raising the effective good outcome threshold from the current 6/18 or better, to 6/12 or better (figure 22). We also examined the effect of changing the threshold for operable cataract, the population denominator, from the current worse than 6/60 to worse than 6/18 and 6/12. Both threshold changes lead to substantial reductions in effective cataract surgical coverage, which could drive action to increase coverage and quality of services.

There is abundant literature tracking the incremental technical development of cataract surgery, which usually restores vision in the absence of other ocular comorbidities. Two most commonly used surgical techniques to treat cataract are phacoemulsification and manual small-incision cataract surgery. Phacoemulsification is the standard of care in high-income countries and requires sophisticated equipment and more expensive intraocular cataract lenses and surgical consumables.

Manual small-incision cataract surgery uses a smaller incision compared with the older technique of sutured extracapsular cataract extraction, causes less surgically-induced astigmatism and achieves uncorrected visual acuity of 6/18 or better in more patients.^325^, ^326^ There has been a major shift from extracapsular cataract extraction to manual small-incision cataract surgery in many LMICs. Manual small-incision cataract surgery is now the most commonly done procedure in south Asia and sub-Saharan Africa.^327^, ^328^ Evidence from randomised controlled trials^329^, ^330^ suggest that people receiving manual small-incision cataract surgery have a small reduction in the chance of achieving uncorrected visual acuity of 6/18 or better after 6-8 weeks compared with phacoemulsification (pooled risk ratio 0·90, 95% CI 0·84–0·96), probably due to more surgically-induced astigmatism, but are likely to achieve similar levels of good best corrected visual acuity (0·99, 0·98–1·01). Complication rates are low with both techniques. The cost of phacoemulsification is two to four-times the cost of manual small incision cataract surgery.^329^, ^330^

The outcome of manual small-incision cataract surgery can be improved using routine ocular biometry to select an appropriately powered intraocular cataract lenses (which is not the standard in some regions),^327^ for meticulous adherence to the surgical technique, and for close integration of refractive and cataract services to prescribe spectacles. These steps are expected to eliminate the small outcome gap between phacoemulsification and manual small-incision cataract surgery. Overall, from the public health perspective and given the unmet need and limited financial and service capacity, this Commission cautions against recommending LMICs to transition from small-incision cataract surgery to phacoemulsification because, without a substantial increase in resources, this change could result in fewer people receiving treatment for cataract.

We reviewed published literature for interventions to improve the quality of cataract services (appendix 1 p 87).^331^ We searched for intervention studies that addressed one or more of the seven dimensions of quality, plus planetary health. We specifically excluded intraoperative intervention studies—eg, those that compared alternative procedures or intraocular lenses—because they have been thoroughly reviewed elsewhere.^329^, ^332^, ^333^ We identified 143 publications, largely (65%) from high-income countries (appendix 1 p 88). Most studies looked at interventions to improve efficiency and people centredness, with a smaller number examining effectiveness, safety, and equity. Appendix 1 (p 89) summarises several interventions found to improve efficiency, people-centredness, and effectiveness. These findings highlight that many interventions can improve the multidimensional quality of cataract surgery services. However, only a few of these studies were done in LMICs. This evidence gap needs to be addressed to guide approaches and improve service quality.

A 2018 *Lancet Global Health* Commission^321^ on high-quality health systems proposed a set of four universal actions for improving health care quality, which provides a useful framework for LMICs. The first universal action is to govern for quality through strong leadership and governance with clear policy, regulations, and accountability. This action is particularly relevant to eye health, as in many countries services are often provided in the private sector. The second action was to redesign service delivery to maximise the quality of care; there needs to be a thoughtful analysis of the appropriate location and specialised teams for service delivery to ensure the best possible outcome. Although there is a clear need to strengthen the delivery of basic eye health services in primary care settings, equally, specialist eye care services need to be appropriately staffed and equipped. The third action is to transform the workforce with intense focus on relevant competency-based training, strengthening health-care training institutions, and establishing enabling work environments. Urgent action is needed to develop and enable the eye health workforce to deliver quality care. The fourth action proposed is to ignite a demand for quality within the population by sharing information about quality and seeking active patient engagement to shape services that meet the needs of the population. This action is particularly relevant to ophthalmic surgery, with increasing publication and awareness of expected outcomes informing choices in some settings, with potential to extend to other areas.

#### Eye health and planetary health

Climate change is occurring, primarily mediated by greenhouse-gas emissions. Global health care is estimated to contribute to approximately 5% of all greenhouse-gas emissions.^334^ Eye care is a high volume service with a large number of consultations and procedures annually, and therefore, a substantial contributor to health-care emissions. With the ageing population increasingly requiring eye care interventions, we need to promote sustainable practices. We did a review to examine the extent and nature of the potential environmental impact of eye health services (appendix 1 pp 90–91). Evidence is scarce, with only eight reports meeting our criteria. A detailed carbon footprint of phacoemulsification cataract surgery has been estimated for individual centres in the UK and India.^61^, ^335^ For the same procedure, the UK centre produced 20-times more CO_2_ emissions than the Indian centre. To improve this field, tools are being developed to routinely measure environmental costs associated with cataract surgery as a mark of quality, alongside the other measures of high-quality services. Every aspect of practice can be examined and opportunities to reduce environmental impact can be identified.

### Increasing access and equity in eye care

Access to eye care is not equally distributed between and within countries, with marginalised and socially disadvantaged populations experiencing more difficulty in accessing the required care. This persistent inequity must be addressed for eye care to be realised within universal health coverage. Indeed, unless equity is prioritised, inequalities will probably increase in pursuit of universal health coverage, as the socially advantaged are more able to use new or improved services.^336^

In many high-income countries, people can access the eye care they need, although often the most marginalised groups such as Indigenous people or other minority ethnic groups are unable to access eye care, such as those in the USA (appendix 2 p 20). Another example is Australia, where most non-Indigenous Australians have access to good quality cataract surgery (effective cataract surgical coverage achieving 6/12 or better, 88·5%, 95% CI 85·2–91·2) compared with only half of Indigenous Australians (51·6%, 42·4–60·7).^312^ To explore how to improve access to eye care for these groups in high-income countries, we did two separate scoping reviews (appendix 1 p 92).^337^, ^338^ In addition, some of the key points for addressing eye health inequity for Indigenous Australians are described in appendix 2 (p 21).

**Figure 23:**
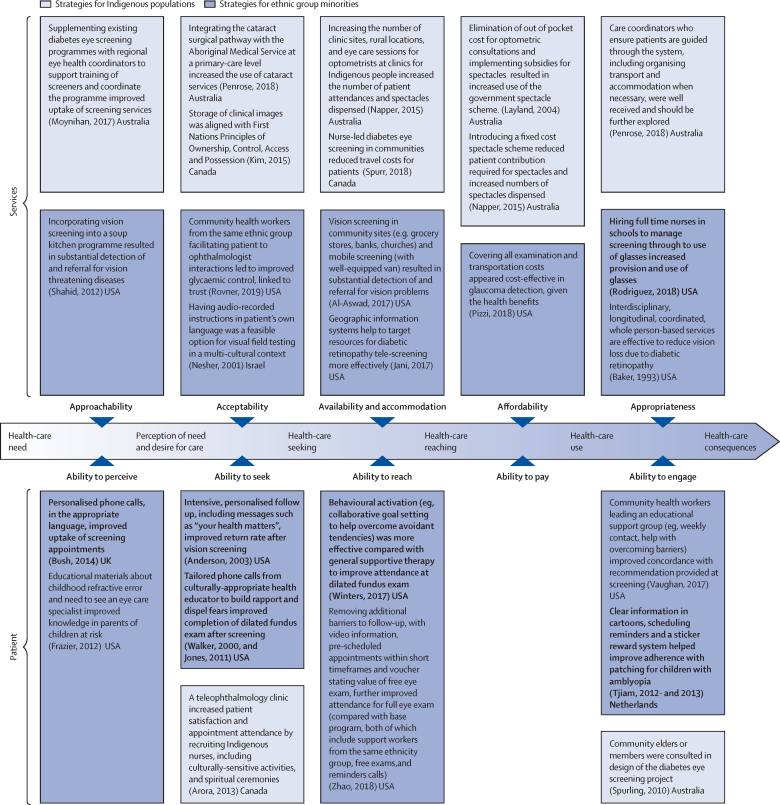
Strategies to improve access to eye care for Indigenous and other minority ethnic groups Identified from scoping reviews for Indigenous people and other non-dominant ethnic groups in high-income countries, mapped to the patient-centred access framework by Levesque et al.^339^ Randomised controlled trials are shown in bold. References can be found in appendix 1 (p 102).

In these reviews we identified 41 studies reporting strategies to improve access for Indigenous people, primarily in Australia (26 [63%]), and separately, we identified 67 studies reporting strategies for other minority ethnic groups, mainly in the USA (60 [90%]).^337^, ^338^ Strategies focused on diabetic retinopathy services were the most common (51% in the first review and 42% in the second). We mapped some of these strategies against a patient-centred health-care framework to show various ways in which access can be enabled through a pathway of having and perceiving a need for care; desiring, seeking, reaching, and using that care; and the subsequent health consequences (figure 23).^339^ The range of strategies outlined in figure 23 shows the complex nature of health-care access from the service and patient perspective, and the breadth of possible approaches to reduce inequity. Several of the most effective interventions addressed three or more access dimensions concurrently, often from the patient and service perspective. Despite the strong emphasis on reducing inequality in these approaches, only a third of studies in each review reported engaging the target communities during the design phase.

A promising strategy in pursuit of universal health coverage for eye care is proportionate universalism, which aims to improve outcomes for all population groups and specifically targets disadvantaged groups to ensure that improvement is proportional to need at the outset, showing the greatest benefit in these groups.^336^ The benefit of this approach was shown in maternal and child health, with countries using proportionate universalism to reduce inequality between the poorest and richest quintiles and improving coverage at the aggregate level compared with countries using other approaches.^340^ There are examples of the need for proportionate universalism in eye health, such as in Nigeria and Sri Lanka, where national surveys revealed that unmarried rural women (mostly widows) had disproportionate cataract blindness.^8^ The social distribution of cataract blindness in these two countries (appendix 1 page 86) highlights the need to allocate resources and action proportionate to need, with a particular focus on identifying strategies to improve access to care for women in rural areas with low social support.

Unfortunately, there are no published reports of proportionate universalism being applied in eye care. Indeed, there is little robust evidence on how to reduce inequity in eye health, including cataract services.^341^ We did an umbrella review of systematic reviews on gender and eye health. Only one of 58 included reviews described interventions to address gender inequality; the remaining reviews reported gender differences in the prevalence of eye conditions or access to cataract services (appendix 1 pp 93–94). To reduce this evidence gap, we did a modified Delphi process to identify priority groups and testable strategies to reduce inequity that can be assessed in future research (appendix 1 pp 95–96). Across two rounds we asked 183 participants worldwide to first nominate and then prioritise the groups which have the most difficulty and represent the largest number of people unable to access cataract services in their region, followed by the most promising strategies to improve access to screening and surgical services for cataract. Globally, three groups that need to be prioritised were identified as: people living in rural or remote areas, those with low socioeconomic status, and those with low social support. In most regions, data are not routinely collected on these characteristics in relation to vision loss from cataract and the access to services.

South Asia was the only region in which women were among the top three prioritised groups, despite all regions having more women than men living with vision loss. One explanation for women not being prioritised might be because they are not universally disadvantaged, with some women able to access the required eye care. Married women in Nigeria and Sri Lanka were found to be the subgroup with the lowest prevalence of cataract blindness (appendix 1 p 86).^8^ This disparity between different subgroups of women highlights the need to disaggregate by more than one sociodemographic factor, which provides a more nuanced understanding of the need distribution and where to target additional resources. Equity-relevant targets are also needed. Services often aim to deliver 50% of services to women to be equitable. However, worldwide there are more women than men with vision loss and equity-relevant service targets would reflect the disproportionate number of women in need of care (figure 10).

The globally prioritised strategies to improve access to screening and surgery involved improving the availability of services, improving integration, and reducing out-of-pocket costs, whereas participants from high-income countries prioritised efficiency, targeting at risk groups and cultural safety (appendix 1 pp 95–97). Some of these strategies have previously been described and evaluated, primarily those targeted to rural dwellers, people of low socioeconomic status, or women.^10^, ^49^ A common reflection of this literature is that a multifaceted approach is needed to address the diverse nature of barriers faced by socially disadvantaged groups. For example, outreach screening combined with counselling, providing transport, and low out-of-pocket costs for surgery increased the uptake in rural Tanzania and Kenya, whereas cataract case finders in Madagascar were not successful as the people identified with operable cataract had no means to reach the hospital.^243^, ^342^ Access and equity are crucial areas in which to develop better evidence in the coming decade.

Universal health coverage will not be realised without a deliberate effort to build equity into design. This needs to be informed by a thorough understanding of the groups that are being left behind and meaningful engagement with communities to co-design approaches that meet needs equitably. More representative leadership in global eye health is also needed, which was highlighted in our analysis of organisations working in this field (appendix 1 p 98).^343^

### Political prioritisation of global eye health

Over the past 30 years the eye health sector, including health ministry staff, civil society, eye health professionals, academics, and WHO, have worked hard to increase the global profile of eye health. Some good progress has been made—several World Health Assembly resolutions have been adopted,^124^, ^344^, ^345^, ^346^, ^347^ action plans implemented, and global coalitions formed.^124^, ^300^, ^348^ Despite these improvements, international and national political leaders have not sufficiently prioritised eye health, leaving it under-resourced and poorly integrated into national health systems.^2^

To analyse factors shaping global political prioritisation for eye health, we applied the framework developed by Shiffman and Smith (panel 6; appendix 1 p 99).^349^ This framework identifies four categories that shape political prioritisation in global health: power of the actors involved, ideas they use to portray the issue, the political context, and characteristics of the issue. The development of eye health services in China since 1949 provides an example of the impact that evolving political prioritisation can have on eye health service provision (appendix 2 p 22).

Panel 6 **Determinants of political priority in global eye health**

**Actor power: strength of individuals and organisations concerned with the issue**

*Policy community cohesion*

There is a strong degree of coalescence among the eye health community on the issue. However, the sector needs to build partnerships outside the sector.

*Leadership*

The sector has produced excellent programmatic leaders but has few system leaders or external champions for the cause.

*Guiding institutions*

WHO has provided some institutional leadership on vision as a health issue; however, such leadership has been largely absent within the broader UN system.

*Civil society mobilisation*

Civil society organisations have mobilised international and national political authorities to address the issue globally; however, eye health has been narrowly framed as a technical eye care issue and involved low public engagement.

**Ideas: the ways in which those involved understand and portray the issue**

*Internal frame*

Eye health actors generally agree on the definitions, causes, and solutions to blindness and vision impairment. However, they have not been sufficiently united in framing the issue.

*External frame*

Eye health has not been portrayed in a way that resonates with external audiences, especially with political leaders.

**Political contexts: the environments in which actors operate**

*Policy windows*

The development of Sustainable Development Goals (SDGs) was a missed opportunity and eye health was not included. The UN Decade of Action on the SDGs and the WHO *Thirteenth General Programme of Work* with triple billion targets, including universal health coverage, provides new policy windows in which to integrate eye health.

*Global governance structures*

Several World Health Assembly resolutions provide high aspirations for improving eye health. The International Agency for the Prevention of Blindness coordinates international efforts in blindness prevention, providing an effective platform for collective action with over 150 member organisations worldwide, including non-governmental organisations and civil societies, corporate organisations, professional bodies, and research and eye care institutions.

**Issue characteristics: features of the problem**

*Credible indicators and targets*

Previous indicators and targets have been clear and ambitious but not realistic and not linked to a clear pathway for achievement, undermining their credibility. New indicators and targets linked to universal health coverage are under development.

*Severity*

The scale of the burden is substantial. But eye health, when compared with other health issues, is often regarded as a second-order issue by governments, because of the historic scarcity of evidence on measures of impact such as mortality. The sector has not yet managed to gain policy traction for the economic and development case.

*Effective interventions*

Effective interventions are available and among some of the most feasible and cost-effective of all health-care interventions.

Many aspects suggest that global eye health is well placed to attract political support. Vision impairment is associated with mortality (figure 6), and eye care interventions are among some of the most feasible and cost-effective in health care. The issue is also global; impaired eye health and the need for eye care affect large numbers of people across all socioeconomic groups and the life course. There is broad agreement within the global eye health community on the policy agenda; partnerships have enabled the development and delivery of highly successful eye health programmes, such as for trachoma control. In addition, the International Agency for the Prevention of Blindness brings together a cohesive community of active organisations, giving the sector a platform and voice for collective action.

However, even when global and national political commitments towards eye health have been made, resources have not been provided at the scale and breadth required to meet growing demand. Several factors could explain this shortcoming. The case for the importance, severity, and ubiquity of eye health has not been made with sufficient force. Key opportunities have been missed; one notable example is that eye health is not referred to in the SDGs.

WHO has provided leadership, dedicated resources, and technical expertise to the great benefit of eye health, even though the institutional priorities set by WHO have fluctuated. Until the encouraging formation of the UN Friends of Vision group of Member States, such institutional leadership has been largely absent in the rest of the UN system. The missions of UNDP, UN Women, UNICEF, and the International Labour Organization all intersect and could be enhanced by better eye health, but generally vision-related activities have not been included in their work.

Internal and external framing of eye health has been ambiguous. Although the sector has argued at times for integration of eye care as part of the mainstream health agenda, many high-profile successes are positioned outside public health systems and are viewed as vertical in approach. This ambiguity has reduced the need to compete against other health issues and eye health has often been omitted from health system strengthening and universal health coverage agendas.

The role of national and international non-governmental organisations and donors in financing and delivering eye health services has sometimes had the unintended effect of discouraging government ownership and resource allocation. In some countries, non-governmental organisations and donors are outside of government systems, limiting the responsibility and accountability of national actors. Some governments have come to wholly rely on the non-government sector for eye health service delivery and therefore have not engaged nor allocated their own resources to eye care within health system strengthening and universal health coverage.

This problem is changing. Increasingly the eye health sector has made a concerted effort to position vision within mainstream health policy, emphasising the need to embed eye care in national health systems and primary health care. Crucially, the WHO *World report on vision*^2^ recommends that eye health should be part of every country's journey towards universal health coverage.

The COVID-19 pandemic will probably transform the importance of health and health systems within government policy in general. Although long-term effects will take time to be understood, conceivably, there will be renewed emphasis on building resilient and responsive health systems. Following the pandemic, eye health needs to be considered as an essential part of health and associated health service packages. However, the pandemic will probably lead to deterioration in the social determinants of eye health for many, through increased poverty and reduced access to services in many countries.

Eye health cuts across multiple SDGs. The Decade of Action and delivery of SDGs, called for by the 2019 UN General Assembly, presents a new opportunity to position eye health equity as an integral part of the development agenda and to have it addressed by broader development institutions. This action would enable the case to be made at national level, to include eye care in economic and development plans, budgets and consolidate its place within health plans for universal health coverage. To integrate vision in the development agenda will require three major actions.

First, eye health needs to be clearly framed as a development issue, which would have substantial and immediate benefits for prosperity and social progress. This Commission shows that addressing vision impairment is a realistic and highly cost-effective way of unlocking human potential, enabling children to gain an education and working-age adults to get and keep a job, and improving equality for women and girls who are more likely than men to have poor vision and less likely to receive treatment. These steps are more likely to resonate with political leaders, donors, and international institutions.

Second, cross-sectoral partnerships need to be built, including economic development, education, women's empowerment, business and transport, civil society, the technology, and private sectors. These partnerships are already taking place in some regions but will need to become much more widespread.

Third, different kinds of leadership skills and capabilities need to be developed. The sector has produced many committed and effective leaders who excel at designing and managing eye health programmes. However, achieving progress across the development agenda will require leaving current approaches behind. A more innovative and adaptive approach that engages broad networks of diverse stakeholders is required. Leaders will need to be able to connect the whole system together.

### Call to action

We call on the global community to consider the recommendations outlined in the Key messages panel for urgent action. Vision is an enabling tool for sustainable development, accelerating delivery of the SDGs. We have presented the benefits of vision for everyone and supported these by evidence. Vision as an integral component of health has been insufficiently represented in the targets of the SDGs but the Decade of Action, called for by the 2019 UN General Assembly, presents an opportunity to reintegrate eye health into developmental and economic plans. To achieve this transition, the sector must build the right bridges, engage in new partnerships, and train new leaders skilled in systems change, which will be a global challenge for the next decade.

Investing in universal eye health is a realistic, cost-effective way of unlocking human potential by improving health and wellbeing, education, work, and the economy; it is essential to achieving the SDGs.
