# Supplementary material for: The Lancet Global Health Commission on Global Eye Health: vision beyond 2020
Source: Lancet Glob Health. 2021 Feb 16;9(4):e489–551. doi: 10.1016/S2214-109X(20)30488-5 (PMC7966694; doi:10.1016/S2214-109X(20)30488-5)
Supplement: Supplementary appendix 1 [file mmc1.pdf]

THE LANCET
Global Health

Supplementary appendix 1

This appendix formed part of the original submission and has been peer reviewed. We post it as supplied by the authors.

Supplement to: Burton MJ, Ramke J, Marques AP, et al. The *Lancet Global Health* Commission on Global Eye Health: vision beyond 2020. *Lancet Glob Health* 2021; published online Feb 16. http://dx.doi.org/10.1016/S2214-109X(20)30488-5.

The Lancet Global Health

Supplementary appendix 1

The appendix formed part of the original submission and has been peer reviewed.
We post it as supplied by the authors.

Supplement to: Burton MJ, Ramke J, Marques AP, et al. Lancet Global Health Commission on Global Eye Health: Vision Beyond 2020

Table of Contents

INTRODUCTION **5**

COMMON ABBREVIATIONS 5
COUNTRIES LISTED BY GBD SUPER REGIONS AND GBD REGIONS 6
VISION 2020: THE RIGHT TO SIGHT 7
LESSONS FROM THE DEVELOPMENT OF GLOBAL EYE HEALTH 8

SECTION 1: THE EYE, VISION IMPAIRMENT AND CONDITIONS **9**

SUPPLEMENTARY FIGURE 1: MEASURING VISUAL FUNCTION 9
DEFINING VISION IMPAIRMENT 10
SUPPLEMENTARY TABLE 1: COMMON EYE CONDITIONS CAUSING VISION IMPAIRMENT 11

SECTION 2: THE IMPORTANCE OF EYE HEALTH **15**

SCOPING REVIEW ON EYE HEALTH AND THE SUSTAINABLE DEVELOPMENT GOALS 15
Summary of methods *15*
VISION IMPAIRMENT AND INCLUSIVE DEVELOPMENT 16
Supplementary Table 2: Educational attainment of children with vision impairment as their main special educational need in England and Wales, 2016 ..... *16*
Supplementary Figure 2: International Classification of Functioning, Disability and Health. *16*
UMBRELLA REVIEW OF REVIEWS ON EYE HEALTH AND QUALITY OF LIFE 17
Summary of methods *17*
Supplementary Figure 3: Quality of Life Umbrella Review Summary *18*
RAPID REVIEW ON VISION IMPAIRMENT, NON-COMMUNICABLE DISEASES AND MENTAL HEALTH 19
DEMENTIA AND VISION IMPAIRMENT 20
SCOPING REVIEW ON DUAL SENSORY IMPAIRMENT 21
Summary of methods *21*
Summary of findings *21*
SYSTEMATIC REVIEW ON DRIVING AND VISION IMPAIRMENT 22
Summary of methods *22*
SYSTEMATIC REVIEW OF VISION IMPAIRMENT AND FALLS 23
Summary of methods *23*
SYSTEMATIC REVIEW ON VISION IMPAIRMENT AND MORTALITY 24
Summary of methods *24*

SECTION 3: MAGNITUDE OF EYE DISEASE **25**

GLOBAL MAGNITUDE VISION IMPAIRMENT IN 2020 25
Supplementary Table 3: Number of people affected and crude and age-standardised prevalence of vision impairment, by Global Burden of Disease Region in 2020 ..... *26*
Supplementary Table 4: Global and regional leading causes of blindness in 2020 ..... *27*
Supplementary Table 5: Global and regional leading causes of moderate and severe vision impairment in 2020 *28*
MAGNITUDE AND CAUSES OF VISION IMPAIRMENT IN CHILDREN 29
Methodology for estimating the number of children who are blind globally in 2020. ..... *29*
Supplementary Table 6: Prevalence estimates for blindness and severe visual impairment using under 5 mortality rates as a proxy *29*
Supplementary Table 7: Number of children who are blind by Global Burden of Disease Region, 2020 ..... *30*
Supplementary Figure 4: Number of children who are blind by Global Burden of Disease Region, 2020 ..... *30*
Supplementary Figure 5: Schema of the number and main causes of blindness in children per 10 million population, by level of socio-economic development *31*
TEMPORAL TRENDS IN VISION IMPAIRMENT 32
Supplementary Table 8: Gender differences in the age-standardised prevalence of blindness and moderate and severe vision impairment. *32*
Supplementary Figure 6: Gender differences in the age-standardised prevalence of blindness and moderate and severe vision impairment. *32*
Supplementary Table 9: Excess number of women over men with blindness and moderate and severe vision impairment. *33*
Supplementary Table 10: Global and regional numbers of adults (aged 20 – 79 years) estimated to have DM, DR and VTDR in 2019 and projected for 2045. ..... *33*
Supplementary Table 11: Global myopia trends, 2000 to 2050 ..... *34*

Supplementary Figure 7: Global myopia trends, 2000 to 2050 .... *34*
NON-VISUALLY IMPAIRING OCULAR CONDITIONS: CONTRIBUTION TO GLOBAL EYE CARE NEED 35
Supplementary Figure 8: The visual acuity level of people attending secondary eye hospitals in India. *35*
Supplementary Table 12: The visual acuity level of people attending secondary eye hospitals in India. *35*
QUANTIFYING THE MAGNITUDE: SURVEY METHODOLOGIES 36
Supplementary Table 13: Comparison of comprehensive and rapid survey methodologies *37*
EYE HEALTH SURVEY DATA GAPS 38
Supplementary Figure 9: Population-based eye health surveys conducted worldwide (2000-2020) *38*
REPORTING EYE HEALTH SURVEY DATA 43
DISABILITY WEIGHTS FOR VISION IMPAIRMENT 44
Supplementary Table 14: Summary of studies estimating a disability weight for blindness and vision impairment *44*

SECTION 4: ECONOMICS OF VISION 45

SYSTEMATIC REVIEW OF THE ECONOMICS OF VISION IMPAIRMENT AND EYE HEALTH 45
Summary of methods *45*
Summary of the PICOS elements for the systematic review *45*
PRISMA flow diagram for systematic review of the economics of vision impairment and eye health *46*
Description of the studies *47*
Supplementary Table 15: Health economic studies by GBD Super Region and condition *48*
Supplementary Table 16: Economic studies for general vision impairment and specific conditions. *49*
Supplementary Table 17: Comparison of global productivity losses from vision impairment. *50*
GLOBAL PRODUCTIVITY LOSSES FROM VISION IMPAIRMENT IN 2020 51
Summary of methods *51*
Supplementary Table 18: Relative reduction in employment among people with VI (%) *52*
COST-EFFECTIVENESS OF INTERVENTIONS FOR CATARACT AND REFRACTIVE ERROR 54
Summary of methods *54*
Supplementary Table 19: Cost effectiveness ratios for cataract surgery (presented in Figure 13) *56*
Supplementary Table 20: Cost effectiveness ratios for refractive error services (presented in Figure 14) *57*

SECTION 5: GLOBAL EYE HEALTH RESEARCH 58

TWENTY YEARS OF EYE HEALTH RESEARCH 58
Summary of methods *58*
Supplementary Table 21: Summary of the research focus and region where it was conducted, 2000 to 2019 .... *59*
ANALYSIS OF RANDOMISED CONTROLLED TRIALS ON EYE HEALTH CONDUCTED IN SUB-SAHARAN AFRICA 60
Summary of methods *60*
Supplementary Table 22: Location and topic of RCTs conducted in Sub-Saharan Africa 2000-2019 .... *61*
DIVERSITY OF EDITORIAL BOARDS OF OPHTHALMOLOGY JOURNALS 62
Summary of methods *62*
Supplementary Table 23: Gender of editors and Editors in Chief of ophthalmology and optometry editorial boards, 2019/2020 .... *62*
GRAND CHALLENGES IN GLOBAL EYE HEALTH 63
Summary of methods *63*

SECTION 6: BEYOND 2020 - DELIVERING HIGH-QUALITY UNIVERSAL EYE CARE 64

CONCEPTUALISING EYE HEALTH WITHIN UNIVERSAL HEALTH COVERAGE 64
Supplementary Figure 10: Universal Health Coverage *64*
DELIVERING INTEGRATED PEOPLE-CENTRED EYE CARE WITHIN UHC 65
Supplementary Table 24: Eye health service components relevant to low-resource, medium-resource and high-resource settings, delivery platform, human resources and integration. *65*
HUMAN RESOURCES FOR EYE HEALTH: BUILDING THE EYE HEALTH TEAM 68
Supplementary Table 25: The eye health team *68*
Supplementary Figure 11: Ophthalmologists per million population, by world region *69*
Supplementary Table 26: Vision impairment and the eye health workforce *70*
INNOVATING DELIVERY: TECHNOLOGY TO SUPPORT EYE HEALTH WITHIN UHC 71
Artificial Intelligence solutions for eye health: a scoping review *71*
Citation for The Commission’s review to identify publicly available datasets of ophthalmic images *71*
SUSTAINABLE FINANCING FOR EYE HEALTH 72
ODA Data Analysis *72*

MEASURING PROGRESS IN EYE HEALTH WITHIN UHC 73
Indicator prioritisation for eye health within UHC *73*
Supplementary Table 27: Indicators for eye health within UHC *74*
Supplementary Figure 12: Cascade for measuring effective coverage of health care services *76*
Supplementary Table 28: Definitions for cataract surgical service indicators *76*
Supplementary Figure 13: Flow diagram illustrating how Effective Refractive Error Coverage (eREC) is estimated. *77*
Supplementary Figure 14: Health service effective coverage cascade for refractive error services *77*
Supplementary Figure 15: Health service effective coverage cascade for glaucoma, diabetic retinopathy and AMD *78*
PROGRESS TOWARDS UHC FOR EYE HEALTH IN 2020 80
Are we advancing Universal Health Coverage through cataract services? A scoping review *80*
Supplementary Table 29: Reporting on progress towards UHC for eye health; numbers of studies, by region, examining Access, Quality, Financial Protection and Equity for cataract surgery *81*
Supplementary Table 30: Cataract Surgical Rate, by GBD Super Region *82*
Supplementary Table 31: Cataract surgical outcome by GBD Super Region *82*
Supplementary Figure 16: Cataract surgical outcome by GBD Super Region *83*
Enhancing cataract services by better integration with refractive error services *84*
Summary of methods for the calculation of eCSC from RAAB data *84*
Supplementary Figure 17: Disaggregated prevalence of effective cataract surgical coverage (eCSC) in the Nigeria National Survey (2005-2007) and Sri Lanka National Survey (2012-2014). *85*
Supplementary Figure 18: Disaggregated prevalence of cataract blindness in the Nigeria and Sri Lanka National Eye Health Surveys. *86*
BUILDING THE QUALITY OF EYE CARE 87
Summary of methods *87*
Supplementary Table 32: The extent of evidence for interventions addressing each quality element of cataract services, by GBD super-region *88*
Supplementary Table 33: Studies reporting interventions to improve quality of cataract surgery (excluding surgical procedure) *89*
EYE HEALTH AND PLANETARY HEALTH 90
Summary of methods *90*
Summary of findings *90*
INCREASING ACCESS AND EQUITY IN EYE CARE 92
Models to improve access to eye care for Indigenous people in high-income countries: a scoping review *92*
Interventions to promote access to eye care for non-Indigenous, non-dominant ethnic groups in high-income countries: a scoping review *92*
GENDER AND EYE HEALTH: OVERVIEW OF SYSTEMATIC REVIEWS 93
Summary of methods *93*
Supplementary Table 34: Gender and eye health, distribution of primary studies included in overview of systematic reviews, by topic and GBD Super-region *94*
Addressing inequity in cataract surgical services – a modified Delphi process *95*
Supplementary Figure 19: Addressing inequity in cataract surgical services *97*
Equity, Diversity and Inclusion in global eye health leadership *98*
POLITICAL PRIORITISATION OF GLOBAL EYE HEALTH 99
REFERENCES FOR FIGURES, TABLES AND PANELS 100
References for Figure 6: Vision impairment and mortality *100*
References for Figure 13: Cost-effectiveness ratios for cataract surgery *101*
References for Figure 14: Cost-effectiveness ratios for refractive error services *101*
References for Figure 23: Strategies to improve access to eye care for Indigenous and other non-dominant ethnic groups *102*
References for Table 2: Eye health and the Sustainable Development Goals *103*
References for Table 5: Population-based studies reporting Effective Refractive Error Coverage (eREC) *104*
References for Panel 1: Vision impairment and dementia *105*
References for Panel 2: The changing epidemiology of eye disease *106*
References for Panel 5: The challenge of glaucoma *109*

REFERENCES 110

Introduction

Common abbreviations

AMD	Age-related macular degeneration
BCVA	Best-corrected visual acuity
DALY	Disability-adjusted life year
DR	Diabetic retinopathy
GBD	Global Burden of Disease
HIC	High-income countries
IOL	Intraocular lens
IOP	Intraocular pressure
IPEC	Integrated people-centred eye care
LIC	Low-income countries
LMIC	Low- and middle-income countries
MIC	Middle-income countries
MSVI	Moderate and severe vision impairment
NCD	Non-communicable disease
NGO	Non-Governmental Organisation
NTD	Neglected tropical disease
ODA	Official Development Assistance
PEC	Primary eye care
PHC	Primary health care
PVA	Presenting visual acuity
QALY	Quality adjusted life years
QoL	Quality of life
SDG	Sustainable Development Goals
UHC	Universal health coverage
URE	Uncorrected refractive error
VA	Visual acuity
VI	Vision impairment
WHA	World Health Assembly
WHO	World Health Organization
WRV	World Report on Vision

Countries listed by GBD Super Regions and GBD Regions

Throughout the report we have categorised countries according to the Global Burden of Disease (GBD) Super Regions (7) and GBD Regions (21) as outlined by the Institute for Health Metrics (2017). These are listed here:

Central Europe, Eastern Europe and Central Asia
Central Europe: Albania, Bosnia and Herzegovina, Bulgaria, Croatia, Czech Republic, Hungary, Macedonia, Montenegro, Poland, Romania, Serbia, Slovakia, Slovenia
Eastern Europe: Belarus, Estonia, Latvia, Lithuania, Moldova, Russian Federation, Ukraine
Central Asia: Armenia, Azerbaijan, Georgia, Kazakhstan, Kyrgyztan, Mongolia, Tajikistan, Turkmenistan, Uzbekistan

High-income
Australasia: Australia, New Zealand
High-income Asia Pacific: Brunei Darussalam, Japan, South Korea, Singapore
High-income North America: Canada, United States
Southern Latin America: Argentina, Chile, Uruguay
Western Europe: Andorra, Austria, Belgium, Cyprus, Denmark, Finland, France, Germany, Greece, Iceland, Ireland, Israel, Italy, Luxembourg, Malta, Netherlands, Norway, Portugal, Spain, Sweden, Switzerland, United Kingdom

Latin America and Caribbean
Andean Latin America: Bolivia, Ecuador, Peru
Caribbean: Antigua and Barbuda, Bahamas, Barbados, Belize, Cuba, Dominica, Dominican Republic, Grenada, Guyana, Haiti, Jamaica, Puerto Rico, Saint Lucia, Saint Vincent and the Grenadines, Suriname, Trinidad and Tobago
Central Latin America: Colombia, Costa Rica, El Salvador, Guatemala, Honduras, Mexico, Nicaragua, Panama, Venezuela
Tropical Latin America: Brazil, Paraguay

North Africa and Middle East
North Africa and Middle East: Afghanistan, Algeria, Bahrain, Egypt, Iran, Iraq, Jordan, Kuwait, Lebanon, Libyan Arab Jamahiriya, Morocco, Occupied Palestinian Territory, Oman, Qatar, Saudi Arabia, Sudan, Syrian Arab Republic, Tunisia, Turkey, United Arab Emirates, Yemen

South Asia
South Asia: Bangladesh, Bhutan, India, Nepal, Pakistan

Southeast Asia, East Asia and Oceania
East Asia: China, Dem. People´s Republic of Korea, Taiwan
Southeast Asia: Cambodia, Indonesia, Lao People´s Democratic Republic, Malaysia, Maldives, Mauritius, Myanmar, Philippines, Seychelles, Sri Lanka, Thailand, Timor-Leste, Vietnam
Oceania: Fiji, Kiribati, Marshall Islands, Micronesia, Papua New Guinea, Samoa, Solomon Islands, Tonga, Vanuatu

Sub-Saharan Africa
Central Sub-Saharan Africa: Angola, Central African Republic, Congo, Democratic Republic of the Congo, Equatorial Guinea, Gabon
Eastern Sub-Saharan Africa: Burundi, Comoros, Djibouti, Eritrea, Ethiopia, Kenya, Madagascar, Malawi, Mozambique, Rwanda, Somalia, Uganda, United Republic of Tanzania, Zambia
Southern Sub-Saharan Africa: Botswana, Lesotho, Namibia, South Africa, Swaziland, Zimbabwe
Western Sub-Saharan Africa: Benin, Burkina Faso, Côte d´Ivoire, Cameroon, Cape Verde, Chad, Gambia, Ghana, Guinea, Guinea-Bissau, Liberia, Mali, Mauritania, Niger, Nigeria, São Tomé and Príncipe, Senegal, Sierra Leone, Togo

VISION 2020: The Right to Sight

VISION 2020 was set up to eliminate avoidable blindness by 2020. It was formed from a partnership between WHO and a Task Force of the IAPB and launched in 1999. The rationale was that the number of people blind in the world was increasing due to population growth, ageing and inadequate eye care services in many LMICs; yet 80% of all blindness was preventable or treatable with proven cost-effective interventions. The strategy that was developed and followed was to developing primary and secondary level eye care services at the district level of health care through human resource and infrastructure development to address the major diseases causing avoidable blindness, which at that time were: cataract, refractive error and low vision, trachoma, onchocerciasis and blindness in children.

Achievements:

- ***Improved coordination and focus*** on priorities to improve eye care and reduce blindness.
- ***Advocacy*** for improved eye care at global and national level with policy makers, health planners and eye care professionals.
- ***Resource mobilisation*** for eye care from national governments, Official Development Assistance (ODA) donor governments, international Non-Governmental Organisations (NGOs) and the corporate sector including drug donation programmes for trachoma and onchocerciasis.
- ***Programme planning and implementation***, increased training of eye care workers, improvements in infrastructure and technology for eye care, and disease control programs including elimination of trachoma and onchocerciasis.

Lessons from the development of Global Eye Health

Lesson 1 – the importance of advocacy in creating global platforms to address a public health issue. After the establishment of WHO in 1948 the first public health activity in eye health started in 1952 with a Task Force on Trachoma. Subsequently, a public health approach to infective and nutritional eye diseases, particularly trachoma, onchocerciasis and vitamin A deficiency led to global and regional programmes at the community level and the concept of *community eye health*. In the 1970s the International Agency for the Prevention of Blindness (IAPB) was formed and WHO established a programme to prevent blindness (PBL).

Lesson 2 – the importance of common definitions, high quality data and scientific evidence on which to develop global, regional and national health programmes. Since the 1970s population-based surveys have collected data to assess the magnitude and causes of vision impairment[1,2]; definitions were updated to reflect actual need for eye care[3,4]; national programmes for prevention of blindness were established and promoted in many countries, and effective interventions for the control of the common blinding diseases were described (cataract, trachoma, vitamin A deficiency, onchocerciasis).[5] All these activities led to a better understanding of the problem of blindness and vision impairment worldwide, and develop rationale solutions tailored to needs.

Lesson 3 – the importance of identifying and addressing specific eye diseases of public health importance which can be eliminated through public-private partnerships. Several major causes of blindness lent themselves to prevention, using a public health approach. Global and regional disease-specific control programmes were initiated, based on the evidence and facilitated by public–private health partnerships (e.g. drug donation programmes) and international development financing. These include the African Programme for Onchocerciasis Control and WHO Alliance for the Global Elimination of Trachoma by 2020 (GET2020). Corneal blindness in young children is addressed through vitamin A supplementation and measles vaccine delivered as part of child health programmes.

Lesson 4 – the VISION 2020 initiative created an easily understood message for advocacy and planning services, and a global partnership involving different stakeholders in public health, including the private sector and non-governmental organisations, which resulted in extra resources and a common goal and focus. There are many "not for profit" (non-government) eye care organisations and providers. Initially a coalition of these organisations (later under the umbrella organisation IAPB working with WHO) developed and launched in 1999 a Global Initiative called "VISION 2020: The Right to Sight" with the goal of eliminating avoidable blindness by 2020.[5,6] This global initiative has had success in advocacy for eye health, some resource mobilisation, and in creating a common focus among a variety of stakeholders for eye care programme activities. The VISION 2020 planning approach has been used in many countries, alongside a range of other reforms, to deliver significant improvements in eye health services. Yet, avoidable blindness has not been eliminated.

Lesson 5 – in promoting a global programme, too little attention was given to engagement and partnership with national ministries of health to ensure national ownership of the challenges, the need to integrate eye care planning and resource allocation into national health systems and plans, and also to share in the achievements and successes. At the same time the strong involvement of the not-for-profit sector resulted in some low-income countries considering that eye care was being addressed by these external agencies and therefore the government could use its limited resources on other priorities. Consequently, there was a lack of ownership by some countries for the planning and financing of eye care services.

Lesson 6 – the transition from elimination of focal eye diseases with regional programmes and international funding, to the development of comprehensive eye care services to achieve Universal Eye Health requires the engagement, commitment and leadership of ministries of health and the willingness of all eye care stakeholders, including the private sector, to support eye care services integrated within national health care plans. In the last decade in order to address Universal Eye Health there has been greater engagement with health ministries. In 2013 the World Health Assembly (WHA) adopted Resolution 66.4 "A Global Action Plan 2014-19 (GAP) for Universal Eye Health", whose vision was a "a world in which no one is needlessly visually impaired, where those with unavoidable vision loss can achieve their full potential and where there is universal access to comprehensive eye care services".[7] The GAP aimed to reduce avoidable visual impairment by 25% in 2019 compared to 2010 estimates. The 2014-19 GAP had three major objectives: (1) generate evidence on the magnitude and causes of VI and use it in advocacy; (2) develop and implement integrated national eye health policies and plans; and (3) ensure multi-sectoral engagement and effective partnerships.

Section 1: The eye, vision impairment and conditions

Supplementary Figure 1: Measuring Visual Function

(a) Testing distance Visual Acuity

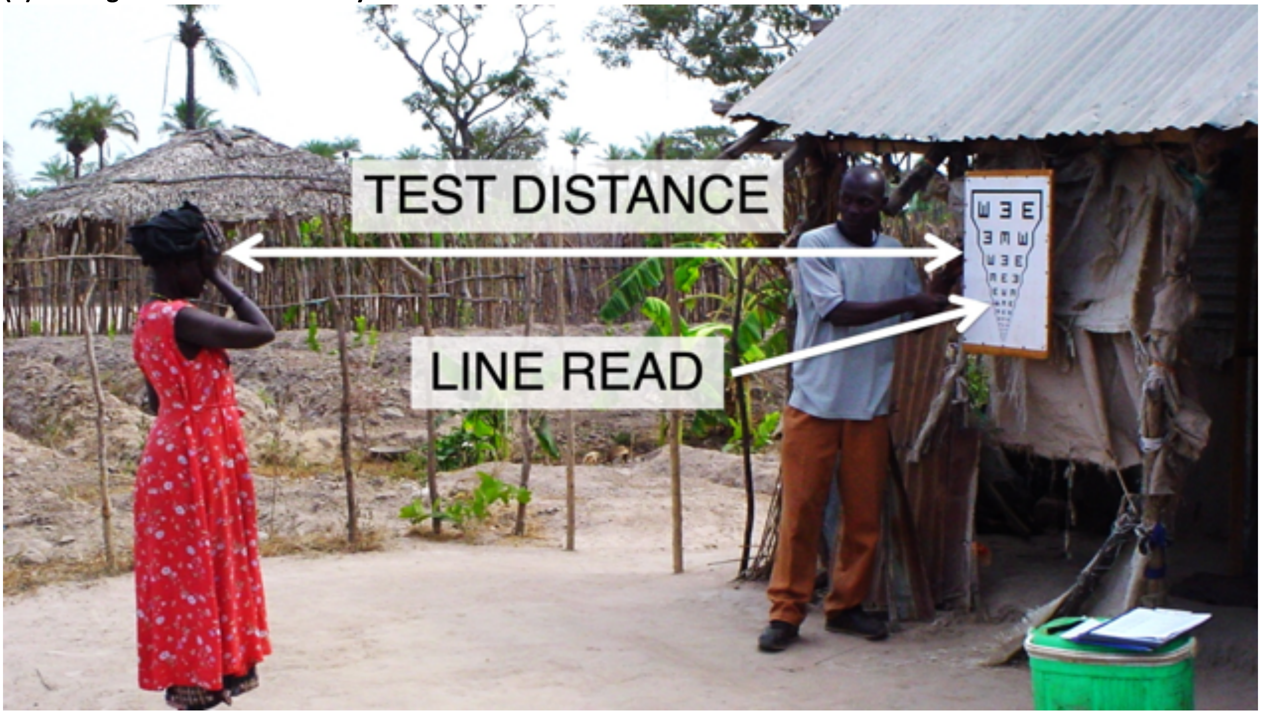

(b) Testing near vision

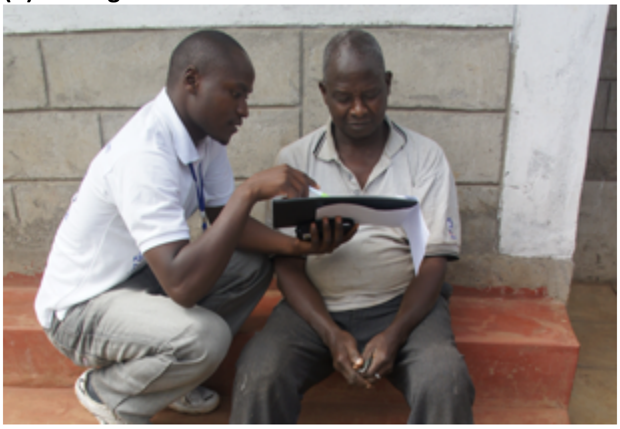

(c) Contrast sensitivity testing

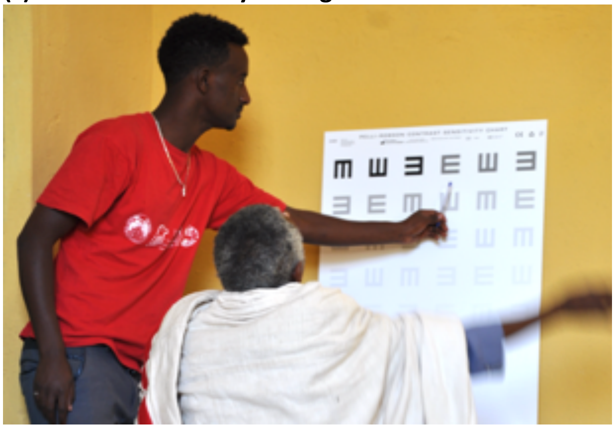

(d) Colour vision testing – Ishihara plate

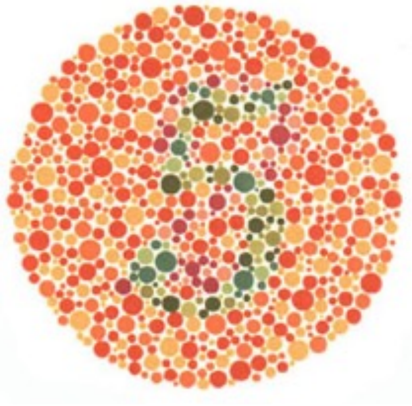

(c) Visual field testing

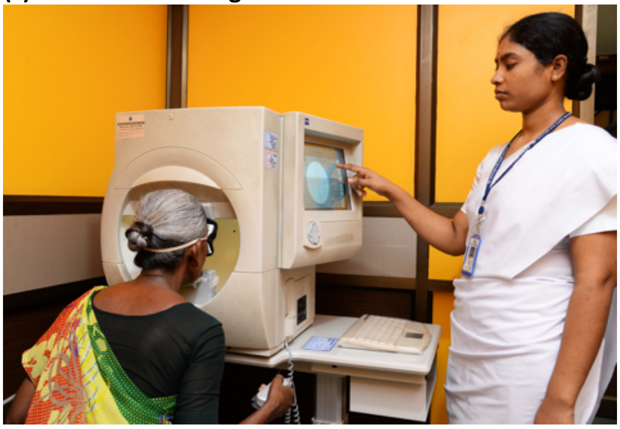

Defining vision impairment

Over recent decades there have been several developments in WHO recommendations on how vision impairment is measured, defined and categorised, particularly for use in population-based epidemiological surveys of vision impairment. The number of sub-categories has increased, there is greater emphasis on standardisation, and changes in the type of VA measured (e.g. corrected and uncorrected acuity).

In 1972, WHO convened a Technical Study Group on The Prevention of Blindness in response to a request for standard definitions of blindness and visual impairment from the WHA.[3] The resulting classification system was based on *"best-corrected visual acuity"* (BCVA). This measure required vision impairment surveys to record the maximum vision achieved by refracting participants. This classification was included in the 9th and 10th revisions of International Classification of Diseases (ICD-9 and ICD-10).

However, using BCVA obscured that, in many settings, people may not own spectacles, and so live with vision impairment from URE. In 2003, WHO convened a consultation to revise these definitions.[4] The group recommended using "presenting vision" instead of BCVA, with epidemiological surveys reporting VA only with spectacles owned by participants, capturing new information on URE. This change, along with additional vision impairment categories, were introduced in a 2010 revision to ICD-10.

In ICD-11 definitions of distance vision impairment were further updated with an additional subdivision of not impaired and mild vision impairment (Commission Report Table 1).[8] WHO has also signalled in the World Report on Vision an intention to change the way vision impairment is primarily reported, moving away from reporting only presenting VA, to also reporting uncorrected VA (without spectacle or contact lenses if worn).[9] Including the measurement of uncorrected acuity allows for better estimation of ongoing service need and the effective coverage of refractive error correction. In addition, ICD-11 recommends measurement of VA for each eye separately and for both eyes together in epidemiological studies, as there is increasing emphasis on the impact on visual function of unilateral (monocular) vision impairment.[10] In prevalence surveys the level of vision impairment is based on VA in the better seeing eye. The Commission uses VA categories defined in Snellen in metres when presenting and discussing data. Moderate and severe vision impairment (MSVI) is defined as VA worse than 6/18 but equal to or better than 3/60.

In addition to defining blindness by VA, blindness is also defined based on constriction of the visual field to within 10° of central fixation from any cause; and cortical blindness, when the person is unable to make sense of visual information due to disease involving the posterior visual pathways and/or the visual cortex. Near vision impairment is present when the near visual acuity is worse than N6, which corresponds approximately to a distance visual acuity of <6/12.

Supplementary Table 1: Common eye conditions causing vision impairment

Condition	Short clinical description	Epidemiology (2020 data)	Treatment / Disease Control
**Cataract**	Lens opacities obstruct or scatter light entering the eye.	Cataract is the leading cause of blindness globally (17.8 million) and the second leading cause of moderate or severe vision impairment (83.2 million). Most cataracts are the result of age-related changes, but cataract is also linked to UV damage, smoking, dehydration crisis, metabolic disturbance such as diabetes, galactosemia, and steroid use. Blindness and vision impairment due to cataract are more common among women than men. Young children may also be affected.	Cataract surgery with intraocular lens (IOL) implantation, usually under local anaesthesia, is a very effective treatment. There are several different types of procedure. Most patients (70%-90%) attain a best-corrected visual acuity of 6/18 or better by 2 months. Cataract surgery is a highly cost-effective intervention.[11] Cataract surgery for young children is more complex and requires expertise and a general anaesthetic
**Refractive error**	Blurred vision because light is not sharply focused on the retina, due to a mismatch between the axial length of the eye and the refractive power of the cornea and/or lens. Hypermetropia (long sight) is difficulty seeing near objects. Astigmatism arises from irregular corneal curvature and affects vision at all distances. Myopia (short sight) is difficulty seeing distant objects. High myopia can be accompanied by degenerative changes in the retina (atrophy, holes, detachment) and sclera (posterior staphyloma).[12]	Uncorrected refractive error is the second leading cause of blindness globally (3.7 million) and the first leading cause of moderate or severe vision impairment (157.5 million). Myopia is the most common form, usually developing in childhood and adolescence. Globally there is an ongoing major increase in the prevalence of myopia and high myopia. This is most marked in the Asia-Pacific region.[13] In Asian children this may be attributable to intense schooling practices.	Refractive error can be corrected using spectacles, contact lenses, IOL implantation during cataract surgery or laser refractive surgery. Spectacles can be provided for as little as US$ 5 per pair.[14]<br><br>Interventions proven to reduce incidence and/or progression of myopia include low-dose atropine, certain designs of spectacles and contact lenses reducing peripheral optical defocus, orthokeratology and increasing time outdoors.[15,16]
**Presbyopia**	Loss of accommodation (the ability to change focus from far to near) as a natural part of ageing results in a reduced unaided ability to see near objects, as in reading, using mobile phones, threading needles or recognising money.	The condition begins at around age 40 and becomes essentially universal by the late 50s.[17] Thus, the prevalence peaks during the most productive working years when it is shown to hamper economic productivity.[18] Uncorrected presbyopia affects 507.4 million people.	Near-vision / reading glasses. Spectacles can be provided for as little as US$ 1 per pair. There is trial evidence that their use significantly boosts economic activity.[18]
**Glaucoma**	Progressive damage of the optic nerve, usually of both eyes, accompanied by visual field loss which can lead to total, irreversible blindness. Higher intraocular pressure is a risk factor. There are several underlying pathologic mechanisms, most frequently classified as primary or secondary, open or closed anterior chamber angle. Most glaucoma starts in adult life, but young children and infants can also be affected. Glaucoma also affects function in ways that are not captured by VA-based measures of visual impairment	The glaucomas are the third leading cause of blindness globally (3.6 million) and the fourth leading cause of moderate or severe vision impairment (4.1 million). The risk of developing glaucoma increases with age and is increasing globally with population ageing. The global prevalence of glaucoma in people aged 40 years and above is 3.54%.[19] Primary open-angle glaucoma (POAG) is more common than primary angle-closure glaucoma (PACG) in most populations. Africa has the highest prevalence of POAG (4.2%) while Asia has the highest prevalence of PACG (1.09%).	Detection and treatment are challenging, although artificial intelligence has tremendous potential to improve diagnosis. It is uncertain whether population-based screening of glaucoma is cost-effective. In high income countries it is not, but, in India and China it may be.[20,21] Treatment aims to reduce the intraocular pressure through: topical medication, laser (trabeculoplasty, iridotomy),or surgery (trabeculectomy, aqueous drainage shunts).
**Age-related macular degeneration (AMD)**	Degeneration of the central part of the retina (macula) which is responsible for clear central vision. AMD has "dry" and "wet" forms. The latter is more likely to damage vision due to abnormal, blood vessels that	AMD is the fourth leading cause of blindness globally (1.9 million) and the third leading cause of moderate or severe vision impairment (6.2 million). AMD is the leading cause of vision loss in high-income countries. It	Wet AMD can be effectively controlled by repeated, long term anti-VEGF intravitreal injections, which have the potential to stabilise vision.[22] Extensive research is being undertaken to develop additional therapies.[23,24]

	develop under the retina, which can leak fluid and bleed. However, atrophy of the macula in dry AMD may also cause significant vision loss.	has a complex polygenetic basis which is influenced by other factors (smoking, hypertension, hypercholesterolaemia). Dry AMD is more common than wet AMD. Most vision loss occurs in those above the age of 70 years.	Oral anti-oxidant vitamins, and smoking cessation may reduce the risk of AMD progression.
**Diabetic retinopathy (DR)**	Damage to the small blood vessels in the retina leads to leakage of plasma fluid and blood, which may damage central vision ("diabetic macular oedema, DMO"). Retinal ischemia from blocked vessels can lead to abnormal, fragile new vessel growth on the retina ("proliferative diabetic retinopathy PDR"). These can bleed and the traction this creates may detach the retina. DMO is a more frequent cause of vision loss than PDR.	Diabetic retinal disease (DMO and PDR; DR) is the fifth leading cause of blindness globally (1.1 million) and the fifth leading cause of moderate or severe vision impairment (3.8 million). It is the only leading condition that is currently experiencing an increase in age-standardised prevalence. About one third of people with diabetes mellitus have DR, which is the leading cause of blindness globally among people of working age. Blindness and vision impairment due to DR are more common among women than among men. The risk of DR among people with diabetes increases the longer they have had diabetes, and by poor diabetes control, hypertension and hypercholesterolaemia. Marked increases in DR are being reported as the prevalence of diabetes rises in all world regions.	General medical care with self-management should seek to optimise control of blood sugar, blood pressure and cholesterol. Treatment of DR depends on the type and severity, options include: retinal laser; intravitreal injection of anti-VEGF or steroid; retinal surgery. Timely treatment has been proven to reduce the risk of vision loss.[25] Thus, DR screening programmes (using retinal photography) are becoming more widely adopted globally, and are proven to significantly reduce blindness by promoting early treatment.[26]
**Trachoma**	Caused by repeated infection of the ocular surface with the bacterium *Chlamydia trachomatis*.[27] Chronic conjunctival inflammation (active trachoma), characterised by lymphoid follicles results in conjunctival scarring and inward-turning of the eyelids (entropion) and eyelashes (trichiasis). Vision loss results from corneal scarring.	In May 2020, trachoma was known to be a public health problem in 45 countries, mostly in sub-Saharan Africa. Around 1.9 million people were estimated to be blind/vision impaired from trachoma and 137 million lived in areas requiring programmes to control active trachoma.[28] The transmission of *Chlamydia trachomatis* is thought to be through direct person-to-person contact, on fomites or by eye-seeking flies.[27] Trachoma flourishes in environments with limited access to water and sanitation, and clusters in poorer communities. Children are the major reservoir of infection (though they themselves are not usually visually-affected), and those who care for them (usually women) are at greatest risk for visually-significant disease.	Trachoma elimination programmes use the SAFE strategy.[27] Eyelid surgery ("S") can correct entropion and trichiasis. Mass antibiotic treatment ("A"), usually with oral azithromycin, is given to whole districts once a year. Facial cleanliness ("F") and environmental improvement ("E"), particularly increased access to water and sanitation, are promoted to suppress transmission. As of June 2020, nine countries had been validated as having eliminated trachoma as a public health problem using this strategy and more were poised to do so soon. Activities leading to trachoma elimination are coordinated internationally within the WHO Alliance for the Global Elimination of Trachoma by 2020 (GET2020).
**Onchocerciasis**	Onchocerciasis, known as "river blindness," is caused by infection with *Onchocerca volvulus,* transmitted by the blackfly *Simulium damnosum.* The microfilaria cause an inflammatory reaction in the eye leading to damage to several structures (corneal scars, uveitis, chorioretinal atrophy, optic neuritis and optic atrophy). Skin manifestations are common and can be disfiguring and cause pronounced and prolonged itching.	Onchocerciasis is endemic in 34 countries, mostly in sub-Saharan Africa. Around 21 million people are infected individuals globally and more than 1 million people with vision impairment.[29] The blackfly vector lives and breeds near fast flowing water, and so communities living in these fertile areas are at greatest risk of infection. In hyper-endemic zones, a substantial proportion of adults may be visually impaired.	Disease control involves community-directed mass drug administration of ivermectin. Ivermectin kills microfilariae, and stops adult worms from producing more microfilaria for a few months following treatment. Control of blackfly has been achieved in some countries by spraying rivers with larvicides in coordinated, multi-national programmes. By October

			2018 four countries in Latin America had been verified as having eliminated onchocerciasis.
**Microbial keratitis**	The cornea can be infected by bacteria, fungi, viruses (such as herpes simplex virus) and protozoa (such as acanthamoeba). Corneal ulcers are characterised by pain, reduced vision, and signs of acute inflammation. On resolution, the cornea may be left with visually disabling or cosmetically-significant scarring. Delayed or inadequate treatment may lead to the loss of the infected eye.	Major risk factors for bacterial and fungal infections are contact lens use, minor abrasions to the cornea surface and pre-existing ocular surface disease. The use of traditional eye medicine may also exacerbate the problem. Bacterial infections predominate in temperate regions and fungal infections are more common in tropical regions. The annual global incidence of fungal keratitis exceeds 1 million.[30] Acanthamoeba is acquired through exposure to contaminated water or contact lens solutions. Agricultural workers and those living in rural areas are at particular risk of trauma may have poor access to care.	The risk of corneal infection is reduced by careful contact lens hygiene and the use of protective eyewear in the workplace. Early diagnosis and application of prophylactic antibiotics to corneal abrasions reduces risk of progression to microbial keratitis.[31] Treatment of established infections requires urgent, intense topical therapy (lasting days to many weeks) with an appropriate antimicrobial, ideally informed by microbiological results.
**Trauma**	Injury to the eye and adjacent structures can occur due to penetrating injury, mechanical force, or exposure to chemicals, heat, or radiation.	Peak ages: 4-7 years, mid-20s and among older people. Males more than females. Most children are injured during play; most adult injuries are related to work, sport, violence or motor vehicle collisions. Blindness is often monocular.	Prevention: protective eye wear, seat belts. Treatment of more severe eye injuries is complex and may require surgery with varying visual outcomes. Visual rehabilitation often requires long term treatment, which should include protective lenses to reduce risk of damage to the healthy eye.
**Corneal ectasia**	Conditions include degenerative ectasia (keratoconus), dystrophies and inflammatory conditions. Keratoconus is the most frequent; the cornea bulges outwards, becoming very thin.	Keratoconus usually becomes apparent around puberty with progressive myopia and irregular astigmatism. Prevalence varies globally, being most common in the middle east. It may be associated with allergic eye disease and other systemic conditions.	Progressive keratoconus can be effectively halted by corneal collagen cross-linking. Vision can be improved by correction of refractive error, usually with contact lenses. Corneal transplantation is sometimes needed.
**Corneal scarring in children from vitamin A deficiency (VAD) and Measles**	Vitamin A maintains ocular surface integrity. Acute VAD in young children can lead to corneal melting ("keratomalacia") which is associated with high child mortality. Corneal melting and corneal ulcers usually heal to leave scarring and loss of vision. Measles infection can exacerbate or cause VAD.	Young children (<5 years) and pregnant women living in poor communities are at greatest risk of VAD.[32] In 2005 there were an estimated 5 million young children and 10 million pregnant women affected by VAD. The incidence of corneal scarring and death from VAD is declining globally as a result of control measures.	VAD can be prevented through diets rich in yellow and orange fruits and vegetables, dark green leafy vegetables, eggs and milk. Regular vitamin A supplementation of preschool age children and measles vaccination have reduced corneal blindness in most affected countries. Children with measles and the ocular signs of VAD should be given three doses of high dose vitamin A.
**Retinopathy of prematurity (ROP)**	This disease of premature new-borns is caused by a failure of normal retinal vascular development. Subsequent retinal neovascularisation, and detachment can develop, leading to total, irreversible blindness during infancy.	ROP is among the leading causes of blindness in children globally.[33] Risk factors for ROP include prematurity, low birth weight, poorly-controlled oxygen therapy and sepsis. Blindness from ROP is increasing in regions where neonatal services are being expanded. For 2010 it was estimated 184,000 babies developed ROP of which 32,200 became blind or severely visually impaired.[34]	Risk is reduced by good ante-natal care, maternal steroids in threatened preterm labour, and meticulous neonatal care with tight control of oxygen therapy from immediately after birth.[35] Preterm babies are regularly screened for signs of ROP, which requires significant technical capacity. Treatment options include retinal laser, anti-VEGF intravitreal injections and surgery for retinal detachment.

**Amblyopia**	Amblyopia is a vision development disorder in which the visual pathways fail to fully mature due to impaired visual input in early childhood. It may affect one or, less commonly, both eyes.	Amblyopia can arise for many reasons, including strabismus (squint), RE and ocular media opacities (cataract). It currently affects around 100 million people globally.[36]	Early detection and treatment of the primary cause is important where possible, for example cataract surgery. Assess and address refractive error. Amblyopia treatment with patching may be needed.
**Cerebral visual impairment (CVI)**	CVI arises from damage to the visual pathways within the brain. The eyes and anterior visual pathways appear relatively normal, but pallor of the optic discs usually develop. Affected children may present with visual inattentiveness and they may have other neurological problems such as cerebral palsy.	CVI is the most frequent cause of childhood visual impairment in high income countries. It has many causes including conditions which interfere with the blood supply to the brain, infections, hydrocephalus, trauma, intracranial haemorrhage and prematurity. In low income countries it can be a complication of cerebral malaria and meningitis. In adults, CVI with or without a visual field defect can be the first presentation of a stroke, brain tumour, dementia or other degenerative brain condition.	Early recognition of CVI in someone who may have only mild or moderate impairment of distance visual acuity initially, but profound impairment of visual functioning (eg inability to find their way around safely, or to interpret written information or signs) is important, to enable timely onward referral to a neurologist for diagnosis and appropriate management and support. Comprehensive rehabilitation services should be offered that are matched to the child's needs.
**Uveitis and Scleritis**	Uveitis is a broad grouping of intraocular inflammatory and infectious conditions resulting from inflammation of the uveal tract inside the eye. Scleritis is inflammation of the sclera, the tough white coat of the eyeball. They present with a wide spectrum of symptoms, including pain, redness, sensitivity to light, and loss of vision. In uveitis, examination may reveal inflammatory cells inside the eye and changes to the inner aspect of the cornea, iris, retina, choroid, blood vessels and optic nerve head. Uveitis and scleritis may be classified by cause and location (anterior, intermediate, posterior). Both can lead to secondary cataract and glaucoma.	Both uveitis and scleritis may have non-infectious and infectious causes, although an underlying cause cannot be determined in the majority of cases. They are associated with many non-infectious systemic immune-mediated inflammatory diseases.<br>Infectious causes are more common in LMICs (30-60%); these include toxoplasmosis, herpes viruses, Ebola virus, tuberculosis, syphilis, leprosy, Lyme disease, and HIV-related infections (CMV retinitis).[37] Ocular toxoplasmosis is particularly common in S. America, where it is an important cause of blindness from posterior uveitis in children.	Careful ocular and systemic assessment, including relevant investigations and history-taking, to identify any underlying cause. Where an infectious aetiology is suspected, specific antimicrobial therapy is indicated. Inflammation is controlled with a range of ocular and systemic anti-inflammatory therapies. Uveitis and scleritis may follow a relapsing and remitting or chronic, course requiring ongoing treatment and follow-up.

Section 2: The Importance of Eye Health

Scoping review on Eye health and the Sustainable Development Goals

The manuscript for this review is forthcoming. A methods summary is provided below; the review protocol has been published.[38] The principle findings are outlined in the Commission Report Table 2 and Figure 4.

Summary of methods

**Title:**	**Global Eye Health and the Sustainable Development Goals: Protocol for a Scoping Review**
Objective:	We aimed to answer the following two questions:<br>1. What is the nature and the extent of the published evidence that services improving eye health contribute to advancing specific SDGs?<br>2. What are the main pathways by which such services lead to advancement of the SDGs?
Search date:	31 October 2019
Search databases:	MEDLINE, Embase and Global Health
Key inclusion/ exclusion criteria:	Included:<br>• All primary research studies or meta-analyses were included if they reported the relationship between the delivery of an eye health service that aimed to change eye health and: (1) an outcome related to one of the SDGs, or (2) an element on a pathway between eye health and an SDG.<br>• All time periods, world regions, and languages.<br>• Published peer-reviewed manuscripts only.<br><br>Excluded:<br>• For the purposes of this review, we excluded studies that related to SDG 3.<br>• Studies without a comparison group, or studies that only compared different types of eye treatments against each other (e.g. eye drop A versus eye drop B).<br>• Studies where simulation was used in the exposure group (e.g. using goggles to simulate the effects of an eye condition) or outcome (e.g. virtual reality driving simulators).<br>• Studies with sample sizes less than 100 participants.<br>• Grey literature.
Number of included studies:	29
Protocol registration:	Open Science Framework (https://osf.io/gu4z6/); 15 November 2019
Protocol publication:	Zhang JH, Ramke J, Mwangi N, Furtado J, Yasmin S, Bascaran C, Ogundo C, Jan C, Gordon I, Congdon N, Burton MJ. Global eye health and the sustainable development goals: protocol for a scoping review. *BMJ Open*. 2020;10(3):e035789. Published 2020 Mar 18. http://dx.doi.org/10.1136/bmjopen-2019-035789

Vision impairment and inclusive development

Supplementary Table 2: Educational attainment of children with vision impairment as their main special educational need in England and Wales, 2016

Children with vision impairment as their primary Special Educational Need (SEN) had lower educational scores than pupils in general, and this is consistently the case across different subjects and ages in primary and secondary schooling. A caveat is that approximately one quarter of children who had vision impairment as their primary SEN also had one or more additional SEN (e.g. learning difficulties), and so gaps in educational achievement may not be solely attributable to their vision loss. Source: RNIB. Primary school data from RNIB Briefing Report 2: Attainment of early years and primary school aged children with VI as their primary (main) SEN. Year 11 data from RNIB Briefing Report 3: Attainment at Key Stage 4 (GCSE level).[39]

**Education Level**	**Proportion reaching expected level or above**	
	**Children with VI as their primary SEN**	**All pupils**
	N = 11,592*	N = 1,795,306
Year 1 - Phonic decoding	59%	81%
Year 2 – Reading	60%	98%
Year 2 – Writing	44%	79%
Year 2 – Mathematics	59%	91%
Year 2 – Science**	61%	82%
Year 6 – Reading	62%	85%
Year 6 – Writing	57%	89%
Year 6 – Mathematics	62%	87%
Year 6 – Grammar, punctuation, spelling	62%	94%
Year 11 – 5 or more A*-C grades at GCSE	43%	57%

*3,188 children had a secondary special educational need (SEN)
**Only assessed at expected level – not above

Supplementary Figure 2: International Classification of Functioning, Disability and Health.

Source: WHO. International Classification on Functioning, Disability and Health (ICF).[40]

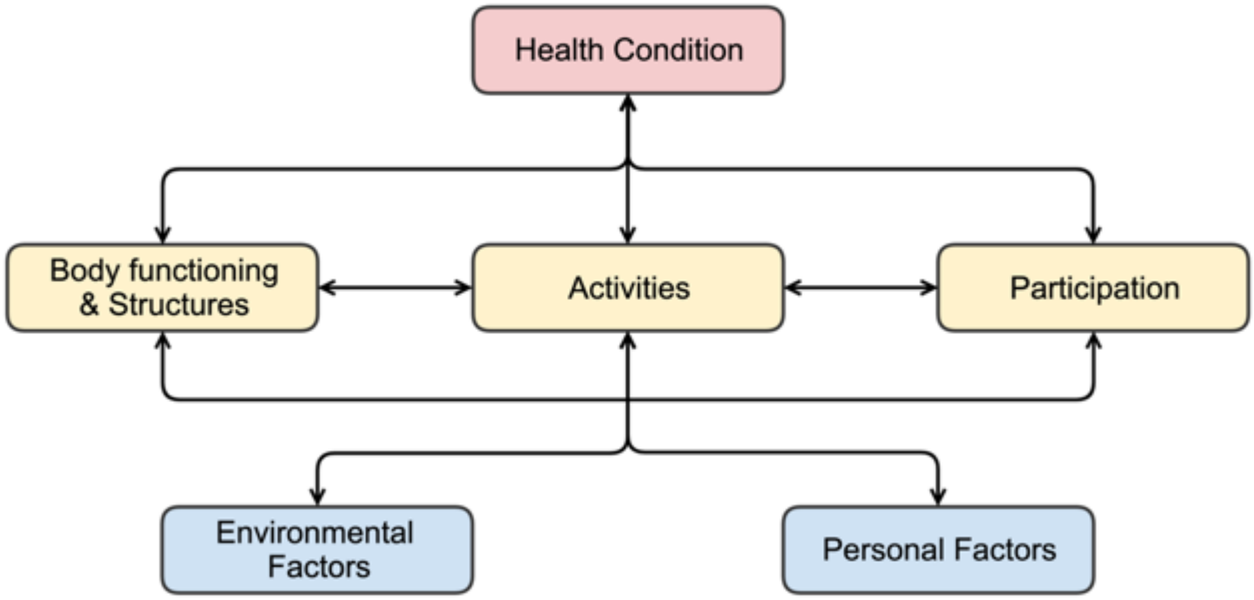

Umbrella review of reviews on Eye Health and Quality of Life

The manuscript for this umbrella review is *In Press:*

Eye Health and Quality of Life: A Global Assessment Through A Systematic Review of Systematic Reviews.
Lama Assi, Fatimah Chamseddine, Perla Ibrahim, Hadi Sabbagh, Lori Rosman, Nathan Congdon, Jennifer Evans, Jacqueline Ramke, Hannah Kuper, Matthew J Burton, Joshua R Ehrlich, Bonnielin K Swenor. *JAMA Ophthalmology 2021, In Press*

A methods summary is provided below; the review protocol has been published.[41] The principal findings are outlined in the Commission Report

Summary of methods

Title:	**Eye Health and Quality of Life: A Global Assessment Through A Systematic Review of Systematic Reviews**
Objective:	We aimed to answer the following two questions:<br>1. What is the association between vision impairment or eye disease and quality of life?<br>2. What is the impact of ophthalmic interventions on quality of life?
Search date:	29 June 2020
Search databases:	Medline Ovid, Embase.com, Cochrane Database of Systematic Reviews, Proquest Dissertations and Theses Global
Key definitions:	Ophthalmic intervention: Any intervention that aims to correct or improve vision, slow down the progression of vision loss, improve functional ability among those with vision loss (e.g., low vision rehabilitation, use of assistive devices), or relieve eye pain or discomfort.<br>Quality of life: Generic or health-related, vision-related, or disease-specific quality of life.<br>Systematic review: A review that includes a research question, a search strategy with the sources searched, inclusion and exclusion criteria, screening methods, a discussion about the quality of included studies and risk of bias, and information about data analysis and synthesis.
Key inclusion/ exclusion criteria:	Included:<br>Systematic reviews and meta-analyses that evaluate the impact of vision impairment, eye disease, or ophthalmic interventions on quality of life;<br>Systematic reviews that reported on quality of life outcomes such as health-related, vision-related, or disease-specific quality of life questionnaires, or qualitative assessment of physical, emotional and social well-being, vision function in day-to-day life.<br>Excluded:<br>Systematic reviews that assessed stroke-related visual impairment and eye diseases or psychologic interventions, such as coping strategies;<br>Systematic reviews that included case series or expert opinion pieces;<br>Systematic reviews in a language other than English.
Number of included studies:	69
Protocol registration:	Open Science Framework (https://osf.io/qhv9g/); 10 February 2020; made "public" on 5 June 2020.
Protocol publication:	Assi L, Rosman L, Chamseddine F, Ibrahim P, Sabbagh H, Congdon N, Evans J, Ramke J, Kuper H, Burton MJ, Ehrlich JR, Swenor BK. Eye Health and Quality of Life: An Umbrella Review Protocol. BMJ Open *In press*

Supplementary Figure 3: Quality of Life Umbrella Review Summary

This figure summarises the findings from the umbrella review assessing the impact on quality of life of ophthalmic interventions, compared to controls or to baseline status (33 interventions). Interventions included: cataract surgery, correction of refractive error, antibiotic use after corneal surgery, anti-VEGF treatment for AMD or DR, trabeculectomy surgery for glaucoma, rehabilitation for untreatable cases of vision impairment, trichiasis surgery in trachoma, uveitis treatment and vision screening. The outer ring represents the overall number of interventional studies identified by eye condition category. In the inner ring, the darker shade represents improvement in quality of life in the group receiving an intervention, compared to baseline or a control group (no intervention, placebo/sham therapy). The inner ring lighter shade represents the lack of an association or no difference in quality of life after an intervention compared to baseline or a control group. Each intervention may be informed by more than one systematic review, and each systematic review may have assessed more than one intervention.

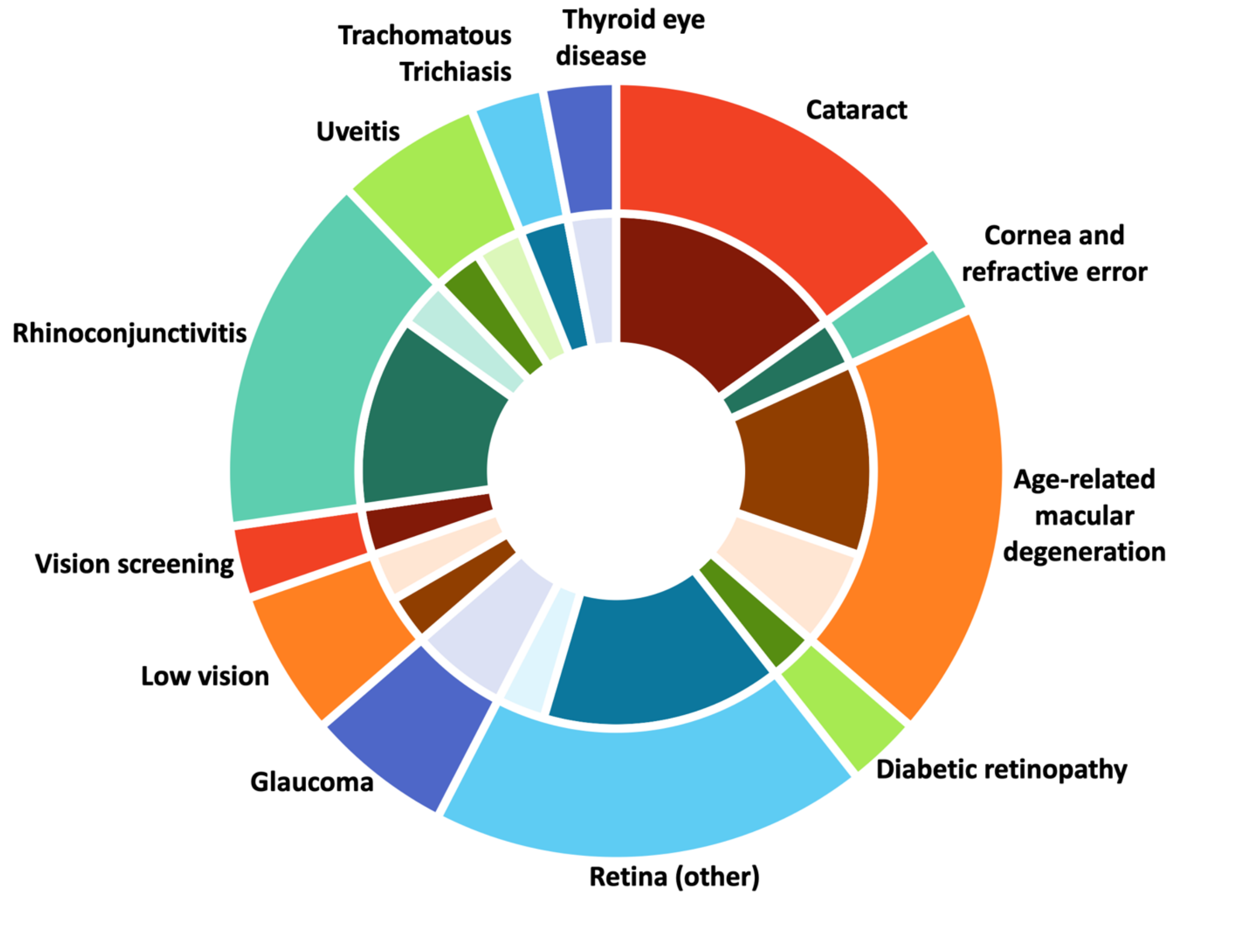

Rapid review on vision impairment, non-communicable diseases and mental health

This review was summarised from the following report:
Keay L, Jalbert I, Ren K, Nguyen H, Vajdic C, Odutola M, Gyawali R, Toomey M, Peters R, Ee N, Dillon L, Hackett M. (2019). An assessment of the intersection of eye health and NCDs – Rapid review of the existing evidence. School of Optometry and Vision Science, The University of New South Wales, Sydney, Australia.

The executive summary of the report is provided below.

This rapid review was convened to investigate the intersection of eye health and common non-communicable diseases (NCDs). A series of pragmatic, rapid reviews were conducted to appraise the available evidence and answer the following key questions:

1. What is the comorbidity prevalence of eye health and NCDs?

This review identified that people with eye health conditions often also have major NCDs:

- Population based data from High Income Countries (HIC) and Middle-Income Countries (MIC) countries find 27% prevalence of cancer, 14% prevalence of Chronic Obstructive Pulmonary Disease (COPD), 39% prevalence of 'heart disease', 25% prevalence of depression in adults with eye diseases and 7% presence of dementia in adults with glaucoma.
- Studies which compare prevalence in adults find that people with eye conditions were more likely than those without (controlled studies) to also report a current or previous diagnosis of the chronic conditions cancer: any cancer (OR 1.2, 95% CI 1.1-1.4), lung cancer (OR 2.1 95% CI 1.1-3.9), cardiovascular disease (OR 1.6, 95% CI 1.4-1.9), depression (OR1.6, 95% CI 1.4-1.8), and dementia (OR 2.0, 95% CI 1.7-2.4).
- Cataract is more common in sun-related skin pathologies (basal cell carcinoma, squamous cell carcinoma and actinic keratosis) possibly due to the common risk factor of sun exposure. Cataract is more prevalent with CVD.
- There is evidence that the comorbid prevalence of NCDs is related to the severity of vision loss. Retinopathy is more prevalent with increasing severity coronary artery disease (CAD). Similarly, depression is more prevalent with more severe vision loss.
- When there are multiple comorbidities, the impact on NCDs like dementia or depression is increased. For example, individuals with dual sensory loss (combined vision and hearing loss) are also have a diagnosis of dementia. Similarly, depression is even more prevalent in those with cancer and vision loss than those with cancer alone.
- More research is needed in Low and Middle-Income Countries (LMIC) to determine the patterns of comorbidities with eye diseases in these settings.

2. What are the common risk factors for eye health conditions and co-morbid NCDs?

- CVD has the most similar profile of risk factors reflecting the fact that many eye diseases involve the retinal microvasculature. Common risk factors include lifestyle factors like tobacco smoking, excessive alcohol consumption and obesity are implicated in both eye diseases and CVD. Consumption of vegetables and fruits, Mediterranean diet and physical activity are protective for eye health conditions and CVD.
- Other NCDs like cancer have less similar profile and the strongest common risk factor is older age for both eye conditions and cancer where 70% of incident cases and 87% of deaths are in people over 50 years of age.
- The lifestyle risk factors above are also common to some cancers. Smoking is a risk factor which is common between COPD and eye conditions though it has a very strong association with COPD (4x increased risk).
- There are some similarities in risk factors between dementia and eye conditions: systemic diseases like diabetes (risk factor for glaucoma and diabetic retinopathy), health indicators like high blood pressure and (limited evidence for risk of age-related macular degeneration (AMD) and diabetic retinopathy) high cholesterol and lifestyle factors including smoking and a protective effect for the Mediterranean diet. However, there were mixed or conflicting support for physical activity as a protective factor for dementia.
- There were some common risk factors for depression and eye conditions, including hypertension, diabetes, obesity and common protective effects including physical activity and diet specifically fish, fruit and vegetables, micronutrients like Vitamin D.
- It is also likely that vision impairment is on the causal pathway for depression as shown by the 1.6x increased risk of depression in adults with vision impairment.

3. What are the evidence gaps?

- More research is needed in LMIC
- Longitudinal studies are needed to establish causality rather than simple associations.
- Social determinants are not always investigated with far stronger focus on biological and environment risk factors

Dementia and vision impairment

This panel drew on the results of a systematic review which is forthcoming, the abstract is below.

Visual Impairment and Cognitive Decline Among Older Adults: A Systematic Review

Niranjani Nagarajan, Lama Assi, Varshini Varadaraj, Mina Motaghi, Yi Sun, Elizabeth Couser, Joshua R. Ehrlich, Heather Whitson, Bonnielin K Swenor

KEY POINTS

Question: Is visual impairment associated with cognitive decline, cognitive impairment, and dementia?

Findings: In this systematic review of 110 strong to moderate quality studies, we found that 91 studies (83%) indicated a significant association between VI and cognitive decline, impairment, and dementia.

Meaning: Existing scientific literature provides consistent evidence for an association of visual impairment with cognitive impairment, cognitive decline, and dementia.

IMPORTANCE

There has been increasing epidemiological research examining the association between visual impairment (VI) and cognitive impairment. Both conditions increase with age and cause substantial morbidity. Poor vision may be a modifiable risk factor for cognitive decline, which makes it critical to clarify the association between these conditions.

OBJECTIVE

The objective of this systematic review is to synthesize the published literature on the association of VI with cognitive decline, cognitive impairment, and dementia, which may inform future research, including efforts to develop interventions, and guide policies that address these important public health issues.

EVIDENCE REVIEW

A literature search was performed in Embase, Medline, and Cochrane library databases and was limited to peer-reviewed journals published in English from inception to March 2020. The review included publications that contained subjective and/or objective measures of vision and cognition, or a diagnosis of visual impairment, cognitive impairment or dementia. Longitudinal or cross-sectional studies with ≥ 100 participants aged >50 years were included. The literature search identified 11,805 relevant articles whose abstracts underwent independent screening by two teams of study authors. Data abstraction was performed by one author. Three reviewers independently assessed study quality using the Effective Public Health Practice Project Quality Assessment Tool (EPHPP)

FINDINGS

After abstract screening, 110 full-text articles were selected for data extraction, of which 53 were cross-sectional, 43 longitudinal, and 14 were case-control studies. The number of participants included in these studies ranged from 112 to 7,210,535. Ninety-one (83%) of these studies reported that VI was associated with cognitive impairment 17 studies received a strong overall quality assessment rating, 12 of which found a positive association between VI and cognitive impairment; 70 studies received an overall moderate rating, 58 of which found a positive association. The remaining 23 studies received a weak rating.

CONCLUSION AND RELEVANCE

Our systematic review indicates that a majority of studies examining the vision-cognition relationship report that VI is associated with cognitive decline, cognitive impairment, and dementia among older adults. This synthesis of evidence supports the need for additional research to understand the mechanisms underlying the association between VI and cognitive impairment and to test interventions that mitigate the cognitive consequences of vision loss.

Scoping review on dual sensory impairment

The manuscript for this review is forthcoming:

Prevalence and impact of combined vision and hearing (dual sensory) impairment: a scoping review

Bright T, Ramke J, Zhang JH, Kitema GF, Mdala S, Safi S, Yoshizaki M, Mactaggart I, Gordon I, Swenor B, Burton MJ, Evans JR.

Summary of methods

**Title:**	**Prevalence and impact of combined vision and hearing (dual sensory) impairment: a scoping review**
Objective:	We aimed to answer the following three questions:<br>1. What is the nature and extent of the evidence on prevalence of DSI globally and across regions?<br>2. What is the nature and extent of the evidence on the impact of DSI on people's lives? (e.g. quality of life, mental health or mortality)<br>3. How was DSI defined in this literature?
Search date:	28 February 2020
Search databases:	MEDLINE, Embase and Global Health
Key inclusion/ exclusion criteria:	Included:<br>• primary research studies (any design) that reported on one or both of the prevalence or impact of DSI<br>• studies conducted in any country, including only human participants of any age<br>• systematic reviews were also included if they reported on the prevalence or impact of DSI<br>Excluded:<br>studies not reported in the English language<br>• editorials, case reports, and comments<br>• no full text available<br>• studies focusing on service provision (e.g. screening techniques) for people with DSI<br>• studies that focus on causes of DSI among a restricted subgroup (e.g. outcomes for pre-term infants) will be excluded, unless they report impact outcomes<br>• studies that report on the prevalence of DSI amongst children from schools for the deaf or blind will be excluded (does not provide population prevalence)
Number of included studies:	151 (137 primary studies and 14 reviews)
Protocol registration:	Open Science Framework (10.17605/OSF.IO/MGYFV); 23 March 2020

Summary of findings

Vision and hearing impairment (dual sensory impairment (DSI)) are prevalent worldwide. Both become increasingly common with age and often occur concurrently.[42] DSI is an important consideration for healthy ageing as global populations grow older.

We identified 137 primary studies, which were mostly population-based and located in HICs (90%). There was striking heterogeneity (67 alternatives) in DSI definitions. A wide range of clinical test methods, thresholds, and self-reported impairment scales were used for both impairments. DSI prevalence was reported for at least 13 different age groups. This makes meta-analyses very difficult and results in a wide prevalence range. For example, in three population-based studies in HICs among people 65 years and older that used clinical assessment tools and similar DSI criteria (vision impairment threshold of <6/18 in better-seeing eye; hearing impairment threshold of >25dB pure tone average in better-hearing ear), prevalence ranged from 3.1% to 24%.[43-45]

Many studies reported DSI impacts people's physical and psychological health and their ability to engage socially and live independent lives. More than half of studies reported consistently worse outcomes for all domains considered, and a further quarter reported a majority of outcomes being worse among the domains considered. Only a few studies (6%) reported no differences in the lived experience of people with DSI.

DSI is relatively common, particularly in older people, and has a major impact on the lives of affected people and is worthy of much more attention. A consensus position on definitions and reporting age groups is needed to enable facilitate estimates to shape services that meet the needs of people with DSI.

Systematic review on driving and vision impairment

A manuscript for this review is forthcoming. A summary of methods is provided below.

Summary of methods

Title:	**Associations between vision impairment and driving and the effectiveness of vision-related interventions: protocol for a systematic review and meta-analysis**
Objective:	The objectives of this systematic review were to:<br>1. Describe the associations between vision impairment and risk of motor vehicle collision involvement across the lifespan<br>2. Evaluate vision-related interventions to reduce motor vehicle collision risk.
Search date:	7 March 2020
Search databases:	MEDLINE, Embase and Global Health
Key definitions:	**Vision-related interventions:** interventions aimed at improving vision.<br>**Vision screening:** these include vision tests, such as visual acuity, visual field, and contrast sensitivity examinations, used to identify potential problems in vision and or eye diseases.<br>**Naturalistic driving:** a research method used to study normal, everyday driving habits by equipping vehicles with small cameras and sensors which continually monitor how the vehicle moves, the driver's behaviour, and the road conditions.
Key inclusion/ exclusion criteria:	Included:<br>• All interventional (randomised controlled trials and quasi-experimental) and observational studies (cohort, cross-sectional, and case-control) studies reported in the English language with full-text available. Systematic reviews were included if meta-analysis was performed. Types of vision-related interventions can include vision screening, refractive correction, cataract surgery or other procedures to restore vision or treat eye diseases.<br>• Studies focused on drivers of all ages using four-wheeled motorised vehicles who have vision impairments caused by either specific eye diseases or specific measures of vision which negatively impact everyday functioning.<br>• Studies reporting on motor vehicle collision involvement, using either state or self-reported data, and other surrogate measures of driving safety including driving errors and performance scores in either on-road driving tests, such as closed-circuit tracks, evaluated by driving instructors, or naturalistic driving with in-vehicle monitoring. Studies which included driving cessation as an outcome measure for driving participation.<br><br>Excluded:<br>• Literature reviews, commentary articles, dissertations, abstracts, editorials and conference presentations.<br>• Studies which use driving simulators or vision impairment simulators.<br>• Studies which report on self-regulatory driving behaviour modifications or self-reported measures of driving confidence.<br>• Studies which do not report on the vision status of its participants or who include drivers with either specific medical conditions (e.g. dementia, stroke) or vision impairments caused by other medical factors (e.g. hemianopia caused by brain damage).
Number of included studies:	115
Protocol registration:	PROSPERO (CRD42020172153 [https://www.crd.york.ac.uk/prospero/display_record.php?ID=CRD42020172153]); 28 April 2020
Protocol publication:	Nguyen H, Di Tanna GL, Coxon K, Brown J, Ren K, Ramke J, Burton MJ, Gordon I, Zhang JZ, Furtado JM, Mdala S, Kitema GF, Keay L. Associations between vison impairment and driving and the effectiveness of vision-related interventions: protocol for a systematic review and meta-analysis. BMJ Open 2020;0;e040881. http://dx.doi.org/10.1136/bmjopen-2020-040881

Road traffic injury is the leading cause of death for children and young adults.[46] SDG3 and SDG 11 have targets to reduce it. Driving is a complex vision-dependent task. Driver licensing systems regulate driving privileges, usually on the basis of VA and visual field; many, although not all jurisdictions have restricted licenses for vision impairment.[47-49] Some jurisdictions have enhanced requirements for older drivers.[47] Having to renew your license in-person when aged 85 years and over reduces crash fatalities (incident RR 0.83 [95%CI 0.72-0.96]).[50]

Deficits in vision have been associated with Motor Vehicle Collisions (MVCs) and unsafe driving practices. Pooled data from four cohorts of older drivers in the US found tests of processing speed and visual attention predicted MVCs.[51] There are less data from LMICs. One study of motorcycle riders in Nigeria found MVCs were associated with visual field defects.[52]

Cataract is associated with increased risk of MVCs (RR 2.46 [95%CI 1.00-6.16]), possibly through reduced contrast sensitivity.[53,54] There is consistent evidence that risk of MVCs is lower following cataract surgery.[55-58] A systematic review and meta-analysis reported an 88% reduction in risk (OR 0.12 [95%CI 0.10-0.16]).[55]

Glaucoma has been associated with MVCs in several studies. In US, the MVC rate was 1.65 times higher for drivers with glaucoma (RR 1.65 [95%CI, 1.20-2.28]).[59] In Japan, a cross-sectional study found the highest crash rate in drivers with severe glaucoma, with 25% involved in MVCs compared to 3.5% for drivers without glaucoma.[60] Several studies of

drivers with glaucoma found at least two times increased risk for MVCs with severe visual field loss.[59,61,62] However, other clinic-based studies of glaucoma patients do not report evidence for increased MVCs, attributing this to self-regulatory driving practices.[63,64]

Pooled analyses from four AMD studies did not demonstrate an association between AMD and increased MVC involvement.[65] There is evidence that drivers with AMD who continue to drive, adapt and restrict their driving.[66] Drivers with neovascular AMD who were treated with anti-VEGF therapy (ranibizumab) reported better driving ability than sham treatment.[67] Treatment with ranibizumab for diabetic macular oedema is also associated with increased driving participation and proportionally more patients meeting vision requirements for driving.[68]

Although MVC involvement is a major concern, driving is also a means to independent mobility and access to education and employment. Young people with amblyopia or unilateral vision impairment are less likely to acquire a drivers' licence.[69] For older drivers, maintenance of good vision is critically important, e.g. through timely access to cataract surgery. A review of drivers on cataract surgery waiting lists in Australia found 31% did not meet vision requirements for driving and 53% believed their cataract had impacted their driving.[70] With the increasing reliance and preference for motor vehicle transport, maintenance of vision for drivers is essential to prevent road traffic injuries and promote independent mobility.

Systematic review of vision impairment and falls

A manuscript for this review is forthcoming. A summary of methods is provided below.

Summary of methods

**Title:**	**Vision impairment as an independent risk factor for falls and vision related interventions to prevent falls: systematic review and meta-analysis**
Objective:	We aimed to answer the following questions:<br>1. To investigate whether visual impairment should be considered as an independent risk factor for falls in people aged over 65 years.<br>2. To examine if visual interventions like expedited cataract surgery, refractive correction or reduced glare, help to reduce the risk of falls in people aged over 65 years.
Search date:	5 January 2020
Search databases:	Medline, Medline Epub ahead of print and in process & other non-indexed, Embase, CINAHL, ProQuest Science and Technology, Web of Science, Scopus
Key definitions:	*Fall*: A fall is defined as an event that results in a person coming to rest inadvertently on the ground or floor or other lower level (WHO).<br>*Vision impairment*: The International Classification of Diseases 11 (2018) classifies vision impairment into two groups, distance and near presenting vision impairment. Distance vision impairment; mild – presenting visual acuity worse than 6/12, moderate – presenting visual acuity worse than 6/18, severe – presenting visual acuity worse than 6/60, blindness – presenting visual acuity worse than 3/60. Near vision impairment; presenting near visual acuity worse than n6 or m.08 with existing correction (WHO).
Key inclusion/ exclusion criteria:	Included:<br>• Full-text available, English language, human studies, population studies, epidemiological studies, intervention studies (both RCTs and non-randomized), longitudinal studies, studies with quantitative methods for data collection and analysis<br>• Primary research studies of any design that reported vision impairment and falls or an intervention related to vision.<br>Excluded:<br>• commentary articles, dissertations, abstracts.
Number of included studies:	129
Protocol registration:	Ashleigh Chandra, Lisa Dillon, Jessie Huang, Iris Gordon, Jacqui Ramke, Matthew Burton, Lisa Keay. Vision impairment and eye-related interventions to reduce falls risk: a systematic review. PROSPERO 2020 CRD42020187617<br>Available from: https://www.crd.york.ac.uk/prospero/display_record.php?ID=CRD42020187617

Globally, one third of people over aged 65 years fall each year and, for adults over 70 years, falls are the leading cause of injury-related death.[29,71] Population ageing and greater mortality risk per fall make this an emerging problem in LMIC and risk mitigation is important.[72]

Falls have multiple causes, including environmental, biological, socio-economic and behavioural factors; however, vision impairment is an independent risk factor.[71] Vision impairment increased risk of falls (OR 1.52, 95% CI 1.29-1.78) and recurrent falls (OR, 1.81, 95% CI 1.58-2.08).[73] Several large longitudinal studies have found increased risk of falls among

people with vision impairment (OR 1.46, 95% CI 1.04-2.04),[74] visual field loss (OR 1.08, 95% CI 1.01-1.10 per 10 points of visual field loss),[75] and peripheral (OR 1.42, 95% CI 1.06-1.91) and central visual field loss (OR 2.4, 95% CI 1.02-5.45).[76] Specific eye diseases (glaucoma, AMD and cataract) have been shown to increase risk of falls.[77-79]

There is consistent evidence that timely access to cataract surgery can reduce falls risk. In one RCT in the UK, for example, people allocated to expedited first eye surgery (4 weeks) had reduced risk of falls (rate ratio (RR) 0.66 [95%CI 0.45-0.96]) and recurrent falls (RR 0.60 [95%CI 0.36-0.98]) compared with people allocated to routine wait (12 months).[80] Hospitalisation data from Australia suggests injurious falls are only reduced after second eye surgery.[81] Data from the US found cataract surgery reduced fracture risk (Incident rate ratio (IRR) 0.84 [95%CI 0.81-0.87])[82] and in Australia reduced falls (IRR 0.67 [95%CI 0.49 to 0.92]).[83]

Falls risk can also be increased by changes in spectacle correction.[83,84]A prospective cohort of adults with cataract found changes in spectacle correction after cataract surgery increased falls risk (IRR 2.17 [95%CI 1.23-3.85]).[83] These findings are consistent with laboratory studies which find changes to spectacle magnification impacts stair negotiation and balance control.[85]

Although falls are multifactorial in aetiology, vision impairment is an independent risk factor. Falls risk assessment tools need to include vision to help identify older people at risk.[86] There is also need for better integration with eye care services as part of falls prevention efforts in view of the strong evidence for maintaining good vision to prevent falls in older age.

Systematic Review on vision impairment and mortality

A methods summary is provided below; the review protocol has been published.[87] The principal findings are outlined in the Commission Report.

The manuscript for this review is forthcoming:
Eye health and the association between visual impairment and mortality: a systematic review and meta-analysis
Ehrlich JR, Ramke J, Macleod D, Swenor BK, Burn H, Lee CN, Waldock W, Zhang JH, Gordon I, Congdon N, Burton MJ, Evans JR../
Lancet Global Health, 2021, In press

Summary of methods

**Title:**	**The Association Between Vision Impairment and Mortality: A Systematic Review and Meta-Analysis**
Objective:	We aimed to answer the following questions:<br>1. What is the extent, strength, and quality of the published evidence that vision impairment is associated with the risk of all-cause mortality?<br>2. To what degree does vision impairment affect the risk of all-cause mortality?<br>3. What are potential causes of variation in the association of vision impairment with mortality (e.g. measurement, study bias, follow-up duration, etc.)?
Search date:	1 February 2020
Search databases:	MEDLINE, Embase and Global Health
Key definitions:	Vision impairment: defined based on objectively measured visual acuity, however thresholds used to classify vision impairment varied widely between studies.
Key inclusion/ exclusion criteria:	Included:<br>• Studies that reported the association between objectively measured visual acuity and all-cause mortality adjusted for age.<br>• Primary retrospective and prospective cohort studies.<br>• Randomized controlled trials that reported the association between vision impairment and mortality independent of the study intervention.<br>Excluded:<br>• Studies that focused only on populations with specific systemic conditions.<br>• Studies in which >50% of the population was under age 40 years.<br>• Studies published in languages other than English.
Number of included studies:	30 cohorts (from 28 publications)
Protocol registration:	Open Science Framework (https://osf.io/weu96). 6 February 2020.
Protocol publication:	Ehrlich JR, Ramke J, Macleod D, Swenor BK, Burn H, Lee CN, Waldock WJ, Zhang JH, Gordon I, Congdon N, Burton M, Evans JR. Association between vision impairment and mortality: protocol for a systematic review and meta-analysis. BMJ Open 2020; 10: e037556. http://dx.doi.org/10.1136/bmjopen-2020-037556

Section 3: Magnitude of Eye Disease

Global magnitude vision impairment in 2020

Supplementary Table 3: Number of people affected and crude and age-standardised prevalence of vision impairment, by Global Burden of Disease Region in 2020

Data source: GBD/VLEG 2020 data.[88] Figures are numbers of people in millions (95% uncertainty interval) or % (95% uncertainty interval).

	**Blind**			**Moderate and severe vision impairment**			**Mild vision impairment**		
	**Number (millions)**	**Crude Prevalence**	**Age-standardised Prevalence**	**Number (millions)**	**Crude Prevalence**	**Age-standardised Prevalence**	**Number (millions)**	**Crude Prevalence**	**Age-standardised Prevalence**
Andean Latin America	0.35 (0.30-0.40)	0.54% (0.47-0.62)	0.60% (0.51-0.68)	2.76 (2.51-3.02)	4.27% (3.88-4.67)	4.56% (4.14-4.99)	2.14 (1.93-2.37)	3.32% (2.99-3.67)	3.52% (3.17-3.91)
Australasia	0.07 (0.06-0.08)	0.23% (0.20-0.27)	0.15% (0.13-0.17)	0.75 (0.68-0.82)	2.55% (2.30-2.79)	2.03% (1.84-2.23)	0.43 (0.38-0.47)	1.45% (1.30-1.61)	1.16% (1.04-1.29)
Caribbean	0.26 (0.22-0.30)	0.55% (0.47-0.63)	0.50% (0.42-0.56)	1.55 (1.41-1.70)	3.29% (2.98-3.61)	3.06% (2.78-3.35)	1.73 (1.55-1.92)	3.66% (3.29-4.06)	3.46% (3.12-3.83)
Central Asia	0.30 (0.26-0.34)	0.32% (0.27-0.36)	0.41% (0.35-0.47)	2.95 (2.66-3.27)	3.12% (2.81-3.46)	3.67% (3.32-4.04)	2.20 (1.99-2.44)	2.33% (2.10-2.59)	2.58% (2.33-2.86)
Central Europe	0.33 (0.28-0.37)	0.29% (0.25-0.33)	0.17% (0.15-0.19)	3.95 (3.49-4.42)	3.47% (3.06-3.88)	2.17% (1.95-2.41)	1.98 (1.77-2.22)	1.74% (1.55-1.95)	1.37% (1.23-1.52)
Central Latin America	1.27 (1.10-1.42)	0.48% (0.42-0.53)	0.51% (0.44-0.57)	9.84 (8.91-10.78)	3.70% (3.35-4.06)	3.84% (3.48-4.20)	9.11 (8.20-10.11)	3.43% (3.09-3.81)	3.57% (3.21-3.96)
Central Sub-Saharan Africa	0.29 (0.25-0.33)	0.22% (0.19-0.25)	0.49% (0.41-0.55)	2.01 (1.81-2.23)	1.52% (1.37-1.69)	2.90% (2.58-3.23)	3.84 (3.43-4.33)	2.91% (2.59-3.28)	3.83% (3.44-4.25)
East Asia	9.09 (7.89-10.34)	0.61% (0.53-0.69)	0.47% (0.41-0.52)	53.90 (47.84-60.37)	3.59% (3.19-4.03)	2.77% (2.49-3.07)	60.15 (53.28-67.25)	4.01% (3.55-4.49)	3.41% (3.06-3.78)
Eastern Europe	0.79 (0.69-0.89)	0.38% (0.33-0.43)	0.24% (0.21-0.27)	11.08 (9.86-12.34)	5.30% (4.72-5.91)	3.64% (3.28-4.01)	5.34 (4.77-5.96)	2.56% (2.29-2.85)	2.04% (1.84-2.27)
Eastern Sub-Saharan Africa	1.97 (1.74-2.20)	0.46% (0.41-0.51)	1.07% (0.92-1.20)	7.01 (6.38-7.67)	1.64% (1.49-1.79)	3.25% (2.94-3.56)	11.33 (10.14-12.79)	2.65% (2.37-2.99)	3.49% (3.16-3.86)
High-income Asia Pacific	0.54 (0.47-0.60)	0.29% (0.25-0.32)	0.14% (0.13-0.16)	5.34 (4.79-5.86)	2.86% (2.57-3.14)	1.77% (1.60-1.94)	9.84 (8.67-10.98)	5.27% (4.64-5.88)	3.34% (3.03-3.71)
High-income North America	0.71 (0.63-0.80)	0.19% (0.17-0.22)	0.12% (0.11-0.14)	7.44 (6.75-8.11)	2.02% (1.83-2.20)	1.59% (1.44-1.75)	5.14 (4.58-5.70)	1.40% (1.24-1.55)	1.13% (1.02-1.26)
North Africa and Middle East	3.09 (2.65-3.52)	0.49% (0.42-0.56)	0.70% (0.59-0.80)	21.84 (19.87-23.90)	3.46% (3.14-3.78)	4.31% (3.91-4.72)	14.43 (13.06-16.04)	2.28% (2.07-2.54)	2.68% (2.42-2.97)
Oceania	0.04 (0.03-0.04)	0.29% (0.25-0.33)	0.55% (0.47-0.63)	0.39 (0.35-0.42)	2.86% (2.59-3.15)	4.93% (4.46-5.40)	0.38 (0.34-0.42)	2.80% (2.51-3.13)	3.65% (3.29-4.05)
South Asia	11.94 (10.36-13.42)	0.65% (0.56-0.73)	0.90% (0.78-1.01)	96.22 (86.37-106.88)	5.23% (4.69-5.80)	6.44% (5.79-7.13)	60.08 (54.12-66.91)	3.26% (2.94-3.63)	3.83% (3.45-4.26)
Southeast Asia	5.95 (5.16-6.68)	0.88% (0.76-0.98)	1.00% (0.87-1.13)	28.77 (26.53-31.09)	4.23% (3.90-4.57)	4.65% (4.30-5.01)	30.51 (27.62-33.79)	4.49% (4.06-4.97)	4.80% (4.35-5.30)
Southern Latin America	0.16 (0.14-0.18)	0.24% (0.20-0.27)	0.19% (0.17-0.22)	2.12 (1.92-2.31)	3.14% (2.85-3.43)	2.81% (2.55-3.07)	1.29 (1.16-1.44)	1.91% (1.72-2.13)	1.75% (1.58-1.95)
Southern Sub-Saharan Africa	0.48 (0.42-0.53)	0.59% (0.51-0.66)	0.82% (0.72-0.92)	1.56 (1.41-1.71)	1.93% (1.75-2.11)	2.39% (2.17-2.62)	2.53 (2.29-2.81)	3.12% (2.82-3.47)	3.58% (3.23-3.96)
Tropical Latin America	1.78 (1.56-1.99)	0.80% (0.70-0.89)	0.74% (0.65-0.83)	10.33 (9.35-11.29)	4.61% (4.17-5.04)	4.36% (3.95-4.76)	8.49 (7.62-9.46)	3.79% (3.40-4.22)	3.64% (3.28-4.03)
Western Europe	1.53 (1.32-1.76)	0.35% (0.30-0.40)	0.18% (0.16-0.20)	15.42 (13.85-16.87)	3.53% (3.17-3.86)	2.39% (2.16-2.62)	10.91 (9.64-12.17)	2.50% (2.21-2.79)	1.73% (1.56-1.91)
Western Sub-Saharan Africa	2.35 (2.07-2.64)	0.50% (0.44-0.56)	1.11% (0.95-1.26)	9.86 (8.94-10.85)	2.08% (1.89-2.29)	4.06% (3.64-4.50)	15.99 (14.36-17.89)	3.38% (3.03-3.78)	4.40% (3.98-4.87)

Supplementary Table 4: Global and regional leading causes of blindness in 2020

Data source GBD/VLEG 2020 data.[88] Figures are numbers of people in millions (95% uncertainty interval) or % (95% uncertainty interval).

REGION	CATARACT		UNCORRECTED REFRACTIVE ERROR		GLAUCOMA		AGE-RELATED MACULAR DEGENERATION		DIABETIC RETINOPATHY		OTHER	
	Number (millions)	Age-standardised Prevalence	Number (millions)	Age-standardised Prevalence	Number (millions)	Age-standardised Prevalence	Number (millions)	Age-standardised Prevalence	Number (millions)	Age-standardised Prevalence	Number (millions)	Age-standardised Prevalence
Andean Latin America	0.13 (0.10-0.15)	0.22% (0.18-0.27)	0.03 (0.02-0.04)	0.05% (0.04-0.06)	0.04 (0.03-0.05)	0.07% (0.05-0.09)	0.01 (0.01-0.02)	0.02% (0.01-0.03)	0.00 (0.00-0.00)	0.00% (0.00-0.00)	0.14 (0.12-0.16)	0.23% (0.20-0.27)
Australasia	0.02 (0.01-0.02)	0.04% (0.03-0.04)	0.00 (0.00-0.00)	0.00% (0.00-0.01)	0.01 (0.01-0.02)	0.03% (0.02-0.03)	0.01 (0.01-0.01)	0.02% (0.01-0.03)	0.00 (0.00-0.00)	0.00% (0.00-0.00)	0.02 (0.02-0.03)	0.06% (0.05-0.07)
Caribbean	0.07 (0.05-0.08)	0.12% (0.10-0.15)	0.01 (0.01-0.01)	0.02% (0.02-0.02)	0.04 (0.03-0.05)	0.07% (0.05-0.09)	0.00 (0.00-0.00)	0.01% (0.00-0.01)	0.01 (0.01-0.02)	0.03% (0.02-0.04)	0.13 (0.11-0.15)	0.25% (0.21-0.29)
Central Asia	0.07 (0.05-0.08)	0.10% (0.08-0.12)	0.01 (0.01-0.01)	0.01% (0.01-0.01)	0.04 (0.03-0.05)	0.06% (0.04-0.07)	0.01 (0.00-0.01)	0.01% (0.01-0.01)	0.00 (0.00-0.00)	0.00% (0.00-0.00)	0.18 (0.15-0.21)	0.23% (0.20-0.27)
Central Europe	0.05 (0.04-0.07)	0.02% (0.02-0.03)	0.00 (0.00-0.00)	0.00% (0.00-0.00)	0.04 (0.03-0.05)	0.02% (0.01-0.02)	0.03 (0.02-0.04)	0.01% (0.01-0.02)	0.01 (0.01-0.01)	0.01% (0.00-0.01)	0.19 (0.16-0.22)	0.11% (0.09-0.12)
Central Latin America	0.36 (0.29-0.43)	0.15% (0.12-0.18)	0.09 (0.08-0.11)	0.04% (0.03-0.04)	0.11 (0.08-0.13)	0.05% (0.03-0.06)	0.03 (0.02-0.04)	0.01% (0.01-0.02)	0.09 (0.07-0.13)	0.04% (0.03-0.05)	0.58 (0.49-0.66)	0.23% (0.19-0.26)
Central Sub-Saharan Africa	0.04 (0.03-0.05)	0.08% (0.06-0.10)	0.00 (0.00-0.01)	0.01% (0.00-0.01)	0.02 (0.02-0.03)	0.07% (0.05-0.09)	0.00 (0.00-0.00)	0.01% (0.00-0.01)	0.00 (0.00-0.00)	0.00% (0.00-0.00)	0.22 (0.19-0.25)	0.32% (0.27-0.37)
East Asia	3.10 (2.59-3.71)	0.15% (0.13-0.18)	1.19 (0.97-1.39)	0.06% (0.05-0.07)	0.52 (0.40-0.65)	0.03% (0.02-0.03)	0.33 (0.23-0.46)	0.02% (0.01-0.02)	0.24 (0.16-0.35)	0.01% (0.01-0.02)	3.71 (3.22-4.21)	0.20% (0.18-0.23)
Eastern Europe	0.17 (0.14-0.21)	0.05% (0.04-0.06)	0.02 (0.01-0.02)	0.01% (0.00-0.01)	0.11 (0.08-0.13)	0.03% (0.02-0.04)	0.03 (0.02-0.04)	0.01% (0.01-0.01)	0.00 (0.00-0.00)	0.00% (0.00-0.00)	0.47 (0.41-0.53)	0.15% (0.13-0.17)
Eastern Sub-Saharan Africa	0.72 (0.61-0.82)	0.45% (0.38-0.52)	0.11 (0.09-0.13)	0.04% (0.03-0.05)	0.20 (0.16-0.25)	0.16% (0.13-0.20)	0.07 (0.05-0.09)	0.05% (0.04-0.07)	0.03 (0.02-0.04)	0.01% (0.01-0.02)	0.85 (0.74-0.97)	0.35% (0.30-0.41)
High-income Asia Pacific	0.10 (0.08-0.12)	0.02% (0.02-0.03)	0.02 (0.01-0.02)	0.01% (0.01-0.01)	0.15 (0.12-0.18)	0.03% (0.02-0.03)	0.05 (0.03-0.06)	0.01% (0.01-0.01)	0.03 (0.02-0.04)	0.01% (0.01-0.01)	0.19 (0.17-0.22)	0.07% (0.06-0.08)
High-income North America	0.16 (0.13-0.19)	0.02% (0.02-0.03)	0.02 (0.01-0.02)	0.00% (0.00-0.01)	0.15 (0.12-0.19)	0.02% (0.02-0.03)	0.08 (0.06-0.11)	0.01% (0.01-0.02)	0.06 (0.04-0.08)	0.01% (0.01-0.01)	0.24 (0.21-0.27)	0.05% (0.04-0.06)
North Africa and Middle East	0.84 (0.68-1.04)	0.21% (0.17-0.26)	0.19 (0.16-0.23)	0.03% (0.03-0.04)	0.46 (0.35-0.58)	0.12% (0.10-0.16)	0.20 (0.14-0.26)	0.05% (0.03-0.07)	0.07 (0.05-0.11)	0.02% (0.01-0.02)	1.33 (1.14-1.54)	0.26% (0.23-0.31)
Oceania	0.02 (0.02-0.02)	0.31% (0.26-0.37)	0.00 (0.00-0.00)	0.01% (0.01-0.01)	0.00 (0.00-0.00)	0.06% (0.04-0.07)	0.00 (0.00-0.00)	0.01% (0.01-0.02)	0.00 (0.00-0.00)	0.03% (0.02-0.05)	0.01 (0.01-0.02)	0.13% (0.11-0.15)
South Asia	6.35 (5.37-7.42)	0.51% (0.44-0.59)	1.52 (1.26-1.77)	0.10% (0.08-0.12)	0.58 (0.44-0.73)	0.05% (0.04-0.06)	0.30 (0.20-0.42)	0.02% (0.02-0.03)	0.20 (0.14-0.29)	0.01% (0.01-0.02)	3.00 (2.57-3.45)	0.20% (0.17-0.23)
Southeast Asia	3.18 (2.69-3.69)	0.56% (0.47-0.64)	0.22 (0.18-0.26)	0.03% (0.03-0.04)	0.24 (0.18-0.30)	0.04% (0.03-0.06)	0.16 (0.11-0.22)	0.03% (0.02-0.04)	0.09 (0.06-0.12)	0.01% (0.01-0.02)	2.07 (1.80-2.36)	0.33% (0.29-0.37)
Southern Latin America	0.05 (0.04-0.06)	0.06% (0.05-0.07)	0.01 (0.00-0.01)	0.01% (0.01-0.01)	0.03 (0.02-0.03)	0.03% (0.02-0.04)	0.01 (0.01-0.01)	0.01% (0.01-0.02)	0.01 (0.01-0.01)	0.01% (0.01-0.01)	0.06 (0.05-0.07)	0.08% (0.07-0.09)
Southern Sub-Saharan Africa	0.18 (0.15-0.22)	0.34% (0.29-0.40)	0.02 (0.01-0.02)	0.02% (0.02-0.03)	0.05 (0.04-0.07)	0.11% (0.08-0.13)	0.01 (0.00-0.01)	0.01% (0.01-0.01)	0.01 (0.01-0.02)	0.02% (0.01-0.03)	0.21 (0.18-0.23)	0.32% (0.28-0.37)
Tropical Latin America	0.55 (0.46-0.66)	0.23% (0.19-0.27)	0.08 (0.07-0.10)	0.03% (0.03-0.04)	0.15 (0.12-0.19)	0.06% (0.05-0.08)	0.02 (0.02-0.03)	0.01% (0.01-0.01)	0.14 (0.10-0.20)	0.06% (0.04-0.08)	0.83 (0.71-0.94)	0.35% (0.30-0.39)
Western Europe	0.18 (0.14-0.22)	0.02% (0.02-0.02)	0.04 (0.03-0.05)	0.01% (0.01-0.01)	0.44 (0.34-0.55)	0.04% (0.03-0.05)	0.45 (0.34-0.57)	0.04% (0.03-0.05)	0.07 (0.05-0.09)	0.01% (0.01-0.01)	0.36 (0.31-0.42)	0.06% (0.05-0.07)
Western Sub-Saharan Africa	0.67 (0.56-0.78)	0.38% (0.32-0.45)	0.13 (0.10-0.15)	0.05% (0.04-0.06)	0.23 (0.18-0.29)	0.16% (0.13-0.20)	0.05 (0.04-0.07)	0.03% (0.02-0.05)	0.01 (0.01-0.02)	0.01% (0.00-0.01)	1.26 (1.10-1.42)	0.48% (0.41-0.55)
Global	17.01 (14.40-19.93)	0.21% (0.17-0.24)	3.70 (3.10-4.29)	0.04% (0.04-0.05)	3.61 (2.81-4.42)	0.04% (0.03-0.05)	1.85 (1.35-2.43)	0.02% (0.02-0.03)	1.07 (0.76-1.51)	0.01% (0.01-0.02)	16.04 (14.00-18.06)	0.20% (0.17-0.22)

Supplementary Table 5: Global and regional leading causes of moderate and severe vision impairment in 2020

Data source GBD/VLEG 2020 data.[88] Figures are numbers of people in millions (95% uncertainty interval) or % (95% uncertainty interval).

REGION	CATARACT		UNCORRECTED REFRACTIVE ERROR		GLAUCOMA		AGE-RELATED MACULAR DEGENERATION		DIABETIC RETINOPATHY		OTHER	
	Number (millions)	Age-standardised Prevalence	Number (millions)	Age-standardised Prevalence	Number (millions)	Age-standardised Prevalence	Number (millions)	Age-standardised Prevalence	Number (millions)	Age-standardised Prevalence	Number (millions)	Age-standardised Prevalence
Andean Latin America	0.70 (0.60-0.81)	1.23% (1.04-1.42)	1.61 (1.43-1.80)	2.58% (2.29-2.88)	0.04 (0.03-0.05)	0.07% (0.05-0.09)	0.05 (0.04-0.06)	0.09% (0.07-0.11)	0.02 (0.01-0.02)	0.03% (0.02-0.04)	0.33 (0.28-0.39)	0.55% (0.46-0.66)
Australasia	0.17 (0.15-0.21)	0.32% (0.27-0.38)	0.43 (0.39-0.48)	1.38% (1.23-1.55)	0.01 (0.01-0.02)	0.03% (0.02-0.03)	0.01 (0.01-0.01)	0.02% (0.01-0.02)	0.01 (0.01-0.01)	0.02% (0.02-0.03)	0.11 (0.09-0.12)	0.26% (0.22-0.30)
Caribbean	0.26 (0.22-0.32)	0.50% (0.41-0.59)	0.81 (0.71-0.91)	1.65% (1.45-1.84)	0.03 (0.02-0.04)	0.06% (0.05-0.08)	0.01 (0.01-0.01)	0.02% (0.01-0.02)	0.02 (0.01-0.02)	0.03% (0.02-0.05)	0.42 (0.35-0.49)	0.80% (0.68-0.94)
Central Asia	0.72 (0.60-0.84)	1.06% (0.88-1.24)	1.55 (1.37-1.74)	1.73% (1.53-1.94)	0.03 (0.02-0.04)	0.05% (0.04-0.06)	0.04 (0.03-0.06)	0.06% (0.05-0.08)	0.03 (0.02-0.04)	0.04% (0.03-0.05)	0.59 (0.49-0.71)	0.74% (0.61-0.89)
Central Europe	0.83 (0.68-0.99)	0.37% (0.30-0.44)	2.12 (1.86-2.41)	1.32% (1.17-1.48)	0.05 (0.04-0.07)	0.02% (0.02-0.03)	0.12 (0.09-0.14)	0.05% (0.04-0.06)	0.05 (0.03-0.07)	0.02% (0.02-0.03)	0.78 (0.65-0.93)	0.39% (0.33-0.46)
Central Latin America	1.91 (1.61-2.22)	0.79% (0.67-0.92)	5.71 (5.03-6.37)	2.16% (1.91-2.41)	0.19 (0.15-0.24)	0.08% (0.06-0.10)	0.10 (0.08-0.12)	0.04% (0.03-0.05)	0.20 (0.15-0.27)	0.08% (0.06-0.11)	1.74 (1.50-2.00)	0.69% (0.59-0.79)
Central Sub-Saharan Africa	0.13 (0.11-0.16)	0.33% (0.27-0.40)	1.21 (1.06-1.36)	1.55% (1.37-1.76)	0.03 (0.02-0.04)	0.08% (0.06-0.10)	0.01 (0.01-0.01)	0.02% (0.02-0.03)	0.01 (0.01-0.01)	0.02% (0.01-0.03)	0.62 (0.55-0.71)	0.89% (0.78-1.02)
East Asia	16.35 (13.67-19.28)	0.81% (0.68-0.94)	27.71 (24.47-31.41)	1.47% (1.31-1.65)	0.92 (0.72-1.15)	0.04% (0.03-0.06)	2.43 (1.94-2.98)	0.11% (0.09-0.14)	0.91 (0.66-1.23)	0.04% (0.03-0.06)	5.58 (4.88-6.40)	0.29% (0.26-0.33)
Eastern Europe	1.63 (1.33-1.93)	0.46% (0.38-0.54)	5.99 (5.32-6.78)	2.18% (1.94-2.43)	0.13 (0.10-0.16)	0.04% (0.03-0.05)	0.07 (0.05-0.08)	0.02% (0.01-0.02)	0.07 (0.05-0.09)	0.02% (0.01-0.03)	3.20 (2.75-3.71)	0.93% (0.81-1.07)
Eastern Sub-Saharan Africa	1.55 (1.33-1.77)	1.04% (0.90-1.19)	3.09 (2.71-3.50)	1.03% (0.91-1.15)	0.12 (0.09-0.15)	0.09% (0.07-0.11)	0.10 (0.08-0.13)	0.07% (0.06-0.09)	0.06 (0.05-0.08)	0.04% (0.03-0.05)	2.09 (1.82-2.37)	0.98% (0.85-1.13)
High-income Asia Pacific	1.51 (1.28-1.76)	0.29% (0.24-0.33)	2.75 (2.45-3.05)	1.19% (1.05-1.34)	0.14 (0.11-0.18)	0.03% (0.02-0.03)	0.06 (0.05-0.08)	0.01% (0.01-0.01)	0.07 (0.05-0.10)	0.02% (0.01-0.02)	0.81 (0.70-0.94)	0.24% (0.21-0.27)
High-income North America	1.76 (1.50-2.04)	0.26% (0.22-0.30)	4.14 (3.65-4.61)	1.04% (0.91-1.18)	0.14 (0.11-0.18)	0.02% (0.02-0.03)	0.10 (0.08-0.13)	0.02% (0.01-0.02)	0.15 (0.11-0.20)	0.02% (0.02-0.03)	1.14 (1.00-1.30)	0.23% (0.20-0.26)
North Africa and Middle East	5.40 (4.57-6.30)	1.33% (1.13-1.55)	12.83 (11.40-14.35)	2.24% (2.00-2.50)	0.33 (0.25-0.42)	0.08% (0.06-0.11)	0.49 (0.39-0.61)	0.12% (0.10-0.15)	0.46 (0.34-0.61)	0.10% (0.07-0.13)	2.32 (2.02-2.67)	0.44% (0.38-0.50)
Oceania	0.12 (0.10-0.13)	2.15% (1.86-2.46)	0.24 (0.22-0.27)	2.48% (2.20-2.80)	0.00 (0.00-0.00)	0.04% (0.03-0.05)	0.00 (0.00-0.00)	0.02% (0.02-0.03)	0.00 (0.00-0.00)	0.05% (0.03-0.06)	0.02 (0.02-0.03)	0.19% (0.16-0.23)
South Asia	28.74 (24.67-33.51)	2.15% (1.85-2.49)	53.88 (47.76-60.92)	3.37% (2.99-3.81)	0.96 (0.75-1.20)	0.07% (0.06-0.09)	1.22 (0.97-1.51)	0.09% (0.07-0.11)	0.44 (0.32-0.60)	0.03% (0.02-0.04)	10.98 (9.65-12.53)	0.72% (0.63-0.82)
Southeast Asia	11.77 (10.51-13.17)	2.06% (1.84-2.29)	11.78 (10.51-13.13)	1.75% (1.56-1.95)	0.24 (0.19-0.30)	0.04% (0.03-0.05)	0.33 (0.27-0.40)	0.06% (0.05-0.07)	0.27 (0.20-0.36)	0.04% (0.03-0.06)	4.37 (3.79-5.01)	0.70% (0.61-0.81)
Southern Latin America	0.42 (0.35-0.50)	0.49 (0.40-0.57)	1.27 (1.12-1.41)	1.79% (1.59-2.01)	0.03 (0.02-0.04)	0.04% (0.03-0.05)	0.02 (0.01-0.02)	0.02% (0.02-0.03)	0.03 (0.02-0.04)	0.04% (0.03-0.05)	0.35 (0.29-0.40)	0.43% (0.37-0.50)
Southern Sub-Saharan Africa	0.27 (0.23-0.32)	0.52% (0.44-0.61)	0.98 (0.86-1.10)	1.41% (1.25-1.59)	0.04 (0.03-0.04)	0.07% (0.06-0.09)	0.02 (0.01-0.02)	0.04% (0.03-0.04)	0.03 (0.02-0.04)	0.05% (0.04-0.07)	0.23 (0.19-0.26)	0.31% (0.27-0.35)
Tropical Latin America	1.79 (1.51-2.08)	0.75% (0.63-0.87)	6.56 (5.82-7.31)	2.78% (2.47-3.09)	0.23 (0.19-0.29)	0.10% (0.08-0.12)	0.18 (0.14-0.22)	0.07% (0.06-0.09)	0.21 (0.15-0.28)	0.08% (0.06-0.11)	1.37 (1.20-1.56)	0.58% (0.51-0.66)
Western Europe	4.35 (3.68-5.09)	0.42% (0.36-0.49)	8.50 (7.58-9.43)	1.64% (1.45-1.84)	0.26 (0.20-0.34)	0.02% (0.02-0.03)	0.55 (0.43-0.68)	0.05% (0.04-0.06)	0.15 (0.11-0.21)	0.02% (0.01-0.02)	1.60 (1.36-1.89)	0.24% (0.20-0.27)
Western Sub-Saharan Africa	3.10 (2.67-3.54)	1.77% (1.53-2.02)	4.35 (3.82-4.92)	1.30% (1.14-1.46)	0.21 (0.17-0.26)	0.13% (0.10-0.16)	0.32 (0.25-0.40)	0.19% (0.15-0.23)	0.06 (0.05-0.09)	0.03% (0.02-0.04)	1.82 (1.59-2.08)	0.64% (0.56-0.74)
Global	83.48 (71.76-95.98)	1.01 (0.87-1.15)	157.5 (140.3-175.5)	1.91% (1.71-2.13)	4.13 (3.24-5.18)	0.05% (0.04-0.06)	6.23 (5.04-7.59)	0.07% (0.06-0.09)	3.28 (2.41-4.34)	0.04% (0.03-0.05)	40.47 (35.49-46.01)	0.49% (0.44-0.56)

Magnitude and causes of vision impairment in children

Methodology for estimating the number of children who are blind globally in 2020.

In the absence of precise estimates of the prevalence of blindness in children from population-based surveys, under 5 mortality rates have been used as a proxy indicator. There is also some evidence of an association between under 5 mortality rates and the prevalence of blindness in children, using the available data.[89] The rationale for this is that under 5 mortality rates indicate whether populations of children are at risk of vitamin A deficiency, which is associated with higher mortality and blindness.[90]

The under 5 mortality rate for the year half way through the 16 years of childhood (0-15 years) is used, as this best represents the prevailing socio-economic, public health and health system factors. For example, for the 2020 estimate, under 5 mortality rates for 2013 were used.[91]

The Global Burden of Diseases population projections for the year 2020 were used, which present data for each country by age group and sex (http://ghdx.healthdata.org/countries).

A spreadsheet of the child population aged 0-15 years (to be consistent with previous estimates) was prepared for every country by GBD region. 2013 under 5 mortality rates for each country were extracted and added, and the prevalence was estimated, using the scale outlined in Supplementary Table 6.[89] For example, the projected child population Chad in 2020 is 8,936,484; the under 5 mortality rate in 2013 was 148/1000 births, with a prevalence estimate of 1.0/1000 children. The total number of blind children is Chad is estimated to be 8,936.

Data from several recent studies of blindness in children, in Fiji, Bangladesh and Vietnam have population estimates which are very similar to those derived using under 5 mortality rates as a proxy.[92-94]

The regional numbers were derived from the country level estimates and shown in Supplementary Table 7 and represented in Supplementary Figure 4.

The major diseases causing blindness in children vary widely, being largely determined by socio-economic factors, and are represented schematically in Supplementary Figure 5. Globally approximately 40% of children are blind from conditions which could have been prevented or managed, with a higher proportion being avoidable in LICs.

In HICs the main causes of blindness are central nervous system lesions often as a consequence of preterm birth.[95] Potentially avoidable causes include cataract, glaucoma and ROP. In MICs, initiatives to reduce neonatal mortality through improving access to intensive neonatal care, are increasing the survival of preterm infants. This has led to a dramatic increase in blindness from ROP.[96] In LICs patterns of blindness have changed over the last 30 years as global strategies to control measles infection and vitamin A deficiency have reduced corneal blindness. In these countries cataract has become the most common avoidable cause, and ROP is emerging in the larger cities. In all regions unavoidable causes include genetic retinal conditions and congenital eye anomalies. These children require early vision rehabilitation.

Supplementary Table 6: Prevalence estimates for blindness and severe visual impairment using under 5 mortality rates as a proxy.

Under 5 mortality rate/1000 births	Blindness prevalence estimate[89]
0-19	0.3 per 1,000
20-39	0.4 per 1,000
40-59	0.5 per 1,000
60-79	0.6 per 1,000
80-99	0.7 per 1,000
100-119	0.8 per 1,000
120-139	0.9 per 1,000
140-159	1.0 per 1,000
160-179	1.1 per 1,000
180-199	1.2 per 1,000
200-219	1.3 per 1,000
220-239	1.4 per 1,000
240+	1.5 per 1,000

Supplementary Table 7: Number of children who are blind by Global Burden of Disease Region, 2020

GBD region	Number blind	Regional prevalence / 1000
South Asia	298901	5.4
Western Sub-Saharan Africa	167673	7.8
Eastern Sub-Saharan Africa	117112	6.1
North Africa and Middle East	82830	4.2
Southeast Asia	75057	4.2
East Asia	74431	3.0
Central Sub-Saharan Africa	51299	8.6
Central Latin America	23926	3.2
Western Europe	21925	3.0
High-income North America	21518	3.0
Tropical Latin America	15822	3.0
Southern Sub-Saharan Africa	14590	5.6
Central Asia	13220	4.7
Eastern Europe	11583	3.0
High-income Asia Pacific	7500	3.0
Caribbean	6718	4.4
Central Europe	5526	3.0
Andrean Latin America	5294	3.3
Southern Latin America	4843	3.0
Oceania	2701	5.6
Australasia	1790	3.0
Total	1,024,260	0.48

Supplementary Figure 4: Number of children who are blind by Global Burden of Disease Region, 2020

Regional estimates of the total number of blind children were derived using under 5 mortality rates as a proxy for prevalence, methodology is described in the supplementary annex. The number at the top of each bar is the regional prevalence in number per 10,000 children.

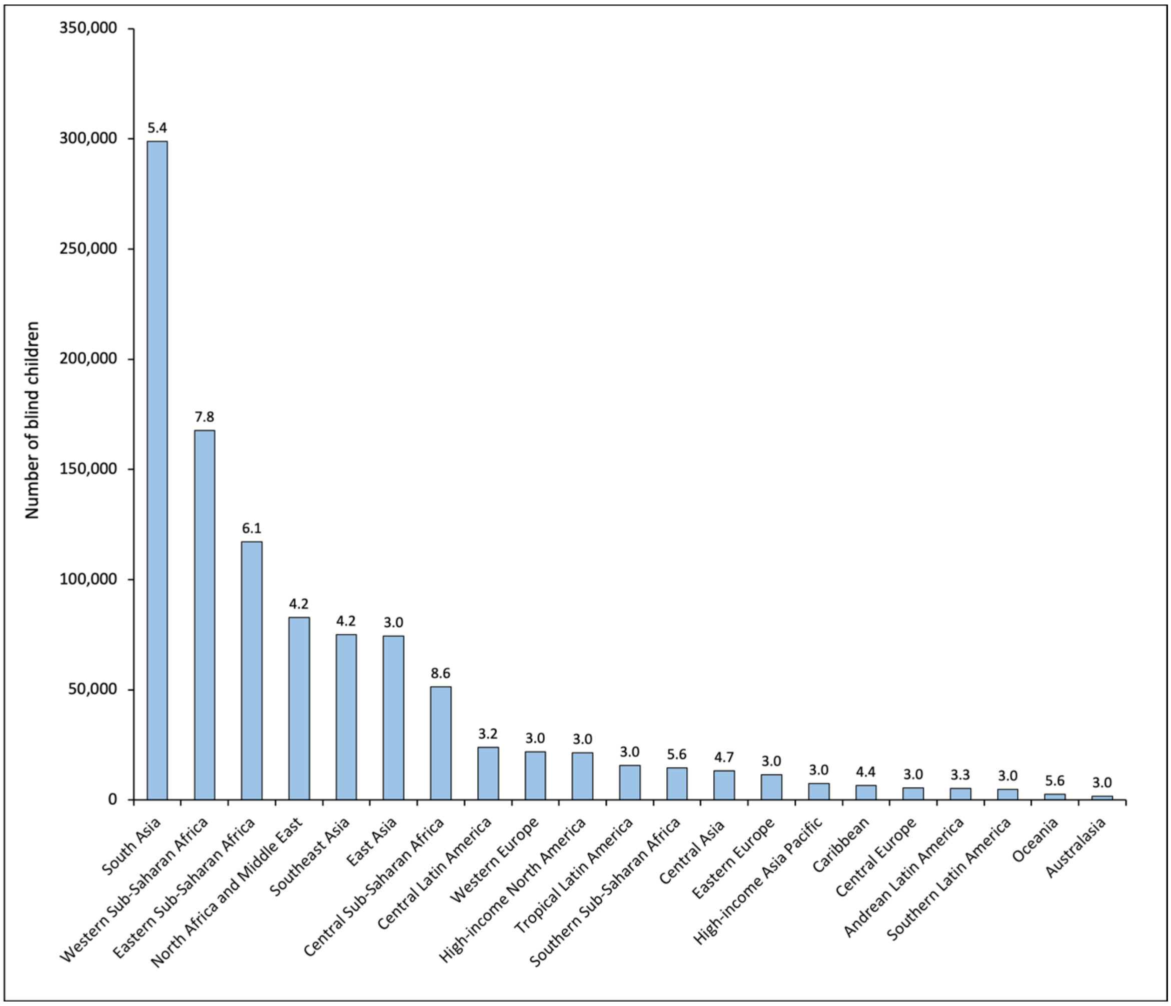

Supplementary Figure 5: Schema of the number and main causes of blindness in children per 10 million population, by level of socio-economic development

Adapted from: Visual impairment and blindness in children. Clare Gilbert and Jugnoo Rahi. Epidemiology of Eye Disease. 3rd Edition. 2012 Ed. G Johnson, D Minassian, R Weale, S West.

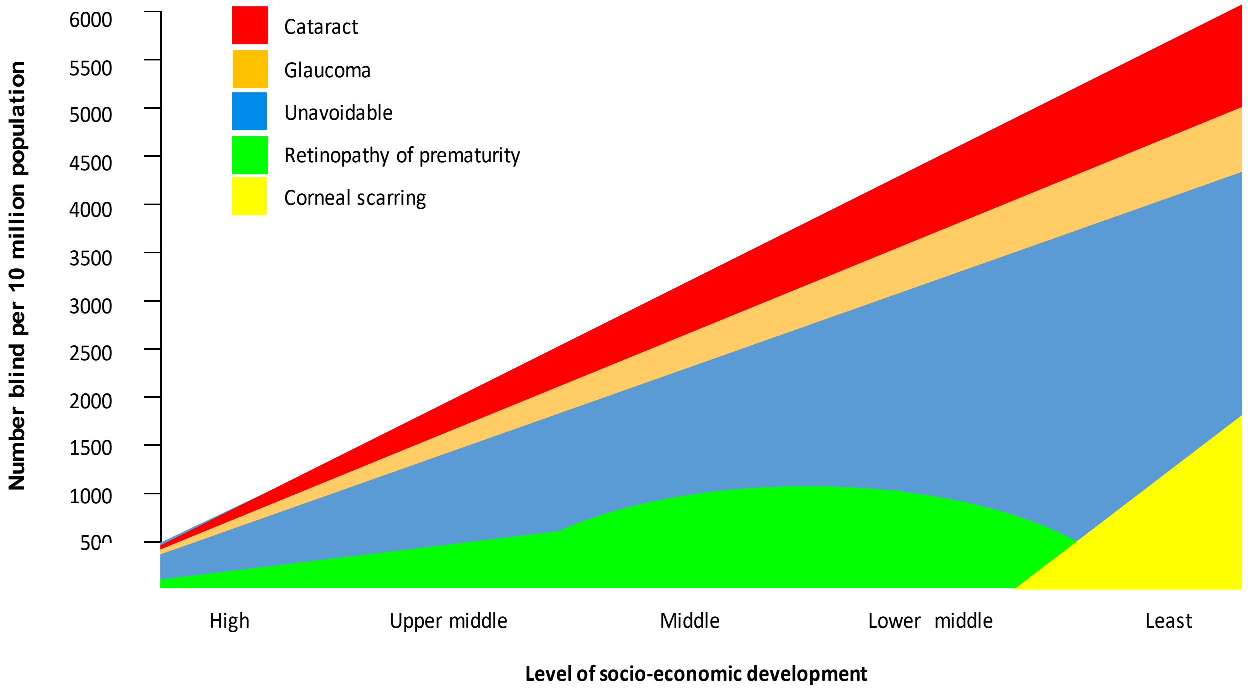

Temporal trends in vision impairment

Supplementary Table 8: Gender differences in the age-standardised prevalence of blindness and moderate and severe vision impairment.

The gender difference in adults ≥50 years was derived from: age-standardised % in women - age-standardised % in men. MSVI, moderate and severe vision impairment. Data source: GBD/VLEG 2020 data.[88]

Region	Blind (%)			MSVI (%)		
	Men	Women	Difference	Men	Women	Difference
Andean Latin America	2.21	2.18	-0.03	12.91	13.09	0.18
Australasia	0.49	0.49	0.00	3.88	4.24	0.36
Caribbean	1.84	1.65	-0.19	8.08	8.42	0.34
Central Asia	1.56	1.44	-0.13	10.97	12.72	1.75
Central Europe	0.58	0.58	-0.01	5.98	7.44	1.46
Central Latin America	1.87	1.79	-0.08	10.65	10.86	0.21
Central Sub-Saharan Africa	1.74	1.81	0.07	10.18	9.86	-0.32
East Asia	1.40	1.69	0.29	8.37	10.56	2.19
Eastern Europe	0.87	0.87	0.00	10.05	12.38	2.33
Eastern Sub-Saharan Africa	3.79	4.16	0.37	10.89	11.59	0.70
High-income Asia Pacific	0.47	0.39	-0.09	3.82	3.97	0.16
High-income North America	0.41	0.39	-0.03	2.88	3.62	0.74
North Africa and Middle East	2.57	2.81	0.24	12.39	13.72	1.32
Oceania	1.74	2.22	0.48	16.80	17.80	1.00
South Asia	3.31	3.73	0.42	22.00	23.87	1.87
Southeast Asia	3.05	4.18	1.14	14.93	15.77	0.84
Southern Latin America	0.72	0.61	-0.11	6.06	7.03	0.97
Southern Sub-Saharan Africa	3.32	2.95	-0.37	7.55	7.20	-0.36
Tropical Latin America	2.74	2.65	-0.09	10.50	10.63	0.13
Western Europe	0.55	0.64	0.09	4.63	5.57	0.94
Western Sub-Saharan Africa	4.15	4.29	0.13	13.52	15.28	1.76

Supplementary Figure 6: Gender differences in the age-standardised prevalence of blindness and moderate and severe vision impairment.

The gender difference in adults aged ≥50 years was derived by calculating: [age-standardised prevalence in women aged ≥50 years] – [age-standardised prevalence in men aged ≥50 years]. Data are from Supplementary Table 8 and are plotted for the 21 GBD Regions. LA, Latin America. SSA, Sub-Saharan Africa. MSVI, moderate and severe vision impairment. Data source: GBD/VLEG 2020 data.[88]

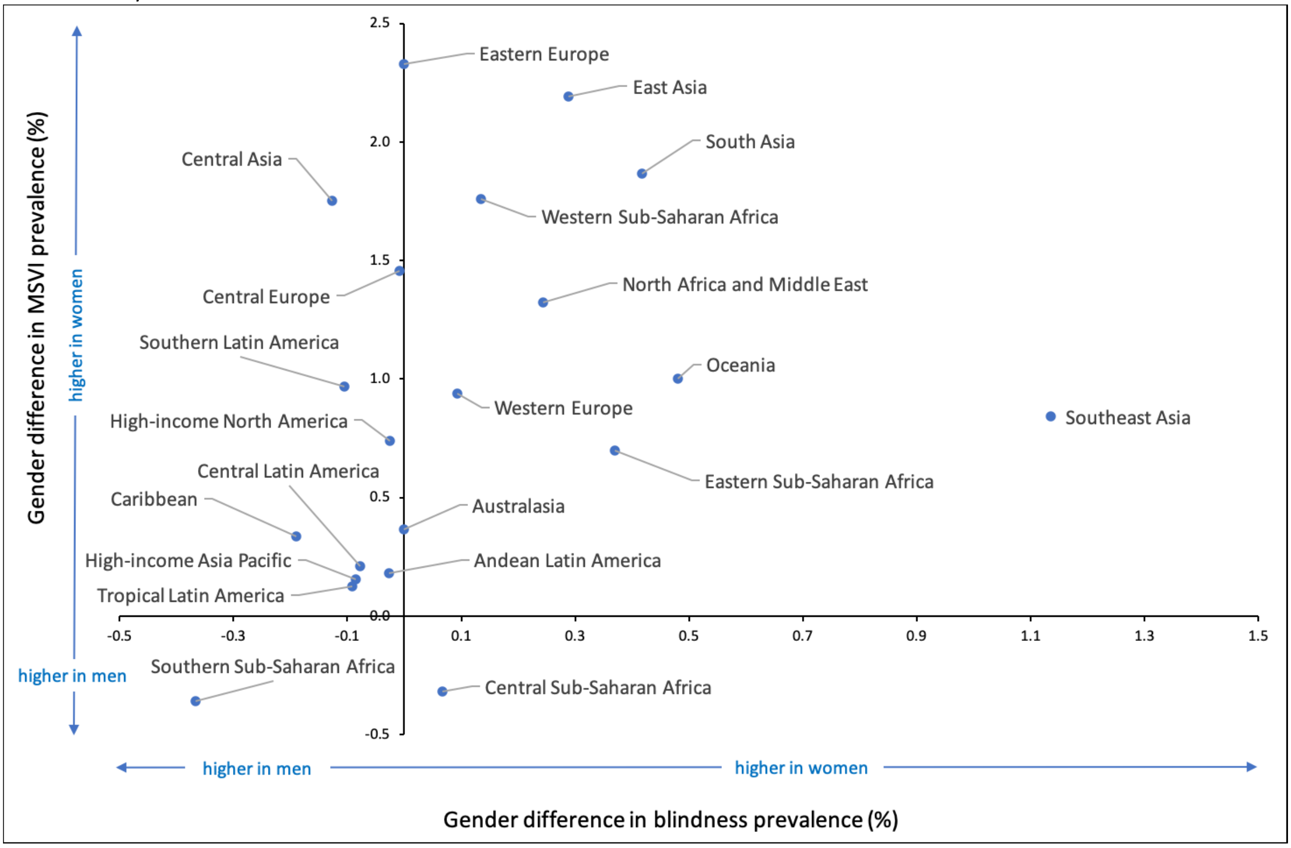

Supplementary Table 9: Excess number of women over men with blindness and moderate and severe vision impairment.

The excess number of women over men, in adults ≥50 years. Size of the bubble represents the total number of adults ≥50 years with blindness and MSVI in that region. MSVI, moderate and severe vision impairment. Data source: GBD/VLEG 2020 data.

The data plotted in Commission Report Figure 10 are outlined below.

Region	Blind			MSVI			Total Blind & MSVI
	Men n	Women n	Excess women	No. men	No. women	Excess women	
Andean Latin America	72,594	94,004	21,410	465,543	564,454	98,911	1,196,594
Australasia	10,564	12,543	1,979	110,977	111,204	227	245,287
Caribbean	4,365,168	5,216,852	851,684	32,075,348	36,762,569	4,687,221	78,419,936
Central Asia	95,102	126,736	31,634	738,269	1,195,247	456,977	2,155,354
Central Europe	512,072	837,719	325,647	4,257,994	6,823,678	2,565,684	12,431,463
Central Latin America	148,237	205,070	56,833	361,971	515,110	153,138	1,230,388
Central Sub-Saharan Africa	451,417	518,387	66,971	2,603,933	3,173,448	569,515	6,747,185
East Asia	633,725	796,190	162,465	2,493,393	3,198,023	704,630	7,121,331
Eastern Europe	130,875	143,827	12,953	773,317	853,432	80,115	1,901,451
Eastern Sub-Saharan Africa	262,755	328,069	65,314	1,841,976	2,933,373	1,091,397	5,366,172
High-income Asia Pacific	211,053	239,046	27,993	1,688,913	2,444,119	755,206	4,583,131
High-income North America	113,313	165,679	52,366	1,200,230	2,082,886	882,656	3,562,108
North Africa and Middle East	98,722	106,168	7,446	436,939	533,715	96,776	1,175,544
Oceania	3,141,101	4,122,650	981,550	18,768,812	26,123,912	7,355,100	52,156,474
South Asia	25,704	31,749	6,045	204,085	269,678	65,593	531,215
Southeast Asia	1,676,473	2,873,033	1,196,560	8,586,701	11,272,611	2,685,910	24,408,818
Southern Latin America	535,597	668,478	132,880	1,629,450	1,983,778	354,328	4,817,303
Southern Sub-Saharan Africa	702,926	789,362	86,437	2,450,519	3,089,003	638,484	7,031,810
Tropical Latin America	56,533	68,354	11,820	487,001	763,944	276,943	1,375,832
Western Europe	1,088,269	1,256,915	168,646	5,629,161	6,398,541	769,381	14,372,887
Western Sub-Saharan Africa	231,047	450,578	219,530	2,825,748	6,139,620	3,313,873	9,646,993

Supplementary Table 10: Global and regional numbers of adults (aged 20 – 79 years) estimated to have DM, DR and VTDR in 2019 and projected for 2045.

Regions are ordered on the projected % increase between 2019 and 2045. This table summarises the number of adults with diabetic retinopathy (DR) and vision-threatening diabetic retinopathy (VTDR) in 2019 and projected for 2045 by region, giving an indication of where the largest projected increase is expected. These estimations were calculated by multiplying the estimated number of people with DM by region and globally (taken from Table 1. Saeedi et al. 2019[97]) by the global DR and VTDR prevalence estimates (taken from Table 2. Yau et al. 2012[98]). These numbers provide a sense of the magnitude of DR and VTDR, however as there is known heterogeneity in the prevalence of DR across world regions and countries (Lee et al. 2015[99]), they are to be taken only as estimations.

Region	2019 - Number (millions)			2045 - Number (millions)			% Increase
	DM	DR	VTDR	DM	DR	VTDR	
Sub-Saharan Africa	19.4	6.7	2.0	47.1	16.3	4.8	242.8%
Middle East & North Africa	54.8	19.0	5.6	107.6	37.2	11.0	196.4%
South – East Asia	87.6	30.3	8.9	152.8	52.9	15.6	174.4%
South and Central America	31.6	10.9	3.2	49.1	17.0	5.0	155.4%
North America & Caribbean	47.6	16.5	4.9	63.2	21.9	6.4	132.8%
Western Pacific	162.6	56.3	16.6	212.2	73.4	21.6	130.5%
Europe	59.3	20.5	6.0	68.1	23.6	6.9	114.8%
Global	463.0	160.2	47.2	700.2	242.3	71.4	151.2%

DM – diabetes mellitus; DR – diabetic retinopathy; VTDR – vision-threatening DR

Supplementary Table 11: Global myopia trends, 2000 to 2050

Global myopia trends, 2000 to 2050

Estimated number of people globally with myopia (≥ –0.5 D) and high myopia (≥ –5 D) in millions from 2000 to 2050. The broken lines represent the upper and lower 95% confidence intervals. Adapted from Holden et al.[13]

Year	Myopia	Lower limit	Upper limit	High myopia	Lower limit	Upper limit
2000	1406	932	1932	163	86	387
2010	1950	1422	2543	277	153	589
2020	2620	1976	3366	399	233	815
2030	3361	2578	4217	517	298	1082
2040	4089	3145	5128	696	381	1518
2050	4758	3620	6056	938	479	2105

Supplementary Figure 7: Global myopia trends, 2000 to 2050

Adapted from Holden et al.[13]

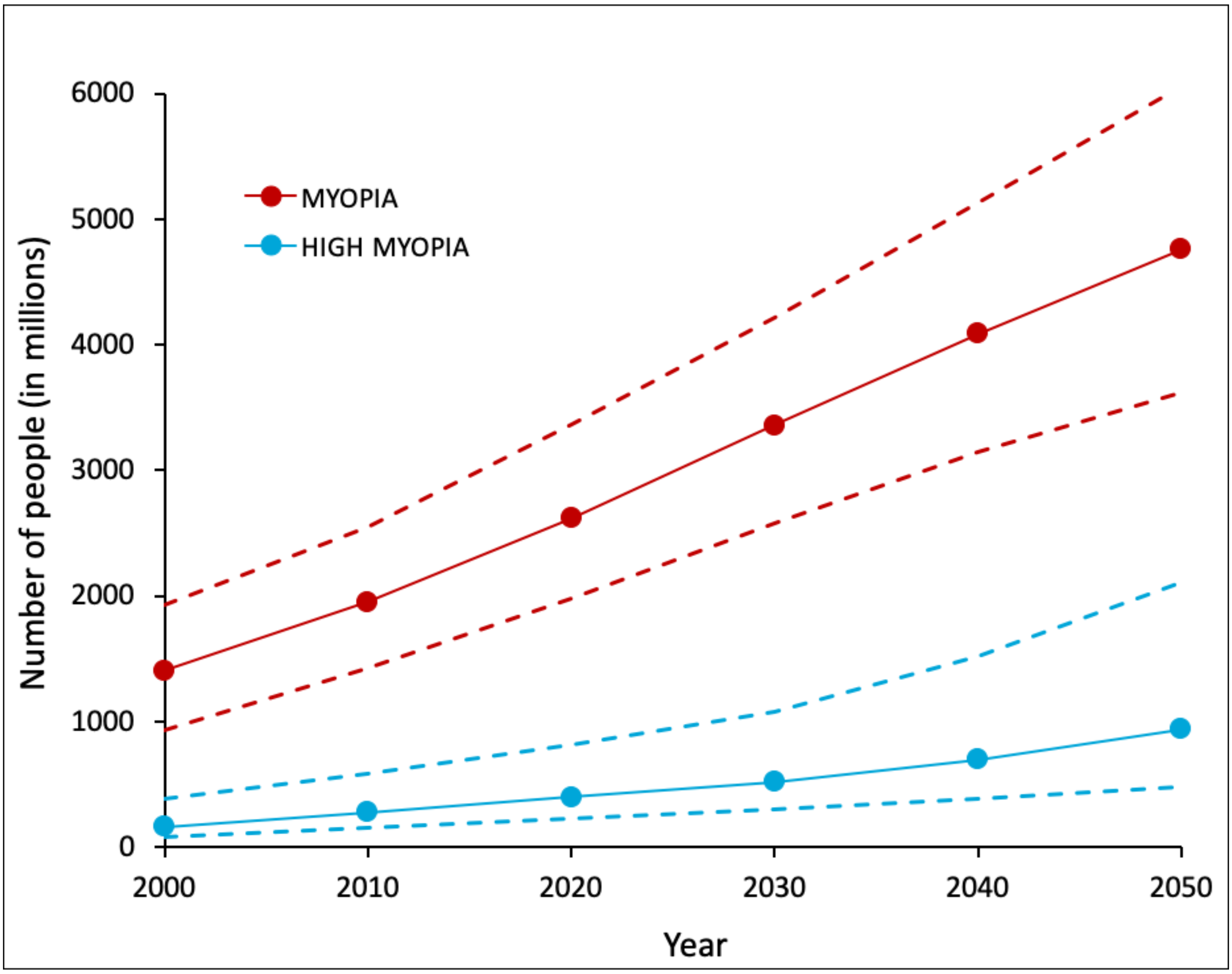

Non-visually impairing ocular conditions: contribution to global eye care need

Supplementary Figure 8: The visual acuity level of people attending secondary eye hospitals in India.
Presenting visual acuity in the worse eye of 272,000 patients attending secondary eye hospitals of Aravind Eye Care System in India. Data were collected in 2019 by Aravind Eye Hospital.

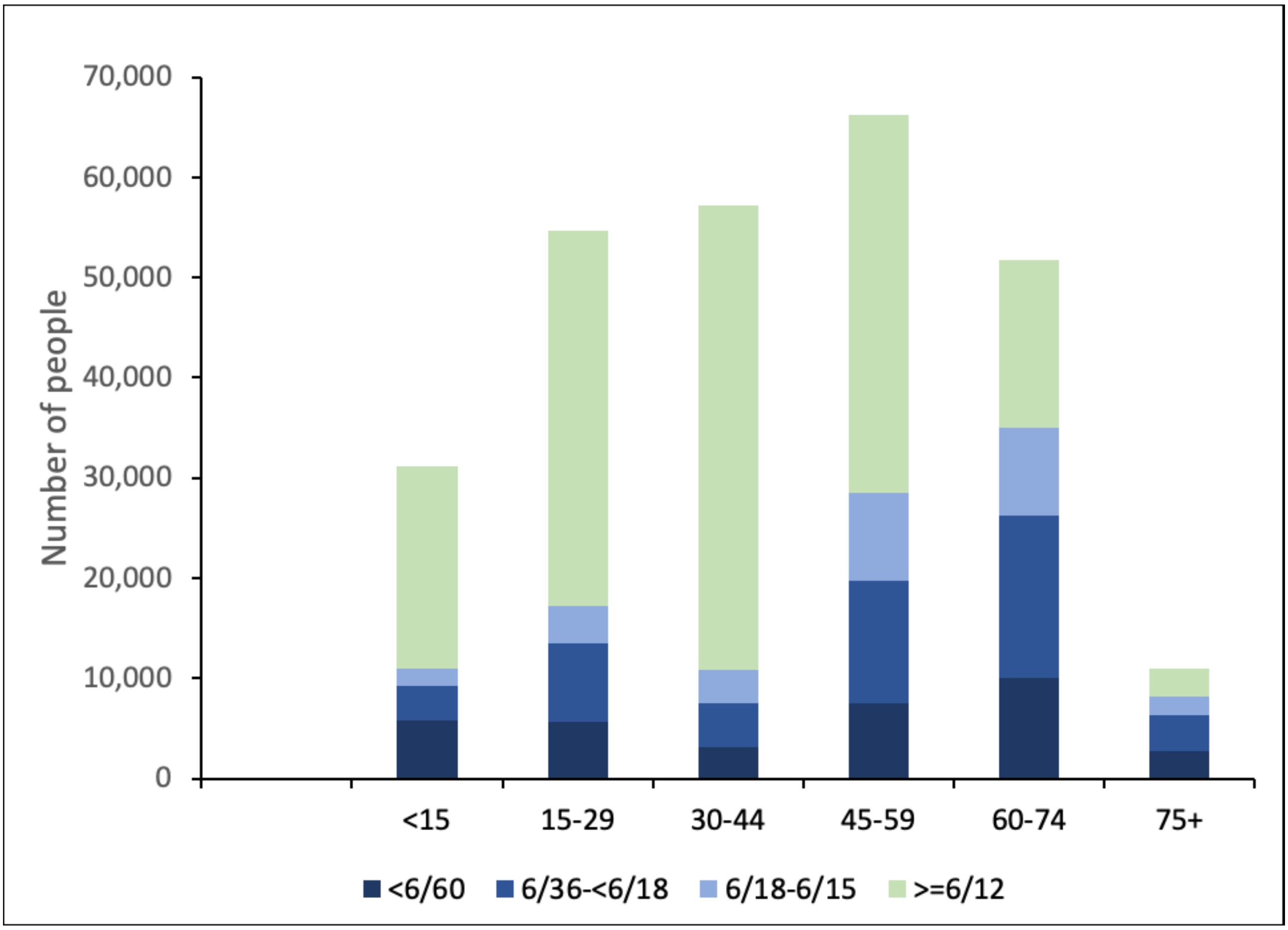

Supplementary Table 12: The visual acuity level of people attending secondary eye hospitals in India.
Presenting visual acuity in the worse eye of 275,000 patients attending secondary eye hospitals of Aravind Eye Care System in India. Data were collected in 2019 by Aravind Eye Hospital.

	Presenting visual acuity in the worse eye				
**Age (years)**	**6/12 or better**	**<6/12 ≥ 6/18**	**<6/18 ≥ 6/60**	**worse than 6/60**	**Total**
<15	21,492	1,430	2,821	5,401	31,144
15-29	40,502	3,103	6,501	4,592	54,698
30-44	50,293	2,345	2,774	2,030	57,442
45-59	49,207	7,413	7,527	3,066	67,213
60-74	30,579	9,282	10,149	3,691	53,701
75+	5,461	2,333	2,385	1,283	11,462
**Total**	**197,534**	**25,906**	**32,157**	**20,063**	**275,660**

Quantifying the magnitude: survey methodologies

Representative population-based surveys provide accurate estimates of disease prevalence, service coverage and outcomes.[100] In ophthalmic epidemiology, stakeholders are interested in the prevalence and magnitude of eye diseases, vision impairment and blindness. In some cases this may involve a focus on marginalised sub-groups, associated risk factors, individual and societal impacts, and access to eye care services.[101] These data are needed to support effective planning, resourcing, implementation, monitoring and evaluation of services, and can be aggregated to provide regional or global estimates for advocacy and comparison purposes.[88,102-104]

There are several survey methodologies used to obtain a population-representative random sample.[105,106] These include taking a simple or systematic random sample of individuals or clusters of individuals, with or without stratification. An important principle is that each individual in the target population has an equal chance of being selected to take part in the study. This can be achieved by techniques such as sampling without replacement and maintaining probability sampling.

Survey methodology depends on the goals and the sample size needed. Broadly, there are two approaches to eye health surveys, comprehensive and rapid. Supplementary Table 13 compares the key attributes of comprehensive surveys with the most common rapid assessment method (RAAB, the Rapid Assessment of Avoidable Blindness). The most applicable approach ultimately balances epidemiological rigour and resources.

Comprehensive eye health surveys typically include in-depth ophthalmic examination with imaging for independent retinal grading, and rich data on risk factors.[107-109] The need to diagnose eye disease in field testing sites, rather than in a well-equipped hospital clinic, necessitates some diagnostic compromises, which are reflected in epidemiological case definitions of disease. Good progress has been made towards standardising case definitions for some eye diseases, such as glaucoma, cataract, and posterior segment diseases visible on retinal imaging.[110] However, with variation in equipment, staff and examination protocols, case definitions continue to vary between surveys, which can present a challenge for comparability.[104,111,112] Greater standardisation of methodologies and reporting of comprehensive eye health surveys is imperative.[9]

A number of rapid assessment methodologies are available for generating population-based estimates of common causes of vision impairment.[113] The most commonly used of these is the Rapid Assessment of Avoidable Blindness (RAAB), originally developed as the Rapid Assessment of Cataract Surgical (RACSS) with a focus on planning and monitoring district-level cataract services in India in the 1990s.[114-116] "RAAB" is used to describe RAAB and its predecessors throughout this text. RAAB is a standardised methodology, that only includes the population 50 years and older, in which the prevalence of blindness is relatively high, and therefore the required sample size (and associated costs) can be lower compared to an all-age survey. It also uses relatively simple examination techniques, again limiting resource need, however this also limits the ability to diagnose some conditions.[117] Similar, but less widely used, rapid assessments include the Rapid Assessment of Refractive Error (RARE, population of interest 15-50 years) and the Rapid Assessment of Visual Impairment (RAVI, population of interest 40 years and older).[118,119] A recent study in rural China compared the findings of a RAAB to a comprehensive survey, conducted on the same group of people, by separate teams.[120] RAAB had >90% sensitivity and specificity for detecting blindness, vision impairment and impairment due to cataract.

Supplementary Table 13: Comparison of comprehensive and rapid survey methodologies

	**Comprehensive Survey**	**Rapid Survey (RAAB and RARE)**
Primary Aim	To estimate prevalence of VI and blindness	To estimate prevalence of avoidable vision impairment and blindness
Secondary Aim	To estimate prevalence of many eye diseases, risk factors, impacts and access to eye care services.	To estimate prevalence of cataract and URE and access to eye care services
Population of Interest – age group	Any age. Varies depending on primary aim	Varies depending on primary aim, for example 50+ in RAAB and 15 – 49 in RARE
Population of Interest - size	Any size – national, regional or local.	Usually sub-national, although multiple sub-national surveys have to be aggregated with weighting to provide national estimates
Population of interest – special populations	Usually the non-institutionalised population, but with some surveys including or focusing on subgroups, including homeless/refugees, institutionalised (special schools, nursing homes, prisons etc)	Exclusively the non-institutionalised population who are usual residents of the selected households.
Sample Size Calculation	Varies. It may be based on disease prevalence (lowest prevalence out of all diseases of interest), blindness prevalence or vision impairment prevalence (which differ depending on presenting or best-corrected)	Based on expected prevalence of blindness or SVI / blindness 50+ and both sexes. This limits the ability to disaggregate by age-sex group or to estimate disease prevalence
Visual Acuity Measurement - Distance	Measurement protocols (stopping rules, illuminance, test distance) and vision charts (optotype, crowding, scale) vary. Usually a continuous measure of visual acuity, including in the 'best-corrected' (following subjective refraction) or autorefracted (as a proxy) state, in addition to 'presenting' acuity.	Measurement protocol standardised, with use of a single 'Tumbling E' optotype (size 6/12, 6/18 and size 6/60), to screen for vision impairment in presenting and pinhole states. Since 2017 mRAAB has included a digital VA test using an E optotype with a crowding box. Uncorrected VA is being built into RAAB methodology in 2020.
Visual Acuity Measurements - Near	Measurement protocols and chart types (e.g. reading charts versus acuity charts) vary	Not collected up to 2020
Examination Techniques	Varies. Usually includes ophthalmoscopy and slit lamp examination with tonometry +/- fundus imaging, visual field assessment, biometry etc. Dilation in all, or in those with VI, is standard	Simplified: ophthalmoscopy, torchlight and possibly slit lamp. Dilation non-standard unless anterior pathology not identified, or optional diabetic retinopathy module incorporated.
Personnel	Variable. May include Optometrists, Ophthalmic Nurses, Technicians, and Ophthalmologists	Examination by ophthalmologist (no specialisation required unless completing Diabetic Retinopathy module), with vision screening by Optometrists, Ophthalmic Nurses or equivalent.
Timeframe	Usually 6 – 30 months	Usually 6 – 8 weeks
Standardisation	No single standardised survey methodology, so not always easy to compare surveys across different settings and time points. Trade-off between standardisation and flexibility to answer different research questions in different sub populations.	Standardised methods, analysis, reporting (using WHO definitions for VI category) and team training
Quality Assurance	Varies by survey. Not standardised.	Standardised, and includes certified trainer scheme, bespoke software for sample size calculation, sample selection, data entry and automated analyses and reports[117]
Repository	Global Vision Database – continuously updated database of population-based studies curated by Vision Loss Expert Group, not publicly available.[102]	RAAB Repository, currently holds RAAB data only when permitted by RAAB principal investigators. A new publicly repository is currently being developed.[117]

Eye Health Survey Data Gaps

Supplementary Figure 9: Population-based eye health surveys conducted worldwide (2000-2020)

The maps indicate where and when the most recent eye health surveys have been conducted since 2000. These are a mixture of both national and sub-national surveys. (A) All RAABs surveys since 2000 (Source: RAAB Repository). (B) All comprehensive surveys since 2000 (Source: Vision Loss Expert Group, list used to compile the 2020 GBD report with additions based on personal communications). (C) All RAAB Surveys since 2000 – Sub-national studies mapped to Admin Level 1. (D) Composite map showing the most recent survey (national or sub-national) completed in each country since 2000.

The following methods were used to generate the maps of survey coverage.

Admin Level 0 (Country) and Admin Level 1 (highest within-country administrative region) world base maps were sourced from Natural Earth (www.naturalearth.com), a free resource compiled by global cartographers.

Data on RAABs since 2000 were sourced from the RAAB Repository (http://raabdata.info/). RAAB meta data stored in the repository back-end as a CSV were imported into R. National level RAABs were joined directly to the Admin Level 0 base map, whilst sub-national RAABs were first joined to a dataset of ISO_3166_2 Subdivision Admin 1 codes, a free resource available from www.ip2location.com, and then the Admin Level 1 map.

The VLEG 2020 source list was converted from Word to Excel, and then imported into R.[88] Pre 2000 sources, and sources marked “Rapid” were deleted from the list so that only comprehensive studies remained. In its current format, the VLEG source list denotes national versus sub-national comprehensive surveys, but does not detail which sub-national area the latter covers. Therefore, both national and sub-national comprehensive surveys have been joined to the Admin Level 0 world base map for presentation.

In each case, the most recent survey per country is depicted on the map. Where national and sub-national level data exist simultaneously, sub-national data that are more recent than national data are layered above national data. Antarctica is not displayed on any of the maps.

Developing reliable estimates of the prevalence and pattern of eye disease depends on good quality population-based data from surveys. Where there are no or limited data for a region, modelling approaches are sometimes used to fill in gaps. The Global Vision Database contains data from >300 surveys (comprehensive and rapid) conducted since 1980. Overall, there have been more than 330 RAABs across 81 countries to date, many of which contribute to the GBD estimates.[121] Supplementary Figure 8 presents population-based eye health surveys since 2000 (RAABs, comprehensive and combined). There have been 44 national RAABs since 2000 (30 in the last 10 years), predominantly in Central and Southern Latin America, and Southeast Asia. Over 200 subnational RAABs have been completed in the last decade, providing data from the majority of provinces or states in South Asia and much of Eastern and Southern Sub-Saharan Africa. Only five comprehensive, national surveys have been completed since 2010: Spain (2011), Trinidad and Tobago (2013), Sri Lanka (2015), Australia (2016) and The Gambia (2019).[122-124] In comparison, over 20 sub-national comprehensive studies have been completed in the same time period across 11 countries.

Certain regions have fewer survey data available than others. Most notably, since 2000 scant data are available for the majority of HICs across North America and Central Europe. In addition, there are comparably fewer, or older, data available for Western, Central, and some of Southern Sub-Saharan Africa, and Eastern Europe. The reasons why some countries have undertaken multiple surveys to support service planning, while others have never collected data from their populations, requires further investigation. While RAAB is available in French, Francophone countries may need additional support to initiate surveys, and for Lusophone countries, RAAB is yet to be translated.

The data indicate a near ten-fold difference in the quantity of RAABs versus comprehensive surveys at both national and sub-national level over the last two decades. RAABs are not a substitute for comprehensive surveys, and the advancement of mobile technology, artificial intelligence and tele-medicine will continue to improve the affordability and feasibility of comprehensive eye health surveys over time. The availability of standardised rapid methodologies such as RAAB to provide reliable eye health planning data is a valuable contribution to the evidence base alongside comprehensive surveys.

Supplementary Figure 8A: All RAAB surveys since 2000

This map presents all RAABs known to the RAAB Repository (whether or not data, reports or publications are available on the Repository) from 2000 onwards. National and Sub-National RAABs are displayed at the national level (Admin Level 0) and denoted by colour scheme (green denotes sub-national, red denotes national).

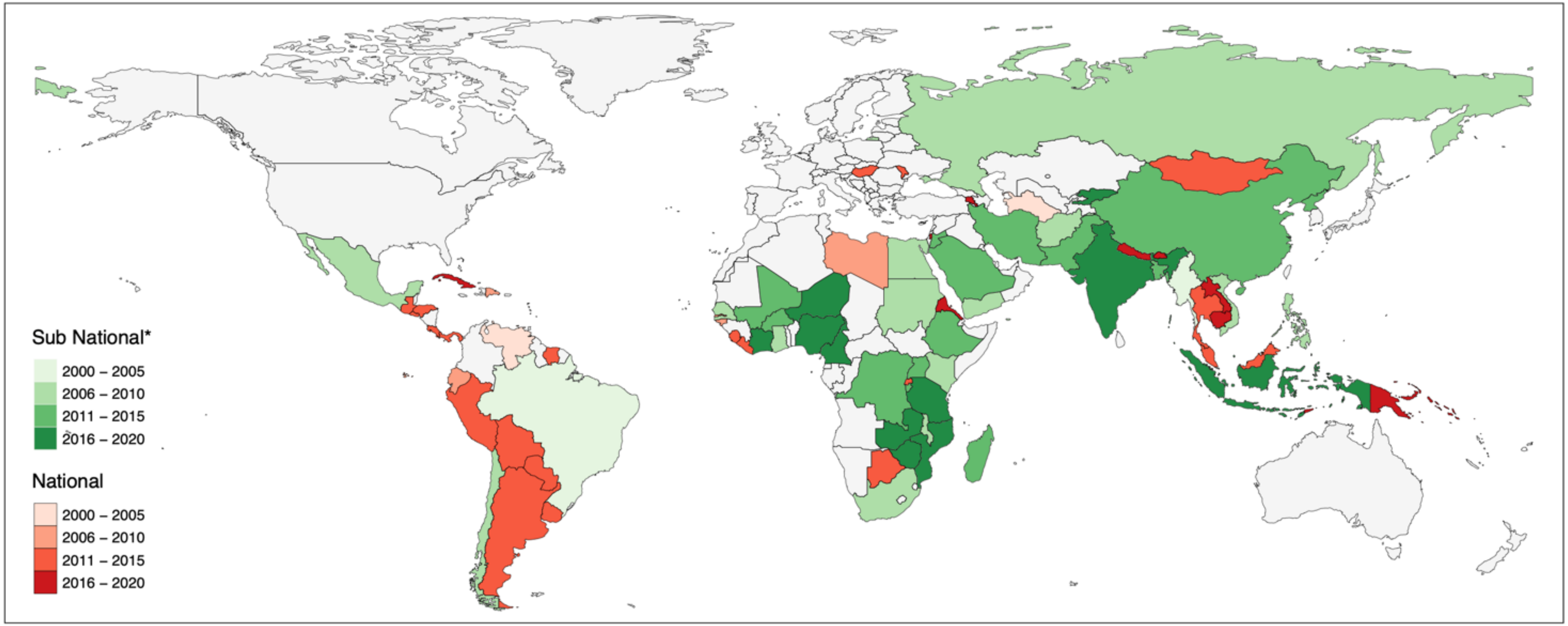

Supplementary Figure 8B: All comprehensive surveys since 2000

This map presents all surveys denoted "Comprehensive" in the VLEG source list since 2000. National and Sub-National comprehensive surveys are displayed at the national level (Admin Level 0) and denoted by colour scheme (green denotes sub-national, red denotes national).

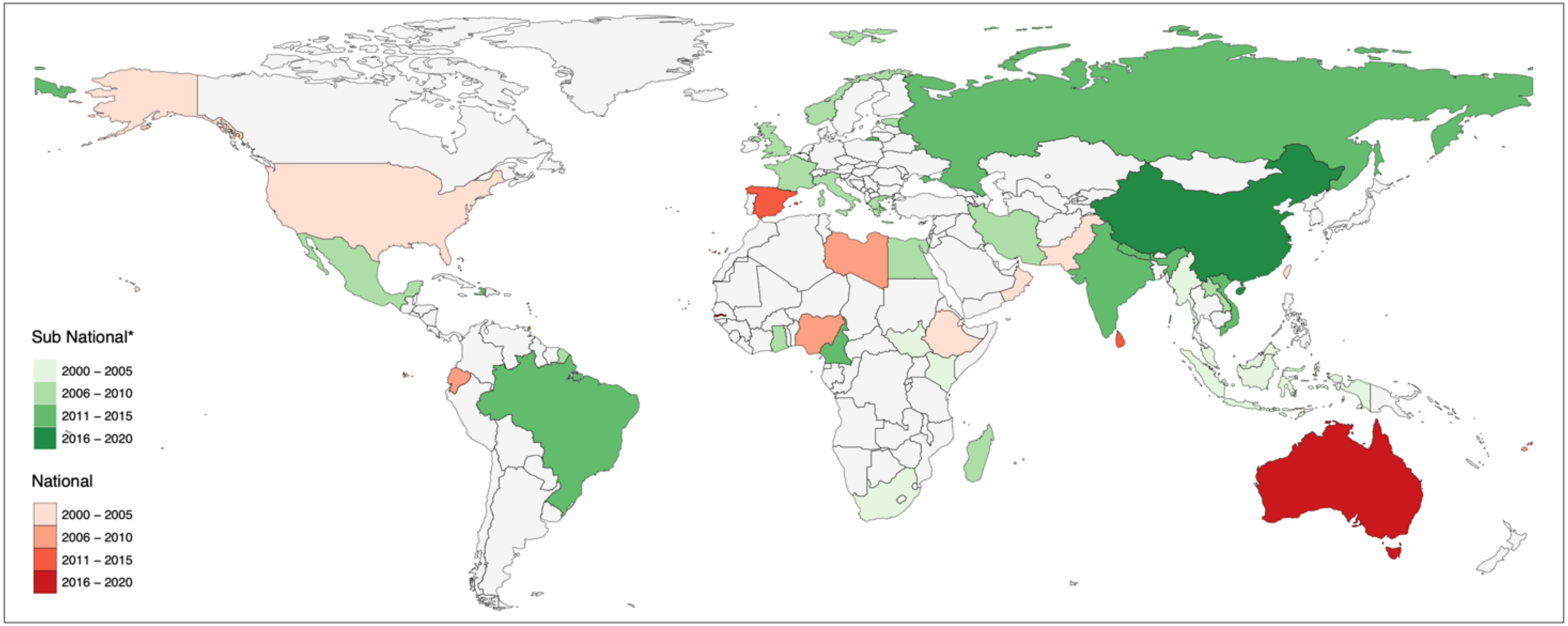

Supplementary Figure 8C: All RAAB Surveys since 2000 – Sub-national studies mapped to Admin Level 1

This map presents the same data as Map A, but whilst national-level data are still presented at a national level, sub-national level data in this map are presented at Admin Level 1. Admin Level 1 is the largest sub-national administrative level in each country, and may still represent a larger area than the RAAB sampling frame.

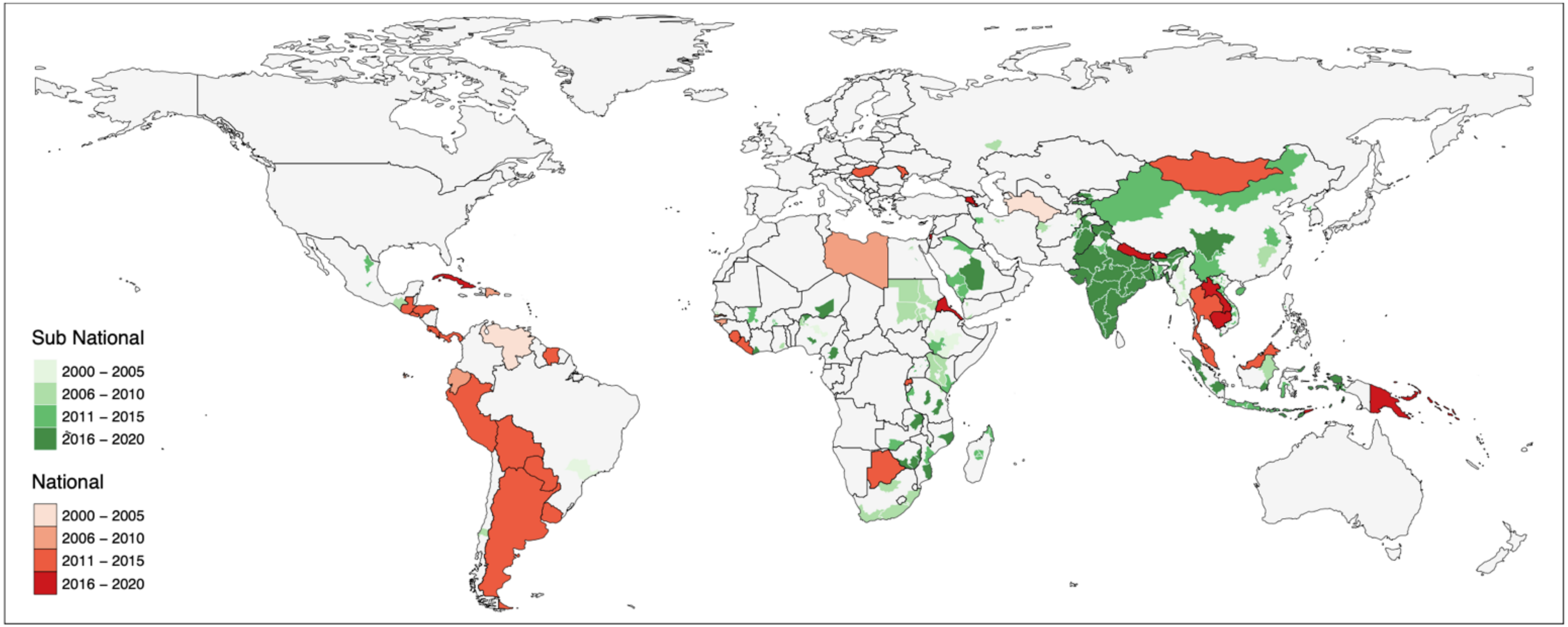

Supplementary Figure 8D: All surveys (rapid and comprehensive, national or sub-national) since 2000

This map highlights the countries which have no data presented in Map (A) and Map (B). The map does not distinguish between RAAB vs comprehensive but presents the most recent survey completed in each country. Note the presentation is at national level, even if the most recent survey in the country was sub-national. Note also that the RAAB and VLEG Comprehensive source lists may not be exhaustive of eye health surveys including rapid surveys that are not RAAB, surveys of specific population subgroups.

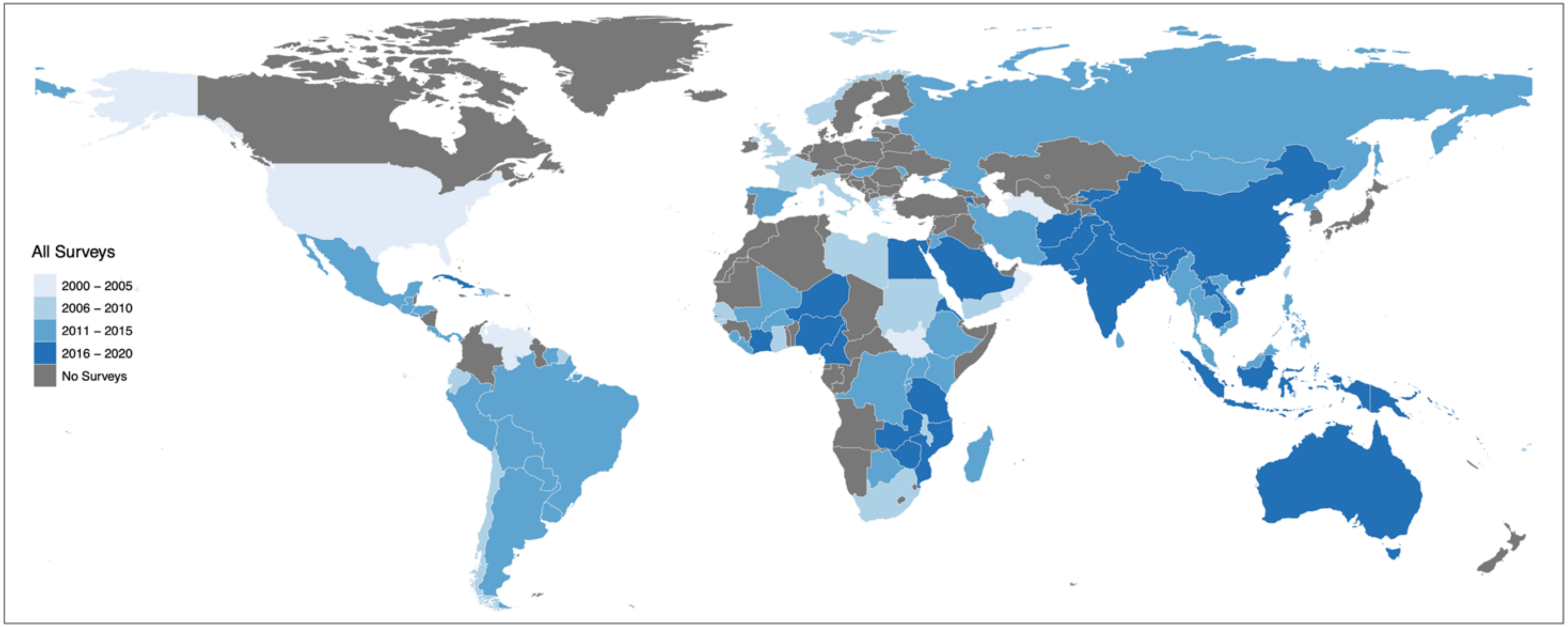

Reporting eye health survey data

Methods for Commission Report Panel 3: Key recommendations for improving population-based vision impairment survey design, conduct, reporting and interpretation of bias

Panel Members: A Bastawrous, RRA Bourne, T Braithwaite, A Bron, C Bunce, M Burton, R Casson, N Clough, N Congdon, A Conolly, AK Denniston, M Dirani, J Evans, J Foreman, J Furtado, S Gichuhi, E Harding-Esch, J Jonas, J Keeffe, J Kempen, V Lansingh, J Leasher, N Leveziel, H Limburg, I Mactaggart, M Malik, M Meltzer, I McCormick, GVS Murthy, K Naidoo, M Novak, K Ono, MR Ortiz, A Palagyi, T Peto, J Ramke, P Ramulu, S Resnikoff, S Salomao, S Selvam, BR Shamanna, I Sheriff, Y Shweikh, J Silva, A Solomon, GA Stevens, H Taylor, R Varma, R Wormald, and 4 anonymous panel contributors.

We launched the proposal to use a Delphi expert consensus process to develop more standardised guidelines for the reporting and design/conduct of population-based vision impairment prevalence surveys at the World Ophthalmology Congress workshop in 2016 in Mexico, inviting interested ophthalmic epidemiology experts to join the project working group. In 2019, the list of working group members was reviewed by the project steering committee, and extended through snowball sampling, seeking to recruit investigators with expertise of delivering surveys in all world regions. The study was approved by the London School of Hygiene & Tropical Medicine Ethics Committee.

The two round Delphi was set up and distributed online. We used the 22-item STROBE checklist as the foundation of the questionnaire, to which we added additional items of specific relevance and potential importance to the reporting, design, conduct or analysis of population-based vision impairment prevalence surveys. Panel members were asked whether they considered each item to be essential or not essential for the conduct of eye health surveys, considering comprehensive and rapid survey designs independently. They were also invited to add additional items they considered important. The Round 1 summary results and commentary, plus new items added by panel members, were presented in Round 2, with invitation to review earlier responses and comment further.

To identify the most important sources of bias in eye health surveys, in Round 1 we presented panel members with 14 potential sources of bias and asked them to rate the importance of each source. In Round 2 panel members ranked each source according to those which future survey investigators should prioritise investing greatest resources to minimise. After each Round, the Steering Group reviewed and consolidated the scoring and commentary, accounting for duplication of themes and relevant omissions.

The panel of 53 were predominantly ophthalmic epidemiologists, 69.1% male, 76.4% were aged between 35 and 64 years, 55% were currently based in Europe, with 11% in North America, 9% in Latin America, 5% in each of South Asia, Southeast Asia, Australasia, and Sub-Saharan Africa, and 4% in Asia Pacific. 80% were previous investigators and between them had conducted >100 surveys of vision impairment. Panel members had worked on vision impairment or eye disease prevalence surveys In Sub-Saharan Africa (n=18 panel members), North Africa and Middle East (n=3 panel), Oceania (n=7 panel members), North America (n=5), Latin America(n=11), Caribbean (n=5), Europe (n=12), Australiasia (n=8), Southeast Asia (n=14), South Asia (n=12), East Asia (n=6), Central Asia. (n=3), and Asia Pacific (n=2).

The generic STROBE checklist items were endorsed as essential to high-quality reporting in both comprehensive and rapid surveys. The panel identified several STROBE items that were frequently poorly applied in the reporting of eye health surveys, and several vision specific extensions to the items. Additionally, the group highlighted a need for specific guidance to enhance the standardisation and quality of future surveys. Areas of concern included transparent reporting of random selection processes for participants, survey response rate estimation and reporting, variable quality of vision measurement, case definitions for common eye diseases and 'avoidable vision loss', and reporting crude data without appropriate adjustment to account for complex survey sampling designs. Differences between the panels' expectations for comprehensive versus rapid surveys were generally small, with most panellists recommending equivalent rigour of design, conduct, analysis and reporting in both, even though rapid surveys utilise a more swift, simple, and low-cost examination protocol.

Disability weights for vision impairment

Supplementary Table 14: Summary of studies estimating a disability weight for blindness and vision impairment

Year Reference	Region	Panel composition	Panel size	Health states considered	Valuation methods	Vision category	Disability Weight (95% CI)	Construct
1994[125]	Global	Independent experts	NS	6	Magnitude estimation	Blind	0.6	Disability
1996[126]	Global	Medical experts	10	483	PTO and VAS	Blind	0.6 (0.50 – 0.70)	Disability
1997[127]	Netherlands	Medical experts	38	175	PTO and VAS	Blind	0.43 (0.34 -0.52)	Disability
2002[128]	Burkina Faso	Lay people, health workers	39 lay people,<br>17 health workers	9	Culturally adapted VAS	Blind	0.36 (SD 0.10)	Disability
2009[129]	Estonia	Medical experts	25	283	PTO and VAS	Blind<br>VI	0.478<br>0.028	Disability
2012[130]	Global	Population-based samples	30,230	220	PC and PHE	Blind<br>SVI<br>MVI<br>Mild VI<br>Near VI	0.195 (0.132-0.272)<br>0.191 (0.129-0.269)<br>0.033 (0.020-0.052)<br>0.004 (0.001-0.010)<br>0.013 (0.006-0.024)	Health loss
2013[131]	Global	Based on EQ5D regression analysis of GBD 2010 weights	NS	220	Regression analysis	Blind<br>SVI<br>MVI<br>Mild VI<br>Near VI	0.338<br>0.314<br>0.089<br>0.005<br>0.047	Loss of health and functioning
2015[132]	Europe: Sweden, Italy, Netherlands, Hungary	Population (quota sampling of internet panels, population representative, 18-65 years)	30,660	255	PC and PHE	Blind<br>SVI<br>MVI<br>Mild VI<br>Near VI	0.173 (0.145 -0.213)<br>0.158 (0.13-0.193)<br>0.034 (0.027-0.042)<br>0.004 (0.002-0.005)<br>0.012 (0.008-0.015)	Health loss
2015[133]	Global	Population (combined data)[128,129]	60,890	183 or 235	PC	Blind<br>SVI<br>MVI<br>Mild VI<br>Near VI	0.187 (0.124 - 0.260)<br>0.184 (0.125-0.258)<br>0.031 (0.019-0.049)<br>0.003 (0.001-0.007)<br>0.011 (0.005-0.020)	Health loss

GBD = Global Burden of Disease, PC = Paired comparison, NS= Not specified, PHE = Population Health Equivalence, PTO = person trade off, VAS = visual analogue scale, VI = vision impairment, SVI = severe vision impairment, MVI = moderate vision impairment,

Section 4: Economics of vision

Systematic review of the economics of vision impairment and eye health

Forthcoming manuscript: Ana Patricia Marques, Jacqueline Ramke, John Cairns, Thomas Butt, Justine H. Zhang, Hannah Faal, Hugh Taylor, Iain Jones, Nathan Congdon, Andrew Bastawrous, Tasanee Braithwaite, Marty Jovic, Serge Resnikoff, Allyala Nandakumar, Debbie Muirhead, Peng T. Khaw, Rupert Bourne, Iris Gordon, Kevin Frick, Matthew J. Burton
Manuscript Under Review, 2020

Summary of methods

The protocol for this review has been published, and a summary of methods is provided here.[134]

A literature search was performed in MEDLINE (Ovid) and the CRD database (Centre for Reviews and Dissemination) which includes the National Health Service Economics Evaluation Database (NHS EED), Database of Abstracts of Reviews of Effects (DARE) and the Health Technology Assessment (HTA) database. The search strategy combined key words related to:

- Vision impairment (VI), blindness, uncorrected refractive error, cataract, age related macular degeneration (AMD), glaucoma, diabetic retinopathy, corneal opacity and trachoma; and
- economic terms such as cost of illness, cost and costs analysis, cost effectiveness and cost benefit and global burden of disease.

No language or geographical restriction was applied although the search was restricted to papers published from 1 January 2000 up to December 2019. The inclusion criteria are summarized and complemented with PICOS details in the table below**Error! Reference source not found.**.

Summary of the PICOS elements for the systematic review

Participants	Participants with VI from an unspecified cause or due to one of the leading causes of VI globally (i.e. cataract, uncorrected refractive error, diabetic retinopathy, glaucoma, AMD, corneal opacity and trachoma)
Interventions	Any report that provides information about costs of illness, burden of diseases and /or loss of well-being, estimates of the impact of VI on labour market outcomes (e.g. employment chances, labour market participation rate), informal care (e.g. number of caregiver hours) or in terms of well-being (e.g. Quality Adjusted Life Years [QALYs] and Disability Adjusted Life Years [DALYs]) in participants with VI was included.
Comparators	Not relevant
Outcomes	Direct costs, indirect costs, productivity losses (e.g absenteeism costs, lost work days, employment chances), informal care (e.g. caregivers costs, number of caregivers hours), intangible costs [e.g. QALYs, DALYs], transfer payments or deadweight losses.
Study Design	Partial economic evaluation studies such as cost of illness studies, burden of illness/diseases and full economic evaluation studies such as cost-effectiveness and cost-benefit studies were included. Model-based economic evaluation studies not reporting any costs primary data or based on reviews of existing studies were excluded.

All titles and abstracts were screened by two investigators independently in Covidence systematic review software. After completing the screening process, full texts were assessed by two investigators independently to establish eligibility for inclusion into the study. Any conflict in relation to screening and appraisal was discussed between the two investigators and resolved with a third investigator when necessary. A detailed description about the systematic review protocol is available in the published protocol. The PRISMA flow diagram outlines the search process and the reasons for study exclusion.

APM extracted all relevant data which was then verified by one of TB, JZ or JR. The items extracted included:

- Study details: study period, country/ countries of study, age range of participants, study design (e.g. cost of illness, burden of illness/diseases cost effectiveness or cost benefit studies);
- Methodological details: epidemiological approach (i.e. incidence or prevalence based), perspective of analysis (e.g. societal/ government/ healthcare system/ payer/ healthcare provider or patient), method of resource quantification (e.g. top-down, bottom-up, combination), discounting methods (i.e. discount rate applied and justification);
- Data and definitions: main data sources (e.g. published expenditures report, administrative database, population survey, patient clinical records, patient diaries, specially designed questionnaires, published literature), VI definition & severity (i.e. blind/ moderate or severe VI), cause of VI (and definition) if specified, disease stage if specified, currency in which costs were reported, year of cost data, cost components (e.g. direct costs, productivity costs,

informal care costs), loss of well-being measures (e.g. intangible costs measured with QALYs, DALYs, years of sight loss);

- Analysis of uncertainty (e.g. type of uncertainty analysed (parameter uncertainty, methodological uncertainty or modelling uncertainty), choice of parameters included in sensitivity analysis, univariate sensitivity analysis, probabilistic sensitivity analysis).

All included studies were appraised by two investigators (APM, TB, JC, IJ, MJ, DM, AM) using the British Medical Journal Checklist[135] for economic submissions adapted for cost of illness studies.[136]

PRISMA flow diagram for systematic review of the economics of vision impairment and eye health

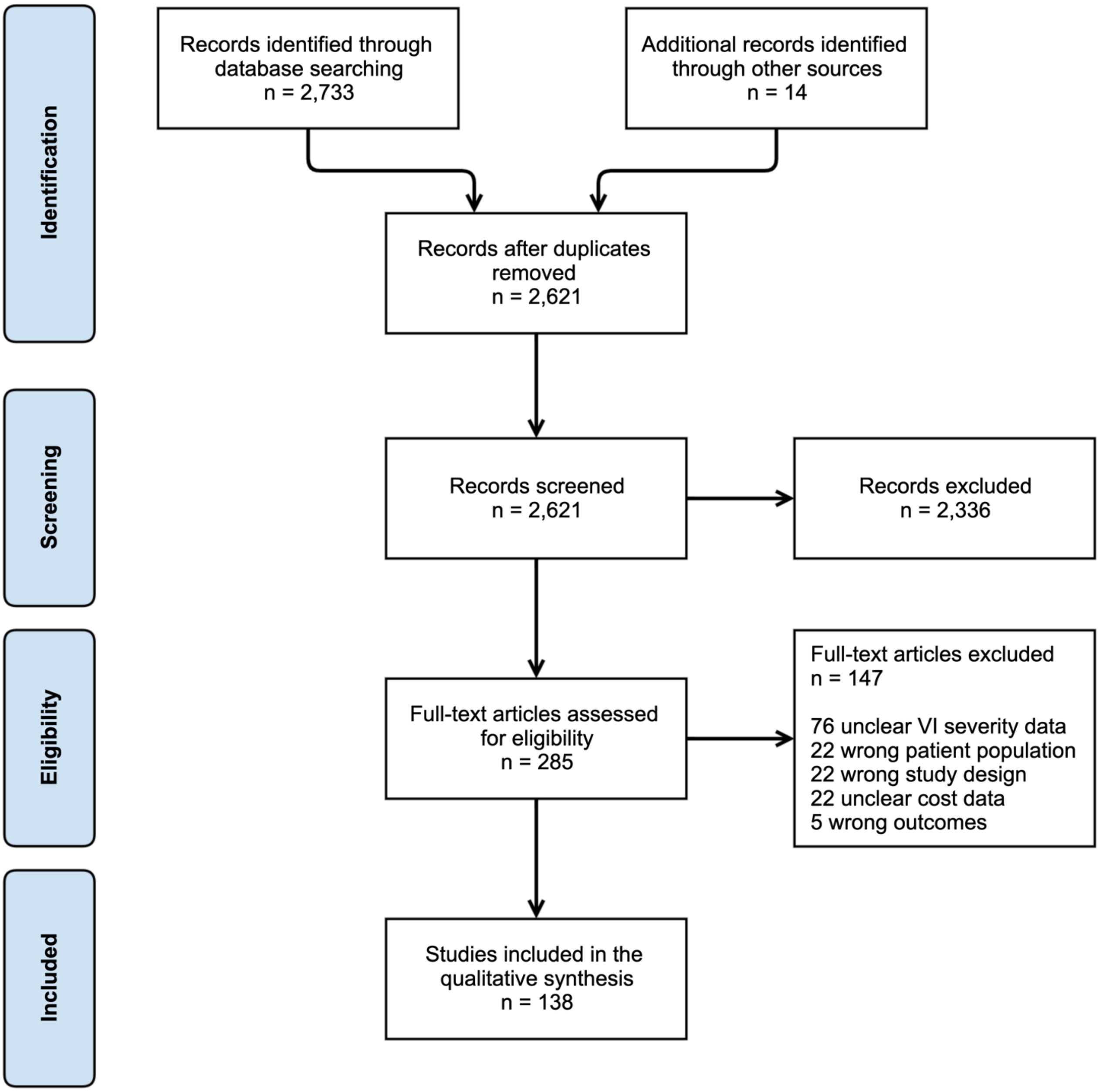

Description of the studies

A total of 2733 studies were identified from the search and 14 additional grey literature studies referenced by field experts. After the titles, abstracts and full text were screened, 138 studies remained.

A total of 38 studies reported data for VI and/or blindness (designated as general studies).[137-172]

A total of 100 studies reported condition specific data (designated as condition specific studies).[173-272] The conditions included: for uncorrected refractive error, cataracts, age macular degeneration, glaucoma, diabetic retinopathy, corneal opacity and trachoma.

The geographical distribution of general and condition specific reports are shown in Supplementary Table 15. Several studies published more than one related report. Global data were presented in 14 reports and there are 134 distinct regional reports.

Nine of the 21 GDB Regions had no reports. Five studies reported data from more than one GDB Super Region[137,139,187,210,258], seven studies report data from more than one GDB Region.[137,139,187,246,247,258,273] In total, 96/134 (72%) of the regional estimates provide data from the GBD High Income Super Region.

Of the 100 studies reporting disease-specific costs, several reported estimates for more than one region or condition. Overall, these 100 studies provide 105 regional condition-specific estimates as follows: 34 studies reported costs for AMD, 32 studies for cataract, 16 studies for glaucoma, 11 studies for uncorrected refractive error, 6 studies for diabetic retinopathy, 5 studies for trachoma and 1 study for corneal opacity (Supplementary Table 15).

Many studies were not comprehensive in approach; the majority (97/138; 70%) only considered one category of cost (from direct health care costs, direct health non care costs, productivity loss, informal care, intangible costs), 25 studies (18%) considered two and only 16 (12%) considered three or more.

Supplementary Table 15: Health economic studies by GBD Super Region and condition

	Southeast Asia, East Asia, Oceania	South Asia	Central Europe, Eastern Europe, Central Asia	North Africa Middle East	Sub-Saharan Africa	Latin America Caribbean	High Income	Global Studies	Total
**General studies**	3	2	1	0	1	1	30	5	**43**
**URE**	0	1	0	1	1	0	4	4	**11**
**Cataract**	4	4	1	0	4	3	15	1	**32**
**Glaucoma**	0	1	0	0	1	2	12	0	**16**
**AMD**	1	0	1	1	0	1	30	0	**34**
**DR**	0	1	0	0	0	0	5	0	**6**
**Corneal opacity**	0	1	0	0	0	0	0	0	**1**
**Trachoma**	0	0	0	0	1	0	0	4	**5**
**Total**	**8**	**10**	**3**	**2**	**8**	**7**	**96**	**14**	**148**

NOTE: Several studies report data from multiple regions. We record the different regions separately, therefore, the total of region-specific reports exceeds the total number of studies in the systematic review.

No. of studies
0
1
2-3
4-9
10-19
20-29
30+

Supplementary Table 16: Economic studies for general vision impairment and specific conditions.

Studies were assessed for perspective, epidemiological approach, design, reported costs, and approaches to quantify resources and uncertainty. Some studies assessed more than one type of cost.

	General studies (n=38)		Condition specific studies (n=100)		Total (n=138)	
	**n**	**%**	**n**	**%**	**n**	**%**
**Study perspective**						
Societal	21	55.3%	27	27.0%	48	34.8%
Healthcare system	4	10.5%	21	21.0%	25	18.1%
Payer	1	2.6%	22	22.0%	23	16.7%
Patient	7	18.4%	7	7.0%	14	10.1%
Hospital	0	0%	7	7.0%	7	5.1%
Governmental	4	10.5%	0	0%	4	2.9%
Caregiver	0	0%	1	1.0%	1	0.7%
Employers	0	0%	1	1.0%	1	0.7%
Multiple	0	0%	11	11.0%	11	8.0%
Not applicable	1	2.6%	3	3.0%	4	2.9%
**Study epidemiological approach**						
Prevalence based	34	89.5%	90	90.0%	124	89.9%
Incidence based	3	7.9%	5	5.0%	8	5.8%
Incidence and prevalence based	0	0%	3	3.0%	3	2.2%
Not applicable	1	2.6%	2	2.0%	3	2.2%
**Study design**						
Cost of illness study	28	73.7%	56	56.0%	84	60.9%
Cost analysis	4	10.5%	24	24.0%	28	20.3%
Cost effectiveness study	0	0.0%	17	17.0%	17	12.3%
Others	6	15.8%	3	6.0%	9	6.5%
**Type of reported costs**						
Direct Costs	28	39.4%	87	66.0%	115	56.9%
Productivity losses	19	26.8%	18	14.0%	37	18.3%
Informal care	14	19.7%	16	12.0%	30	14.9%
Intangible costs	10	14.1%	10	8.0%	20	9.9%
**Method use to assess uncertainty**						
Sensitivity analysis	13	34.2%	28	28.0%	41	29.7%
None	25	65.8%	72	72.0%	97	70.3%

Supplementary Table 17: Comparison of global productivity losses from vision impairment.

	Marques 2020 (Commission Report)	Gordois 2012[168]	Bastawrous 2019[172]	Frick 2003[170]
Definition of vision impairment	Blindness is defined as presenting distance visual acuity <3/60 in the better eye and MSVI as presenting distance visual acuity of <6/18 to 3/60 in the better eye.	Blindness was defined as visual acuity <6/60 and moderate VI as visual acuity <6/18 to 6/60	Blindness was defined as presenting visual acuity <20/400 in the better-seeing eye; MSVI was defined as presenting visual acuity <20/60 to 20/400 in the better-seeing eye	Blindness was defined as visual acuity less than 3/60 in the better eye; Low vision is defined as visual acuity less than <6/18 to 3/60 in the better eye.
Year of cost data	2018	2010	2017	2000
Prevalence data source	Crude prevalence and 95% uncertainty intervals for blindness and MSVI in people within working age, by GBD region. Data source: these data were specifically calculated by GBD Vision Loss Expert Group for 2020 for this analysis.[88]	Prevalence data using WHO global VI estimates.[274,275]	Prevalence data for blindness and MSVI by country were obtained from the GBD Vision Loss Expert Group 2015 estimates.[102] It was assumed that 40% and 50% of the blind and MSVI population were of working age respectively.	Prevalence data for Blindness and low vision by World Development region and age group were obtained for 1995.[276]
Regions of report	Global	WHO ´developed` regions AMR-A, EUR-A, EUR-B1, EUR-B2, EUR-C, WPR-A1, and WPR-A2. It was assumed that only in developed countries a loss in productivity due to VI is expected to represent a real cost to an economy	Global	Global
Working age population	15 to 64 years	15 to 64 years	15 to 64 years	15 to 64 years
Probability of Employment in the general population	It is assumed that someone with is just as likely to have been employed in the absence of their VI condition. This likelihood is measured using the employment to population ratio.	It was assumed that someone with VI is just as likely to be employed in the absence of their VI condition. This likelihood was expressed using employment to population ratio	Not included	Labour force participation rate and unemployment rate were used to calculate probability of employment
Relative reduction in employment due to vision impairment	Under-unemployment in people with VI was derived from a literature review. Reports from 15 countries and regions were used to estimate under-employment in people with VI by GBD region using a weighted average (total population used as weight)	Under - unemployment in people with VI was derived from a literature review. Reports from 5 countries was used to extrapolate data for each subregion.	Under - unemployment in people with VI was assumed to be equal to 50% for people with blindness or MSVI	Under - unemployment in people with VI was assumed to be equal to disability weight: 60% for people with blindness and 24.5% for people with VI
Value of production loss equal to	GDP & GNI per capita, USD ppp	GDP per capita	GNI per capita	GDP per capita
Productivity losses components included estimates	Reduced employment participation	Reduced employment participation and premature mortality	Reduced employment participation and reduced wages (based on USA data only)	Reduced employment participation
Productivity loss estimate Converted to 2018 USD ppp	Annual Global productivity losses were estimated to be 410.7 billion USD ppp (range $322.1 - $518.7 billion) in GDP model and $408.5 billion (range $320.4 - $515.9 billion) in GNI model	Annual Global productivity losses from blindness and moderate VI in the 7 ´developed` WHO regions was estimated at 193.36 billion USD PPP including 1 billion due to premature mortality	Global productivity losses from blindness and VI around 381 billion USD PPP in 2020. Estimated to rise cumulatively to 19.4 trillion USD PPP in 2050.	Annual Global productivity losses ranged from 26.8 billion USD ppp (for blindness) to 59.4 billion USD ppp of productivity loss (blindness and low vision)

VI = Vision Impairment; MSVI = Moderate to Severe Vision Impairment; GBD = Global Burden Disease; WHO= World Health Organization;; GDP = Gross Domestic Product; GNI = Gross National Income; USD ppp = United Stated Dollar purchasing power parity.

Global productivity losses from vision impairment in 2020

Summary of methods

In this study we aimed to estimate the cost of potential lost productivity due to blindness and moderate and severe vision impairment (MSVI) across the 21 Global Burden of Diseases (GBD) regions.

We combined the most recent economic, demographic and prevalence data on vision impairment and blindness to estimate the cost of potential productivity losses due to reduced employment participation among people with vision loss. Calculations were limited to the working age population (15-64 years) and were presented in 2018 US Dollar purchasing power parity (ppp). Two separate models – using Gross Domestic Product (GDP) and Gross National Income (GNI) - were calculated to enable comparability with previous estimates.[137,139,168,172,180]

The annual cost of potential productivity loss was calculated for each region as the product of prevalence of blindness and MSVI, the population employment ratio, the relative reduction in employment for people with vision impairment or blindness and GDP/GNI.

Data sources for each of these is outlined below.

1. Prevalence data

The number of people aged 15-64 years with blindness (presenting visual acuity <3/60 in the better eye) or MSVI (presenting visual acuity of <6/18 to 3/60 in the better eye) (crude prevalence and 95% uncertainty lower and upper intervals data supplied by the GBD Vision Loss Expert Group, data presented and described elsewhere.[88]

2. **Employment to population ratio**:

This represents the proportion of a country´s population that is likely employed. Data were sourced from the World Bank´s World Development Indicator database for 2018 (or for the most recent year available)[277]

3. **Relative reduction in employment**:

For people with MSVI or blindness compared to people without vision loss. An estimate of the relative reduction in employment for people with vision impairment was identified through a literature search. We sought studies or reports from any country published since the year 2000 that reported the employment status and/or employment gap in people with vision impairment.

We identified 11 published studies[144,152,157,159,162,168,222,226,259,278-284] that provided employment gap data for 15 countries or regions which we categorised into 8 GBD regions and 3 super region (Supplementary Table 17). The employment gap for each region and super region was calculated as the weighted average employment gap (with the total population of each country being the weight) of the countries included in each region or super region that reported data.

When estimating productivity losses by region we used the super region average whenever there was no data for a specific region. If there were no data for either region or super region the average of all super region was used.

4. GDP/GNI

The valuation of the annual production loss for people unemployed due to MSVI or blindness was assumed equal to GDP per capita (model 1) or GNI per capita (model 2). Data were sourced from the World Bank´s World Development Indicator database in 2018 US Dollar PPP for 2018 or the most recent year for which data were available:

- Gross Domestic Product (GDP) (sum of gross value added by all resident producers in the economy) per capita [285]
- Gross National Income (GNI) (sum of value added by all resident producers plus net receipts of primary income [compensation of employees and property income] from abroad) per capita[286].

Supplementary Table 18: Relative reduction in employment among people with VI (%)

GDB Super Regions and Regions	Relative reduction in employment in people with VI (%)
**High Income**	**32.1**
High-income Asia Pacific	26.7
Australasia	32.4
Western Europe	20.6
Southern Latin America	No data
High-income North America	43.5
**Central Europe, Eastern Europe, and Central Asia**	**22.5**
Central Asia	22.5
Central Europe	22.5
Eastern Europe	22.5
**Latin America and Caribbean**	No data
Caribbean	No data
Andean Latin America	No data
Central Latin America	No data
Tropical Latin America	No data
**North Africa, Middle East**	No data
**South Asia**	No data
**Southeast Asia, East Asia, and Oceania**	No data
Asia Southeast	No data
East Asia	No data
Oceania	No data
**Sub-Saharan Africa**	**28.9**
Central Sub-Saharan Africa	No data
Eastern Sub-Saharan Africa	No data
Southern Sub-Saharan Africa	No data
Western Sub-Saharan Africa	28.9
**Number of countries**	**15***
**Average**	**30.2**

* In addition, one study reported data for WHO regions

The weighted average of relative reduction in employment for each region was calculated as a weighted average of relative reduction in employment in each country included in each region, with the total population of each country being the weight.[285] It was calculated as the quotient between:

Relative reduction of employment per region = Weighted average of Relative Reduction in employment (RR employment) of the countries included in each region that reported data

$$\frac{\sum_{n=nr\ countries}(RR\ employment_{\ country\ a}\ x\ Tot.pop_{country\ a})+(RR\ employment_{country\ b}\ x\ Tot.pop_{country\ b})+\cdots\left(RR\ employment_{\ country\ n}\ x\ Tot\ Pop_{Country\ n}\right)}{\sum_{n=nr\ countries} Tot.Pop_{country\ a}+Tot.Pop_{\ country\ b}+\cdots Tot.Pop_{Country\ n}}$$

GDP per capita PPP and GNI per capita PPP for each region were calculated as a weighted average of GDP or GNI per capita of each country included in each region, with the total population of each country being the weight.[285] The weighted average of GDP per capita PPP of all the countries included in each region was calculated as the quotient between:

1. Sum of the product of (GDP per capita of each country and the total population of each country in the region); and
2. Sum of the total population of each country in the region

GDP per capita per region = Weighted Average of GDP per capita PPP of all the countries included in each region

$$=\frac{\sum_{n=nr\ countries}(GDP\ pc_{country\ a}\ x\ Tot.pop_{country\ a})+(GDP\ pc_{country\ b}\ x\ Tot.pop_{country\ b})+\cdots(GDP\ pc_{country\ n}\ x\ Tot\ Pop_{Country\ n})}{\sum_{n=nr\ countries} Tot.Pop_{country\ a}+Tot.Pop_{\ country\ b}+\cdots Tot.Pop_{Country\ n}}$$

Tot.Pop designates Total Population; n= number of countries included in each region; pc=per capita

The weighted average of GNI per capita PPP was calculated in the same way.

There was no data for GDP or GNI per capita PPP in Cuba, Korea Dem. People's Republic, Syrian Arab Republic, Djibouti, Somalia and Andorra, therefore these countries were not included in this calculation.

The weighted average of employment to population ratio for each region was calculated using the same approach as described previously for weighted GDP per capita PPP adapted as follows:

$$\frac{\sum_{n=nr\ countries} (Empl.ratio\ x\ Tot.pop_{count\ a}) + (Empl\ ratio\ _{count\ b}\ x\ Tot.pop_{count\ b}) + \cdots\ (Empl\ ratio\ _{count\ n}\ x\ Tot\ Pop_{Count\ n})}{\sum_{n=nr\ countries\ in\ each\ subregion} Tot.Pop_{country\ a} +\ Tot.Pop\ _{country\ b} + \cdots Tot.Pop_{Country\ n}}$$

Tot.Pop designates Total Population; n= number of countries included in each subregion; Empl. ratio=Employment to population ratio

Employment data were unavailable for Seychelles, Antiqua and Barbuda, Dominica, Grenada, Syrian Arab Republic, Kiribati, Marshall Islands, Micronesia, Djibouti and Andorra and therefore these countries were not included in this weighted average.

Productivity losses by region were reported in billion 2018 USD ppp and as a percentage of GDP, PPP (current international $).[286] GDP PPP per region was calculated the sum of GDP, PPP of the countries included in each GBD region.

Cost-effectiveness of interventions for cataract and refractive error

Summary of methods

The aim of this analysis was to quantify the cost-effectiveness of cataract surgery and refractive error treatment globally.

Data sources and study selection

Articles were identified from the systematic literature search (2000 onwards) outlined above, whereby the title and abstract (when available) was searched for the terms *cataract* or *refractive error* combined with *cost-effectiveness* or *cost utility*. The full text of studies that were potentially eligible were assessed against the inclusion and exclusion criteria. References of studies that met the inclusion criteria were reviewed to identify additional relevant studies.

Selection criteria

We included studies that reported health benefits in Quality Adjusted Life Years (QALYS) gained or Disability Adjusted Life Years (DALYS) averted and analysed cost-effectiveness in terms of:

- Cataract: cataract surgical procedures in the first eye [such as extracapsular cataract extraction (ECCE), manual small incision cataract surgery (MSICS) or phacoemulsification (PHACO)] and
- Refractive error: any treatment.

We excluded studies that reported health benefits without using QALYs or DALYs. We also excluded studies that reported cost-effectiveness for bilateral cataract surgery, intra-capsular cataract extraction, or inserting an intraocular lens.

Data analysis and results

Cost transformation was undertaken, whereby costs were inflated to 2018 values using a country specific Gross Domestic Product (GDP) deflator [287] and converted to USD purchasing power parities [288] (ppp). When the year of cost data was not available, the year of publication was used as the year from which to inflate costs. Two studies reported global data by region using 2000 USD ppp. In these cases we used a US GDP deflator across all regions to convert 2000 USD ppp to 2018 USD ppp. Two review studies reported country data using 2004 USD and another study presented results for India using 2007 USD. In these cases we also used US GDP deflator across all countries to convert 2004 USD to 2018 USD ppp.

<u>Average</u> Cost Effectiveness Ratios (CER) reported in the same study, for the same country or region and for the same type of treatment were grouped together if the CERs were:

- for the same region but for different refractive error screening alternatives [289,290] *OR*
- in the same surgical intervention but for different coverage rates [211] *OR*
- in the same country showing discounted and undiscounted values [291] *OR*
- in the same type of cataract surgery and same discount methods [292] *OR*
- in the same country, same type of cataract surgery and 2 different discount methods.[293]

Results were presented separately for refractive error and cataract, with CERs ordered from lowest to highest minimum values.

Reflections on the included studies

182 studies were identified in the initial screening and 16 met our inclusion criteria. We excluded 3 reviews because they only reported studies already referenced by other included studies. Therefore, we included 13 studies that provided 124 CERs for cataract surgery and 89 for refractive error.[193,211,289-299]

The wide range of CERs shown in Figures 13 and 14 suggests regional variation in resource utilization, costs and patient characteristics and heterogeneity in cost effectiveness study methodology, particularly on the cost side.

Studies reported by the same group of authors such as Baltussen et al [211,289] and Lansingh [292,293] estimated health benefits and used discounting methods in a similar way. Baltussen et al [211,289] followed WHO–CHOICE guidelines and measured health benefits using DALYs averted and discounted both costs and benefit at a 3% rate for a period of 10 years. Lansingh et al [292,293] used QALYs to measure health benefits and discounted costs and benefits at 3%: 1) over 12 years [293]; 2) over 5 years [293] and 3) over the lifespan of patients [292].

Fewer similarities were found across the rest of the studies. Sources of variability include the use of different discounts rates [298-300], the use of different health related quality of life measures [297-299], the measurement of health benefit in different time periods [291,293], and the assumed duration of the health benefits.[193,292,294]

Studies varied in terms of the cost components included. Some included healthcare costs related to the cataract surgery [296,298] or refractive error [290] while others included: cost of cataract surgery complications [193,294]; costs to the provider and to the programme administration[211,289]; costs to the patient and provider [291]; personal social service costs in addition to primary and secondary health care costs [299]. Costs reported by the two reviews [292,293] included in this analysis were also heterogeneous despite attempts at standardisation.

There is always heterogeneity when comparing cost-effectiveness studies from different settings due, for example, to different healthcare system organization and prices or due to the adoption of different analytical perspectives. Even within the same setting and for the same intervention the adoption of different methodologies such as different perspectives and different choice of comparators (when reporting incremental cost effectiveness analysis) heterogeneity exists. Therefore, comparisons should always be made with caution.

Supplementary Table 19: Cost effectiveness ratios for cataract surgery (presented in Figure 13)

Reference	Intervention	Country/Region	Min	Max
Lansingh et al [292]	**Outcome:** QALYs gained<br>**Approach:** Minimum and maximum value are obtain from studies in the same country, same type of surgery and same discounting method<br>**Intervention:** SICS	Nepal		4.57
		India	6.00	6.26
	**Intervention:** Cataract surgery (not further specified)	India	4.83	45.78
		Canada	207.77	367.68
		China	330.77	838.92
		Sweden	377.07	831.35
		Spain	425.20	582.24
		United Kingdom	467.59	883.14
		Singapore	737.58	883.00
		Australia	1,055.56	1,361.42
		United States	1,386.20	1,768.75
		Nepal		5.87
		Ethiopia		18.0
		Kenya		32.61
		Uganda		36.39
		Nigeria		47.08
		Brazil		79.43
		New Zealand		604.41
		Denmark		664.54
		Finland		1,305.33
	**Intervention:** ECCE	Malaysia		228.64
		India	5.74	6.78
	**Intervention** : Phaco	India		11.48
		Nepal		46.56
		Malaysia		309.12
Lansingh et al [293]	**Outcome:** QALYs gained<br>**Approach:** Minimum and maximum value are obtain from studies in the same country, same type of surgery and 2 different discounting method<br>**Intervention** : ECCE	Tanzania	10.17	108.52
		India	11.45	138.25
		Malaysia	346.94	7,885.74
	**Intervention**: SICS	India	11.35	144.78
	**Intervention** : PHACO	India	20.22	216.51
		Malaysia	413.46	9,800.44
	**Intervention** : Cataract surgery (not further specified)	Brazil	193.04	2,069.91
		Canada	319.55	3,217.68
	**Intervention**: Outpatient	Germany	753.88	15,688.01
		France	858.22	13,271.16
Baltussen et al [211]	**Intervention:** ECCE	Southeast Asia D	76.56	88.26
	**Outcome:** DALYs averted<br>**Approach:** Minimum and maximum value are obtain from 3 levels of population coverage	Southeast Asia B	83.00	85.97
		African Region D	126.67	152.15
		Eastern Mediterranean D	140.21	152.55
		African Region E	150.29	178.13
		Eastern Mediterranean B	164.68	237.01
		West Pacific B	169.09	222.52
		America B	191.28	266.93
		America D	197.08	235.66
		Europe C	219.47	260.23
		Europe B	527.91	658.58
		America A	1,028.80	1,217.80
		Europe A	1,837.07	2,397.94
		West Pacific A	3,267.74	4,376.56
Khan et al [291]	**Outcome:** QALYs gain<br>**Intervention**: SICS	India		94.81
	**Intervention**: PHACO	India		142.49
Griffiths et al [295]	**Intervention**: Cataract surgery (not further specified)<br>**Outcome:** QALYs gain<br>**Approach:** Deterministic analysis	Zambia		517.92
Hiratsuka et al [296]	**Intervention**: Cataract surgery **Outcome:** QALYs gain	Japan		1,200.72

Reference	Intervention	Country/Region	Min	Max
Brown et al [193]	**Intervention**: Cataract surgery **Outcome:** QALYs gain	United States		1,809.61
Busbee et al [294]	**Intervention**: PHACO and ECCE **Outcome:** QALYs gain	United States		3,115.76
Kobelt et al [297]	**Intervention**: Cataract surgery<br>**Outcome:** QALYs gain<br>**Approach:** Minimum and maximum value are obtain from undiscounted and discounted values	Sweden	5,701.99	6,579.22
Rasanen et al [298]	**Intervention**: Cataract surgery **Outcome:** QALYs gain	Finland		11,988.52
Sach et al [299]	**Intervention**: Cataract surgery<br>**Outcome:** QALYs gain	United Kingdom		24,782.71

Supplementary Table 20: Cost effectiveness ratios for refractive error services (presented in Figure 14)

Reference	Details	Country/Region	Min	Max
Baltussen et al [289]	**Intervention**: Screening and treating RE in school children<br>**Outcome:** DALYs averted<br>**Approach**: Minimum and maximum value obtained from 6 alternative screening strategies	Southeast Asia D	94.89	184.11
		West Pacific B	144.46	199.69
		Eastern Mediterranean D	145.87	327.16
		Southeast Asia B	151.54	229.43
		America D	191.19	386.64
		Europe B	209.61	379.56
		African Region E	220.94	522.60
		African Region D	233.68	627.40
		Europe C	243.60	505.60
		Eastern Mediterranean B	250.68	651.48
		America B	252.09	365.39
		Europe A	648.65	1039.53
		America A	767.61	984.30
		West Pacific A	987.13	2127.22
Frick et al [290]	**Intervention:** Screening and treating RE in urban school children<br>**Outcome:** DALYs averted<br>**Approach**: Minimum and maximum value obtained from 2 alternative screening strategies	Urban India	264.23	686.29
		Rural India	367.05	1,447.89
Griffiths et al [295]	**Intervention:** Adults RE/Presbyopia correction **Outcome:** QALYs<br>**Approach:** Deterministic analysis	Zambia		752.79

Section 5: Global eye health research

Twenty years of eye health research

The aim of this analysis was to summarise the nature and extent of peer-reviewed publishing on the leading causes of vision impairment over the 20 years since Vision 2020 was launched. This will be presented in a forthcoming publication.

Summary of methods

Sample frame

The sample was constructed by an information specialist on MEDLINE in April 2020 and is shown in Box 1. We included any primary study on an eye related topic, which could include animal studies and basic laboratory studies. We excluded case reports, editorials, comments, and evidence syntheses (e.g. narrative or systematic review).

**Box 1: Summary of search to construct sample**	
1	exp eye diseases/
2	limit 1 to yr=2000 – 2019
3	case reports/
4	2 not 3
5	limit 4 to journal article
6	limit 5 to (meta analysis or "review" or "systematic review" or systematic reviews as topic)
7	5 not 6

Data preparation and analysis

Main eye condition

The list of MeSH headings and sub-headings under ‘explode eye disease’ were used to categorise each record to one of the following eight categories: 1) cataract 2) refractive error 3) glaucoma 4) AMD 5) diabetic retinopathy 6) corneal opacity 7) trachoma 8) other. The following criteria were used:

1. If a paper only has MeSH terms for one of the categories, code it to that category;
2. If a paper has MeSH terms from more than one of the categories, it was assigned to the category that has the greater number of terms. E.g. if a paper has 3 trachoma terms and 1 glaucoma term, it was assigned to trachoma;
3. If a paper has equal # of MeSH terms from 2 or more categories, it was assigned to the condition that causes the higher magnitude of blindness (ordered from 1 to 7 above);
4. If a paper has no MeSH terms related to categories 1 to 7 it was coded as 'other'.

Location of study

The location of the study was determined using three approaches. The country of affiliation of the first author was extracted and separately, the name of any country in the abstract was extracted. Where only one of these was available, it was used to assign the location of the study. Where the country of affiliation differed from the country in the abstract, the country in the abstract was used. Where neither of these were available, the place of publication was used (~3% of records). Each country was assigned to the relevant region and super-region using GBD categories.

Gender of authors

The first name and surname of all authors were extracted and assigned a gender (male/female/unknown) using validated algorithm (gender-api.com).

Supplementary Table 21: Summary of the research focus and region where it was conducted, 2000 to 2019

GBD Region	Main condition of research (number of published papers)								Total		Trials	
	Cataract	Refractive Error	Glaucoma	ARM	Diabetic retinopathy	Corneal opacity	Trachoma	Other	n	%	n	%
**Central Europe, Eastern Europe, and Central Asia**	**859**	**594**	**949**	**362**	**529**	**368**	**23**	**3387**	**7071**	**4.5**	**111**	**1.7**
Central Asia	13	10	20	1	13	5	-	52	114	0.1	4	0.1
Central Europe	556	354	647	246	392	243	12	2320	4770	3.0	83	1.3
Eastern Europe	290	230	282	115	124	120	11	1015	2187	1.4	24	0.4
**High-income**	**9069**	**9308**	**12313**	**8246**	**7275**	**6263**	**260**	**57346**	**110080**	**70.1**	**4492**	**70.8**
Australasia	434	626	555	360	392	261	44	1992	4664	3.0	178	2.8
High-income Asia Pacific	1434	1430	2329	1118	1382	944	8	8811	17456	11.1	443	7.0
High-income North America	3234	3037	4646	3248	2548	2376	89	21036	40214	25.6	1787	28.2
Southern Latin America	56	28	53	12	38	47	5	338	577	0.4	12	0.2
Western Europe	3911	4187	4730	3508	2915	2635	114	25169	47169	30.1	2072	32.6
**Latin America and Caribbean**	**354**	**262**	**353**	**125**	**276**	**292**	**31**	**2153**	**3846**	**2.5**	**183**	**2.9**
Andean Latin America	19	7	12	4	16	15	-	107	180	0.1	9	0.1
Caribbean	19	9	27	10	15	12	-	119	211	0.1	3	0.0
Central Latin America	74	73	47	28	87	45	4	479	837	0.5	28	0.4
Tropical Latin America	242	173	267	83	158	220	27	1448	2618	1.7	143	2.3
**North Africa and Middle East**	**853**	**841**	**843**	**310**	**684**	**762**	**64**	**5235**	**9592**	**6.1**	**524**	**8.3**
**South Asia**	**1206**	**522**	**824**	**174**	**557**	**591**	**45**	**2805**	**6724**	**4.3**	**417**	**6.6**
**Southeast Asia, East Asia, and Oceania**	**2017**	**2113**	**1693**	**714**	**1486**	**976**	**51**	**8291**	**17341**	**11.0**	**512**	**8.1**
East Asia	1859	2003	1596	688	1377	906	28	7750	16207	10.3	447	7.0
Oceania	5	6	-	1	8	8	13	34	75	0.0	1	0.0
Southeast Asia	153	104	97	25	101	62	10	507	1059	0.7	64	1.0
**Sub-Saharan Africa**	**273**	**184**	**244**	**28**	**88**	**67**	**367**	**1049**	**2300**	**1.5**	**109**	**1.7**
Central Sub-Saharan Africa	14	4	6	1	7	7	4	68	111	0.1	1	0.0
Eastern Sub-Saharan Africa	78	27	46	1	21	17	224	193	607	0.4	43	0.7
Southern Sub-Saharan Africa	34	55	30	7	13	11	1	159	310	0.2	13	0.2
Western Sub-Saharan Africa	147	98	162	19	47	32	138	629	1272	0.8	52	0.8
**Grand Total**	**14631**	**13824**	**17219**	**9959**	**10895**	**9319**	**841**	**80266**	**156954**	**100.0**	**6348**	100.0
(row %)	(9.3)	(8.8)	(11.0)	(6.3)	(6.9)	(5.9)	(0.5)	(51.1)			(4.0)	

Analysis of randomised controlled trials on eye health conducted in Sub-Saharan Africa

Forthcoming publication: Nyawira Mwangi, Shaffi Mdala, Ada Aghaji, Stephen Gichuhi, Kwesi Amissah-Arthur, Abdull Mohammed, Elizabeth Kishiki, Fiston Kitema, Heiko Phillipin, Simon Arunga, Rebecca Oenga, Lily Kimetto, Robert Geneau, Faith Masila, Hilary Rono, Jacqueline Ramke, Matthew J Burton, Fatima Kyari, Hannah Faal, Esmael Habtamu

Sub-Saharan Africa is home to 13.5% of the global population, but only 1.7% of eye health related clinical trials have been conducted in this region since 2000. Given the magnitude vision impairment in Sub-Saharan Africa is disproportionately high, the Commission undertook an in-depth review of RCTs conducted in Sub-Saharan Africa over the past 20 years

Summary of methods

The search was constructed by the Cochrane Eyes and Vision Information Specialist outlined above (Twenty years of eye research) was used for this analysis. An algorithm was created to identify RCTs and studies done in Sub-Saharan Africa within the larger dataset from the title, abstract and MeSH terms. In addition, reference lists of relevant Cochrane reviews were searched, and a wider group of global eye health research experts were contacted to review the list and identify any other potentially relevant studies.

The title and abstract of potentially relevant papers were screened. Duplicates, publications that were not RCTs and those not from Sub-Saharan Africa were identified and removed.

In total, 123 publications were identified as reporting results of a trial (main trial paper) or associated findings of the trial. For example, bigger projects such as the Partnership for Rapid Elimination of Trachoma (PRET) had multiple secondary reports on trachoma but also other nested studies which looked at the impact of Azithromycin on non-eye health conditions such as malaria, bacterial infections, and growth and nutrition. For this analysis, we excluded these secondary reports, leaving 86 trials across 89 reports (some trials were in several countries, and the study was published separately for each country).

The research team undertook data extraction, including:

- *study and publication characteristics:* journal and year of publication, country of trial, registration, funding source, and study period;
- *main trial methodological characteristics*: condition of interest, participants, sample size, trial intervention and primary outcome;
- *author information*: first and surname and affiliations (country, city, institution), position in authorship list (first/last/other).

A second member of the research team checked for completeness and quality of extraction.

Where possible, gender was allocated based on the author being known to a member of the research team. A validated algorithm was used to assign gender to any remaining names (gender-api.com). Affiliations were categorised as being from the country of study, international, or both. For results reported here, we assigned individuals with both local and international affiliations to one of these based on where they did their undergraduate training.

Analysis was conducted using Microsoft Excel. It focused on a) condition of interest by country where the trial was conducted, b) who conducted the trial with respect to number and gender of authors and affiliations (country of study vs international), c) authorship positions for African researcher (men and women).

Results

The 86 trials (in 89 reports) were conducted in 16 countries (Map), and more than half (52, 58%) were from four countries: Nigeria (14), South Africa (14), Ghana (12) or Ethiopia (12), (Figure). There was a modest increase in the number of trials published between 2000-2009 (35) and 2010-2019 (51). The trials were usually funded by organisations outside the continent (70, 81%), almost all of which involved large funding organisations in North America and Europe. Commercial institutions contributed funding to 14 trials, only two of which did not involve other funders. Sixteen trials (19%) were conducted through local academic institution support, primarily in South Africa (5), Ghana (4) and Nigeria (4).

Supplementary Table 22: Location and topic of RCTs conducted in Sub-Saharan Africa 2000-2019

Region	Country	Trachoma	Onchocerciasis	Glaucoma	Cataract	Refractive error	DR	Other[c]	Total
Western	Nigeria	1	-	5	3	1	-	4	14
	Ghana[a]	-	7	1	-	1	-	3	12
	Cameroon	-	7	-	-	-	-	-	7
	The Gambia[b]	6	-	-	-	-	-	-	6
	Niger	3	-	-	-	-	-	-	3
	Liberia[a]	-	2	-	-	-	-	-	2
	Guinea	1	-	-	-	-	-	-	1
	Mali	1	-	-	-	-	-	-	1
Southern	South Africa	-	-	7	4	-	-	3	14
Eastern	Ethiopia	12	-	-	-	-	-	-	12
	Kenya	-	-	1	1	-	1	4	7
	Tanzania[b]	4	-	1	-	-	-	-	5
	Rwanda	-	-	-	-	-		1	1
	Uganda				1	-	-	-	1
Central	DRC[a]	-	1	1	-	-	-	-	2
	Angola	-	-	-	-	-	-	1	1
**Total**		**28**	**17**	**16**	**9**	**2**	**1**	**16**	**89**

DRC: Democratic Republic of Congo, DR: diabetic retinopathy

a multicountry study

b multicountry study

c includes pterygium (4) ophthalmia neonatorum (3), conjunctivitis (2), effect of caffeine (2), chalazion, Choroido-retinal vascular diseases, school screening, Ocular surface squamous neoplasia, open global trauma (all 1)

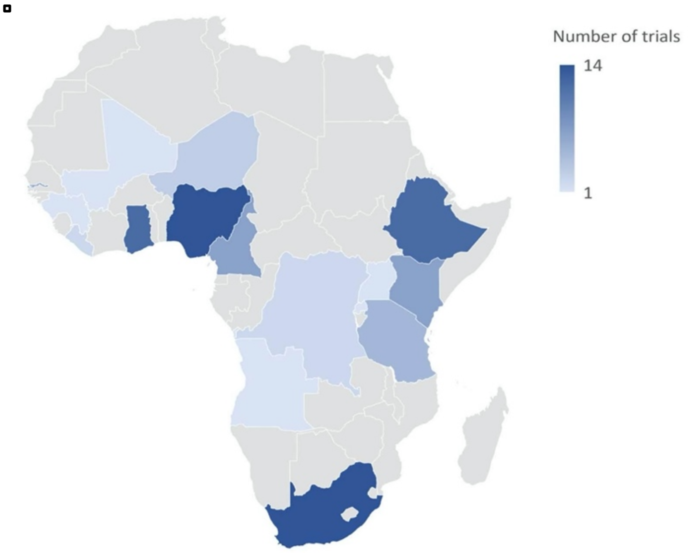

Trachoma was the most frequently studied condition (28, 33%) followed by onchocerciasis (17, 20%) and glaucoma (16, 19%). While trachoma and onchocerciasis are important conditions in their own right, there has been a relative underinvestment in other conditions and areas of study. Several lessons can be drawn from the NTD research. First, high-quality trial evidence has been vital to effectively address these conditions, shaping disease control approaches being deployed in endemic regions, leading to substantial reductions in prevalence of both Neglected Tropical Diseases (NTDs) over the last 20 years. Second, the trials were characterised by extensive collaboration between multiple well-aligned partners, including government NTD programmes. Third, the research community was relatively well-coordinated in identifying key questions and minimising unnecessary duplication.[301] Fourth, major funders have been very engaged in supporting the research needed to develop elimination strategies.

The African region faces many eye health challenges, particularly in relation to glaucoma, where early detection and appropriate management can reduce loss of vision. It is a challenge that currently does not have good solutions. There is an urgent need for systematic research to develop contextually relevant approaches to identify and manage glaucoma in Africa.

Diversity of editorial boards of ophthalmology journals

Forthcoming publication: Jacqueline Ramke, Justine H Zhang, Sare Safi, Simon Arunga, William Waldock, Nyawira Mwangi, Helen Burn, Miho Yoshizaki, Matthew J Burton

Summary of methods

Sample

On October 25 2019 the list of ophthalmology journals was downloaded from SCImajo, along with the SCImajo rank (https://www.scimagojr.com/). This list contained the name of 121 journals. Of these, three were duplicates of the same journal (listed using a previous name), four were unable to be located by google search (2 Russian journals, 2 Chinese), one had a website that could not be translated (Eastern European) and one had no editor information. We therefore report results for 112 journals.

Data extraction

Data were extracted from websites between October 25 2019 and January 31 2020.
One of the authorship team visited the website of each journal, and extracted the following data:

- *Journal*: name, publisher, region of focus (global/HIC/LMIC/not specified),
- *Editorships*: category (e.g. editor-in-chief, section editor, associate editor, editorial board member), name, affiliation (institution, city, country), availability of photo (yes/no), where a photo was available, whether the editor appeared to be male or female.

All positions with current decisive functions regarding manuscript acceptance were extracted. Advisory board members were included, but not editor emeritus or editorial staff members (e.g. managing editor, editorial assistant, copy editor, technical editor, statistical consultant).
When an editor had more than one affiliation listed, the second was extracted only if it was in a different country to the first affiliation. If the institutional country was not explicitly listed, Google Maps was used to identify the location of the institutional affiliation of the editorship. When an editor was listed with only initials rather than a first name, an internet search was undertaken using the available information (such as the affiliation) to identify the first name where available.

Data analysis

We did not de-duplicate names across journals, so if an individual sat on more than one editorial board, they were counted for each position held.
We categorised editorships into two groups:

- *Editor-in-chief* - captures editorships whose raw titles imply a leadership element, such as editor-in-chief and chief editor;
- *Editor* comprises raw titles such as editor, associate editor, section editor, member of editorial board, editorial committee, advisory board.

The list of editor names was assigned a gender by using the validated Gender-API software (https://gender-api.com/).
For any names unable to be matched, the data collected from photographs (where available) was used to assign gender.
The editorships unable to be matched that did not have a photograph were removed from the analysis.

Supplementary Table 23: Gender of editors and Editors in Chief of ophthalmology and optometry editorial boards, 2019/2020

GBD Super-Region	Editorships*		Editors in Chief**	
	Female n (%)	Total n	Female n (%)	Total n
Central Europe, Eastern Europe, and Central Asia	20 (30.3)	66	1 (33.3)	3
High Income	863 (23.6)	3660	11 (9.9)	111
Latin America and Caribbean	64 (19.4)	330	—	4
North Africa and Middle East	73 (25.3)	288	—	8
Southeast Asia, East Asia, and Oceania	84 (22.2)	378	3 (50.0)	6
South Asia	71 21.9)	324	—	2
Sub-Sharan Africa	3 (20.0)	15	—	—
**Total**	**1178 (23.3)**	**5061**	**15 (11.2)**	**134**

*a further 53 editorships had missing gender or country data. ** Where journals had >1 Editor in chief, we included all

Grand Challenges in Global Eye Health

Forthcoming publication.

Summary of methods
To identify the grand challenges in global eye health we undertook a three-round modified Delphi process. We wanted to reach people not always included in these processes, so in addition to issuing personal invitations to stakeholders known to Commissioners, we issued an invitation to nominate challenges in the Community Eye Health Journal (which has a broad readership in African countries and India) and through newsletters of ICO and WCO organisations (e.g. ARVO, IAPB, Royal College of Ophthalmology). In Round 1 we received responses from people across all GBD super-regions and a broad range of disciplines relevant to global eye health (including clinicians, decision-makers, researchers, advocates, program implementers, educators and people with lived experience of vision loss and eye problems). Ultimately 336 people completed all three Rounds.

In Round 1 participants were asked one question: '*What are the grand challenges in Global Eye Health?' Where a* **grand challenge** was defined as *a specific barrier that, if removed, would help to solve an important health problem. If successfully implemented, the intervention(s) to address this grand challenge would have a high likelihood of feasibility for scaling up and impact.*

Participants could nominate up to 5 challenges. All responses were thematically analysed and an iterative process undertaken to develop a list of 85 unique challenges to present in Round 2, where participants chose and ranked their 20 priority challenges. The 40 highest ranked challenges from Round 2 were presented in Round 3 and participants ranked each challenge against four criteria (disease burden reduction, inequality reduction, immediacy of impact and feasibility) on a four-point scale. The final list of grand challenges includes the 16 highest ranked challenges from Round 2 (which identified participants' overall priorities) and the average score of all criteria in Round 3.

Section 6: Beyond 2020 - Delivering high-quality universal eye care

Conceptualising eye health within Universal Health Coverage

Supplementary Figure 10: Universal Health Coverage

Dimensions for countries to consider when moving towards Universal Health Coverage. Source: WHO.[302]

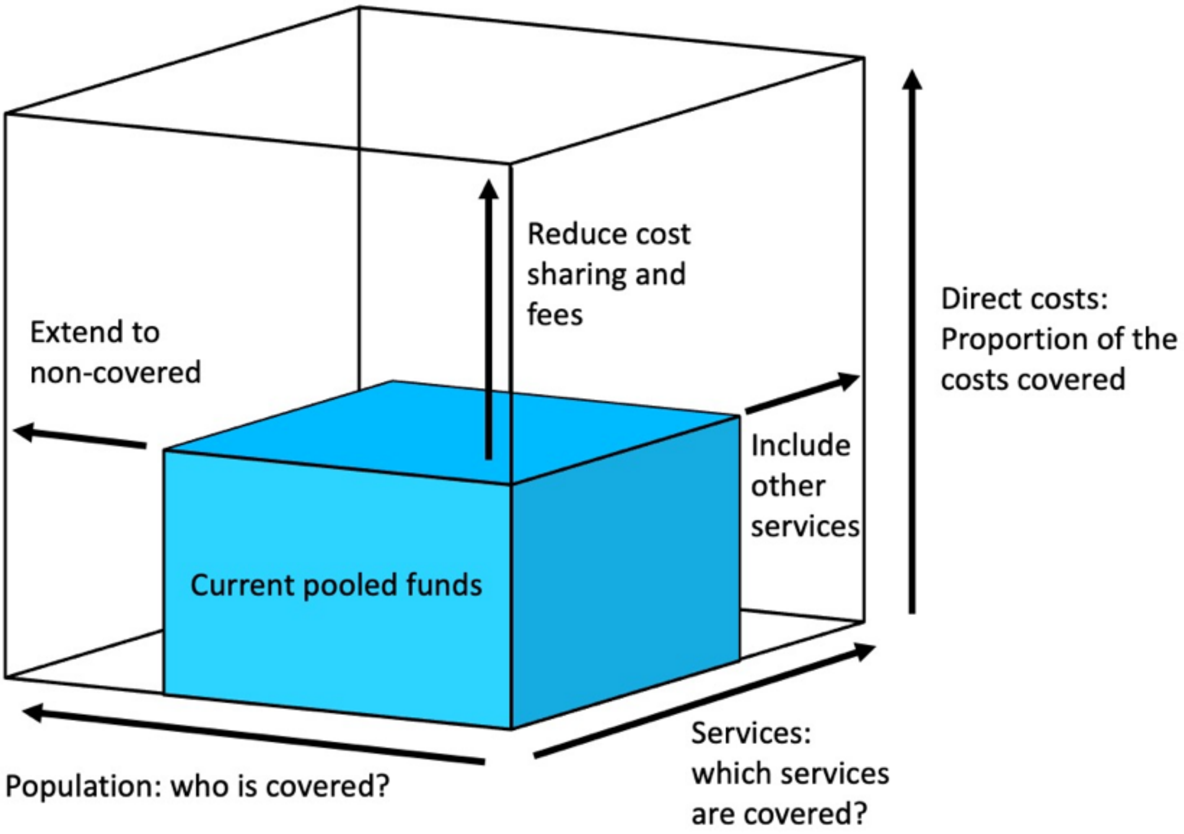

Delivering integrated people-centred eye care within UHC

Supplementary Table 24: Eye health service components relevant to low-resource, medium-resource and high-resource settings, delivery platform, human resources and integration.

Low-resource settings	Community	Primary health care	Secondary health care	Tertiary health care
**Location**	• Community setting	• Health centre	• General hospital<br>• "Stand-alone" secondary eye hospital	• Teaching hospital<br>• "Stand-alone" tertiary eye hospital
**Human resources**	• Community health worker<br>• Health volunteer<br>• School teachers	• Primary health care worker<br>• Outreach services from secondary units	• Ophthalmologist (generalists)<br>• Ophthalmic nurse / assistant<br>• Optometrist<br>• Anaesthetist<br>• Eye care manager	• Ophthalmologist (few sub-specialists)<br>• Ophthalmic nurse / assistant<br>• Optometrist<br>• Orthoptist<br>• Anaesthetist<br>• Counsellor<br>• Equipment technician<br>• Eye care manager
**Human resource development**	• Training of community leaders and volunteers	• Build capacity of community health and general primary health care workers to prevent, identify, treat, advise and refer	• Training of general health workers in eye care	• Training of general ophthalmologists and mid-level eye care workers
**Services**	• Health promotion / education<br>• Vision screening: identifying and referral of people with VI or symptomatic problems<br>• School eye health programmes<br>• Measles and rubella vaccination<br>• Identification and referral of people with vision rehabilitation needs<br><br>If needed:<br>• SAFE strategy for trachoma elimination<br>• Onchocerciasis elimination<br>• Vitamin A distribution	• Detection and referral of cataract<br>• Detection and referral of ophthalmic emergencies<br>• Detection and referral of chronic conditions causing VI<br>• Trichiasis surgery<br>• Treatment of minor conditions (conjunctivitis, foreign bodies)<br>• Basic child eye health (screening of new-borns, prevention of ophthalmia neonatorum)<br>• Non-optical vison rehabilitation services and referral of those with complex needs	• Assessment and treatment of people referred from primary care<br>• Outreach programmes to primary care<br>• Refractive error services /Management of presbyopia<br>• Cataract surgery<br>• Glaucoma medical and surgical management<br>• Diabetic retinopathy assessment and management (if laser available)<br>• Trichiasis surgery<br>• Management of ocular injuries<br>• Investigation and medical treatment of serious ocular infections<br>• Non-optical and optical vision rehabilitation services with provision of optical devices	Services provided in secondary care and:<br>• Assessment and treatment of people referred from secondary care<br>• Paediatric eye services and surgery<br>• Vitreoretinal surgery/ injections<br>• Oculoplastic surgery<br>• Uveitis management<br>• Other complex ocular surgery<br>• Ophthalmic laser treatment<br>• Advanced diagnostic investigations<br>• More advanced rehabilitation services
**Service integration opportunities**	• Vertical integration to primary health centre<br>• Health promotion / education with other health promotion activities<br>• Integration with other community-based healthcare activities (immunisation, family planning, NTD programs, rehabilitation, etc)<br>• Integration with school health programmes	• Vertical integration to secondary unit<br>• Support and supervision of community activities<br>• Eye care integrated with other health care services in the same facility delivered by same general staff<br>• Eye care for children integrated into general children's health services	• Vertical integration to tertiary unit<br>• Support and supervision of primary level services<br>• Introduce diabetic retinopathy screening into general diabetes care<br>• Integrated with the primary care level refractive error services, collaboration with the private sector	• Support and supervision of secondary level services

Medium-resource settings	Community	Primary health care	Secondary health care	Tertiary health care
**Location**	• Community setting	• Health centre<br>• Optometry practice<br>• Vision centre	• General hospital<br>• “Stand-alone” secondary eye hospital	• Teaching hospital<br>• “Stand-alone” tertiary eye hospital
**Human resources**	• Community health worker<br>• Health volunteer<br>• School teachers<br>• Associations for the blind	• Primary health care worker<br>• Mid-level ophthalmic personnel<br>• Optometrist<br>• Outreach services from secondary units	• Ophthalmologist (generalists)<br>• Ophthalmic nurse / assistant<br>• Optometrist<br>• Eye care manager	• Ophthalmologist (sub-specialists)<br>• Ophthalmic nurse / assistant<br>• Optometrist<br>• Orthoptist<br>• Anaesthetist<br>• Counsellor<br>• Equipment technician<br>• Eye care manager
**Human resource development**	• Training of community leaders and volunteers	• Build capacity of community health and general primary health care workers to identify and refer	• Training of general health workers in eye care	• Training of general and sub-specialist ophthalmologists and mid-level eye care workers
**Services**	• Health promotion / education<br>• Vision screening: identifying and referral of people with VI or symptomatic problems<br>• School eye health programmes<br>• Measles and rubella vaccination<br><br>If needed:<br>• SAFE strategy for trachoma elimination<br>• Onchocerciasis elimination<br>• Vitamin A distribution	• Refractive error services<br>• Detection and referral of cataract<br>• Detection and referral of ophthalmic emergencies<br>• Diabetic retinopathy screening services (retinal photography), ± remote grading and referral decisions (using AI or telemedicine)<br>• Detection and referral of other chronic conditions causing VI<br>• Treatment of minor conditions (conjunctivitis, foreign bodies)<br>• Basic child eye health (screening of new-borns, management of ophthalmia neonatorum)<br>• Trichiasis surgery (if needed)<br>• Non-optical and optical vison rehabilitation services and referral of complex cases	• Assessment and treatment of people referred from primary care<br>• Outreach programmes to primary care settings (where required)<br>• Specialist refractive error services<br>• Cataract surgery<br>• Glaucoma medical and surgical management<br>• Diabetic retinopathy assessment and management<br>• Ophthalmic laser treatment<br>• Management of ocular injuries<br>• Investigation and medical treatment of serious ocular infections<br>• Simple rehabilitation services<br>• Trichiasis surgery (if needed)<br>• Advanced rehabilitation services	Services provided in secondary care and:<br>• Assessment and treatment of people referred from secondary care<br>• Advanced diagnostic investigations<br>• Paediatric eye services and surgery<br>• Vitreoretinal surgery<br>• Oculoplastic services and surgery<br>• Cornea services and surgery<br>• Uveitis services<br>• Ocular oncology services<br>• Glaucoma services<br>• More advanced rehabilitation services<br>• Retinopathy of prematurity (ROP) screening and treatment<br>• Complex rehabilitation services
**Service integration opportunities**	• Vertical integration to primary health centre<br>• Health promotion / education with other health promotion activities<br>• Integration with other community-based healthcare activities (immunisation, family planning, rehabilitation etc)<br>• Integration with school health programmes<br>• Care of the elderly<br>• Occupational health	• Vertical integration to secondary unit<br>• Support and supervision of community activities<br>• Eye care integrated with other health care services in the same facility delivered by same general staff<br>• Eye care for Children integrated into general children’s health services<br>• Diabetic retinopathy screening services integrated into general diabetes care	• Vertical integration to tertiary unit<br>• Support and supervision of primary level services<br>• Diabetic retinopathy screening services integrated with general diabetes care<br>• Integrated with the primary care level refractive error services, collaboration with the private sector	• Support and supervision of secondary level services<br>• Integration of ROP screening and treatment into neonatal services

High-resource settings	Community	Primary health care	Secondary health care	Tertiary health care
**Location**	• Community setting	• Optometry practice<br>• Vision centre<br>• Ophthalmology practice<br>• General practice health centre	• General hospital<br>• "Stand-alone" secondary eye hospital	• Teaching hospital<br>• "Stand-alone" tertiary eye hospital
**Human resources**	• Community health workers<br>• Health visitors<br>• Associations for the blind<br>• Alternative routes of communication for health messages.	• Optometrist<br>• General practitioner<br>• DR screening technician	• Ophthalmologist (generalists)<br>• Ophthalmic nurse / assistant<br>• Optometrist<br>• Eye care manager<br>• Rehabilitation specialist	• Ophthalmologist (sub-specialists)<br>• Ophthalmic nurse / assistant<br>• Optometrist<br>• Orthoptist<br>• Anaesthetist<br>• Counsellor<br>• Equipment technician<br>• Eye care manager
**Human resource development**	• Training of community leaders and volunteers	• Build capacity of community health and general primary health care workers to identify and refer	• Training of general health workers in eye care	• Training of general and sub-specialist ophthalmologists and mid-level eye care workers
**Services**	• Health promotion / education<br>• Vision screening: identifying and referral of people with VI or symptomatic problems<br>• School eye health programmes<br>• Measles and rubella vaccination<br>• Non-optical vision rehabilitation services and referral<br><br>If needed:<br>• SAFE strategy for trachoma elimination	• Refractive error services<br>• Detection and referral of cataract<br>• Detection and referral of ophthalmic emergencies<br>• Diabetic retinopathy screening services (retinal photography), ± remote grading and referral decisions (using AI or telemedicine)<br>• Detection and referral of other chronic conditions causing VI<br>• Treatment of minor conditions<br>• Co-management of conditions between primary and secondary care<br>• Basic child eye health (screening of new-borns, management of ophthalmia neonatorum)<br>• Non-optical and optical vision rehabilitation services, with referral of complex cases	• Assessment and treatment of people referred from primary care<br>• Outreach programmes to primary care settings (where required)<br>• Specialist refractive error services<br>• Cataract surgery<br>• Glaucoma medical and surgical management<br>• Diabetic retinopathy assessment and management<br>• Ophthalmic laser treatment<br>• Management of ocular injuries<br>• Investigation and medical treatment of serious ocular infections<br>• Advanced rehabilitation services	Services provided in secondary care and:<br>• Assessment and treatment of people referred from secondary care<br>• Advanced diagnostic investigations<br>• Paediatric eye services and surgery<br>• Vitreoretinal surgery<br>• Oculoplastic services and surgery<br>• Cornea services and surgery<br>• Uveitis services<br>• Ocular oncology services<br>• Glaucoma services<br>• Retinopathy of prematurity (ROP) screening and treatment<br>• More advanced rehabilitation services<br>• Complex rehabilitation services
**Service integration opportunities**	• Vertical integration to primary health centre<br>• Health promotion / education with other health promotion activities.<br>• Integration with school health programmes.<br>• Care of the elderly<br>• Occupational health	• Vertical integration to secondary unit<br>• Support and supervision of community activities<br>• PEC integrated with other health care services in the same facility delivered by same general staff<br>• Diabetic retinopathy screening services integrated with general diabetes care.<br>• Basic children's eye care integrated into general children's health services<br>• Vision rehabilitation services integrated with other rehabilitation programmes	• Vertical integration to tertiary unit<br>• Support and supervision of primary level services<br>• Diabetic retinopathy screening services integrated with general diabetes care<br>• Integrated with the primary care level refractive error services, collaboration with the private sector	• Support and supervision of secondary level services<br>• Integration of ROP screening and treatment into neonatal services

Human resources for eye health: building the eye health team

Supplementary Table 25: The eye health team

The main clinical cadres, their scope of work, resource requirement and integration within the health system. Terminology varies between countries and regions.

Cadre	Role and integration
Ophthalmologist	Doctors who have gone through extensive higher training in ophthalmology, including the diagnosis and advanced management of eye disease and ophthalmic surgery. Usually based in secondary or tertiary eye units, where most eye surgery is performed. Require a large array of complex diagnostic and treatment equipment to perform role.
Optometrist	Have typically undergone four years of training in refraction and carrying out comprehensive eye examination. Provide refractive services and opportunistic screening for non-refractive causes of poor vision. They can work independently practice or work in eye hospitals as part of the team.
Cataract surgeon	Several countries with a shortage of ophthalmologists have trained experienced allied ophthalmic personnel to perform some ophthalmic surgical procedures. They tend to be based in secondary eye units and require diagnostic and treatment equipment to perform role. Ideally, they work under the supportive supervision of an ophthalmologist.
Orthoptist	Have typically undergone three years of training in ocular movement assessment, refraction, visual field assessment and common eye conditions in children. They have particular expertise in assessing vision in children, and in managing strabismus refractive error and amblyopia in children. They usually work in eye hospitals as part of the team.
Refractionist	Trained in assessing common refractive errors. They often work in optical shops or as part of an eye care team in an eye hospital.
Dispensing opticians	Trained to dispense spectacles from a prescription, including frame fitting and lens cutting and centration
Dispensing opticians	Trained to dispense spectacles from a prescription, including frame fitting and lens cutting and centration
Ophthalmic nurse	Have typically undergone specialised training in ophthalmic nursing on a background of a general nursing qualification. They have particular expertise in the nursing care (out-patient, in-patient and / or operating theatre) of eye patients. Some are trained in the diagnosis and management of common eye conditions. They usually work in eye hospitals as part of the team. In some settings they perform a limited set of surgical procedures.
General health workers	These vary from a general physician to a community health worker. Their training and expertise in managing health conditions depends on their cadre. Similarly, their expertise in managing eye health conditions depends on their cadre and the training they have received about eye diseases. Ideally all health workers should receive training in the promotion of eye health and prevention of eye diseases as well as the assessment of a patient with eye symptoms and their appropriate management.
Vision rehabilitation worker	After suitable training, vision rehabilitation can be provided by ophthalmologists, optometrists, rehabilitation workers and ophthalmic nurses depending on complexity.
Community health worker	Based in the community, provide health education / promotion; support activities such as screening and distribution of treatments.
Others	Counsellors, equipment technicians, managers, administrators with skills in data management, anaesthetists

Supplementary Figure 11: Ophthalmologists per million population, by world region

Box and whisker plots: midlines are the median values, the boxes represent the interquartile range, the whiskers the upper and lower adjacent values. Outliers are plotted as individual dots. Data source: Resnikoff et al.[303]

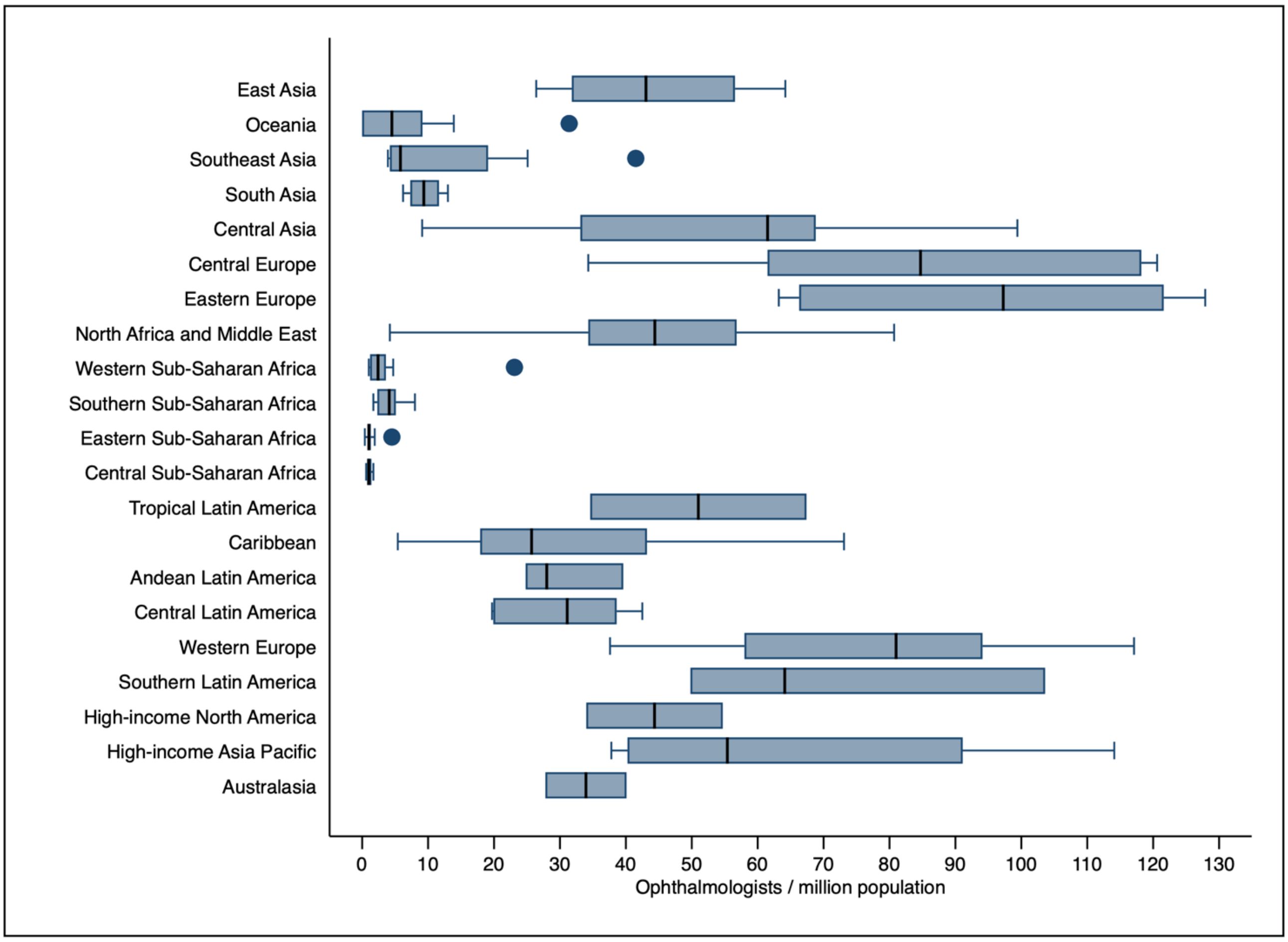

Supplementary Table 26: Vision impairment and the eye health workforce

Data used to prepare Commission Report Figure 18

A) Age-standardised prevalence of blindness (all ages) for the 21 GBD Regions, by the number of ophthalmologists per million population. The area of the bubble is proportional to the number of people who are blind.
Data sources: Resnikoff et al, World Council of Optometry and GBD.VLEG 2020 data.[303,304]

GBD Region	Number of ophthalmologists /million population	Age-standardised prevalence of blindness (%)	People with blindness
Andean Latin America	33.3	0.60	349,273
Australasia	38.1	0.15	68,866
Caribbean	63.2	0.50	259,591
Central Asia	50.5	0.41	300,965
Central Europe	96.0	0.17	327,353
Central Latin America	36.1	0.51	1,265,059
Central Sub-Saharan Africa	1.1	0.49	287,423
East Asia	27.5	0.47	9,085,478
Eastern Europe	88.0	0.24	789,619
Eastern Sub-Saharan Africa	1.1	1.07	1,971,468
High Income Asia Pacific	99.1	0.15	535,124
High Income North America	52.6	0.12	711,990
North Africa and Middle East	37.6	0.70	3,092,627
Oceania	5.0	0.55	39,341
South Asia	12.5	0.90	11,940,894
Southeast Asia	9.8	1.00	5,952,746
Southern Latin America	86.6	0.19	158,312
Southern Sub-Saharan Africa	4.4	0.82	477,384
Tropical Latin America	66.3	0.74	1,784,286
Western Europe	88.4	0.18	1,533,753
Western Sub-Saharan Africa	2.9	1.11	2,348,442

B) Age-standardised prevalence of vision impairment (mild, moderate, severe and blind; all ages) for the 21 GBD Regions, by the number of ophthalmologists and optometrists per million population. The area of the bubble is proportional to the number of people who have vision impairment.

Data sources: Resnikoff et al, World Council of Optometry and GBD.VLEG 2020 data.[303,304]

GBD Region	Number of ophthalmologists and optometrists /million population	Age-standardised prevalence of vision impairment (%)	People with vision impairment
Andean Latin America	86.4	8.67	3,109,914
Australasia	264.0	3.33	820,844
Caribbean	73.7	7.02	1,817,441
Central Asia	50.5	6.63	3,259,016
Central Europe	130.1	3.70	4,293,441
Central Latin America	97.7	7.93	11,134,131
Central Sub-Saharan Africa	1.1	7.21	2,303,906
East Asia	28.9	6.63	63,211,556
Eastern Europe	101.3	5.89	11,894,062
Eastern Sub-Saharan Africa	5.0	7.79	8,992,883
High Income Asia Pacific	358.5	5.26	5,937,088
High Income North America	171.7	2.85	8,175,584
North Africa and Middle East	54.8	7.69	24,960,988
Oceania	8.9	9.13	425,215
South Asia	46.5	11.16	108,215,295
Southeast Asia	36.7	10.42	34,748,688
Southern Latin America	105.4	4.74	2,285,379
Southern Sub-Saharan Africa	55.9	6.81	2,044,464
Tropical Latin America	82.7	8.73	12,104,965
Western Europe	354.1	4.28	16,985,325
Western Sub-Saharan Africa	12.6	9.56	12,225,795

Innovating Delivery: technology to support eye health within UHC

Artificial Intelligence solutions for eye health: a scoping review

**Title:**	**AI solutions for eye health: a scoping review**
Objective:	We aimed to answer the following questions:<br>1. Which AI solutions are currently, or potentially soon available to eye health care practitioners, or eye health care services?<br>(and for each unique AI solution identified):<br>2. What specific problem or condition do the AI solutions address? (i.e.: DR; OCT image segmentation; patient appointments)<br>3. Which area of eye care does the solution focus on? (i.e.: screening, diagnosis, treatment)<br>4. To what extent are these AI solutions regulated and what levels of regulation, approval or permissions for practice do they hold?<br>5. Have any explicit considerations of equity, inclusion and human rights been made in the development or implementation of the AI solutions?
Search date:	13 January 2020
Search databases:	MEDLINE, Embase, clinicaltrials.gov, WHO ICTRP
Key definitions:	*AI solution*: refers to any technology using automated analysis, through machine learning or deep learning algorithms. We use the terms AI "solution, tool, intervention" interchangeably in this review<br>*AI solution for eye health*: refers to AI technology with potential for being used in people by health care practitioners or in eye health care services<br>*AI solution that is "in use or soon in use":* this includes any AI solutions that have undergone external validation or are beyond that stage in the translation path.<br>*External validation*: we defined external validation as the process of testing the AI system on an independent set of data different to that used for the development, internal validation (training and testing) and evaluation of the tool.
Key inclusion/ exclusion criteria:	Included:<br>• Primary studies conducted in humans, of any design, reporting on AI solutions for eye health (preventive, diagnostic or treatment services), that are at external validation stage or beyond in the translation pathway<br>• There will be no restriction based on language or geographical location; studies must be published since 2015.<br>Excluded:<br>• Animal or laboratory studies<br>• Studies reporting AI solutions that have no application in eye care<br>• Reviews, editorials, opinion pieces, conference abstracts<br>• Studies reporting AI solutions that are at development stage
Number of included studies:	We initially identified 1256 primary data reports from which we extracted some data, and ultimately included 113 reports on external validation or successful deployment in clinical settings.
Protocol registration:	Full protocol registered on OSF: https://osf.io/94qt2/

Citation for The Commission's review to identify publicly available datasets of ophthalmic images

Khan SM, Liu X, Nath S, Korot E, Faes L, Wagner SK, Keane PA, Sebire NJ, Burton MJ, Denniston AK. A global review of publicly available datasets for ophthalmological imaging: barriers to access, usability, and generalisability Lancet Digital Health Published: October 01, 2020; DOI: https://doi.org/10.1016/S2589-7500(20)30240-5

Sustainable financing for eye health

ODA Data Analysis

The Organisation for Economic Co-operation and Development's (OECD) Development Assistance Committee (DAC) has a number of online databases that cover bilateral, multilateral aid (ODA) and private providers' aid and other resource flows to developing countries. The Creditor Reporting System (CRS) and the CRS Microdata provide detailed information on individual aid activities, such as sectors, countries and project descriptions.

The OECD DAC CRS database (available at https://stats.oecd.org/) was accessed 19 February 2020. The CRS Microdata was searched for terms related to eye health, using the following terms: blind, blindness, braille, cataract, cornea, corneal, eye, eyecare, eyeglass, eyeglasses, eyehealth, glasses, glaucoma, hyperopia, macular, myopia, myopic, ocular, onchocerciasis, ophthalmic, ophthalmology, ophthalmologist, optic, optical, optician, optrometrist, optrometry, presbyopia, refractive, retina, retinopathy, river blindness, sight, sightedness, spectacles, trachoma, vision, ,visual, visually. The data was filtered to include gross disbursements of aid, in constant 2017 US$, for each year between 2014-2018. Duplicates were removed, adjustments made where visual impairments were one of multiple outcomes, and line items found to be unrelated to visual impairment and blindness were deleted. Between 2014-2018, the annual average aid to eye health was $102 million (range $78-$131 million). The annual estimate of total eye health aid as a percentage of total ODA was then calculated – between 2014-2018 the annual average was 0.056% (range 0.048%-0.069%).

An estimated 66% of the total eye health aid between 2014-2018 was directed at the elimination of blinding neglected tropical diseases, trachoma and onchocerciasis. Other vision related areas that received aid included: inclusive education and visual learning aides; training and income generating opportunities; the building and maintenance of vision centres and eye health hospitals; cataract interventions; and support to organisations representing people who are visually impaired or blind.

Measuring progress in eye health within UHC

Indicator prioritisation for eye health within UHC

Forthcoming publication:
Eye health indicators for universal health coverage: results of a global expert prioritisation process
McCormick I, Mactaggart I, Resnikoff S, Muirhead D, Murthy GV, Silva JC, Bastawrous A, Sterne J, Blanchet K, Wang N, Yusufu M, Cooper A, Gichangi M, Burton MJ, Ramke J on behalf of the Eye Health Indicators Prioritisation Project Group. *Br J Ophthalmol, 2021,* In press

In order to contribute to the global conversation around the selection of suitable indicators, beyond the eCSC and eREC highlighted by WHO, the Commission initiated a two round indicators prioritisation project. The panel consisted of 72 eye health experts from 39 countries, with good representation from all GBD Super Regions (85% representing LMICs) and 40% female.

The panel worked to develop a proposed menu of indicators (core and extended) to monitor eye health and eye health services at the national level, to support governments in their progress towards achieving UHC. In Round 1, the panel scored potential indicators according to priority for their setting. In Round 2, indicators above the median Round 1 priority score were scored on four further criteria: feasibility, actionability, repeatability and international comparability. Core indicators were defined as those that all countries could ideally report for use at national and global levels.

The final menu included 22 indicators and is presented in Supplementary Table 27. These represent key concepts across the results chain, with seven selected as core indicators for monitoring progress towards UHC (Commission Report Table 6). Sub-indicators, drawn from the prioritisation process, were included where additional articulation of key concepts was required. Input (governance, finance, infrastructure, supply chain, information), output (access, quality and safety), outcome (coverage) and impact (improved outcomes) domains were all represented in the final selection.

An equity statement accompanies the menu and all indicators should be disaggregated by key equity dimensions. We anticipate that this menu and future metadata descriptions could be used to inform monitoring and evaluation frameworks for governments and eye health programmes. In addition, existing gaps in indicator availability need to be addressed, for example, around conditions such as glaucoma and diabetic retinopathy. Indicator metadata should also be developed collaboratively to describe preferred methods of data collection, reporting and usage.

Supplementary Table 27: Indicators for eye health within UHC

Full list of 22 indicators identified by the indicator prioritisation panel.

Equity Statement

All indicators summarising population-based and eye care facility-based data should report metrics disaggregated by key equity dimensions of gender, place of residence, socioeconomic position and disability status, where available. Additional options, such ethnicity or marital status, can be recorded by countries as appropriate.

Inputs & processes

Governance

G1 Eye health is integrated into the national health strategy/ plan (or the relevant specific plan e.g. NCD plan)

- G1.1 National health plan includes human resources for eye care (Y/N)
- G1.2 Eye health is integrated into the plans, policies and budget of other initiatives such as:
  - G1.2.1 national essential package of health services (Y/N)
  - G1.2.2 primary health care (Y/N)
  - G1.2.3 maternal and child health care (Y/N)
  - G1.2.4 diabetes care (Y/N)
  - G1.2.5 school health programmes (Y/N)
  - G1.2.6 healthy aging programmes (Y/N)
- G1.3 National eye health policies, plans and programmes refer to a multisectoral approach/ engagement with other sectors (Y/N)
  - *If a national eye health strategy/ plan is unavailable or not up-to-date, record as N*

G2 Is the national eye health plan informed by recent evidence (Y/N):

- G2.1 Time since cited population-based data was collected (in months/years)
- G2.2 Time since cited ESCAT/situational analysis data was collected (in months/years)

Finance

F1 Eye health is integrated into the national health budget (Y/N)

- *Requires a working group to develop sub-indicators and metadata*

F2 Eye health is included in national health finance pooling mechanism (Y/N)

- *Scaled response based on scoring outcomes of sub-indicators in 'checklist'*

If yes, the range/number/list of services addressing leading causes of VI included:

- F2.1 Out-patient consultation (Full/Partial/No)
- F2.2 Cataract (Full/Partial/No)
- F2.3 Refraction services (Full/Partial/No)
- F2.4 Glaucoma medication/surgery (Full/Partial/No)
- F2.5 Diabetic retinopathy – laser/anti-VEGF (Full/Partial/No)

F3 Proportion of population covered via national health finance pooling mechanisms that includes eye care services:

- F3.1 Proportion covered for: Out-patient consultation
- F3.2 Proportion covered for: Cataract
- F3.3 Proportion covered for: Refraction services
- F3.4 Proportion covered for: Glaucoma medication/surgery
- F3.5 Proportion covered for: Diabetic retinopathy – laser/anti-VEGF

Infrastructure

I1 Eye health facility density and distribution, disaggregated by:

- I1.1 Primary
- I1.2 Secondary
- I1.3 Tertiary
- I1.4 Low vision services
  - *By PoR (urban/rural), total numbers (public and private) per million population*
  - *Additional sub-national administrative or geographic divisions as relevant to setting*
- *Additional dimension: Access to PEC and cataract surgery via GPS and geospatial modelling*

I2 Percentage of neonatal units providing screening for retinopathy of prematurity nationally

Supply chain

SC1 Pharmaceuticals specifically for eye care on the National Essential Medicines List

- *Total number and proportion compared with a normative standard for eye health pharmaceuticals (e.g. WHO or IAPB list)*

Information

INFO1 Existence of a National Health Information System that includes eye care service data (Y/N)

Eye health workforce

HR1 Eye health worker density and distribution, disaggregated by:

- HR1.1 Ophthalmologist
- HR1.2 Optometrist
- HR1.3 Ophthalmic nurse
- HR1.4 Other allied ophthalmic personnel
  - *By PoR (urban/rural), total number per million population, and by age groups and sex*
  - *Additional sub-national administrative or geographic divisions as relevant to setting*
  - *Additional dimension: 5-year trends per cadre*

HR2 Is Primary Eye Care integrated into the national Primary Health Care training (if applicable)? (Y/N)

Outputs

Access

AC1 Cataract surgical rate

- *Total number per million population and including variation in rate across urban/rural or districts*

• *Additional dimension: 5-year trend in CSR*
**Quality & safety**
**Q1** Cataract surgical outcome (visual acuity)<br>• *Proportion of eyes with a 'good' outcome (6/18 or better)*<br>• *Proportion of eyes with a 'poor' outcome (worse than 6/60)*
**Q2** Priority eye conditions with quality of care/clinical practice guidelines endorsed by relevant regulatory bodies<br>• Q2.1 Cataract (Y/N)<br>• Q2.2 Refractive error (Y/N)<br>• Q2.3 Glaucoma (Y/N)<br>• Q2.4 Age-related macular degeneration (Y/N)<br>• Q2.5 Diabetic retinopathy (Y/N)<br>• Q2.6 Child eye health (Y/N)
**Responsiveness/affordability**
**AF1** Median (range) of out of pocket payment made for cataract surgery as a proportion of median monthly household (or individual) income<br>• *Report median and mean payment made at point of service (excluding transport, accommodation, sustenance)*<br>• *Disaggregated by provider type (government/public, private for profit, private NGO/charity)*<br>• *Additional dimension: proportion reported for poorest versus wealthiest quintiles*
**Outcomes**
**Coverage**
**C1** Cataract surgical coverage & effective cataract surgical coverage<br>• *CSC, eCSC, 'quality gap' reported, disaggregated by age, gender, SEP, PoR as available*
**C2** Refractive error coverage & effective refractive error coverage<br>• *REC, eREC, 'quality gap' reported, disaggregated by age, gender, SEP, PoR as available*
**C3** Coverage of diabetic retinopathy screening of all people with diabetes (at the frequency recommended in national guidelines)<br>• *Requires a working group to develop complete indicator metadata*<br>• *Disaggregated by age, gender, SEP, PoR as available*
**C4** Coverage of school eye health programmes for schools nationally<br>• *Proportion of schools receiving screening in the past 12 months*<br>• *Disaggregated by primary and secondary schools*
**Impact**
**Improved outcomes**
**P1** Prevalence of vision impairment (VI)<br>• P1.1 Distance VI prevalence, by WHO categories<br>• P1.2 Near VI prevalence, by WHO definition<br>○ *from population-based surveys, disaggregated by age, gender, SEP, PoR as available*
**P2** Cause-specific prevalence of vision impairment<br>• *Prevalence of vision-impairing priority eye conditions from population-based surveys, disaggregated by age, gender, SEP, PoR as available*<br>• P2.1 Avoidable blindness/SVI/MVI/mild VI prevalence disaggregated by age, gender, SEP, PoR as available<br>○ *aggregated from VI causes assigned in surveys*
**P3** Prevalence of childhood vision impairment and blindness<br>• *Blindness/SVI/MVI/mild VI from population-based or key-informant surveys, disaggregated by age, gender, SEP, PoR as available*

Supplementary Figure 12: Cascade for measuring effective coverage of health care services

Adapted from Marsh et al.[305]

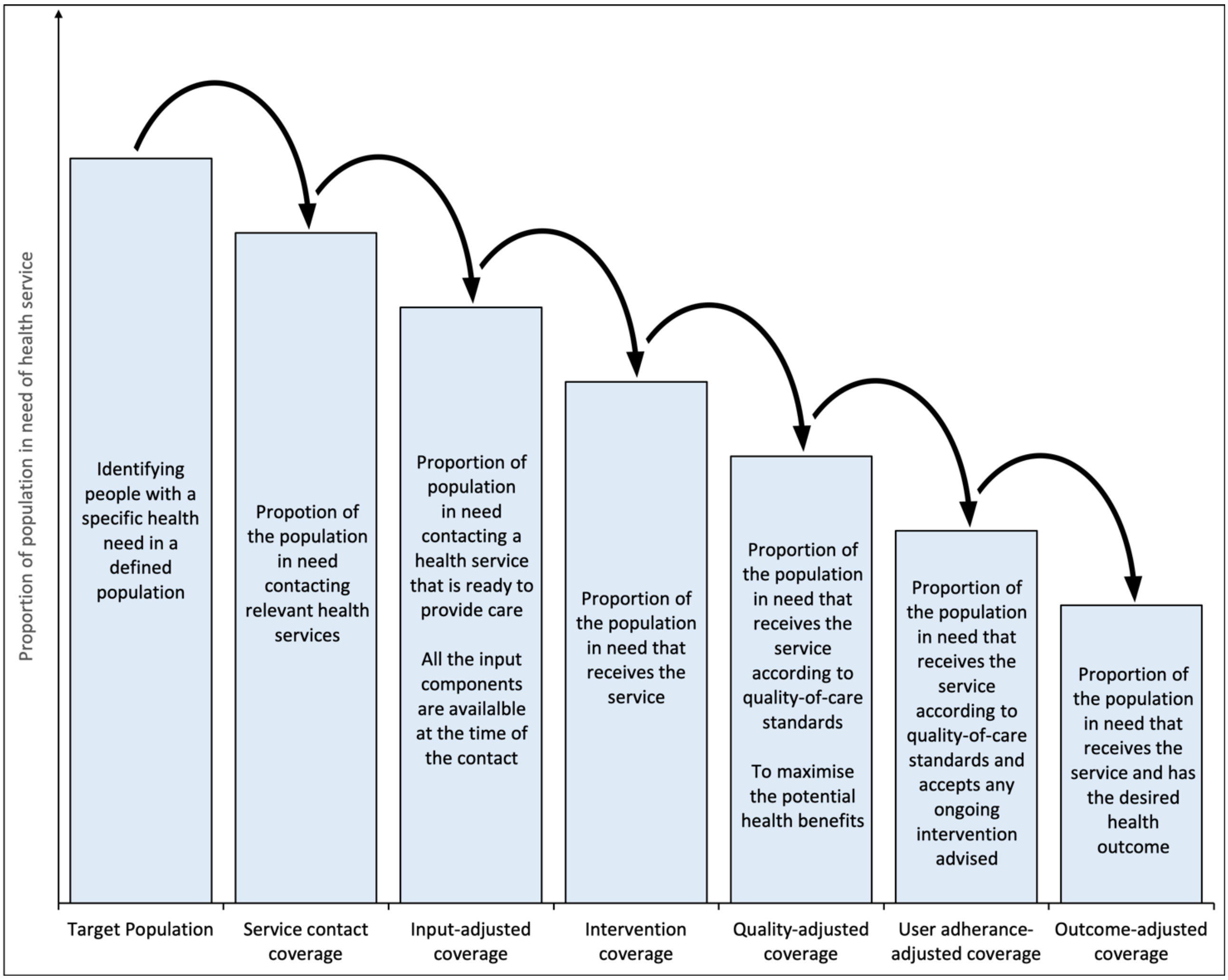

Supplementary Table 28: Definitions for cataract surgical service indicators

**Indicator**	**Definition**
Cataract Surgical Rate (CSR)	The number of cataract operations per million population per year (*Service Output*).[306,307]
Cataract Surgical Outcome (CSO)	The presenting visual acuity of the operated eye, categorised by WHO as good (6/6 – 6/18), borderline (<6/18 – 6/60) and poor (<6/60) (*Effectiveness*).[308]
Cataract Surgical Coverage (CSC)	People in a population who have received cataract surgery, as a proportion of those having operable cataract (at a defined threshold of vision impairment) plus those with operated cataract (*Intervention Coverage*).[309]
Effective cataract surgical coverage (eCSC)	People in a population with a visual acuity of 6/18 or better after cataract surgery (in one or both operated eyes), as a proportion of those having operable and operated cataract (*Outcome-adjusted coverage*).[310]

Supplementary Figure 13: Flow diagram illustrating how Effective Refractive Error Coverage (eREC) is estimated. Adapted from McCormick et al.[311] The visual acuity measures refer to the vision level in the better eye.

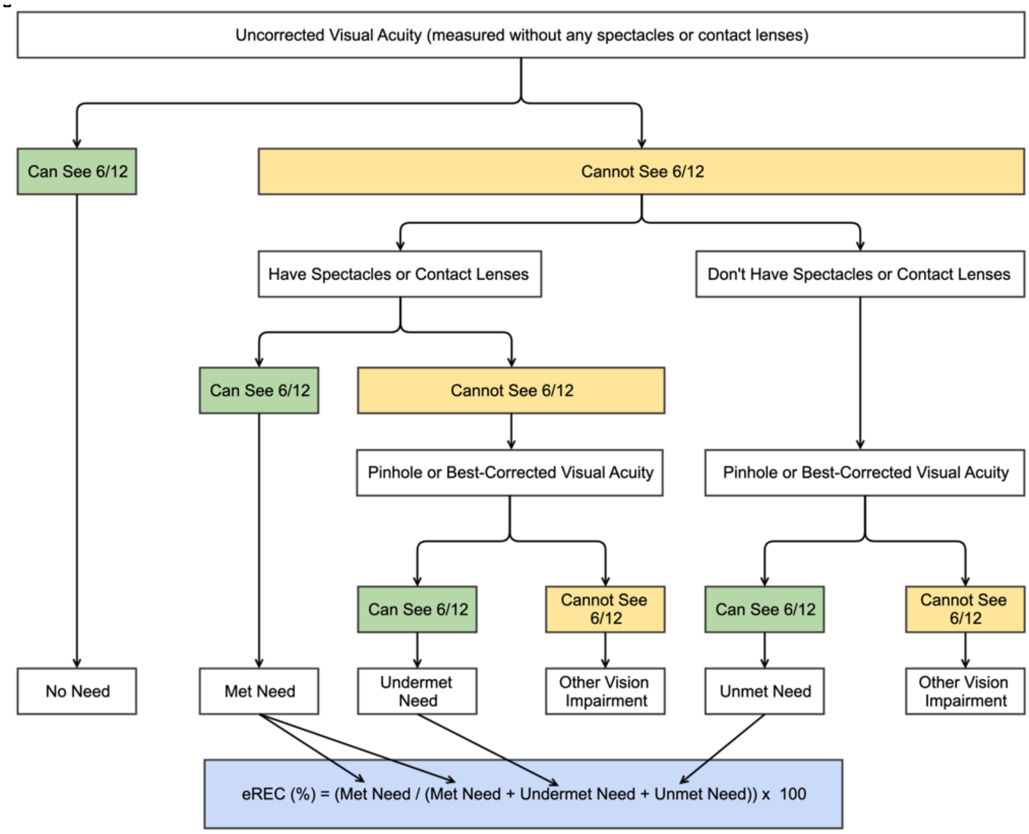

Supplementary Figure 14: Health service effective coverage cascade for refractive error services REC – refractive error coverage; eREC – effective refractive error coverage.

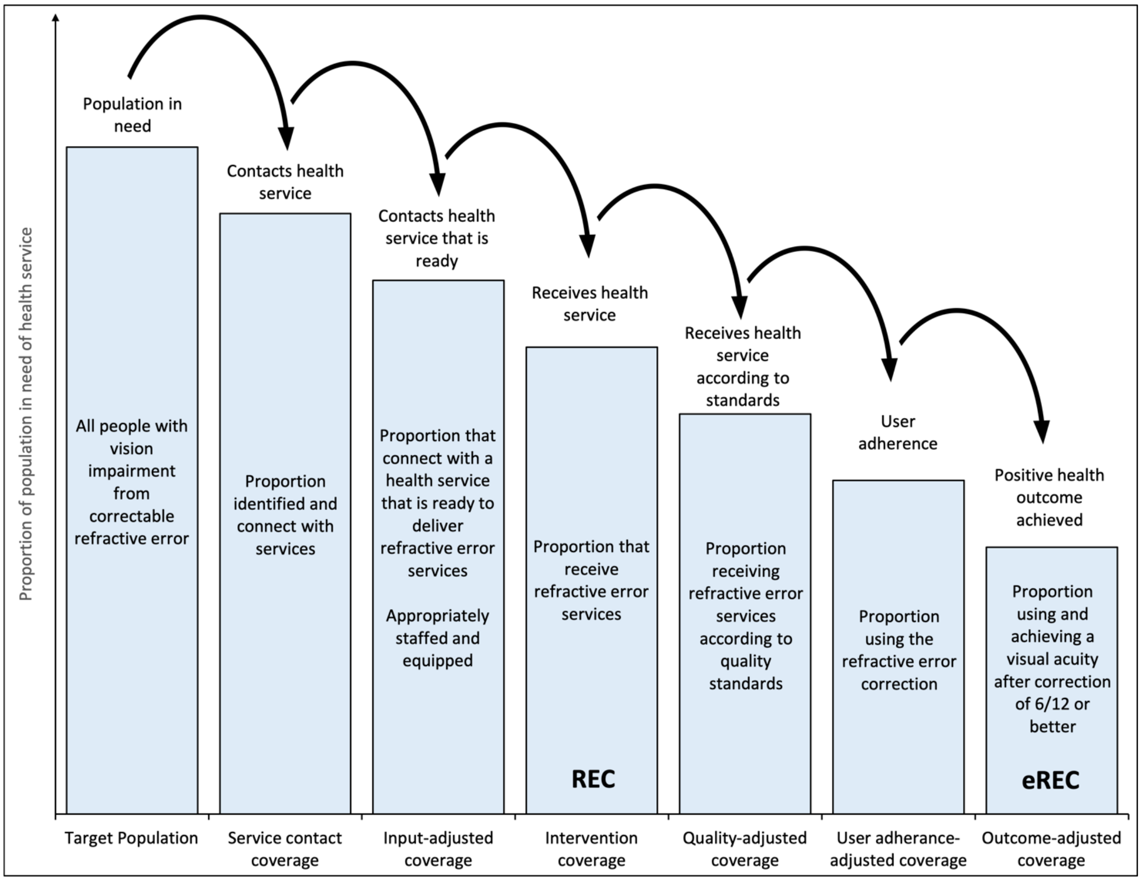

Supplementary Figure 15: Health service effective coverage cascade for glaucoma, diabetic retinopathy and AMD

(A) Effective coverage cascade for glaucoma. GSC, glaucoma service coverage; qGSC, quality-adjusted glaucoma service coverage; uGSC, user adherence-adjusted glaucoma service coverage; eGSC, effective glaucoma service coverage. (B) Effective coverage cascade for diabetic retinopathy. DRC, diabetic retinopathy coverage; qDRC, quality-adjusted diabetic retinopathy coverage; uDRC, user adherence-adjusted diabetic retinopathy coverage; eDRC, effective diabetic retinopathy coverage. (C) Effective coverage cascade for AMD. AMD-SC – AMD service coverage; qAMD-SC - quality-adjusted AMD service coverage; uAMD-SC – user adherence-adjusted AMD service coverage; eAMD-SC – effective AMD service coverage.

(A) Effective coverage cascade for glaucoma

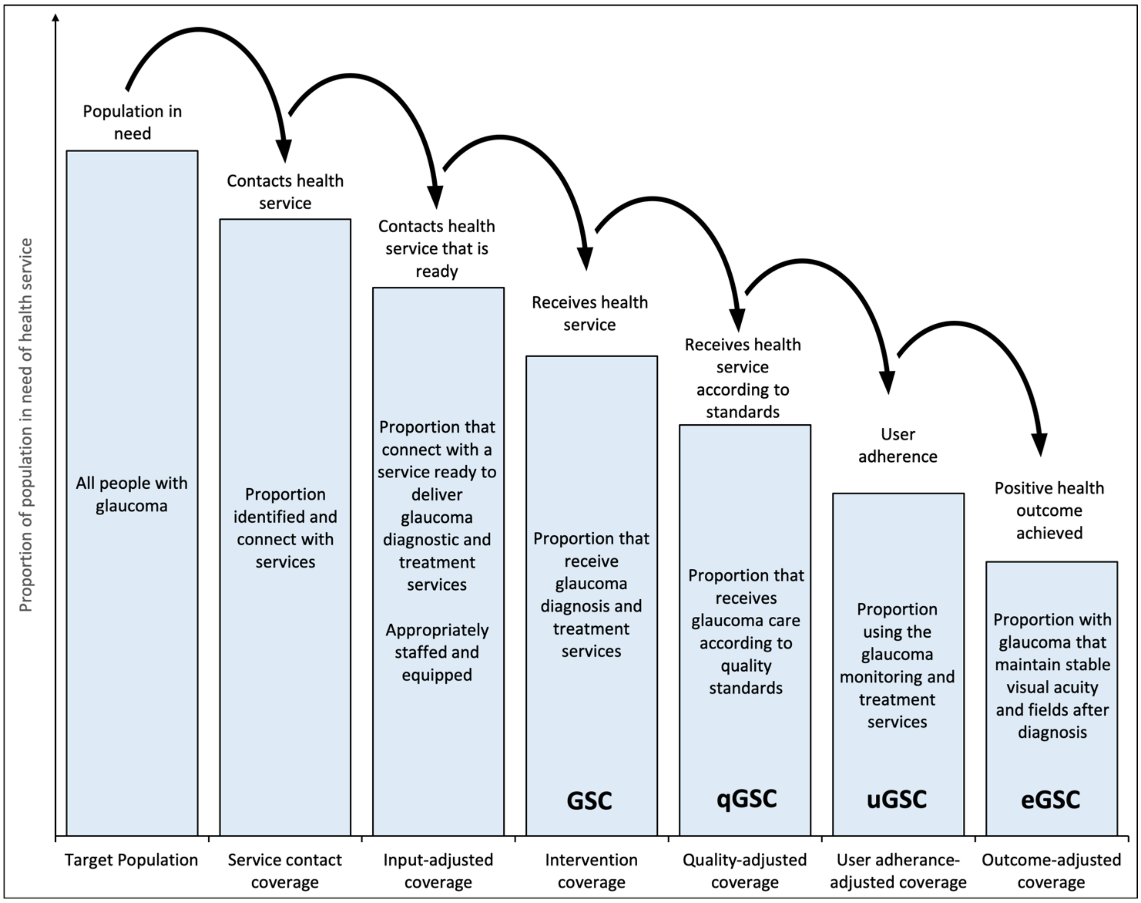

(B) Effective coverage cascade for diabetic retinopathy

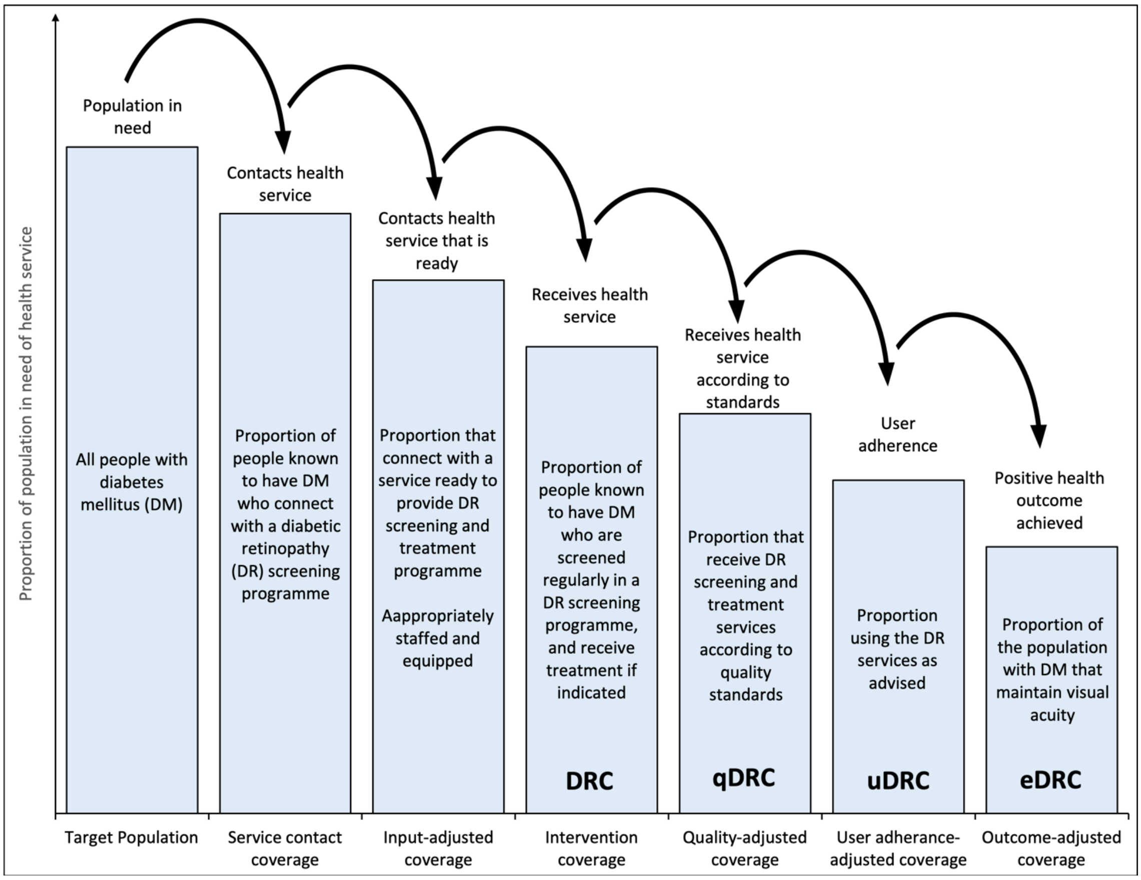

(C) Effective coverage cascade for AMD

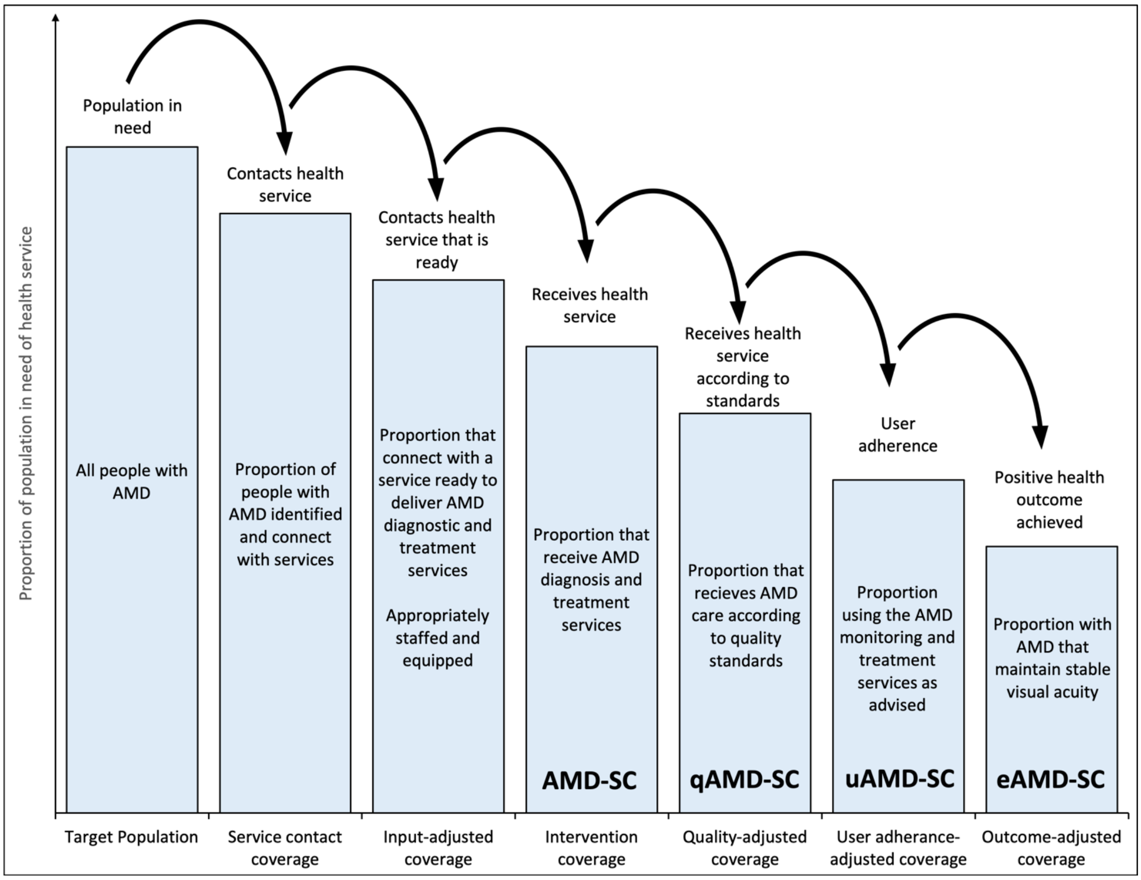

Progress towards UHC for eye health in 2020

Are we advancing Universal Health Coverage through cataract services? A scoping review

Scoping review on progress towards UHC for eye health; numbers of studies, by region, examining Access, Quality, Financial Protection and Equity for cataract surgery. The methods have been published and are summarised below.[312]

**Title:**	**Are we advancing Universal Health Coverage through cataract services? A scoping review**
Objective:	We aimed to answer two questions:<br>1. What is the nature, extent and global distribution of data on the coverage and effectiveness of cataract services?<br>2. What is the nature, extent and global distribution of data on financial protection in relation to cataract services?
Search date:	14 February 2020
Search range:	1st January 2000 – 1st February 2020
Search databases:	MEDLINE, Embase, Global Health
Key definitions: (if applicable)	*Universal Health Coverage*: people have access to needed health services of sufficient quality to be effective while also ensuring that the use of services does not expose the user to financial hardship<br>*Cataract services*: The range of activities from detection of operable cataract to the operative and post-operative care and outcome
Key inclusion/ exclusion criteria:	Included:<br>• All published primary prospective and retrospective population-level research studies and systematic reviews that report quantitative assessment of access, equity, quality or financial protection of cataract surgical services for adults at the subnational, national, regional or global level<br>• Non-published population-level reports and surveys including Rapid Assessment of Avoidable Blindness (RAAB) surveys and non-governmental organisation (NGO) reports<br>Excluded:<br>• Interventional studies<br>• Studies within clinical subpopulations (e.g. diabetic retinopathy)<br>• Studies focused on cataract services for children (aged 18 years or under)<br>• Studies published prior to 1st Jan 2000
Number of included studies:	343
Protocol registration:	Open Science Framework (https://osf.io/k3mwg/); 15th April 2020
Protocol publication:	Lee CN, Ramke J, McCormick I, Zhang JH, Aghaji A, Mwangi N, Burn H, Gordon I, Yusufu M, He M, Silva JC, Burton MJ. Are we advancing universal health coverage through cataract services? Protocol for a scoping review. BMJ Open 2020;10:e039458. http://dx.doi.org/10.1136/bmjopen-2020-039458

The summary results are provided in Supplementary Tables 29, 30 and 31; Commission Report Figure 20.

Supplementary Table 29: Reporting on progress towards UHC for eye health; numbers of studies, by region, examining Access, Quality, Financial Protection and Equity for cataract surgery

Region	Access				Quality				Financial		Equity (disaggregation of data)				Regional Total
	CSR		CSC		CSO		eCSC		Any measure		Access		Quality		
	2000s	2010s	2000s	2010s	2000s	2010s	2000s	2010s	2000s	2010s	2000s	2010s	2000s	2010s	
Andean Latin America	6	6	0	5	0	4	0	2	0	0	0	4	0	5	32
Australasia	1	7	1	2	2	1	0	1	0	0	1	3	0	1	20
Caribbean	5	22	1	4	1	3	0	1	1	0	0	2	0	2	42
Central Asia	0	2	2	3	2	1	0	0	0	0	2	3	1	0	16
Central Europe	0	22	0	1	0	0	0	0	0	0	0	1	0	0	24
Central Latin America	17	17	2	8	2	7	0	3	0	0	2	7	0	8	73
Central Sub-Saharan Africa	0	8	0	0	0	0	0	0	0	0	0	0	0	0	8
East Asia	2	9	14	11	16	15	0	0	1	0	13	10	5	2	98
Eastern Europe	0	11	0	1	0	0	0	0	0	0	0	0	0	0	12
Eastern Sub-Saharan Africa	0	26	11	10	10	7	0	5	0	0	9	8	0	6	92
High-income Asia Pacific	0	8	0	0	1	1	0	0	0	0	0	3	1	0	14
High-income North America	3	8	0	0	0	2	0	0	0	0	2	3	0	0	18
North Africa and Middle East	1	26	4	5	3	5	0	2	0	0	4	9	0	2	61
Oceania	0	9	2	1	2	1	0	0	1	0	1	1	1	0	19
South Asia	0	12	25	17	27	15	0	4	0	1	22	17	11	6	157
Southeast Asia	0	32	18	22	17	21	0	4	0	1	17	23	2	6	163
Southern Latin America	7	8	2	3	2	2	0	3	0	0	2	2	0	7	38
Southern Sub-Saharan Africa	0	9	3	1	2	1	0	0	0	0	1	2	0	0	19
Tropical Latin America	5	4	2	2	3	2	0	0	1	0	3	2	1	1	26
Western Europe	5	49	0	0	1	3	0	0	0	0	3	4	0	0	65
Western Sub-Saharan Africa	2	32	12	8	8	7	0	3	0	0	9	8	0	3	92
**Global**	**54**	**327**	**99**	**104**	**99**	**98**	**0**	**28**	**4**	**2**	**91**	**112**	**22**	**49**	1089

No. of studies
0
1
2-3
4-9
10-19
20-29
30+

Supplementary Table 30: Cataract Surgical Rate, by GBD Super Region

Super Region	Number of countries in the Super Region	Number of countries we found data for	CSR Median	IQR	Range
Sub-Saharan Africa	46	45	494	265-847	95-1993
Latin America and the Caribbean	31	30	1655	829-2352	264-9103
Southeast Asia, East Asia, and Oceania	27	25	1680	1309-2435	196-5386
North Africa and Middle East	21	19	1700	1187-2324	717-6328
South Asia	5	5	2819	1553-4364	1475-4830
Central Europe, Eastern Europe, Central Asia	29	20	5141	1209-8808	455-12560
High income countries	34	31	10136	5326-10922	1750-14188
**Total**	**193**	**175**	**1700**	**720-3906**	**95-14188**

Supplementary Table 31: Cataract surgical outcome by GBD Super Region

(A) Proportion of cataract operations leading to a presenting visual acuity of 6/18 or better.

Super Region	Number of countries in the Super Region	Number of countries we found data for	Number of estimates we found	Median % 6/18 or better	IQR	Range
Sub-Saharan Africa	46	10	13	48	38-60	29-80
Latin America and the Caribbean	31	13	16	61	55-66	42-81
Southeast Asia, East Asia, and Oceania	27	9	31	59	52-66	28-76
North Africa and Middle East	21	5	5	60	38-60	35-62
South Asia	5	5	14	67	60-72	43-81
Central Europe, Eastern Europe, Central Asia	29	1	1	62	.	.
High income countries	34	2	2	76	.	70-82
**Total**	**193**	**45**	**82**	**60**	**50-68**	**28-82**

(B) Proportion of cataract operations leading to a presenting visual acuity of worse than 6/60.

Super Region	Number of countries in the Super Region	Number of countries we found data for	Number of estimates we found	Median % worse than 6/60	IQR	Range
Sub-Saharan Africa	46	11	15	30	21-38	9-51
Latin America and the Caribbean	31	13	16	20	19-22	8-36
Southeast Asia, East Asia, and Oceania	27	9	31	16	12-23	3-30
North Africa and Middle East	21	5	5	19	18-30	17-38
South Asia	5	5	15	14	12-15	8-25
Central Europe, Eastern Europe, Central Asia	29	1	1	23	.	.
High income countries	34	3	3	9	.	3-15
**Total**	**193**	**47**	**86**	**18**	**13-25**	**3-51**

Supplementary Figure 16: Cataract surgical outcome by GBD Super Region

(A) Proportion of cataract operations leading to a presenting visual acuity of 6/18 or better. (B) Proportion of cataract operations leading to a presenting visual acuity of worse than 6/60. Box and whisker plots: midlines are the median values, the boxes represent the interquartile range, the whiskers the upper and lower adjacent values. Outliers are plotted as individual dots. Data are provided in Supplementary Table 31.

(A) Proportion achieving 6/12 or better

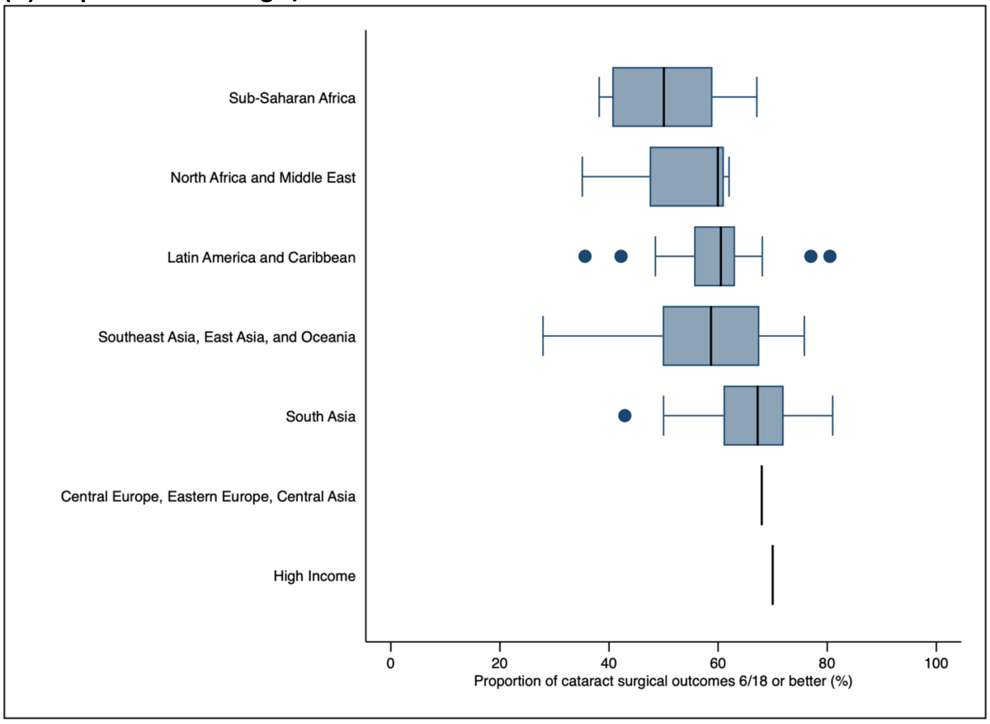

(B) Proportion worse than 6/60

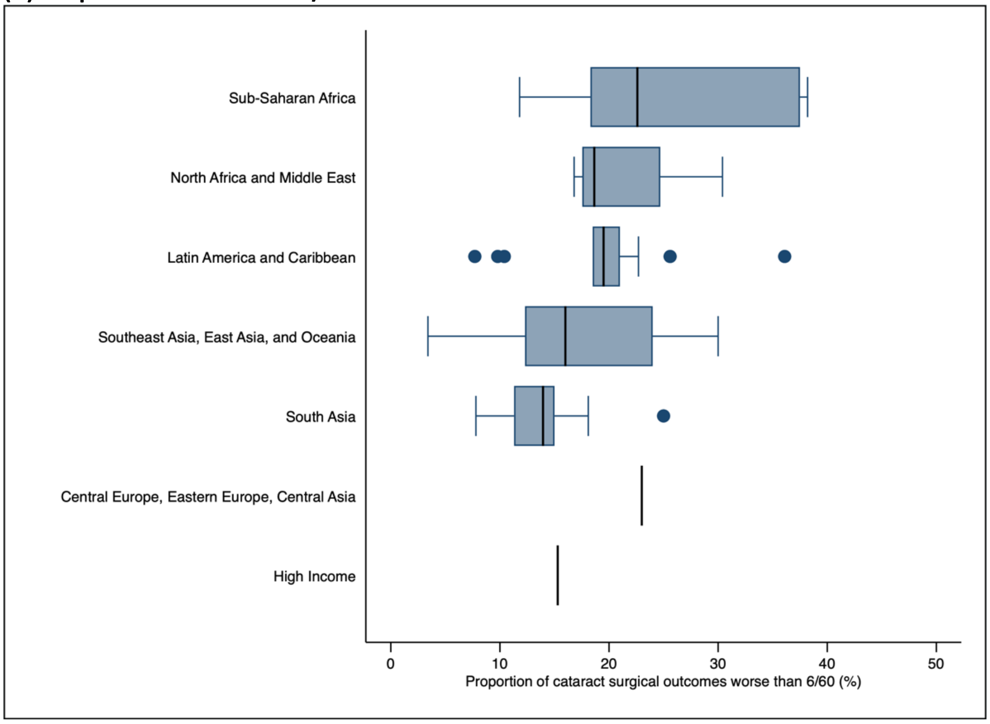

Enhancing cataract services by better integration with refractive error services

Forthcoming comment: **We can improve cataract surgery outcomes by strengthening integration with refractive error services**

We identified all studies from the scoping review outlined on pp 80-83 that reported cataract surgical outcome (CSO) using presenting visual acuity as well as pinhole visual acuity (a proxy for best-corrected visual acuity with spectacles or contact-lenses). Across 112 studies, the median 'good CSO' of 6/18 or better with presenting visual acuity was 58% and with pinhole visual acuity was 70%, a 21% relative improvement. Across 110 studies, the median 'poor CSO' of worse than 6/60 was 21% with presenting visual acuity and 17% with pinhole visual acuity, a 19% relative improvement. Thus, if refractive error services were better integrated with cataract services, poor outcomes could be reduced by 20% and good outcomes could be increased by 20%.

Summary of methods for the calculation of eCSC from RAAB data

Supporting information for:

Commission Report Figure 21: Effective Cataract Surgical Coverage, by GBD Super Region
Commission Report Figure 22: CSC and eCSC in Vietnam.

Publication pending.

The RAAB Repository (www.raabdata.info) curates information relating to surveys conducted from 1995 up to 2019. Where Principal Investigators have given permission for their anonymous and unlinked raw survey data to be made open access and hosted publicly, the Repository provides a link to download that data.

We downloaded each available data set and its associated population age-sex structure information. We calculated adjusted and gender-disaggregated CSC and eCSC estimates per survey using the equations in the box below.

Where a country had only one survey, we included the estimate from that survey in the summary. Where two or more surveys had been conducted in a country, the most recent estimate was included in the summary unless a nationally representative survey had been conducted less than 5 years previous to a sub-national survey, in which case the national estimate was used. Where multiple sub-national surveys had been conducted within a two-year period, we used the median estimate from the series. eCSC estimates used for each country were taken from national or subnational sampling areas depending on availability.

Based on this approach, we included 93 eCSC estimates from 48 countries across all Global Burden of Disease (GBD) Super Regions. Count of individual RAAB survey estimates contributing to each Global Burden of Disease Super Region summary.

**GBD Super Region**	**Contributing estimates (n)**
Sub-Saharan Africa	11
South East Asia, East Asia and Oceania	49
Latin America and Caribbean	13
North Africa and Middle East	7
South Asia	7
Central Europe, Eastern Europe, Central Asia	3
High Income	3
**Total**	**93**

Cataract surgical coverage **(CSC)** is defined as the number of people in a defined population with operated cataract as a proportion of those having operable plus operated cataract (i.e. pinhole visual acuity worse than 6/60).

$$CSC_{persons} = (x + y) / (x + y + z) * 100\ (\%)$$

where
x = individuals with unilateral pseudo/aphakia (i.e. operated cataract) and operable cataract in the other eye;
y = individuals with bilateral pseudo/aphakia, regardless of visual acuity;
z = individuals with bilateral operable cataract.

Effective cataract surgical coverage **(eCSC)** measures the number of people in a defined population with operated cataract and a good outcome (i.e. presenting vision 6/18 or better) as a proportion of those having operable plus operated cataract. As for CSC, eCSC is calculated using the cut-off for operable cataract of worse than 6/60 pinhole visual acuity.

$$eCSC_{persons} = (a + b) / (x + y + z) * 100\ (\%)$$

where
a = individuals with unilateral pseudo/aphakia achieving presenting visual acuity of 6/18 or better in the operated eye and operable cataract in the other eye;
b = individuals with bilateral pseudo/aphakia achieving presenting visual acuity of 6/18 or better in at least one eye;
x, *y* and *z* as above for CSC.

To further summarise this information, we calculated gender-disaggregated median and interquartile range (IQR) eCSC estimates for each Global Burden of Disease super region and presented them as box and whisker plots (Commission Report Figure 21). To investigate temporal trends in CSC and eCSC we compared the median values from a three-survey series conducted at distinct points in time in Vietnam (Figure 22a in main text). Using the same Vietnam data, we demonstrated the effect of changing the visual acuity thresholds for 'operable cataract' and a 'good' outcome on the coverage level achieved (Figure 22b in main text).

Supplementary Figure 17: Disaggregated prevalence of effective cataract surgical coverage (eCSC) in the Nigeria National Survey (2005-2007) and Sri Lanka National Survey (2012-2014).

Data from population-based surveys of people ≥40 years, disaggregated by gender, urban or rural location and marital status. (A) and (B) disaggregated eCSC values (95% CI) for different population sub-groups. The eCSC target of 60% reflects 80% coverage with 80% good outcomes. There were no urban not-married men with cataract blindness in Sri Lanka. Adapted from Ramke et al.[313]

(A) Nigeria eCSC **(B) Sri Lanka eCSC**

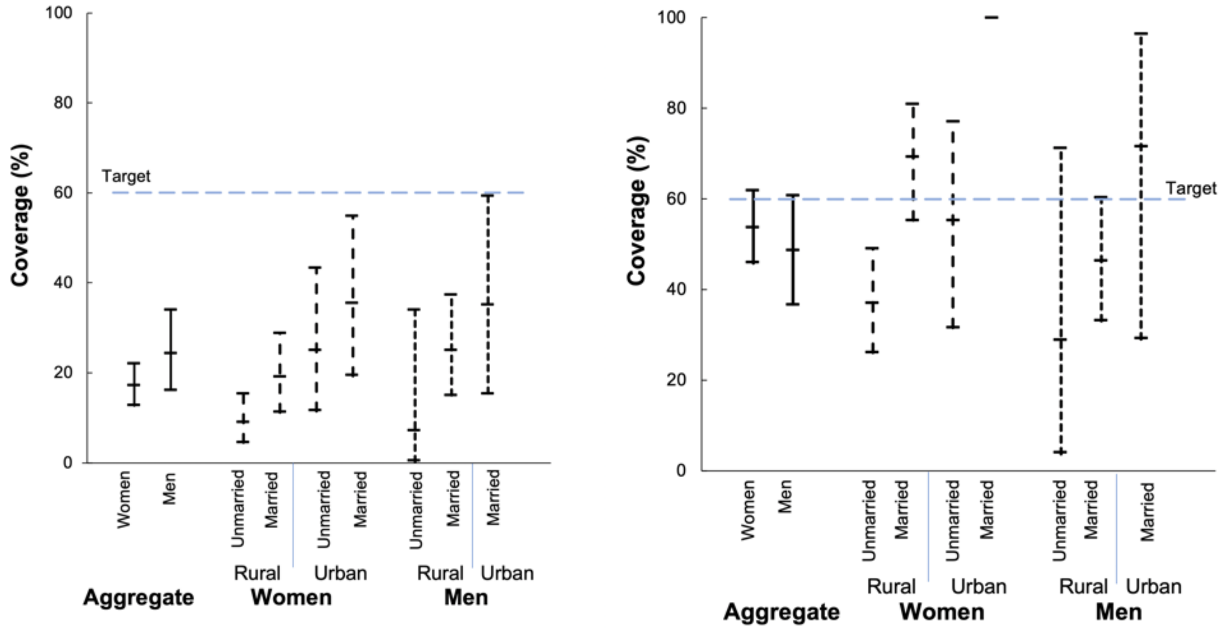

Supplementary Figure 18: Disaggregated prevalence of cataract blindness in the Nigeria and Sri Lanka National Eye Health Surveys.
Data from the Nigeria National Survey (2005-2007) and Sri Lanka National Survey (2012-2014): population-based surveys of people ≥40 years, disaggregated by gender, urban or rural location and marital status. (A) and (B) prevalence of cataract blindness for different population sub-groups. The figures (%) on top of each bar are the proportion of the total population cataract blindness found in that sub-group. **No urban not-married men with cataract blindness in Sri Lanka. Adapted from Ramke et al.[313]

(A) Nigeria cataract blindness prevalence

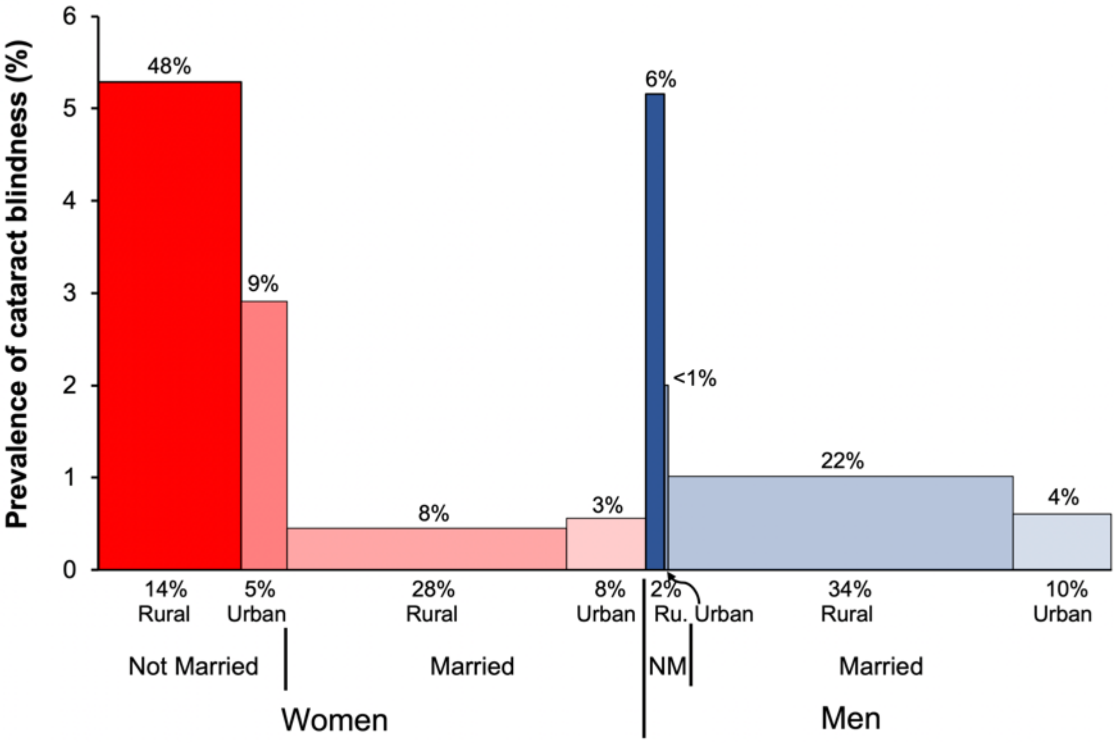

(B) Sri Lanka cataract blindness prevalence

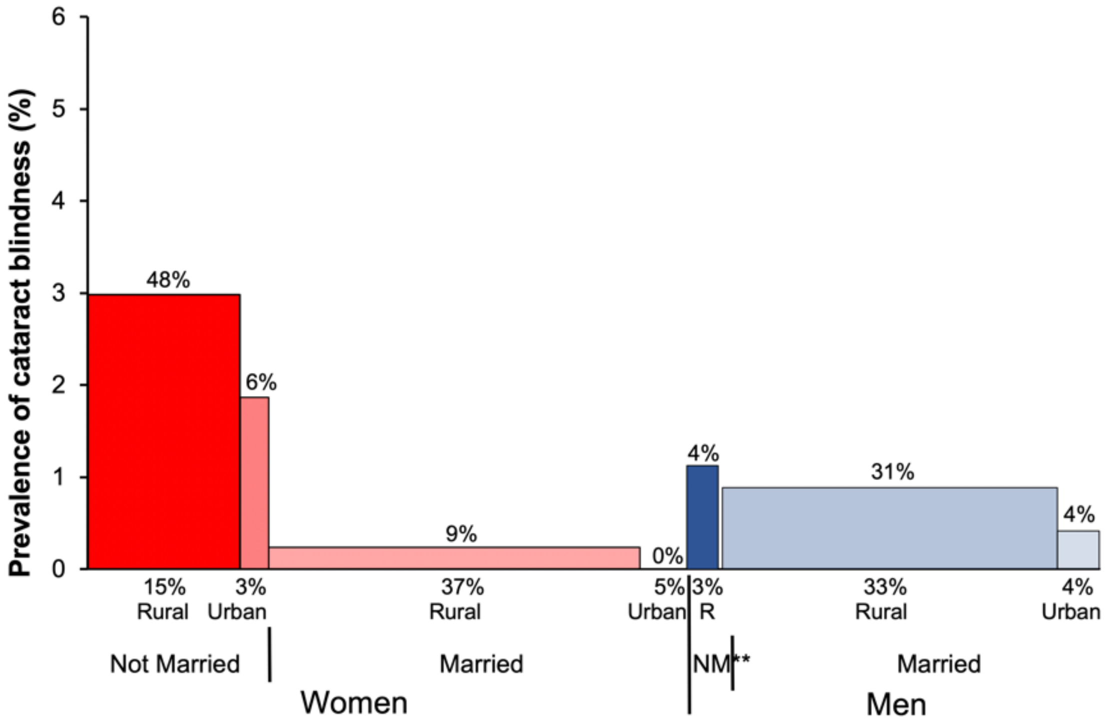

Building the quality of eye care

Summary of methods

**Title:**	**Interventions to improve quality of cataract services: a global scoping review**
Objective:	We aimed to answer the following three questions:<br>1. What interventions to improve quality of cataract services have been described in the published literature?<br>2. Which element(s) of quality did the interventions address?<br>3. Where was the evidence generated (high- vs middle- vs low-income settings, geographic region)?
Search date:	17 November 2019
Search databases:	MEDLINE, Embase and Global Health
Key definitions:	*Cataract services*: the range of activities on the pathway from detecting people with operable cataract, to these people undergoing surgery and receiving post-operative care.<br>*Quality*: WHO's seven elements of quality—effectiveness, efficiency, equity, integration, people-centredness, safety and timeliness—to which we added planetary health.
Key inclusion/ exclusion criteria:	Included:<br>• primary research studies of any design from any country that reported a quality-relevant outcome for primary age-related cataract following an intervention related to quality of cataract services. Systematic reviews were also included if meta-analysis was conducted for a quality-relevant outcome.<br>• studies where an intervention was compared against an alternative (e.g. intervention vs. no intervention / current practice vs. new intervention / before vs. after implementation).<br>Excluded:<br>• studies that assessed specific surgical techniques (e.g. phacoemulsification vs. manual small incision surgery, site of anaesthesia) and/or specific products and medications used during and around the time of surgery<br>• studies focussed exclusively on cataract services for children (aged under 18 years)<br>• studies published prior to 1990
Number of included studies:	143
Protocol registration:	Open Science Framework (https://osf.io/8gktz); 11 December 2019
Protocol publication:	Yoshizaki M, Ramke J, Furtado J, Gichuhi S, Burn H, Gordon I, Aghaji A, Marques AP, Dean W, Congdon N, Buchan J, Burton MJ. Interventions to improve quality of cataract surgery: protocol for a scoping review. BMJ Open 2020 0(8):e036413 http://dx.doi.org/10.1136/bmjopen-2019-036413

Supplementary Table 32: The extent of evidence for interventions addressing each quality element of cataract services, by GBD super-region

	Southeast Asia, East Asia, Oceania	South Asia	Central Europe, Eastern Europe, Central Asia	North Africa Middle East	Sub-Saharan Africa	Latin America Caribbean	High Income	International Studies	Meta- analysis	Total
**Efficiency**	3	1	2	2	0	4	39	1	6	58
**People-centredness**	5	3	1	4	0	0	27	0	0	40
**Effectiveness**	0	0	0	1	2	0	11	0	1	15
**Safety**	3	1	0	1	0	1	6	0	0	12
**Equity**	3	3	0	1	2	0	2	0	0	11
**Integration**	0	1	0	0	0	0	3	0	0	4
**Timeliness**	0	0	0	0	0	0	3	0	0	3
**Planetary Health**	0	0	0	0	0	0	0	0	0	0
**All Studies**	14	9	3	9	4	5	91	1	7	143

No. of studies
0
1
2-3
4-9
10-19
20-29
30+

Supplementary Table 33: Studies reporting interventions to improve quality of cataract surgery (excluding surgical procedure)

Intervention	Summary of findings reported by authors
**Efficiency**	
Day vs. inpatient surgery	• Day surgery reduced cost (*efficiency*) while maintaining postoperative VA (*effectiveness*)
Immediate sequential vs. delayed bilateral cataract surgery	• Immediate sequential reduced provider and patient cost (*efficiency*) and achieved quicker and sustained improvement in QoL and visual function (*people-centredness*)<br>• Immediate sequential did not compromise *effectiveness* or *safety*—similar postoperative VA and complication rates compared to DSBCS
Changes to service delivery model	• Single-function cataract treatment centre reduced cost per patient (*efficiency*) and improved outcomes (*effectiveness*)<br>• Standardised cataract surgery contract that embedded quality measures increased the volume of surgery and proportion of day-surgeries (*efficiency*)
Selective pre-operative medical evaluation	• Conducting preoperative medical evaluation only for those with high risk of adverse medical events reduced cost and time for provider, reduced cancellation rates (*efficiency*) and reduced the number of visits required (*people-centredness*) while maintaining *safety* (adverse medical events) and postoperative VA (*effectiveness*)
Changes to reimbursement	• In Thailand, introduction of a centrally reimbursed fee schedule policy increased the volume of cataract surgery and the cataract surgical rate.<br>• Regulated competition introduced in Netherlands increased the volume and safety of cataract surgery
Postoperative review at 2 weeks	• Omitting Day 1 postoperative review improved *efficiency*, and did not compromise *effectiveness* or *safety*
Change management programmes	• Change management (e.g. plan-do-study-act) involving a multidisciplinary team improved *efficiency*, reduced costs and complications (*safety*).
Task shifting	• Nurse-led sedation improved access to cataract surgery (*efficiency*) without compromising *safety*
Capacity building	• Non-governmental or high-performing hospitals acting as mentor to underperforming hospitals improved in capacity in underperforming hospitals (*efficiency*).
**People-centredness**	
Pre-operative education	• Using multimedia presentations (e.g. video, computer-based) or visual aids (e.g. 3D model of eye) improved patients' knowledge about cataract surgery compared to verbal information only; also reduced time needed for informed consent (*efficiency*) and reduced anxiety among patients (*people-centredness*).
Pain / anxiety management	• Music before and during the operation reduced the pain experience and increased satisfaction but showed mixed results for anxiety level.<br>• Patients who received pre-operative psychological care were more cooperative and satisfied with surgery and required less IV sedation.
Omitting the traditional eye patch	• Omitting the traditional eye patch (no patch, transparent shield only or therapeutic bandage contact lens) provided instant vision for operated eye (*people-centredness*), without compromising *safety* (no difference in incidence of intraocular pressure and flare, corneal condition).
Continuing nursing care	• Continuing nursing care at home for a year following discharge of cataract surgery effectively addressed patients' other physical health needs (e.g. diabetes, blood pressure) as well as achieved better visual acuity (*effectiveness*) in China.
**Effectiveness**	
Second eye surgery	• Second-eye surgery improved clinical vision (e.g. VA, contrast sensitivity), functional vision (e.g. reading speed, facial recognition) and quality of life (*people-centredness*) compared to surgery for one eye alone in patients with bilateral cataract.
Biometry	• Using biometry to inform intraocular lens (IOL) power, rather than using the standard IOL for all patients, improved refractive outcome of cataract patients (*effectiveness*) in LMIC setting.
Monitoring surgical outcomes	• Monitoring of surgical outcomes to each surgeon improved visual outcomes (*effectiveness*) and reduced complication rates (*safety*) in LMIC setting.
Risk stratification and matching of surgeon	• Risk stratification based on potential intraoperative complications risk and matching of surgeon based on their experience improved overall visual outcomes (*effectiveness*) and reduced complication rates (*safety*), hence providing safer system for surgeon training.
Surgeon advice on future need of spectacle use	• Surgeon advice on future need of spectacle use at the time of surgery improved best corrected visual acuity (*effectiveness*), patient satisfaction and vision related activity limitation (*people-centredness*).

Eye health and planetary health

Forthcoming publication:

J Buchan, C Thiel, A Steyn, J Somner, R Venkatesh, M Burton, J Ramke. Addressing the environmental sustainability of eye health delivery: a scoping review.

Summary of methods

**Title:**	**Addressing the environmental sustainability of eye health delivery: a scoping review**
Objective:	We aimed to answer two questions:<br>1. What is the nature and extent of the literature describing the environmental costs of delivering eye care services, and the interventions to diminish those environmental costs?<br>2. What breadth of engagement is there with this issue in terms of sources of evidence? What environmental sustainability themes are not being reported upon?
Search date:	12 March 2020
Search databases:	MEDLINE, Embase and Global Health
Key inclusion/ exclusion criteria:	Included:<br>• Observational studies of the environmental impact of eye health care provision (quantified as carbon dioxide equivalent or other measure of greenhouse gas (GHG) emission, air or water toxins or other pollutants)<br>• Interventional studies investigating strategies to diminish the environmental impact of eye health care provision<br>• Systematic literature reviews or modelling exercises that explore either negative environmental impact of eye health care or interventions to mitigate environmental impact of health care which report eye care specifics<br>Excluded:<br>• Studies of waste (eg wasting water in surgical scrub or wasting drugs) which do not directly or indirectly evaluate the environmental impact of the waste<br>• Studies that increase productivity for the same resource utilisation but do not quantify the environmental costs per unit activity<br>• Editorials or other articles which do not report any primary data
Number of included studies:	8
Protocol registration:	Open Science Framework (https://osf.io/cn5wp/)

Summary of findings

It is now accepted that climate change is happening and that it represents the greatest long-term threat to global population health in the 21st Century.[314] Human activity, primarily mediated through greenhouse gas (GHG) emissions from the world's larger economies, is the major driver.[315] The impact is most acute in LMICs. Global healthcare is estimated to contribute approximately 5% of all GHG emissions.[316,317] Eye care is a high-volume service in many countries, with large number of consultations and procedures annually. For example, in the UK ophthalmology is now the highest volume speciality, accounting for 8.14% of hospital outpatient visits nationally in 2018/19.[318] Therefore, eye care is already a significant contributor to health care emissions. With an ageing global population increasingly requiring eye care interventions, it is essential we promote sustainable practice and deconstruct unsustainable systems now.

To date, the environmental impact of eye care services has received little attention; the climate emergency places responsibility on all sectors to carefully consider their individual contributions to addressing it. To examine the extent and nature of the potential environmental impact of eye health services, a scoping literature review was undertaken as part of the Commission. A summary is provided in the supplementary annex and the full results are forthcoming in a separate publication. There is a profound paucity of evidence; only eight reports met the inclusion criteria. All were published after 2009, showing this to be a new field of study. Most reported carbon dioxide equivalent (CO_2e) as an outcome measure, permitting limited comparisons between studies, although methodologies were diverse. Calculation of CO_2e for the separate components of a service, such as buildings, travel and procurement, were considered impracticable, however using publicly available standard carbon cost frameworks, carbon footprints can be calculated in a standardised way.[319]

A detailed carbon footprint of phacoemulsification cataract surgery has been estimated for individual centres in the UK and India.[320,321] For the same procedure, considering only equivalent components, the CO_2e were 132.9kg CO_2e in the UK and 5.9kg CO_2e in India. The reasons why a single phacoemulsification cataract operation in the UK produces twenty times more CO_2e than the same procedure, delivered safely and to a very high standard of clinical effectiveness in India, need teasing out.[322,323] Excluding transport, the major component comes from procurement of medical supplies (Figure). Even at current activity levels, if Indian cataract services adopted UK cataract surgical practices, this would generate an additional 1.16 megatonnes of CO2e annually, requiring 30 million new tree seedlings growing for 10 years to sequester this amount of carbon.

It is clear from just these two studies that there can be large variations in the GHG emissions associated with the same surgical intervention. To move this field forward, tools are being developed to routinely measure environmental costs

associated with cataract surgery as a mark of quality, alongside the other measures of high-quality services. Convenient life cycle assessment tools have been developed to facilitate evaluation of the carbon footprint of cataract surgery, available as the Eyefficiency App for use on smartphones.[324]

The initial focus on cataract in the literature is understandable, however, this now needs to be broadened to examine many other aspects of practice. The frequent pattern in HICs of using disposable instruments and single-use eye drops in clinical settings add substantially to the carbon footprint.[325,326] This is an approach that is beginning to be questioned on environmental grounds.[320] The main barrier to HIC services adopting more sustainable practices are the regulatory constraints that enforce reduction of perceived clinical risks to zero, but at a financial and environmental price.[325] New ways of ensuring individual patient safety will need to emerge that also protect the environment, which is key to the health of all people. Every aspect of practice can be examined and opportunities to reduce consumption identified. There is an opportunity for HICs to learn much from highly efficient units in LMICs. This process would also benefit from close collaboration between service providers and industry. Changing practice, and the policy environment that regulates this, will need evidence.

Ultimately, both HICs and LMICs will need to function within the boundaries imposed by finite planetary resources. Healthcare providers, in close partnership with pharmaceutical and manufacturing industries, need to migrate to more sustainable delivery. Therefore, as we seek to develop services to deliver eye health within UHC, to cover more conditions and reach more people, expansion must be at least matched by contraction of the environmental resource utilisation per case and a marked increase in efficiency.

Carbon footprint of cataract surgery in the United Kingdom and India
(A) Comparison of the sources of greenhouse gas emissions for phacoemulsification cataract surgery in the UK and India. The total CO2e per case operated was 132.9kg and 5.9kg for the UK and India, respectively. Adapted from: Thiel et al.[320] Refuse generated by (B) one phacoemulsification operation in the UK, and (C) 32 phacoemulsification operations in India.

(A) Emission sources

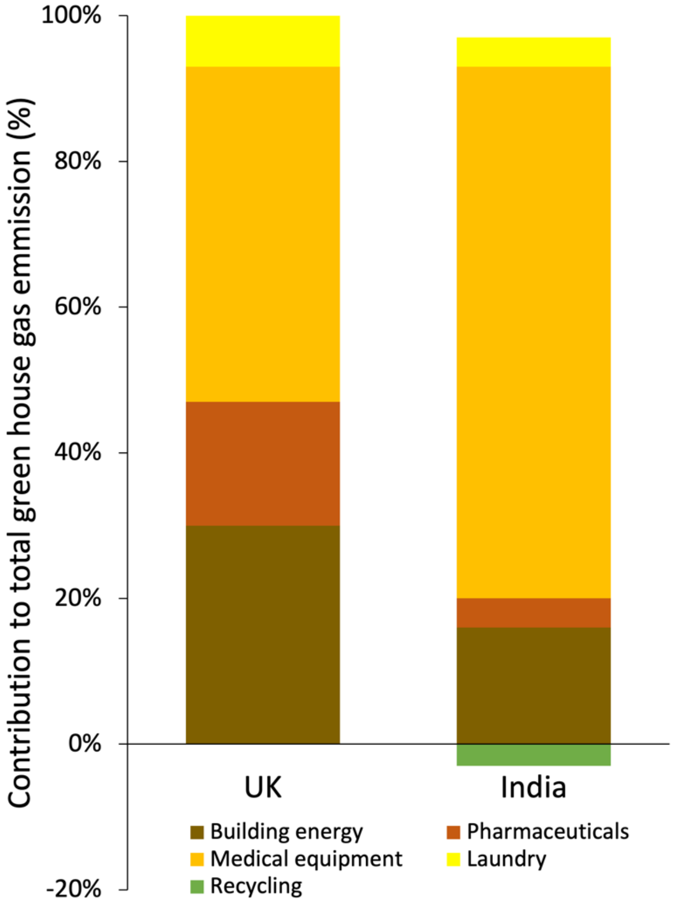

(B) United Kingdom – one cataract operation

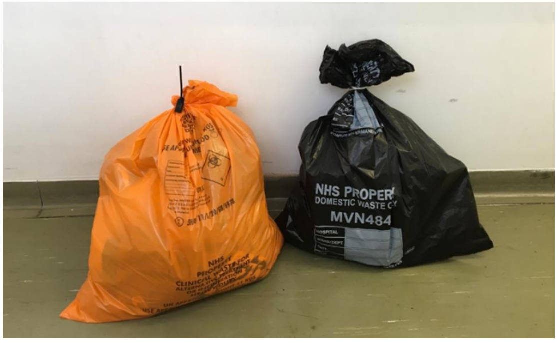

(A) India – 32 cataract operations

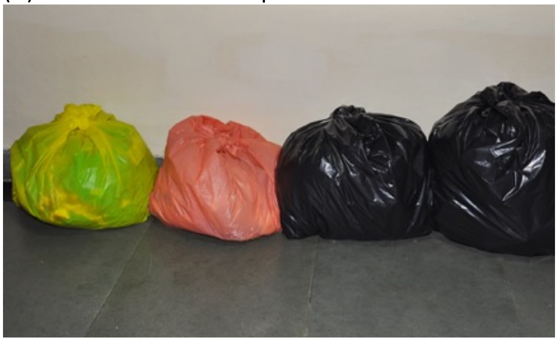

Increasing access and equity in eye care

Supporting information for Commission Figure 23: Strategies to improve access to eye care for Indigenous and other non-dominant ethnic groups. This figure draws on the results of two separate scoping reviews. The protocols are published, the publications are forthcoming, and a methods summary is provided below.

Models to improve access to eye care for Indigenous people in high-income countries: a scoping review
Burn H, Hamm L, Black J, Burnett A, Harwood M, Burton MJ, Evans JR, Ramke J.
BMJ Global Health, 2021, *In press*

Title:	**Eye care delivery models to improve access to eye care for Indigenous people in high-income countries: a scoping review**
Objective:	We aimed to answer three questions:<br>1. What were the number and characteristics of published papers describing service delivery models to improve access to eye care for Indigenous peoples in high-income countries?<br>2. What methods and interventions are used by these service delivery models to improve access to eye care for Indigenous peoples in high-income countries?<br>3. What are the current gaps in the literature and what lessons can be learnt regarding models that have been successful in improving access?
Search date:	25 January 2019 and updated 2 July 2020
Search databases:	MEDLINE, Embase and Global Health
Key definitions:	*Eye care service delivery model:* any organised programme designed to provide or improve eye care services, ranging from non-specialised primary healthcare to tertiary ophthalmic care.<br>*Access:* definition guided by the conceptual framework of patient centred healthcare access by Levesque et al, which emphasises the importance of both the supply and demand sides of healthcare access.[327]<br>*Indigenous peoples:* defined according to the United Nations Permanent Forum on Indigenous Issues using eight criteria.
Key inclusion/ exclusion criteria:	Included:<br>• Primary research studies of any design from high-income countries describing any service delivery model to improve access to eye care, where the target population of the service delivery model was Indigenous peoples.<br>• There was no time limit on publication dates and no language limitations.
Number of included studies:	67
Protocol publication:	Burn H, Black J, Harwood M*, et al.* Eye care delivery models to improve access to eye care for Indigenous people in high-income countries: protocol for a scoping review *BMJ Open* 2019;**9:**e029214.<br>http://doi: 10.1136/bmjopen-2019-029214

Interventions to promote access to eye care for non-Indigenous, non-dominant ethnic groups in high-income countries: a scoping review

Title:	**Interventions to promote access to eye care for non-Indigenous, non-dominant ethnic groups in high-income countries: a scoping review**
Objective:	We aimed to answer three questions:<br>1. What is the extent of the published literature on interventions to promote access to eye care for non-Indigenous, non-dominant ethnic groups living in high-income countries?<br>2. What can we learn from reported effectiveness of interventions?<br>3. What can we learn from authors' reflections on the potential to improve on the interventions?
Search date:	28 July 2019 and updated 26 August 2020
Search databases:	MEDLINE, Embase and Global Health
Key definitions:	*Interventions to promote access to eye care:* any organised programme designed to provide or improve eye care services, which had been executed<br>*Access:* definition guided by the conceptual framework of patient centred healthcare access by Levesque et al, which emphasises the importance of both the supply and demand sides of healthcare access.[327]<br>*Non-Indigenous, non-dominant ethnic groups*: Ethnic identities which are disempowering in their immediate context ('ethnic minority' often used in health research). This could include refugees and recent immigrants as well as those who have lived in the country of residence for many generations. Since we have chosen to address Indigenous populations in a separate review, our definition here is limited to people who are not Indigenous to the country in which the study is based.
Key inclusion/ exclusion criteria:	Included:<br>• Primary research studies of any design from high-income countries describing an intervention to improve access to eye care, where more than 50% of the target population was non-indigenous, non-dominant people.<br>• There was no time limit on publication dates and no language limitations.
Number of included studies:	71 (across 82 reports)
Protocol publication:	Hamm L, Black J, Burn H, Gray C, Harwood M, Peiris-John R, Gordon I, Burton MJ, Evans JR, Ramke J. Interventions to promote access to eye care for, non-Indigenous, non-dominant ethnic groups in high-income countries: a scoping review protocol. *BMJ Open* 2020;10:e033775.<br>http://dx.doi.org/10.1136/bmjopen-2019-033775

Gender and eye health: overview of systematic reviews

Forthcoming publication:
Gender and eye health: an overview of systematic reviews.
Jacqueline Ramke, Nyawira Mwangi, Solange Salomão, Lizette Mowatt, Joanna Black, Anthea Burnett, Fatima Kyari, Sumrana Yasmin, Burton MJ, Jennifer Evans.

Summary of methods

**Title:**	**Gender and eye health: an overview of systematic reviews**
Objective / Review question:	What is the nature and extent of the evidence in published systematic reviews on:<br>1. gender inequality in eye health (e.g. from prevalence surveys); and<br>2. interventions to reduce gender inequality.
Search date:	24 July 2019; updated 24 September 2020
Search databases:	MEDLINE, Embase and Global Health
Definitions:	*Systematic review*: a scientific investigation that focuses on a specific question and uses explicit, pre-specified scientific methods to identify, select, assess, and summarize similar but separate studies.
Key inclusion/ exclusion criteria:	Included:<br>• published systematic reviews that report any of the following outcomes:<br>○ prevalence of eye problems in women and men (or female/male children) separately (e.g. prevalence of visual impairment, barriers to uptake of services); or<br>○ an estimate of the relative prevalence of eye problems in women and men (e.g. prevalence ratio or odds ratio of likelihood of blindness in women compared to men); or<br>○ the uptake of eye health interventions in women and men separately (e.g. uptake of cataract surgery, spectacle compliance).<br>• Any population group; no time limit and no language limits.
Number of included studies:	58 (in 79 reports)
Protocol registration:	Jacqueline Ramke, Nyawira Mwangi, Solange Salomão, Lizette Mowatt, Joanna Black, Anthea Burnett, Fatima Kyari, Sumrana Yasmin, Jennifer Evans. Gender and eye health: protocol for an overview of systematic reviews. PROSPERO 2019 CRD42019139017; 24 July 2019<br>https://www.crd.york.ac.uk/prospero/display_record.php?ID=CRD42019139017

Supplementary Table 34: Gender and eye health, distribution of primary studies included in overview of systematic reviews, by topic and GBD Super-region

[a] General vision loss includes blindness ± MSVI. [b] Other causes includes dry eye disease, pterygium, epiretinal membrane, firework trauma, retinal vein occlusion, strabismus, amblyopia, asteroid hyalosis, meibomian gland dysfunction, keratoconus. [c] One review reported cataract prevalence and access to cataract services so there are 58 outcomes across 57 reviews. [d] Three additional studies could not be assigned a region.

	Southeast Asia, East Asia, Oceania	South Asia	Central Europe, Eastern Europe, Central Asia	North Africa Middle East	Sub-Saharan Africa	Latin America Caribbean	High Income	Total Primary Studies	Total Systematic Reviews
**General vision loss [a]**	17	7	2	26	21	0	15	88	3
**URE**	73	56	6	66	8	6	100	315	8
**Cataract**	56	27	2	30	1	3	19	138	4 [c]
**Glaucoma**	98	32	8	9	14	8	94	263	9
**AMD**	7	2	0	0	0	3	67	79	7
**Diabetic retinopathy**	3	3	0	0	0	0	28	34	1
**Corneal opacity**	0	0	0	0	0	0	0	0	0
**Trachoma**	2	0	0	8	14	1	0	25	1
**Other causes [b]**	176	30	1	84	10	17	163	483	18
**Differential access**	10	62	1	4	15	14	13	119	6 [c]
**Intervention to reduce inequity**	1	4	0	1	7	0	0	13	1
**Total**	445	223	20	228	90	52	499	1557 [d]	57

No. of studies
0
1
2-3
4-9
10-19
20-29
30-49
50-99
100+

Addressing inequity in cataract surgical services – a modified Delphi process

Forthcoming publication:
Cataract services for all: strategies for equitable access from a global modified Delphi process
Ramke J, Silva JC, Gichangi M, Ravilla T, Burn H, Buchan J, Welch V, Gilbert CE, Burton MJ.

A summary of the methods is provided here.

The aim of this study was to prioritise interventions that reduce inequity of cataract services.

183 participants were recruited from all GBD Super-Regions to join a two-round online modified Delphi process. Participants were nominated by a Steering Group with the aim of broad geographic inclusion.

Round 1: Participants answered open-ended questions about the most promising strategies that reduce inequity of cataract screening and cataract surgical services, and the priority groups to target with these interventions. All responses about priority groups were presented to participants in round 2. The responses on strategies were thematically analysed and presented in round 2.

Round 2: For priority groups, participants ranked on a 0 to 5 scale 1) the groups that experienced the most difficult accessing cataract services, and 2) the largest group that experiences access problems. For each of screening and surgery, participants answered a series of questions relating to the feasibility, acceptability, cost, equity and effectiveness of each strategy and ultimately ranked their top 8 strategies.

For the analysis presented here, the priority groups were identified using the average score of the two questions in round 2. The most promising strategies were identified for each of screening and surgery using the top ranked strategies in round 2. All groups and strategies presented to participants in round 2 are shown in Figure 45. The 10 highest ranked groups and strategies are listed below.

Highest ranked groups to prioritise to improve access to cataract services

1. Rural / remote / geographically isolated populations
2. Poor / low socioeconomic status
3. Those with low social support (e.g. without children, spouse, living alone, noone to accompany)
4. People with other disabilities / mobility issues / cognitive impairment (e.g. in wheelchair, dementia, hearing impaired)
5. Elderly
6. Homeless / living in shelters
7. Low education / low health literacy
8. Unemployed / In the informal sector (e.g. daily paid workers, street vendors, farmers & fishermen)
9. People with co-morbidities (e.g. systemic disease, mental illness, TB, HIV, leprosy)
10. In institutions e.g. In prison, orphanages, nursing homes

Highest ranked strategies to improve access to screening to identify operable cataract

1. Establish a primary eye care / screening program through **national policies, guidelines, budgets and plans**
2. **Strengthen skills of staff at primary level** (GPs, nurses, primary health workers, optometrists as relevant) to screen VA, detect cataract and refer in line with treatment guidelines [provide supportive supervision with effective follow-up care]
3. Establish **permanent primary eye services** closer to community level (e.g. vision centres, primary eye care centres)
4. **Eliminate out of pocket costs** for patients e.g. free screening, include eye screening in insurance coverage, provide transport, tiered pricing, cross-subsidy
5. Provide regular **outreach screening** (linked to surgical services) at community facilities to reduce the need for travel to a central facility
6. Improve **collaboration and integration** between levels of care, including referral (e.g. between primary and secondary care / between optometry and ophthalmology / between government, private & NGO sector)
7. **Raise awareness** (e..g. radio, someone who previously had surgery, women's groups) - health education & promotion - eye problems and screening / treatment options / where services are available

8. **Target screening to vulnerable/at risk groups** e.g. nursing homes, the homeless, incarcerated, refugees, people with disability, those aged 65+, newborns
9. **Improve efficiency** of public outpatient clinics / reduce waiting times / minimise the number of visits required
10. **Include screening in established community-based activities** such as trachoma trichiasis, NCD screening, elderly program, newborn screening, or traditional health programs

Highest ranked strategies to improve access to cataract surgery

1. **Decentralize services** so services are closer to people – establish **permanent surgical capacity** at district/subdistrict level and deploy cataract surgeons with appropriate infrastructure
2. Strengthen the **link between screening, referral to surgery and follow-up** (create and comply with protocols)
3. **Reduce or eliminate out of pocket costs** for surgery and post-operative care (e.g. have a subsidised option, philanthropy, insurance)
4. Provide **universal health insurance** that covers cataract surgery and post-operative care
5. Deliver consistently **good quality surgical outcomes** to maximise uptake (monitor outcomes with audit and feedback and improve where necessary)
6. **Improve efficiency** to increase surgical output and reduce waiting time & unit cost e.g. day surgery, streamline referral process to minimise number of appointments needed, specific cataract pathway/list, adequate workforce to meet population need, monitor productivity and link it to payment, twin-table theatres
7. Conduct **regular outreach surgery** away from the main eye department, linked to screening program
8. Design **services and funding mechanisms that give priority to disadvantaged groups** e.g. target services to specific groups such as women in rural areas
9. **Raise awareness** (e..g. radio, someone who previously had surgery, women's groups) - health education & promotion - eye problems and benefits of timely surgery
10. Train more **surgeons in contextually relevant surgical techniques** (e.g. MSICs) and **deploy / incentivize them** to work in rural areas / with disadvantaged populations

Supplementary Figure 19: Addressing inequity in cataract surgical services

Global results of a modified Delphi process with 183 participants. Size of the word reflects how often it was selected. Priority groups: the combination of the groups which experience the most difficulty and the groups which represent the largest number of people unable to access cataract services.

Priority groups

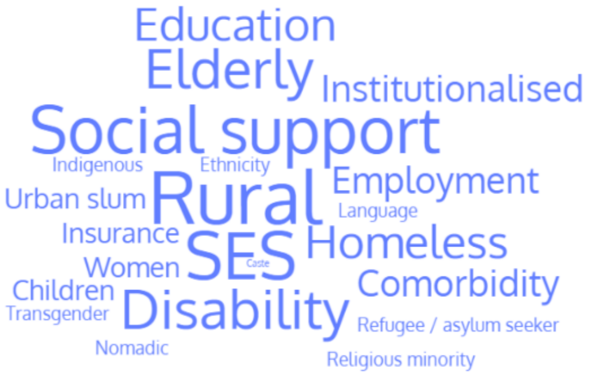

Strategies for screening

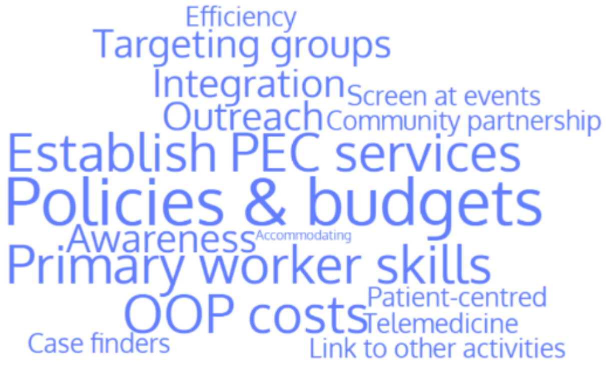

Strategies for surgery

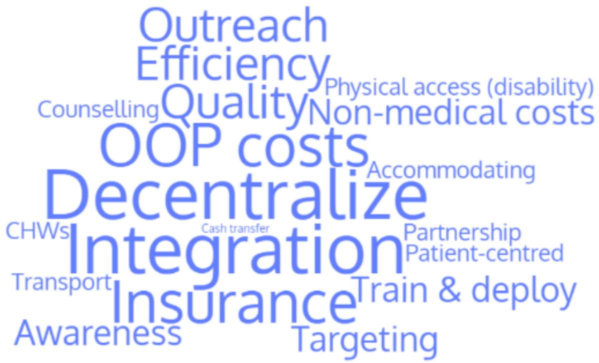

Equity, Diversity and Inclusion in global eye health leadership

This panel draws on two separate analyses. One has been published and one is forthcoming.
Published:
Action needed to improve equity and diversity in global eye health leadership
Yashadhana A, Zhang JH, Yasmin S, Morjaria P, Holland P, Faal H, Burton MJ, Ramke J.
Eye 2020;34:1051-1054.
https://doi.org/10.1038/s41433-020-0843-y

Published:
Gender and ethnic diversity in global ophthalmology and optometry association leadership: a time for change.
Yashadhana A, Clarke NA, Zhang JH, Ahmad J, Mdala S, Morjaria P, Yoshizaki M, Kyari F, Burton MJ, Ramke J.
Ophthalmic and physiological optics, 2021, In Press

The results are summarised below.

Recognising the importance of equity, diversity and inclusion in global eye health organisations, the Commission assessed the proportion of leadership positions held by women in member organisations of the International Agency for the Prevention of Blindness (IAPB), the International Council of Ophthalmology (ICO) and the World Council of Optometry (WCO).[328]

Action is clearly needed to increase the diversity of leadership in these organisations. Women were one-third or fewer of all board members or chairpersons across each of these organisations, with ICO faring better than WCO and IAPB (supplementary annex). There was large regional variation, particularly in chairpersons; women were infrequently chairpersons of IAPB member organisations outside of high-income countries. The intersection of gender and ethnic minority status was explored for the organisations based in North America, Western Europe and Australasia, where ethnic minorities are 15-40% of national populations. Across all three membership organisations, white men held more than half of all positions, outnumbering the combination of white women and ethnic-minority women and men.[328] This contrast was particularly poor in IAPB member organisations, where white men were 72% of all chairpersons, while ethnic minority women held 4% of chair positions.

Evidence from other fields suggests that global eye health organisations that place women, and people from ethnic minority and LMIC backgrounds in leadership positions are likely better placed to respond to the unequal distribution of eye health and care between and within countries.[329] This was reinforced in the *World Report on Vision,* which highlighted the need for inclusive and participatory leadership to deliver Universal Health Coverage for eye health.[9] To realise inclusive leadership, structural change is required to remove the individual and systemic biases that preclude true inclusion of women, particularly women who have other devalued intersectional identities (e.g. ethnic minority).[330] It is critical that white men in high income countries take an active, visible, and positive role as leaders and advocates for change; the work required should not primarily fall to women and other underrepresented groups.[329] The global eye health sector must establish diversity and inclusion goals and identify the most promising strategies to enable change. This analysis provides a baseline to assess our progress.

Table: Proportion of boards and chairs of member organisations of the International Council of Ophthalmology, World Council of Optometry and International Agency for the Prevention of Blindness who are women, 2020.

Position	Organisation		% women (mean) across all organisations	% women across GBD super-region		
	Name	n		Median	IQR	Range
Board member	ICO	123	33.7	37.2	26.6-38.3	19.1-43.2
	WCO	46	34.8	31.8	29.4-34.1	11.5-58.9
	IAPB	119	28.3	21.6	16.4-29.3	14.3-50.0
Chairperson	ICO	123	32.3	34.5	17.1-36.6	0-54.5
	WCO	46	21.7	15.7	3.1-23.5	0-55.6
	IAPB	90	17.8	3.6	0-17.7	0-50.0

IAPB: International Agency for the Prevention of Blindness, ICO: International Council of Ophthalmology, WCO: World Council of Ophthalmology

Political prioritisation of global eye health

Supporting material for:

Panel 6: Determinants of political priority of global eye health

In order to analyse the factors shaping global political prioritisation for eye health, we undertook archival research on the history of eye health prioritisation, reviewing documentation from the WHO, IAPB, non-governmental organisations and other actors in the eye care sector. We reviewed published reports and articles on progress-to-date and lessons learned in the sector. We also consulted minutes of meetings (such as IAPB Board Meetings and Council of Members) of individuals involved in the establishment of initiatives such as 'Vision 2020: the Right to Sight' and the 'Alliance for the Global Elimination of Trachoma by 2020'. The minutes were reviewed to capture the perspectives of eye care actors on the state of political prioritisation for global eye health.

We also undertook a scoping exercise to determine global commitments to eye health over the last 20 years. We reviewed national data as submitted by countries reporting on their progress against the Global Action Plan, noting the prevalence of national eye health coordinators, prevention of blindness committees and national eye health plans as indicators in order to evaluate resources committed and prioritisation given by different countries. Finally, we consulted with key individuals in the eye care sector, including the WHO, and incorporated their feedback on the analysis.

One limitation of the study was the ability to analyse and obtain national level data and actor input. The Global Action Plan data were incomplete such that a full global picture cannot be discerned. Another limitation was that the indicators measured do not conclusively or necessarily connote national commitment to eye health.

We would recommend further research on the national prioritisation and integration of eye health, particularly compared to other health and development initiatives. Finally, expanding the consultative process with the sector, such as through interviews, would supplement our assessment and findings.

References for Figures, Tables and Panels

References for Figure 6: Vision impairment and mortality

1. Buch H, Vinding T, la Cour M, Jensen GB, Prause JU, Nielsen NV. Age-related maculopathy: a risk indicator for poorer survival in women: the Copenhagen City Eye Study. *Ophthalmology* 2005; **112**(2): 305-12.
2. Clemons TE, Kurinij N, Sperduto RD, Group AR. Associations of mortality with ocular disorders and an intervention of high-dose antioxidants and zinc in the Age-Related Eye Disease Study: AREDS Report No. 13. *Arch Ophthalmol* 2004; **122**(5): 716-26.
3. Fisher D, Li CM, Chiu MS, et al. Impairments in hearing and vision impact on mortality in older people: the AGES-Reykjavik Study. *Age Ageing* 2014; **43**(1): 69-76.
4. Foong AW, Fong CW, Wong TY, Saw SM, Heng D, Foster PJ. Visual acuity and mortality in a chinese population. The Tanjong Pagar Study. *Ophthalmology* 2008; **115**(5): 802-7.
5. Karpa MJ, Mitchell P, Beath K, et al. Direct and indirect effects of visual impairment on mortality risk in older persons. *Arch Ophthalmol* 2009; **127**(10): 1347-53.
6. Knudtson MD, Klein BE, Klein R. Age-related eye disease, visual impairment, and survival: the Beaver Dam Eye Study. *Arch Ophthalmol* 2006; **124**(2): 243-9.
7. Lee DJ, Gomez-Marin O, Ma F, Lam BL. Distance visual acuity impairment and survival in African Americans and non-Hispanic Whites. *Ethn Dis* 2003; **13**(4): 485-91.
8. Liao H, Zhu Z, Wang H, Rong X, Young CA, Peng Y. Cognitive Performance Concomitant With Vision Acuity Predicts 13-Year Risk for Mortality. *Front Aging Neurosci* 2019; **11**: 65.
9. Loprinzi PD, Crush E. Sensory Impairment, Functional Balance and Physical Activity With All-Cause Mortality. *J Phys Act Health* 2016; **13**(9): 980-7.
10. Lott LA, Schneck ME, Haegerstrom-Portnoy G, Brabyn JA. Non-standard vision measures predict mortality in elders: the Smith-Kettlewell Institute (SKI) study. *Ophthalmic Epidemiol* 2010; **17**(4): 242-50.
11. Ng SK, Kahawita S, Andrew NH, Henderson T, Craig JE, Landers J. Association of Visual Impairment and All-Cause 10-Year Mortality Among Indigenous Australian Individuals Within Central Australia: The Central Australian Ocular Health Study. *JAMA Ophthalmol* 2018; **136**(5): 534-7.
12. Age-Related Eye Disease Study 2 Research G, Papudesu C, Clemons TE, Agron E, Chew EY. Association of Mortality with Ocular Diseases and Visual Impairment in the Age-Related Eye Disease Study 2: Age-Related Eye Disease Study 2 Report Number 13. *Ophthalmology* 2018; **125**(4): 512-21.
13. Pedula KL, Coleman AL, Hillier TA, et al. Visual acuity, contrast sensitivity, and mortality in older women: Study of osteoporotic fractures. *Journal of the American Geriatrics Society* 2006; **54**(12): 1871-7.
14. Siantar RG, Cheng CY, Gemmy Cheung CM, et al. Impact of Visual Impairment and Eye diseases on Mortality: the Singapore Malay Eye Study (SiMES). *Sci Rep* 2015; **5**: 16304.
15. Khanna RC, Murthy GV, Giridhar P, et al. Cataract, visual impairment and long-term mortality in a rural cohort in India: the Andhra Pradesh Eye Disease Study. *PLoS One* 2013; **8**(10): e78002.
16. Agrawal N, Kalaivani M, Gupta SK, Misra P, Anand K, Pandav CS. Association of blindness and hearing impairment with mortality in a cohort of elderly persons in a rural area. *Indian J Community Med* 2011; **36**(3): 208-12.
17. Crewe JM, Spilsbury K, Morlet N, et al. Health Service Use and Mortality of the Elderly Blind. *Ophthalmology* 2015; **122**(11): 2344-50.

References for Figure 13: Cost-effectiveness ratios for cataract surgery

1. Lansingh VC, Carter MJ. Use of Global Visual Acuity Data in a time trade-off approach to calculate the cost utility of cataract surgery. Arch Ophthalmol. 2009;127(9):1183-93.
2. Lansingh VC, Carter MJ, Martens M. Global cost-effectiveness of cataract surgery. Ophthalmology. 2007;114(9):1670-8.
3. Baltussen R, Sylla M, Mariotti SP. Cost-effectiveness analysis of cataract surgery: a global and regional analysis. Bulletin of the World Health Organization. 2004;82(5):338-45.
4. Khan A, Amitava AK, Rizvi SA, Siddiqui Z, Kumari N, Grover S. Cost-effectiveness analysis should continually assess competing health care options especially in high volume environments like cataract surgery. Indian J Ophthalmol. 2015;63(6):496-500.
5. Griffiths UK, Bozzani FM, Gheorghe A, Mwenge L, Gilbert C. Cost-effectiveness of eye care services in Zambia. Cost Eff Resour Alloc. 2014;12(1):6.
6. Hiratsuka Y, Yamada M, Akune Y, Murakami A, Okada AA, Yamashita H, et al. Cost-utility analysis of cataract surgery in Japan: a probabilistic Markov modeling study. Jpn J Ophthalmol. 2013;57(4):391-401.
7. Brown GC, Brown MM, Menezes A, Busbee BG, Lieske HB, Lieske PA. Cataract surgery cost utility revisited in 2012: a new economic paradigm. Ophthalmology. 2013;120(12):2367-76.
8. Busbee BG, Brown MM, Brown GC, Sharma S. Incremental cost-effectiveness of initial cataract surgery. Ophthalmology. 2002;109(3):606-12; discussion 12-3.
9. Kobelt G, Lundström M, Stenevi U. Cost-effectiveness of cataract surgery. Method to assess cost-effectiveness using registry data. J Cataract Refract Surg. 2002;28(10):1742-9.
10. Räsänen P, Krootila K, Sintonen H, Leivo T, Koivisto AM, Ryynänen OP, et al. Cost-utility of routine cataract surgery. Health Qual Life Outcomes. 2006;4:74.
11. Sach TH, Foss AJE, Gregson RM, Zaman A, Osborn F, Masud T, et al. Falls and health status in elderly women following first eye cataract surgery: an economic evaluation conducted alongside a randomised controlled trial. The British journal of ophthalmology. 2007;91(12):1675-9.

References for Figure 14: Cost-effectiveness ratios for refractive error services

1. Baltussen R, Naus J, Limburg H. Cost-effectiveness of screening and correcting refractive errors in school children in Africa, Asia, America and Europe. Health Policy. 2009;89(2):201-15.
2. Frick KD, Riva-Clement L, Shankar MB. Screening for refractive error and fitting with spectacles in rural and urban India: cost-effectiveness. Ophthalmic Epidemiol. 2009;16(6):378-87.
3. Griffiths UK, Bozzani FM, Gheorghe A, Mwenge L, Gilbert C. Cost-effectiveness of eye care services in Zambia. Cost Eff Resour Alloc. 2014;12(1):6.

References for Figure 23: Strategies to improve access to eye care for Indigenous and other non-dominant ethnic groups

Indigenous

Arora S, Kurji AK, Tennant MT. Dismantling sociocultural barriers to eye care with tele-ophthalmology: lessons from an Alberta Cree community. *Clinical & Investigative Medicine - Medecine Clinique et Experimentale* 2013;36:E57-63.

Kim J, Driver DD. Teleophthalmology for first nations clients at risk of diabetic retinopathy: a mixed methods evaluation. *JMIR Medical Informatics* 2015;3:e10.

Layland B, Holden B, Evans K, Bailey S. ICEE/AHMRC NSW Aboriginal Eye and Visioncare Program, Australia. *Rural & Remote Health* 2004;4:247.

Moynihan V, Turner A. Coordination of diabetic retinopathy screening in the Kimberley region of Western Australia. *Australian Journal of Rural Health* 2017;25:110-15.

Napper G, Fricke T, Anjou MD, Jackson AJ. Breaking down barriers to eye care for Indigenous people: a new scheme for delivery of eye care in Victoria. *Clinical & Experimental Optometry* 2015;98:430-4.

Penrose L, Roe Y, Johnson NA, James EL. Process redesign of a surgical pathway improves access to cataract surgery for Aboriginal and Torres Strait Islander people in South East Queensland. *Australian Journal of Primary Health* 2018;24:135-40.

Spurling GK, Askew DA, Hayman NE, Hansar N, Cooney AM, Jackson CL. Retinal photography for diabetic retinopathy screening in Indigenous primary health care: the Inala experience. *Australian & New Zealand Journal of Public Health* 2010;34 Suppl 1:S30-3.

Spurr S, Bullin C, Bally J, Trinder K, Khan S. Nurse-led diabetic retinopathy screening: a pilot study to evaluate a new approach to vision care for Canadian Aboriginal peoples. *International Journal of Circumpolar Health* 2018;77:1422670.

Ethnic Minority

Al-Aswad LA, Joiner DB, Wang X, et al. Screening for glaucoma in populations at high risk: The eye screening New York project. *Cogent Medicine* 2017;4 (1):14.

Anderson RM, Musch DC, Nwankwo RB, et al. Eye screening can prevent eye disease. Personalized follow-up increases return rate at urban eye disease screening clinics for African Americans with diabetes: Results of a randomized trial. *Ethnicity and Disease* 2003;13:149.

Baker SB, Vallbona C, Pavlik V, et al. A diabetes control program in a public health care setting. *Public Health Reports* 1993;108:595-605.

Bush K, Thomas R, Raymond NT, Sankar S, Barker PJ, O'Hare JP. Cluster randomised controlled trial evaluation of a Link Worker-delivered intervention to improve uptake of diabetic retinopathy screening in a South Asian population. *Diabetes & Vascular Disease Research* 2014;11:294-97.

Frazier M, De La Cruz N, Garces IC. Development of educational materials to improve rates of early eye care for Hispanic children. *Journal of Immigrant & Minority Health* 2012;14:608-16.

Jani PD, Forbes L, McDaniel P, Viera A, Garg S. Geographic Information Systems Mapping of Diabetic Retinopathy in an Ocular Telemedicine Network. *JAMA Ophthalmology* 2017;135:715-21.

Jones HL, Walker EA, Schechter CB, Blanco E. Vision is precious: a successful behavioral intervention to increase the rate of screening for diabetic retinopathy for inner-city adults. *Diabetes Educator* 2010;36:118-26.

Nesher R, Ever-Hadani P, Epstein E, Stern Y, Assia E. Overcoming the language barrier in visual field testing. *Journal of Glaucoma* 2001;10:203-05.

Pizzi LT, Waisbourd M, Hark L, et al. Costs of a community-based glaucoma detection programme: analysis of the Philadelphia Glaucoma Detection and Treatment Project. *British Journal of Ophthalmology* 2018;102:225-32.

Rodriguez E, Srivastava A, Landau M. Increasing Screening Follow-Up for Vulnerable Children: A Partnership with School Nurses. *International Journal of Environmental Research & Public Health [Electronic Resource]* 2018;15:25.

Rovner BW, Casten RJ. Trust and Glycemic Control in Black Patients With Diabetic Retinopathy: A Pilot Study. *Diabetes Spectrum* 2019;32:152-55.

Shahid K, Kolomeyer AM, Nayak NV, et al. Ocular telehealth screenings in an urban community. *Telemedicine Journal & E-Health* 2012;18:95-100.

Tjiam AM, Holtslag G, Van Minderhout HM, et al. Randomised comparison of three tools for improving compliance with occlusion therapy: an educational cartoon story, a reward calendar, and an information leaflet for parents. *Graefes Archive for Clinical & Experimental Ophthalmology* 2013;251:321-9.

Tjiam AM, Holtslag G, Vukovic E, et al. An educational cartoon accelerates amblyopia therapy and improves compliance, especially among children of immigrants. *Ophthalmology* 2012;119:2393-401.

Vaughan EM, Johnston CA, Cardenas VJ, Moreno JP, Foreyt JP. Integrating CHWs as Part of the Team Leading Diabetes Group Visits: A Randomized Controlled Feasibility Study. *Diabetes Educator* 2017;43:589-99.

Walker EA, Schechter CB, Caban A, Basch CE. Telephone intervention to promote diabetic retinopathy screening among the urban poor. *American Journal of Preventive Medicine* 2008;34:185-91.

Winters D, Casten R, Rovner B, et al. Cost-Effectiveness of Behavior Activation Versus Supportive Therapy on Adherence to Eye Exams in Older African Americans With Diabetes. *American Journal of Medical Quality* 2017;32:661-67.

Zhao D, Guallar E, Bowie JV, et al. Improving Follow-up and Reducing Barriers for Eye Screenings in Communities: The SToP Glaucoma Study. *American Journal of Ophthalmology* 2018;188:19-28.

References for Table 2: Eye health and the Sustainable Development Goals

1. Reddy PA, Congdon N, MacKenzie G, et al. Effect of providing near glasses on productivity among rural Indian tea workers with presbyopia (PROSPER): a randomised trial. *Lancet Glob Health* 2018; 6(9): e1019-e27.
2. Danquah L, Kuper H, Eusebio C, et al. The long term impact of cataract surgery on quality of life, activities and poverty: results from a six year longitudinal study in Bangladesh and the Philippines. *PLoS One* 2014; 9(4): e94140.
3. Kuper H, Polack S, Mathenge W, et al. Does cataract surgery alleviate poverty? Evidence from a multi-centre intervention study conducted in Kenya, the Philippines and Bangladesh. *PLoS One* 2010; 5(11): e15431.
4. Finger RP, Kupitz DG, Fenwick E, et al. The impact of successful cataract surgery on quality of life, household income and social status in South India. *PLoS One* 2012; 7(8): e44268.
5. Essue BM, Li Q, Hackett ML, et al. A multicenter prospective cohort study of quality of life and economic outcomes after cataract surgery in Vietnam: the VISIONARY study. *Ophthalmology* 2014; 121(11): 2138-46.
6. Glick P, Luoto J, Orrs MS, et al. The individual and household impacts of cataract surgery on older blind adults in ethiopia. *Ophthalmic Epidemiol* 2019; 26(1): 7-18.
7. Fireison CK, Moore JE. Employment outcomes and educational backgrounds of legally blind adults employed in sheltered industrial settings. *Journal of Visual Impairment and Blindness* 1998; 92(11): 740-7.
8. Giesen JM, Hierholzer A. Vocational rehabilitation services and employment for SSDI beneficiaries with visual impairments. *Journal of Vocational Rehabilitation* 2016; 44(2): 175-89.
9. Brown GC, Brown MM, Menezes A, Busbee BG, Lieske HB, Lieske PA. Cataract surgery cost utility revisited in 2012: a new economic paradigm. *Ophthalmology* 2013; 120(12): 2367-76.
10. Haynes R, Gale S, Mugford M, Davies P. Cataract surgery in a community hospital outreach clinic: patients' costs and satisfaction. *Soc Sci Med* 2001; 53(12): 1631-40.
11. Frick KD, Hanson CL, Jacobson GA. Global burden of trachoma and economics of the disease. *The American journal of tropical medicine and hygiene* 2003; 69(5 Suppl): 1-10.
12. Frick KD, Keuffel EL, Bowman RJ. Epidemiological, demographic, and economic analyses: measurement of the value of trichiasis surgery in The Gambia. *Ophthalmic Epidemiol* 2001; 8(2-3): 191-201.
13. Glewwe P, Park A, Zhao M. A better vision for development: Eyeglasses and academic performance in rural primary schools in China. *Journal of Development Economics* 2016; (122): 170-82.
14. Glewwe P, West KL, Lee J. The Impact of Providing Vision Screening and Free Eyeglasses on Academic Outcomes: Evidence from a Randomized Trial in Title I Elementary Schools in Florida. *J Policy Anal Manage* 2018; 37(2): 265-300.
15. Hannum E, Zhang Y. Poverty and Proximate Barriers to Learning: Vision Deficiencies, Vision Correction and Educational Outcomes in Rural Northwest China. *World Dev* 2012; 40(9): 1921-31.
16. Ma X, Zhou Z, Yi H, et al. Effect of providing free glasses on children's educational outcomes in China: cluster randomized controlled trial. *BMJ* 2014; 349: g5740.
17. Ma Y, Congdon N, Shi Y, et al. Effect of a Local Vision Care Center on Eyeglasses Use and School Performance in Rural China: A Cluster Randomized Clinical Trial. *JAMA Ophthalmol* 2018; 136(7): 731-7.
18. Hark LA, Thau A, Nutaitis A, et al. Impact of eyeglasses on academic performance in primary school children. *Can J Ophthalmol* 2020; 55(1): 52-7.
19. Joseph L. Refractive Errors and Academic Achievements of Primary School Children. *Nurs J India* 2014; 105(6): 269-71.
20. Bruce A, Kelly B, Chambers B, et al. The effect of adherence to spectacle wear on early developing literacy: a longitudinal study based in a large multiethnic city, Bradford, UK. *BMJ open* 2018; 8(6): e021277.
21. Mercer GD, Lyons P, Bassett K. Interventions to improve gender equity in eye care in low-middle income countries: A systematic review. *Ophthalmic Epidemiol* 2019; 26(3): 189-99.
22. Baruwa E, Tzu J, Congdon N, He M, Frick KD. Reversal in gender valuations of cataract surgery after the implementation of free screening and low-priced high-quality surgery in a rural population of southern China. *Ophthalmic Epidemiol* 2008; 15(2): 99-104.
23. Dickey H, Ikenwilo D, Norwood P, Watson V, Zangelidis A. Utilisation of eye-care services: the effect of Scotland's free eye examination policy. *Health Policy* 2012; 108(2-3): 286-93.
24. Subzwari S, Desapriya E, Scime G, Babul S, Jivani K, Pike I. Effectiveness of cataract surgery in reducing driving-related difficulties: a systematic review and meta-analysis. *Inj Prev* 2008; 14(5): 324-8.
25. Owsley C, McGwin G, Jr., Sloane M, Wells J, Stalvey BT, Gauthreaux S. Impact of cataract surgery on motor vehicle crash involvement by older adults. *JAMA* 2002; 288(7): 841-9.
26. Meuleners LB, Brameld K, Fraser ML, Chow K. The impact of first- and second-eye cataract surgery on motor vehicle crashes and associated costs. *Age Ageing* 2019; 48(1): 128-33.
27. Meuleners LB, Hendrie D, Lee AH, Ng JQ, Morlet N. The effectiveness of cataract surgery in reducing motor vehicle crashes: a whole population study using linked data. *Ophthalmic Epidemiol* 2012; 19(1): 23-8.
28. Meuleners LB, Ng JQ, Fraser M, Hendrie D, Morlet N. Impact of gender on first eye cataract surgery and motor vehicle crash risk for older drivers. *Clin Exp Ophthalmol* 2012; 40(6): 591-6.
29. Schlenker MB, Thiruchelvam D, Redelmeier DA. Association of Cataract Surgery With Traffic Crashes. *JAMA Ophthalmol* 2018; 136(9): 998-1007.

References for Table 5: Population-based studies reporting Effective Refractive Error Coverage (eREC)

1. Chan VF, Mebrahtu G, Ramson P, Wepo M, Naidoo KS. Prevalence of refractive error and spectacle coverage in Zoba Ma'ekel Eritrea: a rapid assessment of refractive error. *Ophthalmic Epidemiol* 2013; 20(3): 131-7.
2. Ezelum C, Razavi H, Sivasubramaniam S, et al. Refractive error in Nigerian adults: prevalence, type, and spectacle coverage. *Invest Ophthalmol Vis Sci* 2011; 52(8): 5449-56.
3. Mashayo ER, Chan VF, Ramson P, Chinanayi F, Naidoo KS. Prevalence of refractive error, presbyopia and spectacle coverage in Kahama District, Tanzania: a rapid assessment of refractive error. *Clin Exp Optom* 2015; 98(1): 58-64.
4. Naidoo KS, Chinanayi FS, Ramson P, Mashige KP. Rapid assessment of refractive error in the eThekwini Municipality of KwaZulu-Natal, Durban, South Africa. *Clin Exp Optom* 2016; 99(4): 360-5.
5. Nsubuga N, Ramson P, Govender P, Chan V, Wepo M, Naidoo KS. Uncorrected refractive errors, presbyopia and spectacle coverage in Kamuli District, Uganda. *African Vis Eye Health* 2016; 75: a327.
6. Loughman J, Nxele LL, Faria C. Rapid assessment of refractive error, presbyopia, and visual impairment and associated quality of life in Nampula, Mozambique. *J Vis Impair Blind* 2015; 109: 199-212.
7. Casas Luque L, Naidoo K, Chan VF, et al. Prevalence of Refractive Error, Presbyopia, and Spectacle Coverage in Bogota, Colombia: A Rapid Assessment of Refractive Error. *Optom Vis Sci* 2019; 96(8): 579-86.
8. Fotouhi A, Hashemi H, Raissi B, Mohammad K. Uncorrected refractive errors and spectacle utilisation rate in Tehran: the unmet need. *Br J Ophthalmol* 2006; 90(5): 534-7.
9. Bourne RR, Dineen BP, Huq DM, Ali SM, Johnson GJ. Correction of refractive error in the adult population of Bangladesh: meeting the unmet need. *Invest Ophthalmol Vis Sci* 2004; 45(2): 410-7.
10. Ramke J, du Toit R, Palagyi A, Brian G, Naduvilath T. Correction of refractive error and presbyopia in Timor-Leste. *Br J Ophthalmol* 2007; 91(7): 860-6.
11. Shah SP, Jadoon MZ, Dineen B, et al. Refractive errors in the adult pakistani population: the national blindness and visual impairment survey. *Ophthalmic Epidemiol* 2008; 15(3): 183-90.
12. Foreman J, Xie J, Keel S, Taylor HR, Dirani M. Treatment coverage rates for refractive error in the National Eye Health survey. *PLoS One* 2017; 12(4): e0175353.

References for Panel 1: Vision impairment and dementia

1. GBD Dementia Collaborators. Global, regional, and national burden of Alzheimer's disease and other dementias, 1990-2016: a systematic analysis for the Global Burden of Disease Study 2016. *Lancet Neurol* 2019; 18(1): 88-106.
2. Prince M, Bryce R, Albanese E, Wimo A, Ribeiro W, Ferri CP. The global prevalence of dementia: a systematic review and metaanalysis. *Alzheimers Dement* 2013; 9(1): 63-75 e2.
3. Wimo A, Guerchet M, Ali GC, et al. The worldwide costs of dementia 2015 and comparisons with 2010. *Alzheimers Dement* 2017; 13(1): 1-7.
4. Mukadam N, Sommerlad A, Huntley J, Livingston G. Population attributable fractions for risk factors for dementia in low-income and middle-income countries: an analysis using cross-sectional survey data. *Lancet Glob Health* 2019; 7(5): e596-e603.
5. Ehrlich JR, Langa KM. Potential for dementia prevention in Latin America and Africa based on population-attributable fraction estimates. *Lancet Glob Health* 2019; 7(10): e1323.
6. Swenor BK, Wang J, Varadaraj V, et al. Vision Impairment and Cognitive Outcomes in Older Adults: The Health ABC Study. *J Gerontol A Biol Sci Med Sci* 2019; 74(9): 1454-60.
7. Zheng DD, Swenor BK, Christ SL, West SK, Lam BL, Lee DJ. Longitudinal Associations Between Visual Impairment and Cognitive Functioning: The Salisbury Eye Evaluation Study. *JAMA Ophthalmol* 2018; 136(9): 989-95.
8. Brenowitz WD, Kaup AR, Lin FR, Yaffe K. Multiple Sensory Impairment Is Associated With Increased Risk of Dementia Among Black and White Older Adults. *J Gerontol A Biol Sci Med Sci* 2019; 74(6): 890-6.
9. Fischer ME, Cruickshanks KJ, Schubert CR, et al. Age-Related Sensory Impairments and Risk of Cognitive Impairment. *Journal of the American Geriatrics Society* 2016; 64(10): 1981-7.
10. Schubert CR, Cruickshanks KJ, Fischer ME, et al. Sensory Impairments and Cognitive Function in Middle-Aged Adults. *J Gerontol A Biol Sci Med Sci* 2017; 72(8): 1087-90.
11. Rogers MA, Langa KM. Untreated poor vision: a contributing factor to late-life dementia. *Am J Epidemiol* 2010; 171(6): 728-35.
12. Nael V, Peres K, Dartigues JF, et al. Vision loss and 12-year risk of dementia in older adults: the 3C cohort study. *Eur J Epidemiol* 2019; 34(2): 141-52.
13. Maharani A, Dawes P, Nazroo J, Tampubolon G, Pendleton N, group SE-CW. Cataract surgery and age-related cognitive decline: A 13-year follow-up of the English Longitudinal Study of Ageing. *PLoS One* 2018; 13(10): e0204833.
14. Tran EM, Stefanick ML, Henderson VW, et al. Association of Visual Impairment With Risk of Incident Dementia in a Women's Health Initiative Population. *JAMA Ophthalmol* 2020.
15. Lim ZW, Chee ML, Da Soh Z, et al. Association Between Visual Impairment and Decline in Cognitive Function in a Multiethnic Asian Population. *JAMA Netw Open* 2020; 3(4): e203560.
16. Lee J, Banerjee J, Khobragade PY, Angrisani M, Dey AB. LASI-DAD study: a protocol for a prospective cohort study of late-life cognition and dementia in India. *BMJ open* 2019; 9(7): e030300.
17. Kowal P, Chatterji S, Naidoo N, et al. Data resource profile: the World Health Organization Study on global AGEing and adult health (SAGE). *Int J Epidemiol* 2012; 41(6): 1639-49.
18. Whitson HE, Cronin-Golomb A, Cruickshanks KJ, et al. American Geriatrics Society and National Institute on Aging Bench-to-Bedside Conference: Sensory Impairment and Cognitive Decline in Older Adults. *Journal of the American Geriatrics Society* 2018; 66(11): 2052-8.
19. Chan VTT, Sun Z, Tang S, et al. Spectral-Domain OCT Measurements in Alzheimer's Disease: A Systematic Review and Meta-analysis. *Ophthalmology* 2019; 126(4): 497-510.
20. Pigeon C, Li T, Moreau F, Pradel G, Marin-Lamellet C. Cognitive load of walking in people who are blind: Subjective and objective measures for assessment. *Gait Posture* 2019; 67: 43-9.
21. Tan ZS, Spartano NL, Beiser AS, et al. Physical Activity, Brain Volume, and Dementia Risk: The Framingham Study. *J Gerontol A Biol Sci Med Sci* 2017; 72(6): 789-95.
22. Reas ET, Laughlin GA, Bergstrom J, Kritz-Silverstein D, McEvoy LK. Physical Activity and Trajectories of Cognitive Change in Community-Dwelling Older Adults: The Rancho Bernardo Study. *J Alzheimers Dis* 2019; 71(1): 109-18.
23. Rafnsson SB, Orrell M, d'Orsi E, Hogervorst E, Steptoe A. Loneliness, Social Integration, and Incident Dementia Over 6 Years: Prospective Findings From the English Longitudinal Study of Ageing. *J Gerontol B Psychol Sci Soc Sci* 2020; 75(1): 114-24.
24. Livingstone G. Dementia prevention, intervention, and care: 2020 report of the Lancet Commission. *Lancet* 2020.

References for Panel 2: The changing epidemiology of eye disease

1. Holden BA, Fricke TR, Wilson DA, et al. Global Prevalence of Myopia and High Myopia and Temporal Trends from 2000 through 2050. *Ophthalmology* 2016; 123(5): 1036-42.
2. World Health Organization. The impact of myopia and high myopia: report of the Joint World Health Organization–Brien Holden Vision Institute Global Scientific Meeting on Myopia, University of New South Wales, Sydney, Australia. Geneva: World Health Organization, 2015.
3. GBD 2019 Blindness and Vision Impairment Collaborators, on behalf of the Vision Loss Expert Group of the Global Burden of Disease Study. Trends in prevalence of blindness and distance and near vision impairment over 30 years: an analysis for the Global Burden of Disease Study. *Lancet Glob Health* 2020; published online Dec 1. https://doi.org/10.1016/S2214-109X(20)30425-3.
4. Naidoo KS, Fricke TR, Frick KD, et al. Potential Lost Productivity Resulting from the Global Burden of Myopia: Systematic Review, Meta-analysis, and Modeling. *Ophthalmology* 2019; 126(3): 338-46.
5. Koh V, Yang A, Saw SM, et al. Differences in prevalence of refractive errors in young Asian males in Singapore between 1996-1997 and 2009-2010. *Ophthalmic Epidemiol* 2014; 21(4): 247-55.
6. Vitale S, Ellwein L, Cotch MF, Ferris FL, 3rd, Sperduto R. Prevalence of refractive error in the United States, 1999-2004. *Arch Ophthalmol* 2008; 126(8): 1111-9.
7. Morgan IG, French AN, Ashby RS, et al. The epidemics of myopia: Aetiology and prevention. *Prog Retin Eye Res* 2018; 62: 134-49.
8. Tedja MS, Haarman AEG, Meester-Smoor MA, et al. IMI - Myopia Genetics Report. *Invest Ophthalmol Vis Sci* 2019; 60(3): M89-M105.
9. Morgan IG, Rose KA. Myopia: is the nature-nurture debate finally over? *Clin Exp Optom* 2019; 102(1): 3-17.
10. Siddiqi A, Hertzman C. Economic growth, income equality, and population health among the Asian Tigers. *Int J Health Serv* 2001; 31(2): 323-33.
11. Sun HP, Li A, Xu Y, Pan CW. Secular trends of reduced visual acuity from 1985 to 2010 and disease burden projection for 2020 and 2030 among primary and secondary school students in China. *JAMA Ophthalmol* 2015; 133(3): 262-8.
12. Liu R, Guo X, Xiao O, et al. DIFFUSE CHORIORETINAL ATROPHY IN CHINESE HIGH MYOPIA: The ZOC-BHVI High Myopia Cohort Study. *Retina* 2020; 40(2): 241-8.
13. Fricke TR, Jong M, Naidoo KS, et al. Global prevalence of visual impairment associated with myopic macular degeneration and temporal trends from 2000 through 2050: systematic review, meta-analysis and modelling. *Br J Ophthalmol* 2018; 102(7): 855-62.
14. Dirani M, Crowston JG, Wong TY. From reading books to increased smart device screen time. *Br J Ophthalmol* 2019; 103(1): 1-2.
15. Huang HM, Chang DS, Wu PC. The Association between Near Work Activities and Myopia in Children-A Systematic Review and Meta-Analysis. *PLoS One* 2015; 10(10): e0140419.
16. Rose KA, Morgan IG, Smith W, Burlutsky G, Mitchell P, Saw SM. Myopia, lifestyle, and schooling in students of Chinese ethnicity in Singapore and Sydney. *Arch Ophthalmol* 2008; 126(4): 527-30.
17. Wen L, Cao Y, Cheng Q, et al. Objectively measured near work, outdoor exposure and myopia in children. *Br J Ophthalmol* 2020.
18. Wu PC, Chen CT, Lin KK, et al. Myopia Prevention and Outdoor Light Intensity in a School-Based Cluster Randomized Trial. *Ophthalmology* 2018; 125(8): 1239-50.
19. Wildsoet CF, Chia A, Cho P, et al. IMI - Interventions Myopia Institute: Interventions for Controlling Myopia Onset and Progression Report. *Invest Ophthalmol Vis Sci* 2019; 60(3): M106-M31.
20. Walline JJ, Lindsley KB, Vedula SS, et al. Interventions to slow progression of myopia in children. *Cochrane Database Syst Rev* 2020; 1: CD004916.
21. Cheung CMG, Arnold JJ, Holz FG, et al. Myopic Choroidal Neovascularization: Review, Guidance, and Consensus Statement on Management. *Ophthalmology* 2017; 124(11): 1690-711.
22. Ang M, Flanagan JL, Wong CW, et al. Review: Myopia control strategies recommendations from the 2018 WHO/IAPB/BHVI Meeting on Myopia. *Br J Ophthalmol* 2020.
23. Wong TY, Cheung CM, Larsen M, Sharma S, Simo R. Diabetic retinopathy. *Nat Rev Dis Primers* 2016; 2: 16012.
24. Saeedi P, Petersohn I, Salpea P, et al. Global and regional diabetes prevalence estimates for 2019 and projections for 2030 and 2045: Results from the International Diabetes Federation Diabetes Atlas, 9(th) edition. *Diabetes Res Clin Pract* 2019; 157: 107843.
25. World Health Organization. Global Report on diabetes. Geneva: World Health Organization, 2016.
26. Manne-Goehler J, Geldsetzer P, Agoudavi K, et al. Health system performance for people with diabetes in 28 low- and middle-income countries: A cross-sectional study of nationally representative surveys. *PLoS Med* 2019; 16(3): e1002751.
27. Flaxman SR, Bourne RRA, Resnikoff S, et al. Global causes of blindness and distance vision impairment 1990-2020: a systematic review and meta-analysis. *Lancet Glob Health* 2017; 5(12): e1221-e34.
28. Leasher JL, Bourne RR, Flaxman SR, et al. Global Estimates on the Number of People Blind or Visually Impaired by Diabetic Retinopathy: A Meta-analysis From 1990 to 2010. *Diabetes Care* 2016; 39(9): 1643-9.
29. Yau JW, Rogers SL, Kawasaki R, et al. Global prevalence and major risk factors of diabetic retinopathy. *Diabetes Care* 2012; 35(3): 556-64.
30. Klein R, Klein BE, Moss SE, Davis MD, DeMets DL. The Wisconsin epidemiologic study of diabetic retinopathy. II. Prevalence and risk of diabetic retinopathy when age at diagnosis is less than 30 years. *Arch Ophthalmol* 1984; 102(4): 520-6.
31. Klein R, Klein BE, Moss SE, Davis MD, DeMets DL. The Wisconsin epidemiologic study of diabetic retinopathy. III. Prevalence and risk of diabetic retinopathy when age at diagnosis is 30 or more years. *Arch Ophthalmol* 1984; 102(4): 527-32.

32. Sabanayagam C, Banu R, Chee ML, et al. Incidence and progression of diabetic retinopathy: a systematic review. *Lancet Diabetes Endocrinol* 2019; 7(2): 140-9.
33. Sabanayagam C, Yip W, Ting DS, Tan G, Wong TY. Ten Emerging Trends in the Epidemiology of Diabetic Retinopathy. *Ophthalmic Epidemiol* 2016; 23(4): 209-22.
34. Diabetes_Control_and_Complications_Trial_Research_Group. The effect of intensive treatment of diabetes on the development and progression of long-term complications in insulin-dependent diabetes mellitus. *N Engl J Med* 1993; 329(14): 977-86.
35. Intensive blood-glucose control with sulphonylureas or insulin compared with conventional treatment and risk of complications in patients with type 2 diabetes (UKPDS 33). UK Prospective Diabetes Study (UKPDS) Group. *Lancet* 1998; 352(9131): 837-53.
36. Tight blood pressure control and risk of macrovascular and microvascular complications in type 2 diabetes: UKPDS 38. UK Prospective Diabetes Study Group. *BMJ* 1998; 317(7160): 703-13.
37. Do DV, Wang X, Vedula SS, et al. Blood pressure control for diabetic retinopathy. *Cochrane Database Syst Rev* 2015; 1: CD006127.
38. Arar NH, Freedman BI, Adler SG, et al. Heritability of the severity of diabetic retinopathy: the FIND-Eye study. *Invest Ophthalmol Vis Sci* 2008; 49(9): 3839-45.
39. Huang YC, Lin JM, Lin HJ, et al. Genome-wide association study of diabetic retinopathy in a Taiwanese population. *Ophthalmology* 2011; 118(4): 642-8.
40. Kowluru RA, Kowluru A, Mishra M, Kumar B. Oxidative stress and epigenetic modifications in the pathogenesis of diabetic retinopathy. *Prog Retin Eye Res* 2015; 48: 40-61.
41. Vujosevic S, Aldington SJ, Silva P, et al. Screening for diabetic retinopathy: new perspectives and challenges. *Lancet Diabetes Endocrinol* 2020; 8(4): 337-47.
42. Wong TY, Sun J, Kawasaki R, et al. Guidelines on Diabetic Eye Care: The International Council of Ophthalmology Recommendations for Screening, Follow-up, Referral, and Treatment Based on Resource Settings. *Ophthalmology* 2018; 125(10): 1608-22.
43. World Health Organization. Best buys' and other recommended interventions for the prevention and control of non-communicable diseases. Updated 2017. Appendix 3 of the Global Action Plan for the prevention and control of non-communicable diseases 2013-2020. Geneva: World Health Organization, 2017.
44. Poore S, Foster A, Zondervan M, Blanchet K. Planning and developing services for diabetic retinopathy in Sub-Saharan Africa. *Int J Health Policy Manag* 2015; 4(1): 19-28.
45. Burgess PI, Msukwa G, Beare NA. Diabetic retinopathy in sub-Saharan Africa: meeting the challenges of an emerging epidemic. *BMC Med* 2013; 11: 157.
46. Denniston AK, Lee AY, Lee CS, et al. United Kingdom Diabetic Retinopathy Electronic Medical Record (UK DR EMR) Users Group: report 4, real-world data on the impact of deprivation on the presentation of diabetic eye disease at hospital services. *Br J Ophthalmol* 2019; 103(6): 837-43.
47. Foreman J, Keel S, Xie J, Van Wijngaarden P, Taylor HR, Dirani M. Adherence to diabetic eye examination guidelines in Australia: the National Eye Health Survey. *Med J Aust* 2017; 206(9): 402-6.
48. Global, regional, and national incidence, prevalence, and years lived with disability for 354 diseases and injuries for 195 countries and territories, 1990-2017: a systematic analysis for the Global Burden of Disease Study 2017. *Lancet* 2018; 392(10159): 1789-858.
49. World Health Organization. Eliminating river blindness: making a difference - 30 years of Research and Capacity Building in Tropical Diseases. Geneva: World Health Organization, 2008.
50. Bourne RR, Stevens GA, White RA, et al. Causes of vision loss worldwide, 1990-2010: a systematic analysis. *Lancet Glob Health* 2013; 1(6): e339-49.
51. Tang FF, Chang HL, Huang YT, Wang KC. Studies on the etiology of trachoma with special reference to isolation of the virus in chick embryo. *Chin Med J* 1957; 75(6): 429-47.
52. Bailey RL, Arullendran P, Whittle HC, Mabey DC. Randomised controlled trial of single-dose azithromycin in treatment of trachoma. *Lancet* 1993; 342(8869): 453-6.
53. World Health Organization. Future approaches to trachoma control: report of a Global Scientific Meeting, Geneva, 17-20 June 1996 (WHO/PBL/96.56). Geneva: World Health Organization, 1997.
54. World Health Organization. Global initiative for the elimination of avoidable blindness (WHO/PBL/97.61 Rev 2). Geneva: World Health Organization, 2000.
55. World Health Organization. A framework and indicators for monitoring VISION 2020 – The Right to Sight: The Global Initiative for the Elimination of Avoidable Blindness. Geneva: World Health Organization, 2003.
56. World Health Organization. WHO Alliance for the Global Elimination of Trachoma by 2020: progress report, 2019. *Wkly Epidemiol Rec* 2020; 30(95): 349-60.
57. World Health Organization. Global Health Observatory data repository: Status of elimination of trachoma as a public health problem, data by country [available at: https://apps.who.int/gho/data/node.main.A1645T?lang=en; accessed 08 April 2020]; 2020.
58. World Health Assembly. Global elimination of blinding trachoma. WHA51.11. 1998. https://www.who.int/blindness/causes/WHA51.11/en/ (accessed 24/07/2020).
59. Courtright P, Rotondo LA, MacArthur C, et al. Strengthening the links between mapping, planning and global engagement for disease elimination: lessons learnt from trachoma. *Br J Ophthalmol* 2018; 102(10): 1324-7.
60. Solomon AW, Pavluck A, Courtright P, et al. The Global Trachoma Mapping Project: methodology of a 34-country population-based study. *Ophthalmic Epidemiol* 2015; 22(3): 214-25.
61. Solomon AW, Willis R, Pavluck AL, et al. Quality Assurance and Quality Control in the Global Trachoma Mapping Project. *Am J Trop Med Hyg* 2018; 99(4): 858-63.
62. World Health Organization. Network of WHO Collaborating Centres for Trachoma: 2nd meeting report, Decatur, GA, USA, 26 June 2016 (WHO/HTM/NTD/PCT/2017.06). Geneva: World Health Organization, 2017.

63. Solomon AW, Le Mesurier RT, Williams WJ. A diagnostic instrument to help field graders evaluate active trachoma. *Ophthalmic Epidemiol* 2018: 1-4.
64. Gower EW, Kello AB, Kollmann KHM. Training trichiasis surgeons: ensuring quality. *Community Eye Health* 2014; 27(87): 58.
65. Merbs SL, Kello AB, Gelema H, West SK, Gower EW. The trachomatous trichiasis clamp: a surgical instrument designed to improve bilamellar tarsal rotation procedure outcomes. *Arch Ophthalmol* 2012; 130(2): 220-3.
66. London School of Hygiene & Tropical Medicine. Eliminating Trachoma: Discover how communities and experts are joining together to end trachoma disease across over 44 endemic countries [available at: https://www.lshtm.ac.uk/study/courses/short-courses/free-online-courses/eliminating-trachoma, accessed 08 April 2020]. 2020.
67. World Health Organization. Ending the neglect to attain the Sustainable Development Goals: a road map for neglected tropical diseases 2021–2030 (draft). Geneva: World Health Organization, 2020.
68. Habtamu E, Wondie T, Aweke S, et al. Posterior lamellar versus bilamellar tarsal rotation surgery for trachomatous trichiasis in Ethiopia: a randomised controlled trial. *Lancet Glob Health* 2016; 4(3): e175-84.
69. Last A, Versteeg B, Shafi Abdurahman O, et al. Detecting extra-ocular Chlamydia trachomatis in a trachoma-endemic community in Ethiopia: Identifying potential routes of transmission. *PLoS Negl Trop Dis* 2020; 14(3): e0008120.

References for Panel 5: The challenge of glaucoma

1. GBD 2019 Blindness and Vision Impairment Collaborators, on behalf of the Vision Loss Expert Group of the Global Burden of Disease Study. Trends in prevalence of blindness and distance and near vision impairment over 30 years: an analysis for the Global Burden of Disease Study. *Lancet Glob Health* 2020; published online Dec 1. https://doi.org/10.1016/S2214-109X(20)30425-3.
2. Tham YC, Li X, Wong TY, Quigley HA, Aung T, Cheng CY. Global prevalence of glaucoma and projections of glaucoma burden through 2040: a systematic review and meta-analysis. *Ophthalmology* 2014; 121(11): 2081-90.
3. Vijaya L, George R, Baskaran M, et al. Prevalence of primary open-angle glaucoma in an urban south Indian population and comparison with a rural population. The Chennai Glaucoma Study. *Ophthalmology* 2008; 115(4): 648-54 e1.
4. Kyari F, Entekume G, Rabiu M, et al. A Population-based survey of the prevalence and types of glaucoma in Nigeria: results from the Nigeria National Blindness and Visual Impairment Survey. *BMC Ophthalmol* 2015; 15: 176.
5. Ramakrishnan R, Nirmalan PK, Krishnadas R, et al. Glaucoma in a rural population of southern India: the Aravind comprehensive eye survey. *Ophthalmology* 2003; 110(8): 1484-90.
6. Buhrmann RR, Quigley HA, Barron Y, West SK, Oliva MS, Mmbaga BB. Prevalence of glaucoma in a rural East African population. *Invest Ophthalmol Vis Sci* 2000; 41(1): 40-8.
7. Kyari F, Abdull MM, Bastawrous A, Gilbert CE, Faal H. Epidemiology of glaucoma in sub-saharan Africa: prevalence, incidence and risk factors. *Middle East Afr J Ophthalmol* 2013; 20(2): 111-25.
8. Abdull MM, Gilbert CC, Evans J. Primary open angle glaucoma in northern Nigeria: stage at presentation and acceptance of treatment. *BMC Ophthalmol* 2015; 15: 111.
9. World Health Organization. Global initiative for the elimination of avoidable blindness (WHO/PBL/97.61 Rev 2). Geneva: World Health Organization, 2000.
10. Garway-Heath DF, Crabb DP, Bunce C, et al. Latanoprost for open-angle glaucoma (UKGTS): a randomised, multicentre, placebo-controlled trial. *Lancet* 2015; 385(9975): 1295-304.
11. Comparison of glaucomatous progression between untreated patients with normal-tension glaucoma and patients with therapeutically reduced intraocular pressures. Collaborative Normal-Tension Glaucoma Study Group. *Am J Ophthalmol* 1998; 126(4): 487-97.
12. Heijl A, Leske MC, Bengtsson B, et al. Reduction of intraocular pressure and glaucoma progression: results from the Early Manifest Glaucoma Trial. *Arch Ophthalmol* 2002; 120(10): 1268-79.
13. Newman-Casey PA, Niziol LM, Gillespie BW, Janz NK, Lichter PR, Musch DC. The Association between Medication Adherence and Visual Field Progression in the Collaborative Initial Glaucoma Treatment Study. *Ophthalmology* 2020; 127(4): 477-83.
14. Gazzard G, Konstantakopoulou E, Garway-Heath D, et al. Selective laser trabeculoplasty versus eye drops for first-line treatment of ocular hypertension and glaucoma (LiGHT): a multicentre randomised controlled trial. *Lancet* 2019; 393(10180): 1505-16.
15. Poitras V, Wells C, Hutnik C, et al. Optimal Use of Minimally Invasive Glaucoma Surgery: A Health Technology Assessment. Ottawa (ON); 2019.
16. Che Hamzah J, Daka Q, Azuara-Blanco A. Home monitoring for glaucoma. *Eye (Lond)* 2020; 34(1): 155-60.
17. Li Z, He Y, Keel S, Meng W, Chang RT, He M. Efficacy of a Deep Learning System for Detecting Glaucomatous Optic Neuropathy Based on Color Fundus Photographs. *Ophthalmology* 2018; 125(8): 1199-206.

References

1. Brilliant LB, Pokhrel RP, Grasset NC, et al. Epidemiology of blindness in Nepal. *Bull World Health Organ* 1985; **63**(2): 375-86.
2. Faal H, Minassian D, Sowa S, Foster A. National survey of blindness and low vision in The Gambia: results. *Br J Ophthalmol* 1989; **73**(2): 82-7.
3. World Health Organization. The Prevention of Blindness. Report of a WHO Study Group. Technical Report Series No. 518. Geneva: World Health Organization, 1973.
4. World Health Organization. Consultation on development of standards for characterization of vision loss and visual functioning (WHO/PBL/03.91). Geneva: World Health Organization, 2003.
5. World Health Organization. Global initiative for the elimination of avoidable blindness (WHO/PBL/97.61 Rev 2). Geneva: World Health Organization, 2000.
6. World Health Organization. Global Initiative for the Elimination of Avoidable Blindness: action plan 2006-2011. Geneva: World Health Organization, 2007.
7. World Health Organization. Universal Eye Health: a global action plan 2014-2019. Geneva: World Health Organization, 2013.
8. World Health Organization. ICD-11 for Mortality and Morbidity Statistics (Version : 04 / 2019). 9D90 Vision impairment including blindness. Geneva: World Health Organization, 2019.
9. World Health Organization. World report on vision. Geneva: World Health Organization, 2019.
10. McKibbin M, Farragher TM, Shickle D. Monocular and binocular visual impairment in the UK Biobank study: prevalence, associations and diagnoses. *BMJ Open Ophthalmol* 2018; **3**(1): e000076.
11. Horton S, Gelband H, Jamison D, Levin C, Nugent R, Watkins D. Ranking 93 health interventions for low- and middle-income countries by cost-effectiveness. *PLoS One* 2017; **12**(8): e0182951.
12. Ohno-Matsui K, Jonas JB. Posterior staphyloma in pathologic myopia. *Prog Retin Eye Res* 2019; **70**: 99-109.
13. Holden BA, Fricke TR, Wilson DA, et al. Global Prevalence of Myopia and High Myopia and Temporal Trends from 2000 through 2050. *Ophthalmology* 2016; **123**(5): 1036-42.
14. Wodon Q, Male C, Nayihouba A, Smith E. Looking Ahead: Visual Impairment and School Eye Health Programs. The Price of Exclusion: Disability and Education Notes Series. Washington, DC: The World Bank, 2019.
15. He M, Xiang F, Zeng Y, et al. Effect of Time Spent Outdoors at School on the Development of Myopia Among Children in China: A Randomized Clinical Trial. *JAMA* 2015; **314**(11): 1142-8.
16. Khanal S, Phillips JR. Which low-dose atropine for myopia control? *Clin Exp Optom* 2019.
17. Fricke TR, Tahhan N, Resnikoff S, et al. Global Prevalence of Presbyopia and Vision Impairment from Uncorrected Presbyopia: Systematic Review, Meta-analysis, and Modelling. *Ophthalmology* 2018; **125**(10): 1492-9.
18. Reddy PA, Congdon N, MacKenzie G, et al. Effect of providing near glasses on productivity among rural Indian tea workers with presbyopia (PROSPER): a randomised trial. *Lancet Glob Health* 2018; **6**(9): e1019-e27.
19. Tham YC, Li X, Wong TY, Quigley HA, Aung T, Cheng CY. Global prevalence of glaucoma and projections of glaucoma burden through 2040: a systematic review and meta-analysis. *Ophthalmology* 2014; **121**(11): 2081-90.
20. John D, Parikh R. Cost-effectiveness and cost utility of community screening for glaucoma in urban India. *Public Health* 2017; **148**: 37-48.
21. Tang J, Liang Y, O'Neill C, Kee F, Jiang J, Congdon N. Cost-effectiveness and cost-utility of population-based glaucoma screening in China: a decision-analytic Markov model. *Lancet Glob Health* 2019; **7**(7): e968-e78.
22. Khanna S, Komati R, Eichenbaum DA, Hariprasad I, Ciulla TA, Hariprasad SM. Current and upcoming anti-VEGF therapies and dosing strategies for the treatment of neovascular AMD: a comparative review. *BMJ Open Ophthalmol* 2019; **4**(1): e000398.
23. Gahn GM, Khanani AM. New Therapies of Neovascular AMD beyond Anti-VEGF Injections. *Vision (Basel)* 2018; **2**(1).
24. Yerramothu P. New Therapies of Neovascular AMD-Beyond Anti-VEGFs. *Vision (Basel)* 2018; **2**(3).
25. Early photocoagulation for diabetic retinopathy. ETDRS report number 9. Early Treatment Diabetic Retinopathy Study Research Group. *Ophthalmology* 1991; **98**(5 Suppl): 766-85.
26. Scanlon PH. The English National Screening Programme for diabetic retinopathy 2003-2016. *Acta Diabetol* 2017; **54**(6): 515-25.
27. Taylor HR, Burton MJ, Haddad D, West S, Wright H. Trachoma. *Lancet* 2014; **384**(9960): 2142-52.
28. World Health Organization. WHO Alliance for the Global Elimination of Trachoma by 2020: progress report, 2019. *Wkly Epidemiol Rec* 2020; **30**(95): 349-60.
29. GBD 2017 Disease and Injury Incidence and Prevalence Collaborators. Global, regional, and national incidence, prevalence, and years lived with disability for 354 diseases and injuries for 195 countries and territories, 1990-2017: a systematic analysis for the Global Burden of Disease Study 2017. *Lancet* 2018; **392**(10159): 1789-858.
30. Brown L, Leck AK, Gichangi M, Burton MJ, Denning DW. The global incidence and diagnosis of fungal keratitis. *Lancet Infectious Diseases* 2020; **In Press**.

31. Upadhyay MP, Karmacharya PC, Koirala S, et al. The Bhaktapur eye study: ocular trauma and antibiotic prophylaxis for the prevention of corneal ulceration in Nepal. *Br J Ophthalmol* 2001; **85**(4): 388-92.
32. World Health Organisation. Global prevalence of vitamin A deficiency in populations at risk 1995–2005: WHO global database on vitamin A deficiency. Geneva: World Health Organisation, 2009.
33. Kong L, Fry M, Al-Samarraie M, Gilbert C, Steinkuller PG. An update on progress and the changing epidemiology of causes of childhood blindness worldwide. *J AAPOS* 2012; **16**(6): 501-7.
34. Blencowe H, Lawn JE, Vazquez T, Fielder A, Gilbert C. Preterm-associated visual impairment and estimates of retinopathy of prematurity at regional and global levels for 2010. *Pediatr Res* 2013; **74 Suppl 1**: 35-49.
35. Chan-Ling T, Gole GA, Quinn GE, Adamson SJ, Darlow BA. Pathophysiology, screening and treatment of ROP: A multi-disciplinary perspective. *Prog Retin Eye Res* 2018; **62**: 77-119.
36. Fu Z, Hong H, Su Z, Lou B, Pan CW, Liu H. Global prevalence of amblyopia and disease burden projections through 2040: a systematic review and meta-analysis. *Br J Ophthalmol* 2019.
37. Tsirouki T, Dastiridou A, Symeonidis C, et al. A Focus on the Epidemiology of Uveitis. *Ocul Immunol Inflamm* 2018; **26**(1): 2-16.
38. Zhang JH, Ramke J, Mwangi N, et al. Global eye health and the sustainable development goals: protocol for a scoping review. *BMJ open* 2020; **10**(3): e035789.
39. RNIB. Key Statistics on Pupils with Vision Impairment. 2017. https://www.rnib.org.uk/insight-online/statistics-pupils-vision-impairment (accessed 17/10 2019).
40. World Health Organization. Towards a Common Language for Functioning, Disability and Health. Geneva: World Health Organization, 2002.
41. Assi L, Rosman L, Chamseddine F, et al. Eye health and quality of life: an umbrella review protocol. *BMJ open* 2020; **10**(8): e037648.
42. Heine C, Browning C. Dual Sensory Loss in Older Adults: A Systematic Review. *Gerontologist* 2015; **55**(5): 913-28.
43. Kiely KM, Anstey KJ, Luszcz MA. Dual sensory loss and depressive symptoms: the importance of hearing, daily functioning, and activity engagement. *Front Hum Neurosci* 2013; **7**: 837.
44. Schneck ME, Lott LA, Haegerstrom-Portnoy G, Brabyn JA. Association between hearing and vision impairments in older adults. *Ophthalmic Physiol Opt* 2012; **32**(1): 45-52.
45. Kim Y, Kwak Y, Kim JS. The association between suicide ideation and sensory impairment among elderly Koreans. *Aging Ment Health* 2015; **19**(7): 658-65.
46. World Health Organization. Global status report on road safety 2018. . Geneva: World Health Organization, 2018.
47. Bohensky M, Charlton J, Odell M, Keeffe J. Implications of vision testing for older driver licensing. *Traffic Inj Prev* 2008; **9**(4): 304-13.
48. Honavar SG. Driving blind - Should tests of visual function be mandatory for driving license? *Indian J Ophthalmol* 2019; **67**(2): 193-4.
49. Isawumi MA, Adeoti CO, Ubah JN, Oluwatimilehin IO, Raji RA. Ocular status of commercial drivers in Osun State, Nigeria. *Afr J Med Med Sci* 2011; **40**(4): 405-11.
50. Grabowski DC, Campbell CM, Morrisey MA. Elderly licensure laws and motor vehicle fatalities. *JAMA* 2004; **291**(23): 2840-6.
51. Cross JM, McGwin G, Jr., Rubin GS, et al. Visual and medical risk factors for motor vehicle collision involvement among older drivers. *Br J Ophthalmol* 2009; **93**(3): 400-4.
52. Achigbu EO, Fiebai B. Visual defects and commercial motorcycle accidents in south eastern Nigeria. *Niger J Med* 2013; **22**(4): 299-303.
53. Owsley C, Stalvey B, Wells J, Sloane ME. Older drivers and cataract: driving habits and crash risk. *J Gerontol A Biol Sci Med Sci* 1999; **54**(4): M203-11.
54. Owsley C, Stalvey BT, Wells J, Sloane ME, McGwin G, Jr. Visual risk factors for crash involvement in older drivers with cataract. *Arch Ophthalmol* 2001; **119**(6): 881-7.
55. Subzwari S, Desapriya E, Scime G, Babul S, Jivani K, Pike I. Effectiveness of cataract surgery in reducing driving-related difficulties: a systematic review and meta-analysis. *Inj Prev* 2008; **14**(5): 324-8.
56. Owsley C, McGwin G, Jr., Sloane M, Wells J, Stalvey BT, Gauthreaux S. Impact of cataract surgery on motor vehicle crash involvement by older adults. *JAMA* 2002; **288**(7): 841-9.
57. Meuleners LB, Brameld K, Fraser ML, Chow K. The impact of first- and second-eye cataract surgery on motor vehicle crashes and associated costs. *Age Ageing* 2019; **48**(1): 128-33.
58. Schlenker MB, Thiruchelvam D, Redelmeier DA. Association of Cataract Surgery With Traffic Crashes. *JAMA Ophthalmol* 2018; **136**(9): 998-1007.
59. Kwon M, Huisingh C, Rhodes LA, McGwin G, Jr., Wood JM, Owsley C. Association between Glaucoma and At-fault Motor Vehicle Collision Involvement among Older Drivers: A Population-based Study. *Ophthalmology* 2016; **123**(1): 109-16.

60. Tanabe S, Yuki K, Ozeki N, et al. The association between primary open-angle glaucoma and motor vehicle collisions. *Invest Ophthalmol Vis Sci* 2011; **52**(7): 4177-81.
61. McGwin G, Jr., Huisingh C, Jain SG, Girkin CA, Owsley C. Binocular visual field impairment in glaucoma and at-fault motor vehicle collisions. *J Glaucoma* 2015; **24**(2): 138-43.
62. Ono T, Yuki K, Asaoka R, et al. Glaucomatous visual field defect severity and the prevalence of motor vehicle collisions in Japanese: a hospital/clinic-based cross-sectional study. *J Ophthalmol* 2015; **2015**: 497067.
63. Yuki K, Asaoka R, Tsubota K. The relationship between central visual field damage and motor vehicle collisions in primary open-angle glaucoma patients. *PLoS One* 2014; **9**(12): e115572.
64. Deshmukh AV, Murthy GJ, Reddy A, Murthy PR, Kattige J, Murthy VR. Older Drivers and Glaucoma in India: Driving Habits and Crash Risks. *J Glaucoma* 2019; **28**(10): 896-900.
65. McGwin G, Jr., Mitchell B, Searcey K, et al. Examining the association between age-related macular degeneration and motor vehicle collision involvement: a retrospective cohort study. *Br J Ophthalmol* 2013; **97**(9): 1173-6.
66. Sengupta S, van Landingham SW, Solomon SD, Do DV, Friedman DS, Ramulu PY. Driving habits in older patients with central vision loss. *Ophthalmology* 2014; **121**(3): 727-32.
67. Bressler NM, Chang TS, Varma R, et al. Driving ability reported by neovascular age-related macular degeneration patients after treatment with ranibizumab. *Ophthalmology* 2013; **120**(1): 160-8.
68. Bressler NM, Varma R, Mitchell P, et al. Effect of Ranibizumab on the Decision to Drive and Vision Function Relevant to Driving in Patients With Diabetic Macular Edema: Report From RESTORE, RIDE, and RISE Trials. *JAMA Ophthalmol* 2016; **134**(2): 160-6.
69. Baker JM, Drews-Botsch C, Pfeiffer MR, Curry AE. Driver licensing and motor vehicle crash rates among young adults with amblyopia and unilateral vision impairment. *J AAPOS* 2019; **23**(4): 230-2.
70. Keay L, Palagyi A, Do V, et al. Vision and driving status of older Australians with cataract: an investigation of public hospital waiting lists. *Clin Exp Optom* 2016; **99**(5): 449-55.
71. World Health Organization. WHO global report on falls prevention in older age. Geneva: World Health Organization, 2007.
72. James SL, Lucchesi LR, Bisignano C, et al. The global burden of falls: global, regional and national estimates of morbidity and mortality from the Global Burden of Disease Study 2017. *Inj Prev* 2020.
73. Deandrea S, Lucenteforte E, Bravi F, Foschi R, La Vecchia C, Negri E. Risk factors for falls in community-dwelling older people: a systematic review and meta-analysis. *Epidemiology* 2010; **21**(5): 658-68.
74. Hong T, Mitchell P, Burlutsky G, Samarawickrama C, Wang JJ. Visual impairment and the incidence of falls and fractures among older people: longitudinal findings from the Blue Mountains Eye Study. *Invest Ophthalmol Vis Sci* 2014; **55**(11): 7589-93.
75. Freeman EE, Munoz B, Rubin G, West SK. Visual field loss increases the risk of falls in older adults: the Salisbury eye evaluation. *Invest Ophthalmol Vis Sci* 2007; **48**(10): 4445-50.
76. Patino CM, McKean-Cowdin R, Azen SP, et al. Central and peripheral visual impairment and the risk of falls and falls with injury. *Ophthalmology* 2010; **117**(2): 199-206 e1.
77. Lamoureux EL, Chong E, Wang JJ, et al. Visual impairment, causes of vision loss, and falls: the singapore malay eye study. *Invest Ophthalmol Vis Sci* 2008; **49**(2): 528-33.
78. Szabo SM, Janssen PA, Khan K, Lord SR, Potter MJ. Neovascular AMD: an overlooked risk factor for injurious falls. *Osteoporos Int* 2010; **21**(5): 855-62.
79. Ivers RQ, Cumming RG, Mitchell P, Attebo K. Visual impairment and falls in older adults: the Blue Mountains Eye Study. *Journal of the American Geriatrics Society* 1998; **46**(1): 58-64.
80. Harwood RH, Foss AJ, Osborn F, Gregson RM, Zaman A, Masud T. Falls and health status in elderly women following first eye cataract surgery: a randomised controlled trial. *Br J Ophthalmol* 2005; **89**(1): 53-9.
81. Meuleners LB, Fraser ML, Ng J, Morlet N. The impact of first- and second-eye cataract surgery on injurious falls that require hospitalisation: a whole-population study. *Age Ageing* 2014; **43**(3): 341-6.
82. Tseng VL, Yu F, Lum F, Coleman AL. Risk of fractures following cataract surgery in Medicare beneficiaries. *JAMA* 2012; **308**(5): 493-501.
83. Palagyi A, Morlet N, McCluskey P, et al. Visual and refractive associations with falls after first-eye cataract surgery. *J Cataract Refract Surg* 2017; **43**(10): 1313-21.
84. Cumming RG, Ivers R, Clemson L, et al. Improving vision to prevent falls in frail older people: a randomized trial. *Journal of the American Geriatrics Society* 2007; **55**(2): 175-81.
85. Elliott DB. The Glenn A. Fry award lecture 2013: blurred vision, spectacle correction, and falls in older adults. *Optom Vis Sci* 2014; **91**(6): 593-601.
86. Russell MA, Hill KD, Day LM, Blackberry I, Gurrin LC, Dharmage SC. Development of the Falls Risk for Older People in the Community (FROP-Com) screening tool. *Age Ageing* 2009; **38**(1): 40-6.
87. Ehrlich JR, Ramke J, Macleod D, et al. Association between vision impairment and mortality: protocol for a systematic review and meta-analysis. *BMJ open* 2020; **10**(6): e037556.

88. Bourne R. Magnitude, temporal trends, and projections of the global prevalence of blindness and distance and near vision impairment: a systematic review and meta-analysis - VLEG/GBD 2020. *SUBMITTED* 2020.
89. World Health Organization. Preventing blindness in children. WHO/PBL/00.77. Geneva: World Health Organization, 2000.
90. Sommer A, Davidson FR, Annecy A. Assessment and control of vitamin A deficiency: the Annecy Accords. *J Nutr* 2002; **132**(9 Suppl): 2845S-50S.
91. United Nations. Under 5 mortality data: Report 2014 UN Interagency Group for Child Mortality Estimation: United Nations, 2014.
92. Limburg H, Gilbert C, Hon DN, Dung NC, Hoang TH. Prevalence and causes of blindness in children in Vietnam. *Ophthalmology* 2012; **119**(2): 355-61.
93. Cama AT, Sikivou BT, Keeffe JE. Childhood visual impairment in Fiji. *Arch Ophthalmol* 2010; **128**(5): 608-12.
94. Murthy GV, Mactaggart I, Mohammad M, et al. Assessing the prevalence of sensory and motor impairments in childhood in Bangladesh using key informants. *Arch Dis Child* 2014; **99**(12): 1103-8.
95. Rahi JS, Cable N, British Childhood Visual Impairment Study G. Severe visual impairment and blindness in children in the UK. *Lancet* 2003; **362**(9393): 1359-65.
96. Gilbert C, Fielder A, Gordillo L, et al. Characteristics of infants with severe retinopathy of prematurity in countries with low, moderate, and high levels of development: implications for screening programs. *Pediatrics* 2005; **115**(5): e518-25.
97. Saeedi P, Petersohn I, Salpea P, et al. Global and regional diabetes prevalence estimates for 2019 and projections for 2030 and 2045: Results from the International Diabetes Federation Diabetes Atlas, 9(th) edition. *Diabetes Res Clin Pract* 2019; **157**: 107843.
98. Yau JW, Rogers SL, Kawasaki R, et al. Global prevalence and major risk factors of diabetic retinopathy. *Diabetes Care* 2012; **35**(3): 556-64.
99. Lee R, Wong TY, Sabanayagam C. Epidemiology of diabetic retinopathy, diabetic macular edema and related vision loss. *Eye Vis (Lond)* 2015; **2**: 17.
100. Levy PS, Lemeshow S. Sampling of populations: methods and applications: John Wiley & Sons; 2013.
101. World Health Organisation. Universal eye health: a global action plan 2014-2019. 2013. https://www.who.int/blindness/AP2014_19_English.pdf (accessed 21.08 2019).
102. Bourne RRA, Flaxman SR, Braithwaite T, et al. Magnitude, temporal trends, and projections of the global prevalence of blindness and distance and near vision impairment: a systematic review and meta-analysis. *Lancet Glob Health* 2017; **5**(9): e888-e97.
103. Ramke J, Zwi AB, Silva JC, et al. Evidence for national universal eye health plans. *Bull World Health Organ* 2018; **96**(10): 695.
104. Flaxman SR, Bourne RRA, Resnikoff S, et al. Global causes of blindness and distance vision impairment 1990-2020: a systematic review and meta-analysis. *Lancet Glob Health* 2017; **5**(12): e1221-e34.
105. Demographic and Health Survey Sampling and Household Listing Manual. Calverton, USA: ICF International, 2012.
106. World Health Organization. The WHO STEPwise approach to noncommunicable disease risk factor surveillance. Geneva: World Health Organization, 2017.
107. Foreman J, Keel S, Dunn R, van Wijngaarden P, Taylor HR, Dirani M. Sampling methodology and site selection in the National Eye Health Survey: an Australian population-based prevalence study. *Clinical & experimental ophthalmology* 2017; **45**(4): 336-47.
108. Dineen B, Gilbert CE, Rabiu M, et al. The Nigerian national blindness and visual impairment survey: Rationale, objectives and detailed methodology. *BMC ophthalmology* 2008; **8**(1): 17.
109. Braithwaite T, Verlander NQ, Bartholomew D, et al. The National Eye survey of Trinidad and Tobago (NESTT): rationale, objectives and methodology. *Ophthalmic Epidemiol* 2017; **24**(2): 116-29.
110. Foster PJ, Buhrmann R, Quigley HA, Johnson GJ. The definition and classification of glaucoma in prevalence surveys. *Br J Ophthalmol* 2002; **86**(2): 238-42.
111. Bastawrous A, Burgess PI, Mahdi AM, Kyari F, Burton MJ, Kuper H. Posterior segment eye disease in sub-S aharan A frica: review of recent population-based studies. *Trop Med Int Health* 2014; **19**(5): 600-9.
112. Ramke J, Kuper H, Limburg H, et al. Avoidable waste in ophthalmic epidemiology: a review of blindness prevalence surveys in low and middle income countries 2000–2014. *Ophthalmic Epidemiol* 2018; **25**(1): 13-20.
113. Marmamula S, Keeffe JE, Rao GN. Rapid assessment methods in eye care: An overview. *Indian journal of ophthalmology* 2012; **60**(5): 416.
114. Limburg H, Kumar R, Indrayan A, Sundaram K. Rapid assessment of prevalence of cataract blindness at district level. *International journal of epidemiology* 1997; **26**(5): 1049-54.
115. Limburg H. Rapid assessment of cataract surgical services. 2001.
116. Kuper H, Polack S, Limburg H. Rapid assessment of avoidable blindness. *Community Eye Health* 2006; **19**(60): 68.

117. Mactaggart I, Limburg H, Bastawrous A, Burton MJ, Kuper H. Rapid Assessment of Avoidable Blindness: looking back, looking forward. *Br J Ophthalmol* 2019; **103**(11): 1549-52.
118. Marmamula S, Keeffe JE, Rao GN. Uncorrected refractive errors, presbyopia and spectacle coverage: results from a rapid assessment of refractive error survey. *Ophthalmic Epidemiol* 2009; **16**(5): 269-74.
119. Marmamula S, Khanna RC, Shekhar K, Rao GN. A population-based cross-sectional study of barriers to uptake of eye care services in South India: the Rapid Assessment of Visual Impairment (RAVI) project. *BMJ open* 2014; **4**(6): e005125.
120. Zhang XJ, Leung CKS, Li EY, et al. Diagnostic Accuracy of Rapid Assessment of Avoidable Blindness: A Population-based Assessment. *Am J Ophthalmol* 2020; **213**: 235-43.
121. Mactaggart I, Wallace S, Ramke J, et al. Rapid assessment of avoidable blindness for health service planning. *Bull World Health Organ* 2018; **96**(10): 726.
122. Garin N, Olaya B, Lara E, et al. Visual impairment and multimorbidity in a representative sample of the Spanish population. *BMC Public Health* 2014; **14**: 815.
123. Braithwaite T, Verlander NQ, Peto T, et al. National Eye Survey of Trinidad and Tobago (NESTT): prevalence, causes and risk factors for presenting vision impairment in adults over 40 years. *Br J Ophthalmol* 2020; **104**(1): 74-80.
124. Foreman J, Keel S, Dunn R, van Wijngaarden P, Taylor HR, Dirani M. Sampling methodology and site selection in the National Eye Health Survey: an Australian population-based prevalence study. *Clin Exp Ophthalmol* 2017; **45**(4): 336-47.
125. Murray CJ. Quantifying the burden of disease: the technical basis for disability-adjusted life years. *Bull World Health Organ* 1994; **72**(3): 429-45.
126. Murray CJ. Rethinking DALYs. In: Murray CJ, Lopez, A.D., ed. The global burden of disease: a comprehensive assessment of mortality and disability from diseases, injuries and risk factors in 1990 and projected to 2020. Cambridge, MA: Harvard University Press; 1996: 1-98.
127. Stouthard ME, Essink-Bot, M.L., Bonsel, G.J. Disability weights for diseases: a modified protocol and results for a Western European region. *European Journal of Public Health* 2000; **10**: 24-30.
128. Baltussen RM, Sanon M, Sommerfeld J, Wurthwein R. Obtaining disability weights in rural Burkina Faso using a culturally adapted visual analogue scale. *Health economics* 2002; **11**(2): 155-63.
129. Lai T, Habicht J, Kiivet RA. Measuring burden of disease in Estonia to support public health policy. *Eur J Public Health* 2009; **19**(5): 541-7.
130. Salomon JA, Vos T, Hogan DR, et al. Common values in assessing health outcomes from disease and injury: disability weights measurement study for the Global Burden of Disease Study 2010. *Lancet* 2013; **380**(9859): 2129-43.
131. World Health Organization. WHO methods and data sources for global burden of disease estimates 2000-2011. Geneva: World Health Organization, 2013.
132. Haagsma JA, Maertens de Noordhout C, Polinder S, et al. Assessing disability weights based on the responses of 30,660 people from four European countries. *Population health metrics* 2015; **13**: 10.
133. Salomon JA, Haagsma JA, Davis A, et al. Disability weights for the Global Burden of Disease 2013 study. *Lancet Glob Health* 2015; **3**(11): e712-23.
134. Marques AP, Ramke J, Cairns J, et al. Estimating the global cost of vision impairment and its major causes: protocol for a systematic review. *BMJ open* 2020; **10**(9): e036689.
135. Drummond MF, Jefferson TO. Guidelines for authors and peer reviewers of economic submissions to the BMJ. The BMJ Economic Evaluation Working Party. *Bmj* 1996; **313**(7052): 275-83.
136. Molinier L, Bauvin E, Combescure C, et al. Methodological considerations in cost of prostate cancer studies: a systematic review. *Value Health* 2008; **11**(5): 878-85.
137. Chakravarthy U, Biundo E, Saka RO, Fasser C, Bourne R, Little J-A. The Economic Impact of Blindness in Europe. *Ophthalmic Epidemiol* 2017; **24**(4): 239-47.
138. Roberts CB, Hiratsuka Y, Yamada M, et al. Economic cost of visual impairment in Japan. *Arch Ophthalmol* 2010; **128**(6): 766-71.
139. Eckert KA, Carter MJ, Lansingh VC, et al. A Simple Method for Estimating the Economic Cost of Productivity Loss Due to Blindness and Moderate to Severe Visual Impairment. *Ophthalmic Epidemiol* 2015; **22**(5): 349-55.
140. Guan X, Fu M, Lin F, Zhu D, Vuillermin D, Shi L. Burden of visual impairment associated with eye diseases: exploratory survey of 298 Chinese patients. *BMJ open* 2019; **9**(9): e030561.
141. Wang M-T, Ng K, Sheu S-J, Yeh W-S, Lo Y-W, Lee W-J. Analysis of Excess Direct Medical Costs of Vision Impairment in Taiwan. *Value in health regional issues* 2013; **2**(1): 57-63.
142. Park H-Y, Ryu H, Kang H-Y, Lee H, Kwon J-W. Clinical and Economic Burden of Visual Impairment in an Aging Society of South Korea. *Asia-Pacific journal of public health* 2015; **27**(6): 631-42.
143. Wang X, Lamoureux E, Zheng Y, Ang M, Wong TY, Luo N. Health burden associated with visual impairment in Singapore: the Singapore epidemiology of eye disease study. *Ophthalmology* 2014; **121**(9): 1837-42.
144. Taylor HR, Pezzullo ML, Keeffe JE. The economic impact and cost of visual impairment in Australia. *Br J Ophthalmol* 2006; **90**(3): 272-5.

145. Taylor HR, Pezzullo ML, Nesbitt SJ, Keeffe JE. Costs of interventions for visual impairment. *Am J Ophthalmol* 2007; **143**(4): 561-5.
146. Chou S-L, Lamoureux E, Keeffe J. Methods for measuring personal costs associated with vision impairment. *Ophthalmic Epidemiol* 2006; **13**(6): 355-63.
147. O'Connor PM, Chou S-L, Lamoureux EL, Keeffe JE. Costs of vision impairment in childhood and youth: diary case studies. *Optometry and vision science : official publication of the American Academy of Optometry* 2008; **85**(11): 1106-9.
148. Wong EYH, Chou S-L, Lamoureux EL, Keeffe JE. Personal costs of visual impairment by different eye diseases and severity of visual loss. *Ophthalmic Epidemiol* 2008; **15**(5): 339-44.
149. Keeffe JE, Chou S-L, Lamoureux EL. The cost of care for people with impaired vision in Australia. *Archives of ophthalmology (Chicago, Ill : 1960)* 2009; **127**(10): 1377-81.
150. Wright SE, Keeffe JE, Thies LS. Direct costs of blindness in Australia. *Clinical & experimental ophthalmology* 2000; **28**(3): 140-2.
151. Hsueh Y-sA, Brando A, Dunt D, Anjou MD, Boudville A, Taylor H. Cost of close the gap for vision of Indigenous Australians: On estimating the extra resources required. *The Australian journal of rural health* 2013; **21**(6): 329-35.
152. Economics A. Clear focus: The economic impact of of vision loss in Australia in 2009: Melbourne, Australia; 2010.
153. Alva ML, Gray A, Mihaylova B, Leal J, Holman RR. The impact of diabetes-related complications on healthcare costs: new results from the UKPDS (UKPDS 84). *Diabetic Medicine* 2015; **32**(4): 459-66.
154. Clarke P, Gray A, Legood R, Briggs A, Holman R. The impact of diabetes-related complications on healthcare costs: results from the United Kingdom Prospective Diabetes Study (UKPDS Study No. 65). *Diabetic Medicine* 2003; **20**(6): 442-50.
155. Lafuma A, Brezin A, Lopatriello S, et al. Evaluation of non-medical costs associated with visual impairment in four European countries: France, Italy, Germany and the UK. *PharmacoEconomics* 2006; **24**(2): 193-205.
156. Chuvarayan Y, Finger RP, Koberlein-Neu J. Economic burden of blindness and visual impairment in Germany from a societal perspective: a cost-of-illness study. *The European journal of health economics : HEPAC : health economics in prevention and care* 2019; (101134867).
157. Pezzullo L, Streatfeild J, Simkiss P, Shickle D. The economic impact of sight loss and blindness in the UK adult population. *BMC health services research* 2018; **18**(1): 63.
158. Meads C, Hyde C. What is the cost of blindness? *Br J Ophthalmol* 2003; **87**(10): 1201-4.
159. Schakel W, van der Aa HPA, Bode C, Hulshof CTJ, van Rens GHMB, van Nispen RMA. The Economic Burden of Visual Impairment and Comorbid Fatigue: A Cost-of-Illness Study (From a Societal Perspective). *Invest Ophthalmol Vis Sci* 2018; **59**(5): 1916-23.
160. Lafuma A, Brezin A, Fagnani F, Mimaud V, Mesbah M, Berdeaux G. Nonmedical economic consequences attributable to visual impairment: a nation-wide approach in France. *The European journal of health economics : HEPAC : health economics in prevention and care* 2006; **7**(3): 158-64.
161. Marques AP, Macedo AF, Hernandez-Moreno L, et al. The use of informal care by people with vision impairment. *PLoS One* 2018; **13**(6): e0198631.
162. Marques AP, Macedo AF, Lima Ramos P, et al. Productivity Losses and Their Explanatory Factors Amongst People with Impaired Vision. *Ophthalmic Epidemiol* 2019; (cg6, 9435674): 1-15.
163. Frick KD, Gower EW, Kempen JH, Wolff JL. Economic impact of visual impairment and blindness in the United States. *Arch Ophthalmol* 2007; **125**(4): 544-50.
164. Frick KD, Walt JG, Chiang TH, et al. Direct costs of blindness experienced by patients enrolled in managed care. *Ophthalmology* 2008; **115**(1): 11-7.
165. Cruess AF, Gordon KD, Bellan L, Mitchell S, Pezzullo ML. The cost of vision loss in Canada. 2. Results. *Canadian journal of ophthalmology Journal canadien d'ophtalmologie* 2011; **46**(4): 315-8.
166. Javitt JC, Zhou Z, Willke RJ. Association between vision loss and higher medical care costs in Medicare beneficiaries costs are greater for those with progressive vision loss. *Ophthalmology* 2007; **114**(2): 238-45.
167. Wittenborn JS, Zhang X, Feagan CW, et al. The economic burden of vision loss and eye disorders among the United States population younger than 40 years. *Ophthalmology* 2013; **120**(9): 1728-35.
168. Gordois A, Cutler H, Pezzullo L, et al. An estimation of the worldwide economic and health burden of visual impairment. *Global public health* 2012; **7**(5): 465-81.
169. Armstrong KL, Jovic M, Vo-Phuoc JL, Thorpe JG, Doolan BL. The global cost of eliminating avoidable blindness. *Indian journal of ophthalmology* 2012; **60**(5): 475-80.
170. Frick KD, Foster A. The magnitude and cost of global blindness: an increasing problem that can be alleviated. *Am J Ophthalmol* 2003; **135**(4): 471-6.
171. Harrabi H, Aubin MJ, Zunzunegui MV, Haddad S, Freeman EE. Visual difficulty and employment status in the world. *PLoS One* 2014; **9**(2): e88306.

172. Bastawrous A, Suni AV. Thirty Year Projected Magnitude (to 2050) of Near and Distance Vision Impairment and the Economic Impact if Existing Solutions are Implemented Globally. *Ophthalmic Epidemiol* 2019: 1-6.
173. Zheng Y-F, Pan C-W, Chay J, Wong TY, Finkelstein E, Saw S-M. The economic cost of myopia in adults aged over 40 years in Singapore. *Invest Ophthalmol Vis Sci* 2013; **54**(12): 7532-7.
174. Lafuma A, Laurendeau C, Lamerain E, Berdeaux G. Economics and attitudes regarding spectacles in daily life: a European perspective. *Ophthalmic epidemiology* 2009; **16**(4): 218-23.
175. Ruiz-Moreno JM, Roura M, en representacion del grupo del estudio M. Cost of myopic patients with and without myopic choroidal neovascularisation. *Archivos de la Sociedad Espanola de Oftalmologia* 2016; **91**(6): 265-72.
176. Vitale S, Cotch MF, Sperduto R, Ellwein L. Costs of refractive correction of distance vision impairment in the United States, 1999-2002. *Ophthalmology* 2006; **113**(12): 2163-70.
177. Mohammadi S-F, Alinia C, Tavakkoli M, Lashay A, Chams H. Refractive surgery: the most cost-saving technique in refractive errors correction. *International journal of ophthalmology* 2018; **11**(6): 1013-9.
178. Angell B, Ali F, Gandhi M, et al. Ready-made and custom-made eyeglasses in India: a cost-effectiveness analysis of a randomised controlled trial. *BMJ open ophthalmology* 2018; **3**(1): e000123.
179. Griffiths UK, Bozzani F, Muleya L, Mumba M. Costs of eye care services: prospective study from a faith-based hospital in Zambia. *Ophthalmic Epidemiol* 2015; **22**(1): 43-51.
180. Smith TST, Frick KD, Holden BA, Fricke TR, Naidoo KS. Potential lost productivity resulting from the global burden of uncorrected refractive error. *Bull World Health Organ* 2009; **87**(6): 431-7.
181. Naidoo KS, Fricke TR, Frick KD, et al. Potential Lost Productivity Resulting from the Global Burden of Myopia: Systematic Review, Meta-analysis, and Modeling. *Ophthalmology* 2019; **126**(3): 338-46.
182. Fricke TR, Holden BA, Wilson DA, et al. Global cost of correcting vision impairment from uncorrected refractive error. *Bull World Health Organ* 2012; **90**(10): 728-38.
183. Frick KD, Joy SM, Wilson DA, Naidoo KS, Holden BA. The Global Burden of Potential Productivity Loss from Uncorrected Presbyopia. *Ophthalmology* 2015; **122**(8): 1706-10.
184. Eye Care Comparative Effectiveness Research T. Cost-effectiveness of cataract surgery in Japan. *Japanese journal of ophthalmology* 2011; **55**(4): 333-42.
185. Malot J, Combe C, Moss A, Savary P, Hida H, Ligeon-Ligeonnet P. [Cost of cataract surgery in a public hospital]. *Journal francais d'ophtalmologie* 2011; **34**(1): 10-6.
186. Nghiem-Buffet MH, de Pouvourville G, Renard G, Ullern M, Boureau C, Chaine G. [Cost of managing cataracts. Evaluation of traditional hospitalization and ambulatory surgery]. *Presse medicale (Paris, France : 1983)* 2001; **30**(39-40 Pt 1): 1924-6.
187. Fattore G, Torbica A. Cost and reimbursement of cataract surgery in Europe: a cross-country comparison. *Health economics* 2008; **17**(1 Suppl): S71-82.
188. Haynes R, Gale S, Mugford M, Davies P. Cataract surgery in a community hospital outreach clinic: patients' costs and satisfaction. *Soc Sci Med* 2001; **53**(12): 1631-40.
189. Minassian DC, Rosen P, Dart JK, et al. Extracapsular cataract extraction compared with small incision surgery by phacoemulsification: a randomised trial. *The British journal of ophthalmology* 2001; **85**(7): 822-9.
190. Sach TH, Foss AJ, Gregson RM, et al. Falls and health status in elderly women following first eye cataract surgery: an economic evaluation conducted alongside a randomised controlled trial. *Br J Ophthalmol* 2007; **91**(12): 1675-9.
191. Lundstrom M, Brege KG, Floren I, Roos P, Stenevi U, Thorburn W. Cataract surgery and effectiveness. 1. Variation in costs between different providers of cataract surgery. *Acta ophthalmologica Scandinavica* 2000; **78**(3): 335-9.
192. Stenevi U, Lundstrom M, Thorburn W. The cost of cataract patients awaiting surgery. *Acta ophthalmologica Scandinavica* 2000; **78**(6): 703-5.
193. Brown GC, Brown MM, Menezes A, Busbee BG, Lieske HB, Lieske PA. Cataract surgery cost utility revisited in 2012: a new economic paradigm. *Ophthalmology* 2013; **120**(12): 2367-76.
194. Dave H, Phoenix V, Becker ER, Lambert SR. Simultaneous vs sequential bilateral cataract surgery for infants with congenital cataracts: Visual outcomes, adverse events, and economic costs. *Archives of ophthalmology (Chicago, Ill : 1960)* 2010; **128**(8): 1050-4.
195. Fenter TC, Naslund MJ, Shah MB, Eaddy MT, Black L. The cost of treating the 10 most prevalent diseases in men 50 years of age or older. *The American journal of managed care* 2006; **12**(4 Suppl): S90-8.
196. Kruger SJ, DuBois L, Becker ER, et al. Cost of intraocular lens versus contact lens treatment after unilateral congenital cataract surgery in the infant aphakia treatment study at age 5 years. *Ophthalmology* 2015; **122**(2): 288-92.
197. Schmier JK, Halpern MT, Covert DW, Matthews GP. Evaluation of costs for cystoid macular edema among patients after cataract surgery. *Retina (Philadelphia, Pa)* 2007; **27**(5): 621-8.
198. O'Brien JJ, Gonder J, Botz C, Chow KY, Arshinoff SA. Immediately sequential bilateral cataract surgery versus delayed sequential bilateral cataract surgery: potential hospital cost savings. *Canadian journal of ophthalmology Journal canadien d'ophtalmologie* 2010; **45**(6): 596-601.

199. Arieta CEL, Nascimento MA, Lira RPC, Kara-Jose N. [Waste of medical tests in preoperative evaluation for cataract surgery]. *Cadernos de saude publica* 2004; **20**(1): 303-10.
200. Kara N, Jr., Sirtoli MGGM, Santhiago MR, Parede TRR, Espindola RFd, Carvalho RdS. Phacoemulsification versus extracapsular extraction: governmental costs. *Clinics (Sao Paulo, Brazil)* 2010; **65**(4): 357-61.
201. Saad Filho R, Saad FGL, Freitas LLd. [Cost of phacoemulsification in the national campaign of elective cataract surgery in Itapolis, SP, Brazil]. *Arquivos brasileiros de oftalmologia* 2005; **68**(1): 55-9.
202. Essue BM, Li Q, Hackett ML, et al. A multicenter prospective cohort study of quality of life and economic outcomes after cataract surgery in Vietnam: the VISIONARY study. *Ophthalmology* 2014; **121**(11): 2138-46.
203. Loo C-Y, Kandiah M, Arumugam G, et al. Cost efficiency and cost effectiveness of cataract surgery at the Malaysian Ministry of Health ophthalmic services. *International ophthalmology* 2004; **25**(2): 81-7.
204. Fang J, Wang X, Lin Z, Yan J, Yang Y, Li J. Variation of cataract surgery costs in four different graded providers of China. *BMC Public Health* 2010; **10**(1): 543.
205. Gogate P, Dole K, Ranade S, Deshpande M. Cost of pediatric cataract surgery in Maharashtra, India. *International journal of ophthalmology* 2010; **3**(2): 182-6.
206. Muralikrishnan R, Venkatesh R, Prajna NV, Frick KD. Economic cost of cataract surgery procedures in an established eye care centre in Southern India. *Ophthalmic epidemiology* 2004; **11**(5): 369-80.
207. Singh AJ, Garner P, Floyd K. Cost-effectiveness of public-funded options for cataract surgery in Mysore, India. *Lancet (London, England)* 2000; **355**(9199): 180-4.
208. Ibrahim N, Pozo-Martin F, Gilbert C. Direct non-medical costs double the total direct costs to patients undergoing cataract surgery in Zamfara state, Northern Nigeria: a case series. *BMC Health Services Research* 2015; **15**(1): 163.
209. Gradin D, Mundia D. Simultaneous bilateral cataract surgery with IOL implantation in children in Kenya. *Journal of pediatric ophthalmology and strabismus* 2012; **49**(3): 139-44.
210. Polack S, Kuper H, Eusebio C, Mathenge W, Wadud Z, Foster A. The impact of cataract on time-use: results from a population based case-control study in Kenya, the Philippines and Bangladesh. *Ophthalmic Epidemiol* 2008; **15**(6): 372-82.
211. Baltussen R, Sylla M, Mariotti SP. Cost-effectiveness analysis of cataract surgery: a global and regional analysis. *Bull World Health Organ* 2004; **82**(5): 338-45.
212. Economics A. Tunnel Vision:The Economic Impact of Primary Open Angle Glaucoma: Melbourne, Australia; 2011.
213. Hagman J. Comparison of resource utilization in the treatment of open-angle glaucoma between two cities in Finland: is more better? *Acta ophthalmologica* 2013; **91 Thesis 3**(101468102): 1-47.
214. Koleva D, Motterlini N, Schiavone M, Garattini L, Study Group G. Medical costs of glaucoma and ocular hypertension in Italian referral centres: a prospective study. *Ophthalmologica Journal international d'ophtalmologie International journal of ophthalmology Zeitschrift fur Augenheilkunde* 2007; **221**(5): 340-7.
215. Thygesen J, Aagren M, Arnavielle S, et al. Late-stage, primary open-angle glaucoma in Europe: social and health care maintenance costs and quality of life of patients from 4 countries. *Current medical research and opinion* 2008; **24**(6): 1763-70.
216. Traverso CE, Walt JG, Kelly SP, et al. Direct costs of glaucoma and severity of the disease: a multinational long term study of resource utilisation in Europe. *The British journal of ophthalmology* 2005; **89**(10): 1245-9.
217. Bramley T, Peeples P, Walt JG, Juhasz M, Hansen JE. Impact of vision loss on costs and outcomes in medicare beneficiaries with glaucoma. *Archives of ophthalmology (Chicago, Ill : 1960)* 2008; **126**(6): 849-56.
218. Gieser DK, Tracy Williams R, O'Connell W, et al. Costs and utilization of end-stage glaucoma patients receiving visual rehabilitation care: a US multisite retrospective study. *Journal of glaucoma* 2006; **15**(5): 419-25.
219. Iskedjian M, Walker J, Vicente C, et al. Cost of glaucoma in Canada: analyses based on visual field and physician's assessment. *Journal of glaucoma* 2003; **12**(6): 456-62.
220. Lee PP, Walt JG, Doyle JJ, et al. A multicenter, retrospective pilot study of resource use and costs associated with severity of disease in glaucoma. *Archives of ophthalmology (Chicago, Ill : 1960)* 2006; **124**(1): 12-9.
221. Prager AJ, Liebmann JM, Cioffi GA, Blumberg DM. Self-reported Function, Health Resource Use, and Total Health Care Costs Among Medicare Beneficiaries With Glaucoma. *JAMA ophthalmology* 2016; **134**(4): 357-65.
222. Rein DB, Wittenborn JS, Lee PP, et al. The cost-effectiveness of routine office-based identification and subsequent medical treatment of primary open-angle glaucoma in the United States. *Ophthalmology* 2009; **116**(5): 823-32.
223. Lazcano-Gomez G, Ramos-Cadena ML, Torres-Tamayo M, Hernandez de Oteyza A, Turati-Acosta M, Jimenez-Roman J. Cost of glaucoma treatment in a developing country over a 5-year period. *Medicine (Baltimore)* 2016; **95**(47): e5341.
224. Guedes RAP, Guedes VMP, Chaoubah A. Resources use, costs and effectiveness of non-penetrating deep sclerectomy according to glaucoma stage. *Arquivos brasileiros de oftalmologia* 2011; **74**(6): 400-4.

225. Chakravarti T. The Association of Socioeconomic Status with Severity of Glaucoma and the Impacts of Both Factors on the Costs of Glaucoma Medications: A Cross-Sectional Study in West Bengal, India. *Journal of ocular pharmacology and therapeutics : the official journal of the Association for Ocular Pharmacology and Therapeutics* 2018; **34**(6): 442-51.
226. Adio AO, Onua AA. Economic burden of glaucoma in Rivers State, Nigeria. *Clinical ophthalmology (Auckland, NZ)* 2012; **6**(101321512): 2023-31.
227. Studnicka J, Rihova B, Rencova E, et al. Cost and effectiveness of therapy for wet age-related macular degeneration in routine clinical practice. *Ophthalmologica Journal international d'ophtalmologie International journal of ophthalmology Zeitschrift fur Augenheilkunde* 2013; **230**(1): 34-42.
228. Hanemoto T, Hikichi Y, Kikuchi N, Kozawa T. The impact of different anti-vascular endothelial growth factor treatment regimens on reducing burden for caregivers and patients with wet age-related macular degeneration in a single-center real-world Japanese setting. *PloS one* 2017; **12**(12): e0189035.
229. Kume A, Ohshiro T, Sakurada Y, Kikushima W, Yoneyama S, Kashiwagi K. Treatment Patterns and Health Care Costs for Age-Related Macular Degeneration in Japan: An Analysis of National Insurance Claims Data. *Ophthalmology* 2016; **123**(6): 1263-8.
230. Kim S, Park SJ, Byun SJ, Park KH, Suh HS. Incremental economic burden associated with exudative age-related macular degeneration: a population-based study. *BMC health services research* 2019; **19**(1): 828.
231. Spooner KL, Mhlanga CT, Hong TH, Broadhead GK, Chang AA. The burden of neovascular age-related macular degeneration: a patient's perspective. *Clinical ophthalmology (Auckland, NZ)* 2018; **12**(101321512): 2483-91.
232. Saxena N, George PP, Hoon HB, Han LT, Onn YS. Burden of Wet Age-Related Macular Degeneration and Its Economic Implications in Singapore in the Year 2030. *Ophthalmic epidemiology* 2016; **23**(4): 232-7.
233. Athanasakis K, Fragoulakis V, Tsiantou V, Masaoutis P, Maniadakis N, Kyriopoulos J. Cost-effectiveness analysis of ranibizumab versus verteporfin photodynamic therapy, pegaptanib sodium, and best supportive care for the treatment of age-related macular degeneration in Greece. *Clinical therapeutics* 2012; **34**(2): 446-56.
234. Bandello F, Augustin A, Sahel J-A, et al. Association between visual acuity and medical and non-medical costs in patients with wet age-related macular degeneration in France, Germany and Italy. *Drugs & aging* 2008; **25**(3): 255-68.
235. Bonastre J, Le Pen C, Soubrane G, Quentel G. The burden of age-related macular degeneration: results of a cohort study in two French referral centres. *PharmacoEconomics* 2003; **21**(3): 181-90.
236. Dakin HA, Wordsworth S, Rogers CA, et al. Cost-effectiveness of ranibizumab and bevacizumab for age-related macular degeneration: 2-year findings from the IVAN randomised trial. *BMJ open* 2014; **4**(7): e005094.
237. Garattini L, Castelnuovo E, Lanzetta P, et al. Direct medical costs of age-related macular degeneration in Italian hospital ophthalmology departments. A multicenter, prospective 1-year study. *The European journal of health economics : HEPAC : health economics in prevention and care* 2004; **5**(1): 22-7.
238. Grieve R, Guerriero C, Walker J, et al. Verteporfin photodynamic therapy cohort study: report 3: cost effectiveness and lessons for future evaluations. *Ophthalmology* 2009; **116**(12): 2471-2.
239. Reich O, Schmid MK, Rapold R, Bachmann LM, Blozik E. Injections frequency and health care costs in patients treated with aflibercept compared to ranibizumab: new real-life evidence from Switzerland. *BMC ophthalmology* 2017; **17**(1): 234.
240. Schmid MK, Reich O, Blozik E, et al. Outcomes and costs of Ranibizumab and Aflibercept treatment in a health-service research context. *BMC ophthalmology* 2018; **18**(1): 64.
241. Schmid MK, Reich O, Faes L, et al. Comparison of Outcomes and Costs of Ranibizumab and Aflibercept Treatment in Real-Life. *PloS one* 2015; **10**(8): e0135050.
242. Ruiz-Moreno JM, Coco RM, Garcia-Arumi J, Xu X, Zlateva G. Burden of illness of bilateral neovascular age-related macular degeneration in Spain. *Current medical research and opinion* 2008; **24**(7): 2103-11.
243. Ke KM. The direct, indirect and intangible costs of visual impairment caused by neovascular age-related macular degeneration. *The European journal of health economics : HEPAC : health economics in prevention and care* 2010; **11**(6): 525-31.
244. Vottonen P. Anti-vascular endothelial growth factors treatment of wet age-related macular degeneration: from neurophysiology to cost-effectiveness. *Acta ophthalmologica* 2018; **96 Suppl A109**(101468102): 1-46.
245. Weyer-Wendl H, Walter P. Financial burden and quality of life of informal caregivers of patients with wet age-related macular degeneration. *Health economics review* 2016; **6**(1): 37.
246. Cruess AF, Zlateva G, Xu X, et al. Economic burden of bilateral neovascular age-related macular degeneration: multi-country observational study. *PharmacoEconomics* 2008; **26**(1): 57-73.
247. Soubrane G, Cruess A, Lotery A, et al. Burden and health care resource utilization in neovascular age-related macular degeneration: findings of a multicountry study. *Archives of ophthalmology (Chicago, Ill : 1960)* 2007; **125**(9): 1249-54.
248. Cruess A, Zlateva G, Xu X, Rochon S. Burden of illness of neovascular age-related macular degeneration in Canada. *Canadian journal of ophthalmology Journal canadien d'ophtalmologie* 2007; **42**(6): 836-43.

249. Brown MM, Brown GC, Lieske HB, Tran I, Turpcu A, Colman S. Societal costs associated with neovascular Age-related Macular Degeneration in the United States. *Retina (Philadelphia, Pa)* 2016; **36**(2): 285-98.
250. Coleman AL, Yu F. Eye-related medicare costs for patients with age-related macular degeneration from 1995 to 1999. *Ophthalmology* 2008; **115**(1): 18-25.
251. Day S, Acquah K, Lee PP, Mruthyunjaya P, Sloan FA. Medicare costs for neovascular age-related macular degeneration, 1994-2007. *American journal of ophthalmology* 2011; **152**(6): 1014-20.
252. Gower EW, Cassard SD, Bass EB, Schein OD, Bressler NM. A cost-effectiveness analysis of three treatments for age-related macular degeneration. *Retina (Philadelphia, Pa)* 2010; **30**(2): 212-21.
253. Gupta OP, Shienbaum G, Patel AH, Fecarotta C, Kaiser RS, Regillo CD. A treat and extend regimen using ranibizumab for neovascular age-related macular degeneration clinical and economic impact. *Ophthalmology* 2010; **117**(11): 2134-40.
254. Schmier JK, Halpern MT, Covert D, Delgado J, Sharma S. Impact of visual impairment on use of caregiving by individuals with age-related macular degeneration. *Retina (Philadelphia, Pa)* 2006; **26**(9): 1056-62.
255. Schmier JK, Covert DW, Lau EC. Patterns and costs associated with progression of age-related macular degeneration. *American journal of ophthalmology* 2012; **154**(4): 675-81.e1.
256. Yildirim S, Akkin C, Oztas Z, Nalcaci S, Afrashi F, Mentes J. Direct Treatment Costs of Neovascular Age-related Macular Degeneration and Comparison of Gained and/or Preserved Vision with Expenditure. *Turkish journal of ophthalmology* 2018; **48**(1): 27-32.
257. Dilokthornsakul P, Chaiyakunapruk N, Ruamviboonsuk P, et al. Health resource utilization and the economic burden of patients with wet age-related macular degeneration in Thailand. *International journal of ophthalmology* 2014; **7**(1): 145-51.
258. Varano M, Eter N, Winyard S, Wittrup-Jensen KU, Navarro R, Heraghty J. The emotional and physical impact of wet age-related macular degeneration: findings from the wAMD Patient and Caregiver Survey. *Clinical ophthalmology (Auckland, NZ)* 2016; **10**(101321512): 257-67.
259. Economics A. The Economic Impact of Diabetic Eye Disease :a dynamic economic model. Melbourne, Australia: Centre Eye Research Australia, University of Melbourne; 2008.
260. Happich M, Reitberger U, Breitscheidel L, Ulbig M, Watkins J. The economic burden of diabetic retinopathy in Germany in 2002. *Graefe's archive for clinical and experimental ophthalmology = Albrecht von Graefes Archiv fur klinische und experimentelle Ophthalmologie* 2008; **246**(1): 151-9.
261. Heintz E, Wirehn AB, Peebo BB, Rosenqvist U, Levin LA. Prevalence and healthcare costs of diabetic retinopathy: a population-based register study in Sweden. *Diabetologia* 2010; **53**(10): 2147-54.
262. Hutton DW, Stein JD, Glassman AR, Bressler NM, Jampol LM, Sun JK. Five-Year Cost-effectiveness of Intravitreous Ranibizumab Therapy vs Panretinal Photocoagulation for Treating Proliferative Diabetic Retinopathy: A Secondary Analysis of a Randomized Clinical Trial. *JAMA Ophthalmol* 2019: 1-9.
263. Brook RA, Kleinman NL, Patel S, Smeeding JE, Beren IA, Turpcu A. United States comparative costs and absenteeism of diabetic ophthalmic conditions. *Postgraduate medicine* 2015; **127**(5): 455-62.
264. Sasongko MB, Wardhana FS, Febryanto GA, et al. The estimated healthcare cost of diabetic retinopathy in Indonesia and its projection for 2025. *Br J Ophthalmol* 2019; (azk, 0421041).
265. Prajna VN, Nirmalan PK, Saravanan S, Srinivasan M. Economic analysis of corneal ulcers in South India. *Cornea* 2007; **26**(2): 119-22.
266. Frick KD, Keuffel EL, Bowman RJ. Epidemiological, demographic, and economic analyses: measurement of the value of trichiasis surgery in The Gambia. *Ophthalmic Epidemiol* 2001; **8**(2-3): 191-201.
267. Baltussen RMPM, Sylla M, Frick KD, Mariotti SP. Cost-effectiveness of trachoma control in seven world regions. *Ophthalmic Epidemiol* 2005; **12**(2): 91-101.
268. Frick KD, Hanson CL, Jacobson GA. Global burden of trachoma and economics of the disease. *The American journal of tropical medicine and hygiene* 2003; **69**(5 Suppl): 1-10.
269. Frick KD, Basilion EV, Hanson CL, Colchero MA. Estimating the burden and economic impact of trachomatous visual loss. *Ophthalmic Epidemiol* 2003; **10**(2): 121-32.
270. Redekop WK, Lenk EJ, Luyendijk M, et al. The Socioeconomic Benefit to Individuals of Achieving the 2020 Targets for Five Preventive Chemotherapy Neglected Tropical Diseases. *PLoS Negl Trop Dis* 2017; **11**(1): e0005289.
271. Kaplan RI, De Moraes CG, Cioffi GA, Al-Aswad LA, Blumberg DM. Comparative Cost-effectiveness of the Baerveldt Implant, Trabeculectomy With Mitomycin, and Medical Treatment. *JAMA ophthalmology* 2015; **133**(5): 560-7.
272. Schmier JK, Halpern MT, Covert DW, Delgado J, Sharma S. Impact of visual impairment on service and device use by individuals with age-related macular degeneration (AMD). *Disability and rehabilitation* 2006; **28**(21): 1331-7.
273. Polack S, Kuper H, Wadud Z, Fletcher A, Foster A. Quality of life and visual impairment from cataract in Satkhira district, Bangladesh. *Br J Ophthalmol* 2008; **92**(8): 1026-30.
274. Resnikoff S, Pascolini D, Etya'ale D, et al. Global data on visual impairment in the year 2002. *Bull World Health Organ* 2004; **82**(11): 844-51.

275. Resnikoff S, Pascolini D, Mariotti SP, Pokharel GP. Global magnitude of visual impairment caused by uncorrected refractive errors in 2004. *Bull World Health Organ* 2008; **86**(1): 63-70.
276. Thylefors B, Negrel AD, Pararajasegaram R, Dadzie KY. Global data on blindness. *Bull World Health Organ* 1995; **73**(1): 115-21.
277. World Bank. World Development Indicators: Employment to population ratio, 15+, total (%) (modeled ILO estimate). Washington: The World Bank Group; 2020.
278. Gordon KD, Cruess AF, Bellan L, Mitchell S, Pezzullo ML. The cost of vision loss in Canada. 1. Methodology. *Can J Ophthalmol* 2011; **46**(4): 310-4.
279. Onabolu OO, Bodunde OT, Ajibode AH, Otulana TO, Ebonhor M, Daniel OJ. Rehabilitation and paid employment for blind people in a low income country. *Journal of Advances in Medicine and Medical Research* 2018; **25** (8): 1-9.
280. American Foundation for the Blind. Reviewing the Disability Employment Research on People who are Blind or Visually Impaired: Key Takeaways. 2020. https://www.afb.org/research-and-initiatives/employment/reviewing-disability-employment-research-people-blind-visually.
281. EuroBlind Organization. About blindness and partial sight: facts and figures. 2020. http://www.euroblind.org/about-blindness-and-partial-sight/facts-and-figures2020).
282. U.S. Bureau of Labor Statistics. Persons with a disability: Labor force characteristics - 2019 Washington, 2020.
283. Blind Foundation. Blind people significantly under employed around the world. 2018. https://www.scoop.co.nz/stories/PO1811/S00173/blind-people-significantly-under-employed-around-the-world.htm.
284. Slade J, Edwards E, RNIB. Employment status and sight loss. Royal National Institute for the Blind; 2017.
285. World Bank. World Development Indicators: Population,Total. Washington: The World Bank Group; 2020.
286. World Bank. World Development Indicators: GDP per capita, PPP (current international $). Washington: The World Bank Group; 2020.
287. World Bank. World Development Indicators: GDP deflator (base year varies by country). Washington: The World Bank Group; 2020.
288. World Bank. World Development Indicators: PPP conversion factor, GDP (LCU per international $). Washington: World Bank; 2020.
289. Baltussen R, Naus J, Limburg H. Cost-effectiveness of screening and correcting refractive errors in school children in Africa, Asia, America and Europe. *Health Policy* 2009; **89**(2): 201-15.
290. Frick KD, Riva-Clement L, Shankar MB. Screening for refractive error and fitting with spectacles in rural and urban India: cost-effectiveness. *Ophthalmic Epidemiol* 2009; **16**(6): 378-87.
291. Khan A, Amitava AK, Rizvi SA, Siddiqui Z, Kumari N, Grover S. Cost-effectiveness analysis should continually assess competing health care options especially in high volume environments like cataract surgery. *Indian J Ophthalmol* 2015; **63**(6): 496-500.
292. Lansingh VC, Carter MJ. Use of Global Visual Acuity Data in a time trade-off approach to calculate the cost utility of cataract surgery. *Arch Ophthalmol* 2009; **127**(9): 1183-93.
293. Lansingh VC, Carter MJ, Martens M. Global cost-effectiveness of cataract surgery. *Ophthalmology* 2007; **114**(9): 1670-8.
294. Busbee BG, Brown MM, Brown GC, Sharma S. Incremental cost-effectiveness of initial cataract surgery. *Ophthalmology* 2002; **109**(3): 606-12; discussion 12-3.
295. Griffiths UK, Bozzani FM, Gheorghe A, Mwenge L, Gilbert C. Cost-effectiveness of eye care services in Zambia. *Cost Eff Resour Alloc* 2014; **12**(1): 6.
296. Hiratsuka Y, Yamada M, Akune Y, et al. Cost-utility analysis of cataract surgery in Japan: a probabilistic Markov modeling study. *Jpn J Ophthalmol* 2013; **57**(4): 391-401.
297. Kobelt G, Lundström M, Stenevi U. Cost-effectiveness of cataract surgery. Method to assess cost-effectiveness using registry data. *J Cataract Refract Surg* 2002; **28**(10): 1742-9.
298. Räsänen P, Krootila K, Sintonen H, et al. Cost-utility of routine cataract surgery. *Health and quality of life outcomes* 2006; **4**: 74.
299. Sach TH, Foss AJE, Gregson RM, et al. Falls and health status in elderly women following first eye cataract surgery: an economic evaluation conducted alongside a randomised controlled trial. *The British journal of ophthalmology* 2007; **91**(12): 1675-9.
300. Busbee BG, Brown MM, Brown GC, Sharma S. CME review: A cost-utility analysis of laser photocoagulation for extrafoveal choroidal neovascularization. *Retina (Philadelphia, Pa)* 2003; **23**(3): 279-4.
301. World Health Organization. Network of WHO Collaborating Centres for Trachoma: 2nd meeting report, Decatur, GA, USA, 26 June 2016 (WHO/HTM/NTD/PCT/2017.06). Geneva: World Health Organization, 2017.
302. World Health Organization. Universal coverage - three dimensions. https://www.who.int/health_financing/strategy/dimensions/en/.
303. Resnikoff S, Lansingh VC, Washburn L, et al. Estimated number of ophthalmologists worldwide (International Council of Ophthalmology update): will we meet the needs? *Br J Ophthalmol* 2020; **104**(4): 588-92.
304. Bourne R, et al. GBD/VLEG 2020 Blindness and vision impairment estimates. Submitted for publication.

305. Marsh AD, Muzigaba M, Diaz T, et al. Effective coverage measurement in maternal, newborn, child, and adolescent health and nutrition: progress, future prospects, and implications for quality health systems. *Lancet Glob Health* 2020; **8**(5): e730-e6.
306. Foster A. Cataract and "Vision 2020—the right to sight" initiative. *British Journal of Ophthalmology* 2001; **85**(6): 635-7.
307. Foster A. Hey simple method for evaluating surgical cataract services in prevention of blindness programs. *Community Eye Health* 1992; (10): 2-5.
308. World Health Organization. Informal consultation on analysis of blindness prevention outcomes. WHO/PBL/98.68. Geneva: World Health Organization, 1998.
309. World Health Organization. 13th General Programme of Work (GPW13) WHO Impact Framework. 2019. https://www.who.int/about/what-we-do/GPW13_WHO_Impact_Framework_Indicator_Metadata.pdf.
310. Ramke J, Gilbert C, Lee ACL, Ackland P, Limburg H, Foster A. Effective cataract surgical coverage: an indicator for measuring quality-of-care in the context of Universal Health Coverage *PLoS One* 2017; **12**(3): e0172342.
311. McCormick I, Mactaggart I, Bastawrous A, Burton MJ, Ramke J. Effective refractive error coverage: an eye health indicator to measure progress towards universal health coverage. *Ophthalmic Physiol Opt* 2020; **40**(1): 1-5.
312. Lee CN, Ramke J, McCormick I, et al. Are we advancing universal health coverage through cataract services? Protocol for a scoping review. *BMJ open* 2020; **10**(7): e039458.
313. Ramke J, Kyari F, Mwangi N, Piyasena M, Murthy G, Gilbert CE. Cataract Services are Leaving Widows Behind: Examples from National Cross-Sectional Surveys in Nigeria and Sri Lanka. *International Journal of Environmental Research and Public Health* 2019; **16**(20): 3854.
314. Costello A, Abbas M, Allen A, et al. Managing the health effects of climate change: Lancet and University College London Institute for Global Health Commission. *Lancet* 2009; **373**(9676): 1693-733.
315. Stocker TF, Qin G-K, Plattner LV, et al. Climate Change 2013: The Physical Science Basis. Contribution of Working Group I to the Fifth Assessment Report of the Intergovernmental Panel on Climate Change *Cambridge University Press, Cambridge, United Kingdom and New York, NY, USA* 2013.
316. Karliner J, Slotterback S, Boyd R, Ashby B, Steele K. Health Care's Climate Footprint: How the Health Sector Contributes to the Global Climate Crisis and Opportunities for Action: Healthcare Without Harm. https://www.arup.com/-/media/arup/files/publications/h/health-cares-climate-footprint.pdf accessed 2020/04/20. 2019.
317. Watts N, Adger WN, Agnolucci P, et al. Health and climate change: policy responses to protect public health. *Lancet* 2015; **386**(10006): 1861-914.
318. National Health Service Digital. Hospital Outpatient Activity 2018-19. 2019. https://digital.nhs.uk/data-and-information/publications/statistical/hospital-outpatient-activity/2018-19.
319. DEFRA (Department of the Environment FaRAU. Specification for the Assessment of the Life Cycle Greenhouse Gas Emissions of Goods and Services. Carbon Trust. Publicly Available Specification (PAS) 2050. 2011.
320. Thiel CL, Schehlein E, Ravilla T, et al. Cataract surgery and environmental sustainability: Waste and lifecycle assessment of phacoemulsification at a private healthcare facility. *J Cataract Refract Surg* 2017; **43**(11): 1391-8.
321. Morris DS, Wright T, Somner JE, Connor A. The carbon footprint of cataract surgery. *Eye (Lond)* 2013; **27**(4): 495-501.
322. Haripriya A, Chang DF, Namburar S, Smita A, Ravindran RD. Efficacy of Intracameral Moxifloxacin Endophthalmitis Prophylaxis at Aravind Eye Hospital. *Ophthalmology* 2016; **123**(2): 302-8.
323. Haripriya A, Chang DF, Reena M, Shekhar M. Complication rates of phacoemulsification and manual small-incision cataract surgery at Aravind Eye Hospital. *J Cataract Refract Surg* 2012; **38**(8): 1360-9.
324. Thiel C, Cassells-Brown A, Goel H, et al. Utilizing off-the-shelf LCA methods to develop a 'triple bottom line' auditing tool for global cataract surgical services. *Resources, Conservation & Recycling* 2020; **158**(104805): 1-7.
325. Somner JE, Cavanagh DJ, Wong KK, Whitelaw M, Thomson T, Mansfield D. The precautionary principle: what is the risk of reusing disposable drops in routine ophthalmology consultations and what are the costs of reducing this risk to zero? *Eye (Lond)* 2010; **24**(2): 361-3.
326. Tauber J, Chinwuba I, Kleyn D, Rothschild M, Kahn J, Thiel CL. Quantification of the Cost and Potential Environmental Effects of Unused Pharmaceutical Products in Cataract Surgery. *JAMA Ophthalmol* 2019.
327. Levesque JF, Harris MF, Russell G. Patient-centred access to health care: conceptualising access at the interface of health systems and populations. *Int J Equity Health* 2013; **12**: 18.
328. Yashadhana A, Zhang JH, Yasmin S, et al. Action needed to improve equity and diversity in global eye health leadership. *Eye (Lond)* 2020; **34**(6): 1051-4.
329. Shannon G, Jansen M, Williams K, et al. Gender equality in science, medicine, and global health: where are we at and why does it matter? *Lancet* 2019; **393**(10171): 560-9.
330. Kang SK, Kaplan S. Working toward gender diversity and inclusion in medicine: myths and solutions. *Lancet* 2019; **393**(10171): 579-86.
