# Supplementary material for: The Lancet Global Health Commission on Global Eye Health: vision beyond 2020
Source: Lancet Glob Health. 2021 Feb 16;9(4):e489–551. doi: 10.1016/S2214-109X(20)30488-5 (PMC7966694; doi:10.1016/S2214-109X(20)30488-5)
Supplement: Supplementary appendix 2 [file mmc2.pdf]

THE LANCET
Global Health

Supplementary appendix 2

This appendix formed part of the original submission and has been peer reviewed. We post it as supplied by the authors.

Supplement to: Burton MJ, Ramke J, Marques AP, et al. The *Lancet Global Health* Commission on Global Eye Health: vision beyond 2020. *Lancet Glob Health* 2021; published online Feb 16. http://dx.doi.org/10.1016/S2214-109X(20)30488-5.

The Lancet Global Health

Supplementary appendix 2 – Case Studies

The appendix formed part of the original submission and has been peer reviewed.
We post it as supplied by the authors.

Supplement to: Burton MJ, Ramke J, Marques AP, et al. Lancet Global Health Commission on Global Eye Health: Vision Beyond 2020

Table of Contents

Case Study 1: The Cataract Impact Study – Kenya, Bangladesh and the Philippines 3
Case Study 2: Improving economic productivity by correcting presbyopia in India: the PROSPER trial 4
Case Study 3: Ministry of Education School Health Programme, Chile 5
Case Study 4: Integrating with education - China's National Myopia Plan 6
Case Study 5: Lessons Learnt from the Lady Health Worker Programme, Pakistan 7
Case Study 6: Integrating eye care for children into primary health care in Tanzania 8
Case Study 7: The UK National Diabetic Eye Screening Programme 9
Case Study 8: Vision Centre's in India - Supportive practices and enabling environments 10
Case Study 9: Eye care seeking journeys in Uganda 11
Case Study 10: Maternal and childcare ROP programme in Argentina 12
Case Study 11: Developing the eye health workforce in the Pacific 13
Case Study 12: mHealth enabled eye health screening programme in Pakistan 14
Case Study 13: Tele-ophthalmology and artificial intelligence for diabetic retinopathy screening 15
Case Study 14: Artificial intelligence in retinal optical coherence tomography (OCT) imaging 16
Case Study 15: Eye Health Financing in Peru 17
Case Study 16: Eye health financing in Rwanda 18
Case Study 17: Cameroon Cataract Development Impact Bond 19
Case Study 18: Eye health inequality in the USA 20
Case Study 19: Indigenous eye health programme in Australia 21
Case Study 20: The development of Eye Health Services in China, 1949 - 2020 22
Case Study 21: Personal stories of eye health 23
References 28

Case Study 1: The Cataract Impact Study – Kenya, Bangladesh and the Philippines

Cataract surgery is among the most cost-effective of health interventions.[1] It is undertaken to improve vision, on the premise that this will make people's lives better. However, historically, data on the impact of cataract surgery were lacking for LMICs. Consequently, the Cataract Impact Study was undertaken in Kenya, Bangladesh, and the Philippines to assess the impact of cataract surgery on poverty, quality of life (QoL) and everyday activities.[2-10]

Approximately 700 people aged ≥50 years with vision impairment from cataract (visual acuity <6/24 in the better eye) were identified across the three countries. Appropriate controls were selected for each case. Participants were interviewed about their health-related quality of life, participation in different daily activities, and household economic situation. People with cataract were then offered free or subsidised surgery and transport and were visited up to four times in their homes to encourage surgery uptake. Participants were re-interviewed using the same tools after one year in all three countries and again after six years in Bangladesh and the Philippines.[11]

Before surgery people who were vision impaired from cataract were on average less well off, whether poverty was measured in terms of monthly expenditure, asset ownership, or self-perceived wealth.[9] They were more likely to need help from others, and less likely to take part in productive activities, such as employment outside the home, housework, or looking after children.[5] People with vision loss reported poorer vision-related QoL and health-related QoL than controls.[2,6,7]

The outcomes of people who received cataract surgery had improved within one year.[10] Monthly expenditure, as a proxy for income, had increased compared to baseline among operated cases (36% increase in Kenya, 44% in Bangladesh, 88% in the Philippines).[10] Operated cases spent more time on productive activities, needed less assistance and reported better QoL compared to baseline.[3,4] Many gaps in well-being and poverty between those with cataract and controls had been closed within one year of cataract surgery. By follow-up, there was no significant difference in per capita expenditure compared to controls.[10]

These improvements were still apparent six years after surgery in Bangladesh and the Philippines. Cases had enjoyed sustained economic benefits, in terms of improved household expenditure and asset ownership.[8] Compared to baseline, they also maintained a higher QoL, more independence, better functioning and greater involvement in productive activities. The conclusions of the Cataract Impact Study were that people with vision impairment from cataract experience poverty, reduced QoL and difficulties in daily activities. However, after surgery they may regain the same levels as their peers.

Case Study 2: Improving economic productivity by correcting presbyopia in India: the PROSPER trial

The presumed causal association between good health and productive work is a cornerstone of global health policy, but until recently only a handful of randomised trials had examined the question of whether health interventions could improve workplace productivity.[12-16] Among these, the majority concerned nutritional interventions,[13-16] and only two had statistically-significant effect sizes, both <15%.[12,15]

Presbyopia, the nearly-universal decline in unaided near vision with ageing, affects over 1 billion people globally.[17] Beginning at around the age of 40 years, presbyopia becomes apparent at the height of the productive working years.[18] Although presbyopia is safely, effectively and inexpensively treated with glasses, as few as 10% of affected persons in LMICs have the spectacles they need for normal near vision.[19,20]

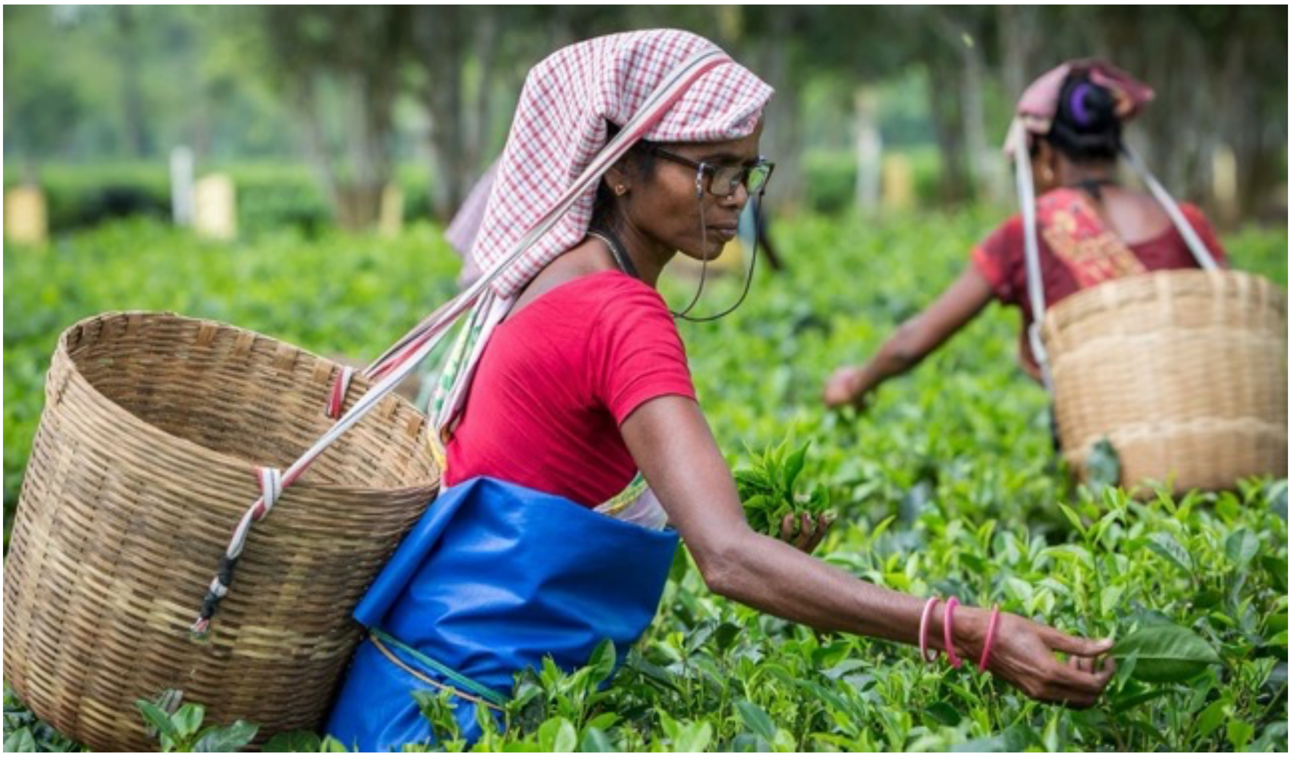

PROSPER (PROductivity Study of Presbyopia Elimination in Rural-dwellers) was the first randomized trial to address whether provision of near-vision spectacles to workers with presbyopia could improve workplace productivity.[21] When spectacles correcting near vision at the usual working distance were provided in random fashion to 751 Indian tea workers aged 40 years and above, mean intervention group productivity (daily tea picked) increased from 25·0 to 34·8 kg/day (9·84 kg/day), which was significantly more than controls (26·0 to 30·6, 4·59 kg/day, P<0·001), a difference of 5·25 kg/day (21·7%, Cohen's d effect size=1.01, 95%CI 4·50-5·99, P<0·001). The intervention cost was low ($10·20 per person), acceptance was high (nearly 85%), presbyopia was common (>50%) among workers aged ≥40 years, and a significantly greater effect of the intervention was observed among older participants in this age-related condition, enhancing biological plausibility. Nearly 95% of those given spectacles reported that they would buy a new pair if theirs were lost or broken.

Provision of near-vision spectacles to older workers may be a scalable and sustainable strategy for poverty alleviation. Innovative strategies are needed to increase access for underserved communities. Correction of even modest degrees of presbyopia led to significantly improved productivity in this setting. Further trials in other work environments and countries are currently underway.

Case Study 3: Ministry of Education School Health Programme, Chile

To raise enrolment, academic performance and prevent students' withdrawals from public schools, the government of Chile delegated the Ministry of Education (MoE) to implement the national school health programme that includes nutrition, vision, hearing, oral and mental health.[22] The programme has been financed by the annual national general budget for more than two decades.[23] The programme produces health care protocols and guidelines[24] aligned with the national health strategies.[25-27] Each municipality has a programme coordinator organizing health promotion and prevention, each school coordinates detection and referrals to public and private health services contracted by tender by the MoE.[22,23]

The school health program uses the integrated people-centred health services approach, engaging the school community by delivering effective health education and empowerment of teachers, parents, and students. Services for detection and referrals of vision impairment and other eye diseases are provided in most public schools in remote areas in all districts and there is no financial burden for the low-income people.

In 2019, the MoE information system reported 111,712 students at 1st and 5th grades had a visual screening, 117,530 spectacles were provided, and 387 children with low vision received aids and rehabilitation. The compliance with spectacle wear in school children is greater than studies in other parts of the world.[28,29] The school community commitment with the health program is high and reports a good perception of access and quality of health services.[30]

In Chile, the school health program led by the MoE, brought eye care to the schools together with other health interventions to improve child development and life trajectories. The health care whole-of-government approach secured financial sustainability, commitment, and engagement by all sectors and recognizes people as participants and beneficiaries of these services.

Case Study 4: Integrating with education - China's National Myopia Plan

The Chinese government has recognized that the high prevalence of childhood myopia constitutes a major public health problem of broad concern. A recent joint national study of more than a million children by the National Health Commission and Ministry of Education has shown that over half of Chinese children were myopic in 2018, reaching 80% by senior high school.

The government's comprehensive national plan for the prevention and control of myopia among children and adolescents, launched by President Xi Jinping in 2018, emphasizes collective action by society as a whole, including schools, eye care facilities, families, and students themselves, as well as coordinated input from eight government departments. The plan targets an annual reduction of 0.5% in childhood myopia between 2018 and 2023.

The main focus of the plan is on myopia prevention. Schools will play a crucial role, with a specific call to increase time spent outdoors during the classroom day to 1-2 hours daily, to reduce myopia onset. Other required measures for schools include lessening the academic burden, including a total absence of written homework in the first two years of elementary school. This is designed to reduce near work, an important factor leading to China's current high myopia prevalence. Such preventive measures can also help to prevent high myopia (6D or more), which affects 10-20% of China' high school graduates and elevates the risk of macular atrophy, glaucoma and other causes of severe vision impairment.

As the proportion of children receiving schooling increases in response to SDG4, other countries will likely face growing prevalence of childhood myopia and may increasingly look to China's national program for clues on how to coordinate multi-sectorial efforts and take effective action.[31]

Case Study 5: Lessons Learnt from the Lady Health Worker Programme, Pakistan

The Lady Health Workers (LHW) Programme was established in Pakistan in 1994 to ensure the provision of primary, preventative, promotive, and curative care services and support health system at the household and community levels. LHWs are fully recognised members of the public sector health workforce and deployed throughout all five provinces. Following her training, an LHW is expected to deliver primary health care services and carry out maternal, newborn and child health services – their role is also focused on increasing the coverage of immunisation and promotion of health education, nutrition and basic sanitation.

The role of LHWs in eye care has been a catalyst to increase access to primary eye care, particularly in remote and underprivileged communities. By bringing primary eye care services closer to the communities, LHWs play a key role in influencing women's health-seeking behaviour, which also directly impacts health and education outcomes for children. An external evaluation highlighted that the population served by LHWs had substantially better health than the population without LHWs.[32]

Key lessons that have been drawn from the LHW Programme are as follows:

1. The programme is implemented in close **collaboration with all National Priority Programmes** and within the existing public health infrastructure to **ensure sustainability and scale up**.
2. MoH used a **phased scale-up strategy** to gradually expand the programme to its current level both in context of **geographical coverage** and **scope of interventions**.
3. A **well-defined and participatory recruitment strategy** fostered community ownership by allowing community members to identify the most suitable LHWs and support the implementation of essential interventions.
4. The programme has **strong networks** at the federal, provincial, and district levels, with each level having clear responsibilities. This results in having **effective and efficient referrals mechanisms** in place and facilitates the **continuum of care**.
5. **Gender-responsiveness** of the programme has been vital to build communities' trust and confidence. Using women in this role is very helpful in a country such as Pakistan, where direct interaction between women and men is not encouraged. LHWs have the advantage of being able to visit women in their homes independently and without challenging any social norms.
6. **Empowerment of women** and improvement of the well-being of LHWs is a positive by-product of the programme. In terms of motivation, social recognition in the form of performance-related rewards and respect from the community has been an important motivator for LHWs' long association with the programme.

Case Study 6: Integrating eye care for children into primary health care in Tanzania

Corneal blindness in children in LICs can be prevented by evidence-based interventions, such as vitamin A supplementation, measles immunization, and ocular prophylaxis of the newborn.[33] Treatable conditions, such as cataract, glaucoma and retinoblastoma need to be detected and managed early to improve outcomes. Recognising that primary health care (PHC) has a key role in delivering these strategies, in 2002 WHO recommended ten activities to improve child eye health, for delivery by PHC workers.[34]

Ten key activities to promote healthy eyes in children, for integration within Maternal and Child Health services.

**Control of conditions which can be associated with vision loss**	
Vitamin A deficiency	1. Give vitamin A supplements to children routinely
	2. Give vitamin A supplements to mothers after delivery
Measles	3. Promote breast feeding and good nutrition
	4. Give vitamin A supplements to children with measles or malnutrition.
	5. Immunise children against measles
**Control of ocular conditions**	
Conjunctivitis of the newborn	6. Clean the eyes of babies at delivery and apply antibiotic drops
Trachoma	7. Keep children's faces clean
Cataract/retinoblastoma/ocular abnormalities	8. Refer children with poor vision, white pupils or ocular abnormality to an eye worker
Traditional eye remedies	9. Avoid the use of traditional eye medicines
Trauma	10. Refer children with history of injury to an eye worker

In 2010 a pilot study was undertaken in urban Tanzania in which staff in primary reproductive child health clinics were trained in the ten activities to prevent or detect eye conditions.[35] Before training, staff had poor knowledge of eye conditions and how to manage them, and lacked confidence in examining children and knowing which to refer. After training their knowledge and skills improved, with improvements largely maintained at one year. Staff changed practices, reinstating ocular prophylaxis for newborns and including eye conditions in health education for mothers.

Subsequently, a larger mixed methods study was undertaken in rural Tanzania, with a view to using the findings to advocate for eye conditions to be integrated into routine PHC for children. Key findings were that staff and mothers had poor knowledge of eye conditions, staff had inadequate case management of eye conditions, and only 24% of children aged 9-24 months had documented evidence of receiving ≥1 dose of vitamin A. Health education did not include eye conditions and staff did not counsel carers about eye problems. Service delivery was sometimes hampered by stock-outs of measles vaccine and vitamin A supplements.

In 2018, the Tanzanian Ministry of Health included a new module on eye conditions in the in-service training curriculum for the Integrated Management of Newborn and Child Health (IMNCH), WHO's global programme for facility based PHC for young children. The eye module was developed in collaboration with the Ministry of Health and IMNCI trainers. A subsequent study showed that training PHC workers in IMNCI including eye care improved their knowledge and skills and was acceptable. The eye module is now fully integrated into routine IMNCI training in Tanzania, undertaken district by district, targeting to train of >60% of all IMNCI staff. More than 2000 PHC workers have been trained to date.

Case Study 7: The UK National Diabetic Eye Screening Programme

The UK National Diabetic Eye Screening Programme (NDESP) has developed steadily since its inception in 2003 when the Delivery Strategy for the National Service Framework for Diabetes announced its establishment in all four nations.[36] While the oversight remains with the UK's National Screening Committee, all NDESPs are delivered locally to provide reliable access within communities in which patients live. Using this approach, NDESP succeeded in placing 85% of eligible people with diabetes in the appropriate clinical pathway.[37] NDESP adheres to nationally agreed quality standards, which have changed substantially as the programme matured.[38] The main themes of the screening process are covered: population coverage and screening-uptake, utilising digital imaging for screening-grading and ensuring timely administration of correct diagnosis and treatment.[38] Continuous failsafe and quality assurance measures are in place to monitor patient safety and drive improvement.

People with diabetes over the age of 12 years who are registered with a general practitioner (GP) are eligible for NDESP. They are called for annual screening based on digital photography. Screening takes place in a variety of settings, such as GP surgeries, health centres, community hubs, optometry practices or in hospitals (usually parallel with diabetes/eye clinics). Image grading is undertaken centrally by trained and accredited graders, adhering to a strict national grading protocol with quality control measures embedded. The results are sent to the patient, their GP and if required, to hospital eye services for timely treatment. Where integration with electronic care records allows it, the final screening results are deposited there to benefit joined-up care.

Most people with diabetes have a yearly screening, but there are established pathways for closer monitoring of at-risk groups (such as pregnant women) or those whose diabetic eye disease does not require treatment. Trained slit lamp operators examine those with ungradable images. Once treatment in hospital eye services has been completed, patients are referred back to NDESP for regular monitoring; this helps to create capacity in hospital eye-clinics and enables patients to be followed up in their community.

As the main aim of diabetic eye screening programmes is to prevent vision loss by enabling timely treatment of sight-threatening diabetic eye disease, monitoring vision impairment and blindness data is paramount. In the UK, as a result of NDESP, no doubt coupled with improvements in diabetes care in general, diabetic eye disease is no longer the leading cause of blindness in the working age-group, with far reaching consequences for the patient and their families, the healthcare system and society.[39]

Case Study 8: Vision Centre's in India - Supportive practices and enabling environments

Aravind Eye Hospitals in South India run a very effective network of 80 Vision Centres which are attended by approximately 700,000 patients annually. Each vision centre is staffed by a team of two, who are not only competent but are deeply committed to providing eye care to everyone in the community served by the Vision Centre. Their motivation and commitment could be attributed to the following, which have ensured high performance, patient satisfaction and low attrition:

- Clarity of what is expected of staff. Their tasks and clinical protocols are very well-defined. They have all the equipment and technology required to perform these tasks. There is a monthly routine to ensure that equipment is in good working condition. The annual attrition rate is typically between 5% and 10%.
- The staff are from the community that they serve. The opportunity to serve their own people adds to their commitment as well as giving them a status in the community. Such respect is maintained by providing good care which is appreciated by the community. This social reinforcement is motivating.
- Such respect is sustained only when they provide good quality care and this subtle community pressure helps.
- There is continuous ongoing support and feedback on every patient seen though telemedicine consultation and monthly benchmarked feedback on performance and patient satisfaction.
- Staff have access to career development training that enriches their work. For example, technicians now routinely do fundus photography and use cloud-based artificial intelligence support for instant interpretation images.
- Patient satisfaction is measured daily on a 10% sample of patients. These are compiled every month and presented to each Vision Centre along with the satisfaction scores of other Vision Centres. This provides some intrinsic motivation for continuous improvement and also leads to appropriate enhancements to training. More than 99% of patients rate the service as good or excellent.
- Each Vision Centre sees an average of 30 outpatients each day. A technician undertakes a comprehensive eye examination, records all the findings in an electronic medical record, and engages with the patient in a live telemedicine consultation with an Aravind ophthalmologist in the base hospital. The technician is supported by an administrator who is responsible for all non-clinical activities.

Case Study 9: Eye care seeking journeys in Uganda

A study from Uganda documented the routes and times taken by a cohort of people who developed severe, painful, sight-threatening microbial keratitis (corneal infection) to navigate through the health system and access appropriate assessment and treatment.

The health facilities visited before reaching the hospital eye unit included health centres, small clinics and pharmacies. All times are the median number of days (total range) for each step. Cumulative values combine the time for all steps in a specific route. Many people visited multiple health facilities before finally reaching the eye unit for assessment and definitive management. Adapted from Arunga et al.[40]

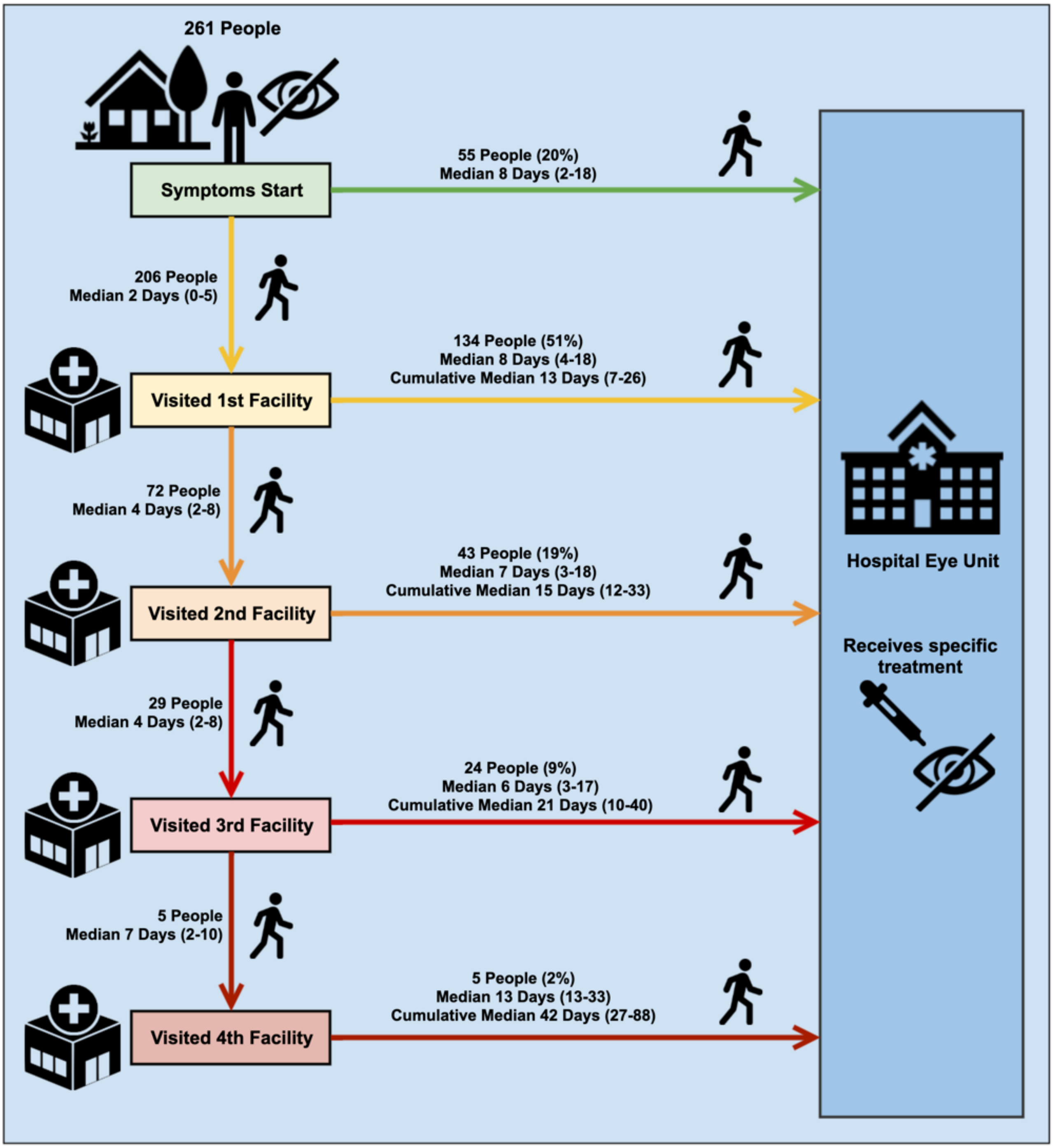

Case Study 10: Maternal and childcare ROP programme in Argentina

Retinopathy of Prematurity (ROP) blindness can be avoided by interventions which reduce preterm birth, high quality neonatal care, and by early detection and laser treatment. The maternal and child care programme at the Ministry of Health of Argentina created a multidisciplinary ROP Collaborating Group in 2003 composed of neonatologists, nurses, ophthalmologists and professional associations to increase their participation in implementing policies to improve service delivery at the neonatal intensive care units (NICU).[41] As a result of their leadership and advocacy efforts, by 2016 the government was supporting the cost of screening and treatment, had legislation mandating the eye examination of preterm infants, produced an annual report of the ROP monitoring system (issued by the Ministry of Health since 2004)[42] and updated the national clinical guidelines using the GRADE method.[43] The ROP programme in Argentina is one of two in South America that covers all national districts, 114 neonatal intensive care units, comprising most of the government sector.[44]

From 2004 to 2016, the mean birth weight of newborns with any ROP decreased by 200 grams (g) and the average gestational age reduced by 3 weeks. In addition, the proportion of babies treated for ROP with birth weight >1500 g or gestational age >32 weeks progressively declined from 30% to 17 % (44/253) in 2016.[45]

The ROP programme in Argentina is an example of a successful integrated eye care initiative lead by the maternal and child health programme at the Ministry of Health that has been effectively embedded within the health and legal systems.[41] Preterm children receive a continuum of care interventions covering promotion, prevention, treatment, and rehabilitation because of a coordination across the different levels and sites of care.

Case Study 11: Developing the eye health workforce in the Pacific

The 21 Pacific Island Countries are home to <1% of the world's population, scattered across a third of the earth's surface. Pacific geography makes health service delivery difficult and expensive. Moreover, socioeconomic changes, rapidly increasing non-communicable diseases, communicable disease outbreaks and health effects of climate change, further impact eye health. Overall workforce constraints de-prioritises eye health, particularly in the smaller island countries as major health disciplines need to be filled first. Furthermore, the absence of appropriate training facilities and postgraduate ophthalmology programmes historically contributed to very limited ophthalmic human resources in the Pacific.

Previously there was a heavy reliance on overseas visiting teams; allied ophthalmic personnel and primary eye health workers provided basic eye care between team visits. In Papua New Guinea, registered nurses underwent a 10-week training course and graduated with a certificate as a basic eye care nurse. They were equipped to work independently in rural areas.[46] In Vanuatu and the Solomon Islands, medical assistants or registered nurses were trained to deliver basic eye health services and minor ocular surgery.[47] More recently, targeted training for regional diabetic retinopathy (DR) and trachoma programmes led to training of primary eye care workers in diabetes eye disease, DR technicians and trachoma master graders.

Ophthalmology training is now offered at the University of Papua New Guinea and Fiji National University. The Pacific Eye Institute in Suva is responsible for curriculum development and training. For nurse training, the Postgraduate Diploma in Eye Care is conducted in Papua New Guinea at Divine Word University, Madang and in Fiji at the Pacific Eye Institute.[48] A collaboration with the Royal Australian and New Zealand College of Ophthalmologists (RANZCO) was key in the curriculum review processes. Furthermore, RANZCO supported sub-specialist teaching, leadership and continuing professional development, as well as provision of external examiners. A workforce support programme provides graduates with assistance in advocacy and clinical or technical support upon their return home.[48] By the end of 2019, eight countries had at least one fully qualified local ophthalmologist, with the most populous countries each having more than four ophthalmologists, giving a total of 23 active Pacific ophthalmologists.

Local training facilities with international standards that focusing on Pacific eye health needs have built up a local workforce who understand the need and deliver appropriate eye health services. With the arrival of COVID-19 teaching has moved online. New technologies (such as artificial intelligence to assist diagnosis) offer new opportunities to deliver more high-quality services to the population.

Case Study 12: mHealth enabled eye health screening programme in Pakistan

In 2018, Pakistan initiated a mHealth enabled eye health programme, involving Lady Health Workers conducting community-based vision and eye health screening. This programme uses smartphone-based data capture and cloud computing to track screening coverage, disaggregated by gender, referral adherence and case mix at the tertiary level. The figure below shows the increasing number of people receiving vision screening services over time, from an initial 724/month, to >10,000/month by early 2020, followed by a precipitous drop once paused due to the COVID-19 pandemic.

Early in the programme a gender bias was observed, with women comprising 82% of vision screening events. This was attributed to female health workers carrying out the screening, and this was a barrier to men accessing services. The addition of male social organisers resulted in a more balanced gender distribution within a few months.

Uptake of referral (figure) was tracked in real-time and service improvements made to promote uptake, including the use of automated messaging, appropriate advice and counselling at each level of the service, in response to regular data reviews and decision forums. Data also drove targeted human resource decisions including the recruitment of optometrists and equipment procurement, which was done in May 2019 in response to a bottleneck in refractive services. Growth in referrals and triage occurred once supply and demand had been equilibrated.

At baseline, 41% of tertiary consultations were refractive error related, despite these services being available in the community. While screenings and referrals continuously increased, the proportion attending tertiary consultations with refractive error dropped to below 1%; the rest were cared for in the community

By establishing the digital system enabled eye health screening programme, the population in need was effectively reached. The ability to assess health system performance in real-time allowed timely identification of bottlenecks and groups of people underserved. This guided decision making, leading to service delivery improvements that could be tracked and evaluated further.

Period covered, November 2018 to June 2020. Numbers of people screened in the community, referred to the eye clinic, and attended the eye clinic after being referred. Note the effect of COVID-19 in April, May and June 2020.

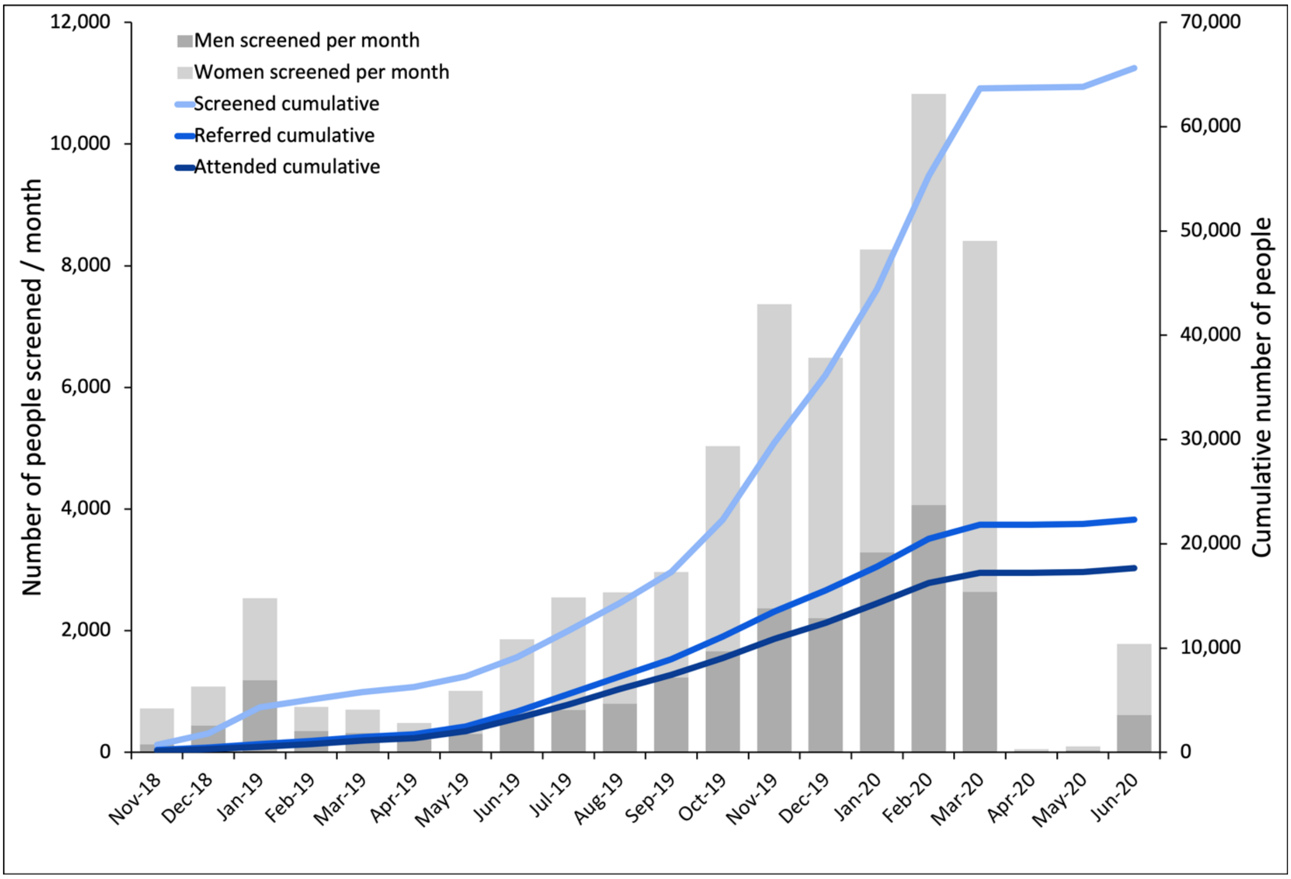

Case Study 13: Tele-ophthalmology and artificial intelligence for diabetic retinopathy screening

The International Diabetes Federation estimates that currently there are approximately 463 million people living with diabetes worldwide, requiring regular diabetic eye care, preferably by imaging the retina, in order to provide timely treatment to those with sight threatening disease.[49] Since 2000, in many parts of the world teleophthalmology using mobile fundus cameras have enabled a large proportion of those requiring retinal photography to be imaged.[50,51] However, the human workforce skilled in analysing these images is insufficient in size, with marked distributional imbalances between populations in need and practioners.[50,52]

Artificial intelligence has the potential to fill the gap for safe and timely provision of image analysis for diabetic eye disease. Using AI in diabetic eye care is compelling on several levels as the disease, its risk factors and its progression to sight-threatening stages are well characterized and understood from landmark studies.[53] In an ideal world, AI would provide assistance to diabetic eye care on multiple levels, none of which have been fully realised so far.[54,55]

There are numerous AI systems in different stages of development and regulatory approval providing either automated or computer-assisted detection of diabetic eye disease.[55-57] However, their design, validation, classification systems, decision-making capability, and the cost-effective incorporation into existing healthcare systems are some of the issues with which many countries are currently struggling.[56] The main focus of research and implementation strategy is to identify lesions on images taken by certain fundus cameras, however, their inability to generalize their learning to different camera systems and different populations is a major limitation, one for which solutions have only recently started to be developed.[58] An ideal system would be able to evaluate image quality at the time of acquisition, alert the technician to sub-optimal image quality and the need to repeat image acquisition, and detect disease progression in a series of images. In addition to the technical limitations, patients' and healthcare professionals' concerns on quality and repeatability and the medico-legal issues arising from misdiagnosis must be addressed so AI can become an integral part of diabetic eye care safely.[59]

Case Study 14: Artificial intelligence in retinal optical coherence tomography (OCT) imaging

Optical coherence tomography (OCT), first described in 1991, is a form of medical imaging analogous to ultrasonography but which measures the reflections of light waves from tissue rather than the echoes of sound.[60] In doing so, it generates high-resolution cross-sectional images of tissue in a quick and non-invasive manner, and has become a standard of care for retinal diseases and glaucoma. While predominantly still used in hospital settings, OCT imaging is increasingly widespread in community eye care, and prototype OCT systems are being developed for use by patients themselves in the home.[61] Binocular OCT systems are also in development, offering the possibility of automated, comprehensive eye examinations.[62]

With such large volumes of data being generated in several settings, retinal OCT imaging is particularly amenable to analysis using deep learning.[63] Much of this initial work has focused on automated, quantitative evaluation of OCT images.[64] These systems delineate or “segment” morphologic compartments of interest (e.g. vitreous, retina and choroid), retinal sublayers, or disease features (e.g. intraretinal fluid), and then generate tissue volumes and/or thickness maps. These measurements can then be used in clinical practice to guide treatment, in clinical trials as surrogate endpoints, and in exploratory research as prognostic indicators. More recent work has focused on the clinical classification of OCT scans for the purposes of retinal disease triage and to assist in the diagnosis of glaucomatous optic neuropathy.[65,66] A number of these systems are in commercial development and seem likely to be in widespread use in the coming years.

New applications for retinal OCT are rapidly emerging. Using deep learning, it is possible to predict, in a clinically actionable time frame, the future progression of diseases such as age-related macular degeneration.[67] Cross-modality training of deep learning systems has also shown promise for improving the diagnostic accuracy of simple imaging modalities using labels from a more advanced modality (e.g., using OCT-derived gold standard labels for training of deep learning models on fundus photographic images in diabetic macular oedema and glaucoma).[68,69]

Case Study 15: Eye Health Financing in Peru

Health financing in Peru combines general government funding, social health insurance, private insurance and out-of-pocket payments. In 2017, 44.4% of the population was covered by the government financing system (Seguro Integral de Salud, SIS), 24.8% by social health insurance (EsSalud) and 5.1% by other health insurance schemes.[70] About 25% was out-of-pocket. EsSalud has provided comprehensive ophthalmology services to affiliated employees, retirees and independent workers since 1999. The Seguro Integral de Salud (SIS) is a health care financing system subsidised entirely by the Government since 2001, covering eye care priority interventions through public services, to people without health insurance and living in poverty.[71] In 2009, cataract operations, glaucoma, refractive errors, diabetes and retinopathy of prematurity were included in the health care priorities for SIS.

In 2019 the Peruvian Government allocated US$5.3 billion on health care (10.8% of the national budget), including US$17 million for priorities in eye care. This represents only 0.3% of government health funding being directed to eye care priority procedures through SIS.[72] This budget allocation uses historical patterns related to service utilization. It prioritises strategic interventions such as screening and treatment of cataract (61% of the eye care budget), refractive error (31%), retinopathy of prematurity (5%) and glaucoma (3%).[72] SIS pays public hospitals on a "fee for service" basis, providing a financial incentive for increased productivity. The billing process also improves the use of the local health information systems and some public hospitals report improved productivity.[71]

Health promotion and education activities for NCDs within primary health care were allocated US$12 million in the 2019 budget, including mental health, hypertension, diabetes and eye health.[72] Funds are allocated to local government health services on a per capita basis, combined with health performance results.[71] Good integration between eye care and primary health care services is crucial to ensure some of this budget is used to promote eye health.

The Government of Peru is investing financial resources in eye health promotion at primary level and service provision for priority procedures, this has contributed to the increasing number of people receiving services at the public hospitals in recent years by reducing financial barriers and incentivizing increased service productivity. For example, 34,885 cataract surgeries were done by the public services (59.0% of all cataract surgeries) in 2018, compared to 14,894 in 2014 (50.6%).[72]

Case Study 16: Eye health financing in Rwanda

The Health Sector in Rwanda has been characterized by successful innovations in health financing, leading to marked improvements in the coverage and financial protection of the population. Overall, the Government of Rwanda health spending (as a proportion of total budget) increased from 8% in 2005 to around 17% in 2018.[73] There are two major health insurance schemes, the Community Based Health Insurance (CBHI) scheme and the Rwanda Social Security Board (RSSB) Public Service Insurance, which cover around 90% of the population. OOP expenses are low at around 8%.[73] However, in 2015 a review of health financing revealed a high reliance on external funding (about 60%), untapped opportunities for local resource mobilization, and a need for active engagement with private sector providers. The CBHI, which serves the poorest, was being run at a deficit, despite some cross subsidy between different insurance schemes.

These findings led to multiple reforms to improve equity and financial sustainability, building on previous developments over recent years and the development of a new Health Finance Strategic Plan for 2018-2024.[73] The fragmentation of health insurance schemes was addressed by merging the management of CBHI and RSSB. This increases the coordination in purchasing services and enables greater emphasis on quality and efficiency. Essential health care packages have been defined based on the burden of disease, with an emphasis on responding to the needs of the most vulnerable members of society and on cost-effectiveness. Service quality is an important criterion for contracting with service providers within the Accreditation Framework for Health Facilities and Performance Based Financing (PBF). The proportion of the population covered increased by making health insurance mandatory by law in 2016. The amounts paid are dependent on socio-economic status. Additional efforts are being made to raise more revenue through several taxation mechanisms.

From 2019, the Government of Rwanda, Ministry of Health, included eye health indicators in their performance-based financing system. This includes indicators for eye health consultations at health centres, referrals from health centres, cataract identification and surgery, supervisory (mentorship) visits, and identification of diabetic retinopathy at hospitals. This model of collaboration between MoH and NGOs strengthens existing national health systems to increase eye health service provision.

Eye care is an integral part of the benefit packages at primary, secondary and tertiary level. These include ophthalmic consultations, refraction, surgery (cataract, glaucoma, retina), and diagnostic procedures such as biometry, perimetry and even OCT. The net result of this has been characterised by improving access to eye health care, reduction in financial risk and a widening of the services that can be obtained. The impact has been documented through reductions in VI at the population level. A RAAB in 2006[74], reported a prevalence of blindness of 1.6% in the population aged 50 and above in Rwanda while a RAAB in 2015 showed a prevalence of 1.1% in the same population. Similarly, the cataract surgical coverage in 2006 was 47% of people with bilateral cataract blindness (VA < 3/60) while it was 68.4% in 2015. Within three months of the introduction of Performance Based Financing the number of consultations reported at Health Centres and District hospitals had increased sharply. Verification processes are in place to assess whether this is due to more careful reporting of patient visits or a true increase due to increased mobilisation of patients at the primary level.

Case Study 17: Cameroon Cataract Development Impact Bond

The Cameroon Cataract Development Impact Bond is an example of a new way of funding eye health care in LMICs through a pay-for-performance loan. It is being used to address avoidable blindness through the provision of 18,000 cataract surgeries in Cameroon over the next five years.

This is a type of Development Impact Bond, a results-based contract in which investors provide financing upfront, and outcome funders repay the investors according to the success of the intervention. In an environment where resources are scarce, these instruments offer a promising new solution for more effective, efficient financing.

The initiative is led by an eye health sector partnership, which provided a $2 million loan to the Magrabi ICO Cameroon Eye Institute, financed by the U.S. International Development Finance Corporation (formerly the Overseas Private Investment Corporation) and the Netri Foundation. The implementing organisation is the African Eye Foundation; the outcome funders are Conrad N. Hilton Foundation, The Fred Hollows Foundation and Sightsavers; and the bond is managed by Volta Capital. A cross-subsidisation pricing model is used to provide quality cataract treatment services to the poor at low or no cost, while raising revenue from wealthier fee-paying patients. The hospital aims to be financially self-sufficient in five years.

Launched in March 2018, during the first year the hospital screened 54,000 people for cataract and performed 2,300 vision-restoring cataract surgeries. Staff at the hospital, including ophthalmologists and nurses, also received training and mentoring. The quality of operations exceeded WHO benchmark standards (80% achieving 6/18 or better), with 92% of patients able to see 6/18 or better following their surgery.[75] Financially, the hospital is performing better than anticipated after its first year and is on track to become financially sustainable within five years.

Case Study 18: Eye health inequality in the USA

Relying on national statistics can lead to complacency, as minority disadvantaged populations may suffer from inequities buried in overall data. The COVID-19 pandemic highlights this point, as United States (US) health officials were unaware that Black, American Indian/Alaskan native, and Latinx populations had an age-adjusted hospitalisation rate 4-5 times that of non-Hispanic whites.[76]

Eye health statistics can also obscure inequity. High Income North America has the lowest age-standardised rate of vision impairment in the world, 0.12%, largely driven by low rates in white persons. Hidden are higher rates of vision loss among women, and of blindness rates in Blacks over 40 years (0.60% in white males and 0.65% in white females compared to 1.47% in Black males and 1.01% in Black females).[77] Inequities in disease-specific rates also exist, as shown in a national sample of persons with diabetes.[78] The rates of diabetic retinopathy in Black and Latinx were 2-3 fold higher than non-Hispanic white persons. Clearly, HICs cannot rest on good metrics at the national level if there are disadvantaged populations that deserve increased attention.

In the US, eye health services are largely not integrated into other health care services. People often must take responsibility to seek care and pay for treatment. Not surprisingly, disparities along racial and socioeconomic status lines are prominent. Among those who report vision impairment, 53% of white persons report having an annual eye exam, compared to only 47% of Black and 37% of Latinx persons.[79]

HICs are not immune to the charge to “leave no one behind” and must disaggregate national data to be certain inequities are not hidden. Surveillance systems to monitor disparities in eye health should include objective measurements of vision, being mindful that questions have cultural perceptions making interpretation difficult. For example, more older Black persons have visual acuity worse than 6/12, and yet report good vision, compared to white persons.[80] In 2018, a Vision and Eye Health Surveillance System was created, which aims to identify disparities in visual health and access to care and monitor trends in prevalence, use, and practice patterns. Hopefully, the increasing awareness of inequities by the US population will also increase demand for equity in health services, including eye health, so the surveillance system will have improvements to measure.[81,82]

Case Study 19: Indigenous eye health programme in Australia

Aboriginal and Torres Strait Islander people suffer from many disadvantages including high prevalence of avoidable vision impairment.[83] A national survey found the prevalence of blindness was six times higher in these groups than for other Australians. Cataract, uncorrected refractive error, diabetic retinopathy and trachoma were the main causes. Only one third had ever had an eye examination. The unmet need was similar in urban and remote areas. A national programme for Indigenous eye health was established to close the gap for vision and provide equity in eye health.

Health system studies showed the pathway of care, or the patient journey, was like a leaky pipe with lots of cracks where people would fall out of the system. If only one or two leaks were fixed the pipe would still leak; all had to be fixed. Forty-two recommendations formed the Roadmap to Close the Gap for Vision, a long-term plan for sustainable, well-coordinated care to meet population-based needs. The key was bringing together regional stakeholders to plan and coordinate eye care from primary care in the Aboriginal Community-Controlled Health Organisations to secondary services including cataract surgery. Now there are regional networks in 59 of 63 regions across Australia. State-level committees support the regional groups.

Changes were needed in government-funded programmes for outreach services and some procedures. With extensive community input, a range of health promotional materials were developed. Eye services have increased three-fold, screening for diabetic retinopathy and cataract surgery has doubled. Although Australia is the only high-income country to still have trachoma; the prevalence has fallen from 21% in 2008 to 4% in 2018.[84] The National Eye Health Survey in 2015 showed that the gap in blindness had been halved from being 6 times to 3 times that of non-Indigenous Australians.[83]

Recommendations required:

- working closely with the community groups and organisations;
- providing care as locally as possible;
- developing regional models and stakeholder partnerships;
- planning services to meet population-based needs;
- building on local service coordination and case-management;
- performance monitoring and reporting.

Effective advocacy and policy change needed:

- a united sector that “spoke with one voice”
- a comprehensive national, state-level and regional approach
- a national focus on “solutions” not “problems”
- a local focus on “what” needs to be done and not specifically on “how” it is done

Although Indigenous Australians still have higher rates of unnecessary blindness and vision loss, substantial progress has been made and hopefully the lessons learnt can be helpful in other circumstances.

Case Study 20: The development of Eye Health Services in China, 1949 - 2020

1949 to 1978: From Liberation to Opening Up

Major eye problems facing the People's Republic of China in 1949 included high rates of vitamin A deficiency and the trachoma.[85,86] The government tackled these with national campaigns and its new Urban and Rural Medical Security Systems.[85,87] Educators targeted severely limited capacity, and the number of ophthalmologists nationwide grew from 100 in 1950 to 1000 a decade later.[88] The first successful isolation of *Chlamydia trachomatis*, the cause of trachoma, occurred in China in 1955, which together with the National Patriotic Health Campaign, helped to spur a decline in national prevalence from 50% to 40% by the early 1960s.[89-91]

1978 to 1998: 1st Round of Healthcare Reform

The new policies of "Reform and Opening Up" catalyzed a more entrepreneurial approach to healthcare, which drove a further expansion in ophthalmologists to 22,000 by 1998.[92] The introduction of new equipment, devices and techniques led to improved diagnosis, treatment and outcomes.[93] A limited number of private eye care facilities began to emerge, and ophthalmology departments at many university hospitals were established as independent eye centres.[92] In the mid-1980s, the National Committee for the Prevention of Blindness was established, with provincial and city-level offices,[93] and cataract was found responsible for half of China's blindness in the first national survey.[94] To boost public understanding of eye health, in 1996 the Ministry of Health designated June 6 as National Eye Care Day.[95]

1998 to present: National Health Insurance and 2nd Round of Healthcare Reform

The government established three basic medical insurance systems to ensure basic medical security for all, including the New Rural Cooperative Medical Care System in 2003. By 2018, over 95% of the population was covered by national basic insurance.[96] In 2009, a new round of medical reform began, aiming to improve equity and accessibility of services. These general reforms were supplemented by eye health campaigns, including the Chinese launch in 1999 of Vision 2020,[97] and cataract surgical programmes such as "The Million Poor Cataract Patient" project.[86,98] Such campaigns, together with capacity building at China's 2800 rural county-level hospitals, brought the national Cataract Surgical Rate from 277/million population/year in 1998 to 2205/million population/year in 2018.[86,99] The "Sight First China Action" project in collaboration with Lions Clubs International officially verified eradication of trachoma in China in 2014.[100,101]

Between 1990 and 2015, refractive error became the most common eye disorder in China, and by 2018 the national prevalence of myopia in children and adolescents reached 53.6%.[102,103] In view of this health threat, eight ministries under the direction of the Ministry of Education began a comprehensive national plan of management for children's myopia in August 2018.

With changes in lifestyle, diabetic retinopathy has become a major cause of vision impairment. Given that 87% of diabetic retinopathy patients are treated at county or lower level facilities, protocols and guidelines for diagnosis, treatment and referral at this level have been issued.[104,105] In addition to the government-led efforts, non-governmental organizations have also played an important role in eye health promotion in China, such as the Lions Clubs International, Lifeline Express, ORBIS International and The Fred Hollows Foundation. Thanks to these combined efforts, the prevalence of blindness and vision impairment among those aged ≥ 50 years fell by 27% and 16% respectively in the decade between 2006 and 2014.[106]

Case Study 21: Personal stories of eye health

Chan Ngin – a man from Cambodia seeing again after cataract surgery
In Cambodia, decades of turmoil have left indelible marks that serve as constant reminders of the past. For people like Chan Ngin, those reminders are close and inescapable. Like many in Cambodia, Ngin lost his legs in a landmine accident. His past is with him every day.

Spending his life in a wheelchair and being largely dependent on his wife Phork to care for him and earn money for the family, has not been easy for Ngin, an active and solidly built man. The determined Ngin did what he could, hiring himself out to clear land for other farmers – working for months at a time to ensure their land was suitable for planting rice or other crops. But Ngin had another emerging problem.

A mobile eye screening camp found cataracts in both Ngin and Phork's eyes. Ngin's were early at the time but Phork's were more advanced, so Ngin chose to wait so his wife could receive surgery first. Over the next 12 months Ngin's vision drastically deteriorated and he experienced loneliness and depression. He struggled with the inability to work and support his family. "I used to travel far away from home in my wheelchair but now I am so depressed about what I cannot do," Ngin said.

When Ngin's surgery approached, he was asked who he looked forward to seeing the most. "My wife and grandchildren – but I want to see my wife first!" he answered quickly. When a nurse removed his eye patch, Ngin was overjoyed when Phork was the first person he saw.

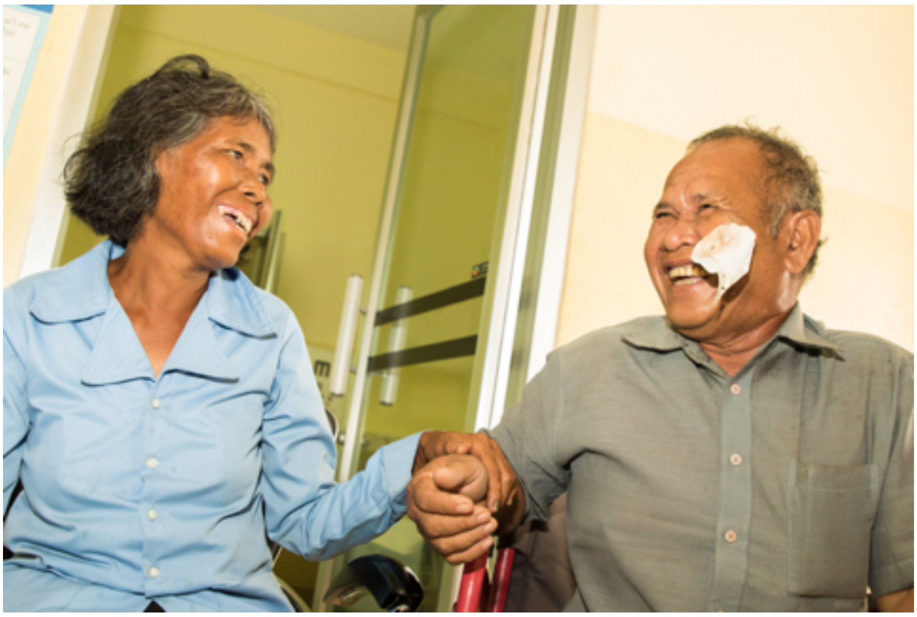

Following the cataract surgery, Ngin's quality of life improved dramatically. He is now working at a farm, supporting his family financially and saving money to build a new house. "Now that I have my sight back, I can help my family. I can get rice from the field and be the head of my family," Ngin said.

Story credit: The Fred Hollows Foundation; Photo credit: Mary Tran, The Fred Hollows Foundation.

Andrew - adjusting to sight loss from glaucoma

“If I had to stop work, I don’t know what I would have done. I love my job and being creative”. Andrew (56) makes graphics for the BBC, recently helping to design their coronavirus logo. His work is intensely visual.

Andrew was diagnosed with severe glaucoma four years ago, already blind in one eye. “People were trying to hand me things and I just wouldn’t see them. I tried to make excuses - that I was tired or not paying attention.” He underwent surgery within months to prevent further vision loss in his good eye. “The first year was a dark year. There was a lot of closing down of things”. The biggest challenge for a self-described “motor-head” (car lover) was losing the ability to drive. Over time, many skills have returned, and new ones learned: “My wife would say that I sped up, like I was needing to prove myself”. Andrew strives to reduce the impact of glaucoma on his life, from taking up painting to traveling alone internationally, running races (assisted by a friend), and continuing his voluntary work with global housing charity Habitat for Humanity.

Despite initial concerns about how his diagnosis would affect work, Andrew’s job has continued largely as before, as he retains a small island of good central vision in his left eye. “They just need to keep the lights up bright for me, it’s harder to manage if it is at all dark.” It hasn’t all been simple. Dealing with crowds (“people seem to jump at me all of a sudden”) and street furniture are challenging. Being aware of obstacles and preparing to navigate difficult situations has become normal practice. “So long as I have time to process things, I’ll be alright”.

Andrew is married with two grown-up children and does his best to minimise disruption to their lives. He takes the bus or taxis. His family sometimes has to guide him and make him aware of upcoming steps, but Andrew describes glaucoma as just another “quirky Dad characteristic, along with about 4,000 others”. People adapt quickly. “A sense of normality has returned, and when life is turned upside down that’s all you want”.

Story credit: Dr Chris Jenkins, Centre for Public Health, Queen's University Belfast.

Brian – the life transforming impact of restoring sight

"He is lazy and doesn't pay attention" Brian's form teacher told us. "His clothes are falling apart and never clean and he doesn't socialise with the other children," the Head Teacher elaborated. A positive future for Brian was difficult to imagine. It turns out it was hard for Brian to see, but nobody knew it, including Brian himself.

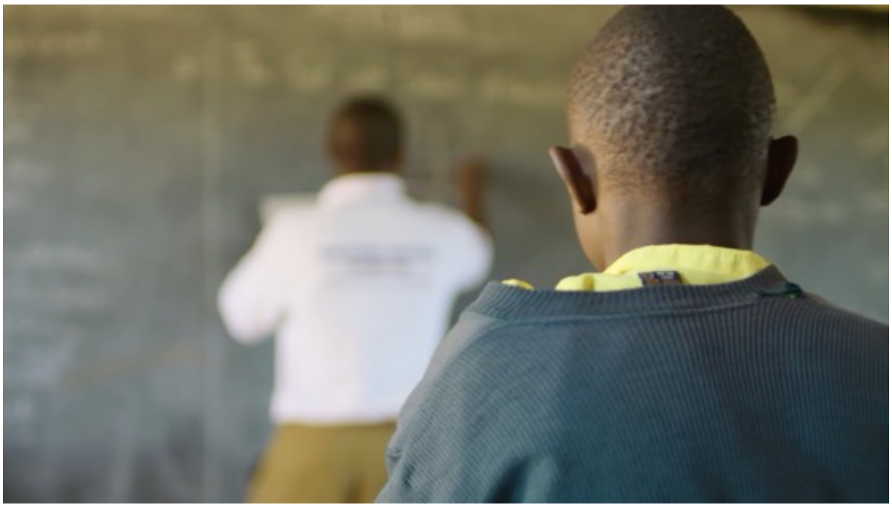

When you grow up with poor sight you have no experience of clear vision to measure against, and therefore "cannot see what you cannot see." The consequences of sight loss are far reaching. As Brian said, "I don't have friends, so I take my lunch alone at break time. My grades are not good, and it is hard for me to keep up." Brian, a twelve-year-old boy living in rural Kenya, was born with cataracts, a treatable condition if identified early and managed by an experienced ophthalmic team.

When a screening team visited Brian's school and found his vision to be severely impaired, a letter was put in his bag to take home for his parents, explaining he needed to be examined by a specialist. Sadly, Brian had lost his parents in infancy. His grandmother is his sole carer and it turns out she has also been blind for many years from cataract. Her biggest fear is that she will poison Brian, as she cannot see what crops she is picking and what she is cooking to feed him. She had only a distant memory of Brian's infant face.

Both Brian and his grandmother had successful cataract surgery, and can now see one another's faces again after ten years. Brian is able to help his grandmother around the house, and school for him is a completely changed experience. Brian's form teacher explained: "He seemed not to want to work or mix with his classmates...I never knew it was because he could not see!"

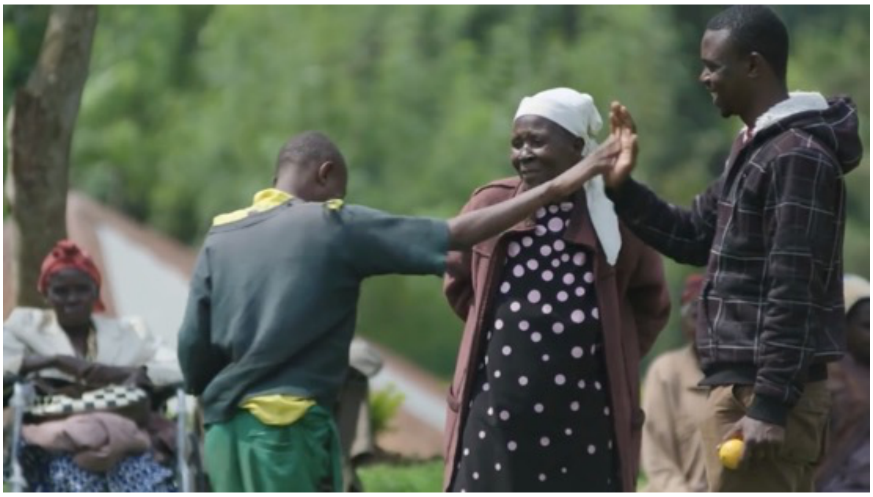

Brian and his grandmother the day they saw each other's faces for the first time in ten years.

Story and Photo credit: Peek Vision Foundation.

Rattana – eyes open to learning

Rattana and her four siblings live with their grandparents in Cambodia, as their parents have divorced and are working in Thailand. Because wages for unskilled labour are so low, neither one is able to send money home. When Rattana was younger, she had an accident that left her with poor vision and deviation of the injured eye. The injury affected both her vision and her self-esteem. She looked different from the other children and was teased because of her crossed eyes. She had difficulty with sports and struggled at school.

Through the intervention of a donor, who had also had crossed eyes as a child, Rattana had an operation to correct her condition: "Before the operation I could not see and read well…After this operation I can easily read books, and see letters on the blackboard clearly. Also, I can walk and see that everything is so beautiful. I am very happy!"

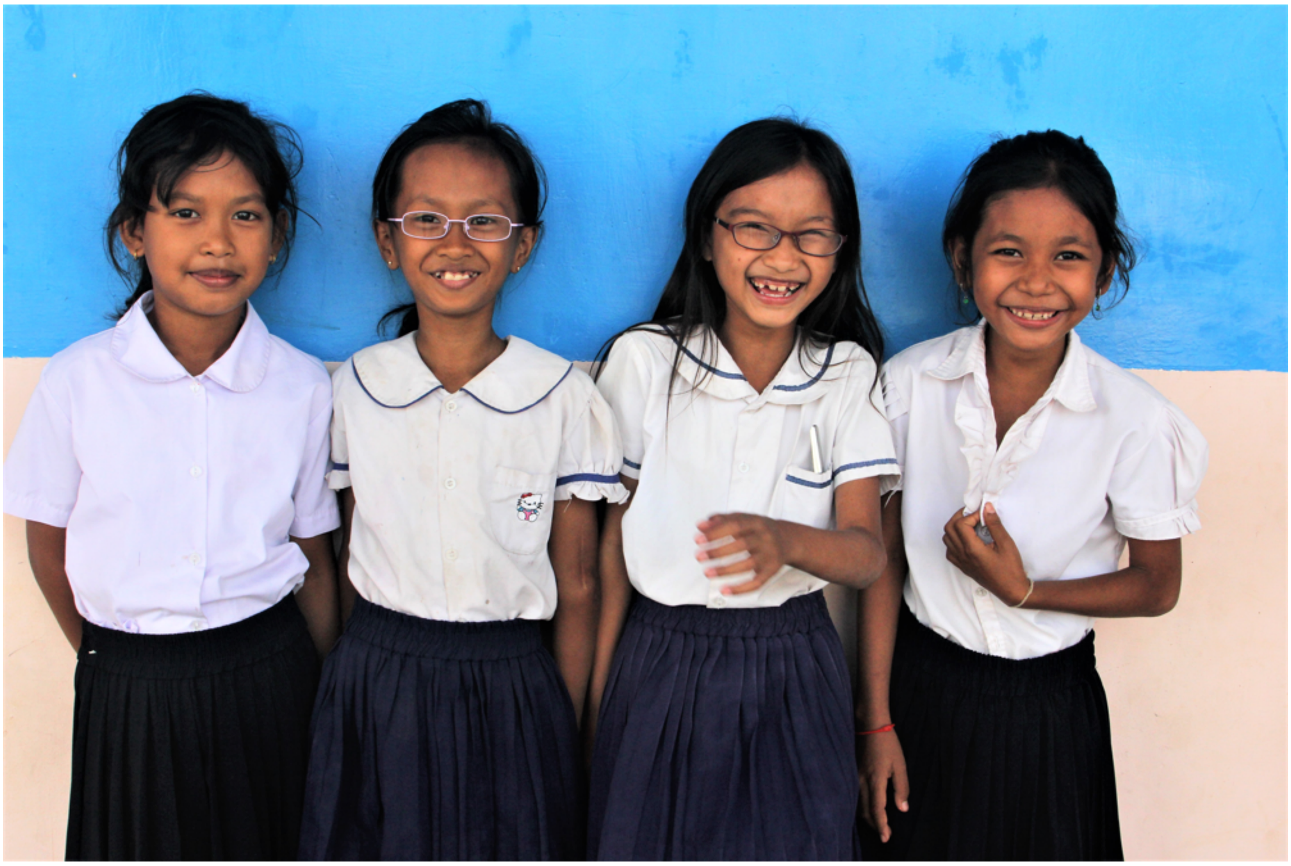

Thanks to the great success of Rattana's operation, the donor arranged for a screening outreach team to visit her school. All 350 students and staff received eye examinations, and 17 children and four teachers were found to have uncorrected refractive error. They chose from a stylish selection of frames offered by the team, and two days later received their new glasses, for many of them the first time they had had clear vision in their lives.

A lot has changed for Rattana and her schoolmates since they received vision services. Rattana is now graduating from the 9th grade. Previously shy and withdrawn, she is now frequently in the company of her friends. But ever since surgery opened up her visual world, Rattana has a new love: her classmates and teachers all tell us that she is never seen without a book under her arm, something that would have been unthinkable before her operation. Though she now has a supportive group of friends, reading on her own is her greatest pleasure. And how about Rattana's school? After the vision screening, glasses became an immediate fashion hit! All the cool kids wear them, and even students who don't need them want a pair.

Story and Photo credit: Marissa Troxel, Seva.

Abdul Mahjid – a community advocate for eye health

Mr Abdul was able to make enough money to support his family as a tailor in West Bengal, India. Three years ago, his eyesight began to fade: “I am a tailor. Losing my vision meant I was going to be unable to work.” As Mr Abdul’s vision worsened, it became harder and harder for him to thread a needle. Before long, he had lost his vision completely, and worried deeply about how to support his family. Then Mr Abdul learned about an outreach camp organized near his home. He went and learned that mature cataracts were causing his blindness. He was also told that he could receive sight-restoring surgery free of charge at a nearby donor-supported eye hospital serving the poor.

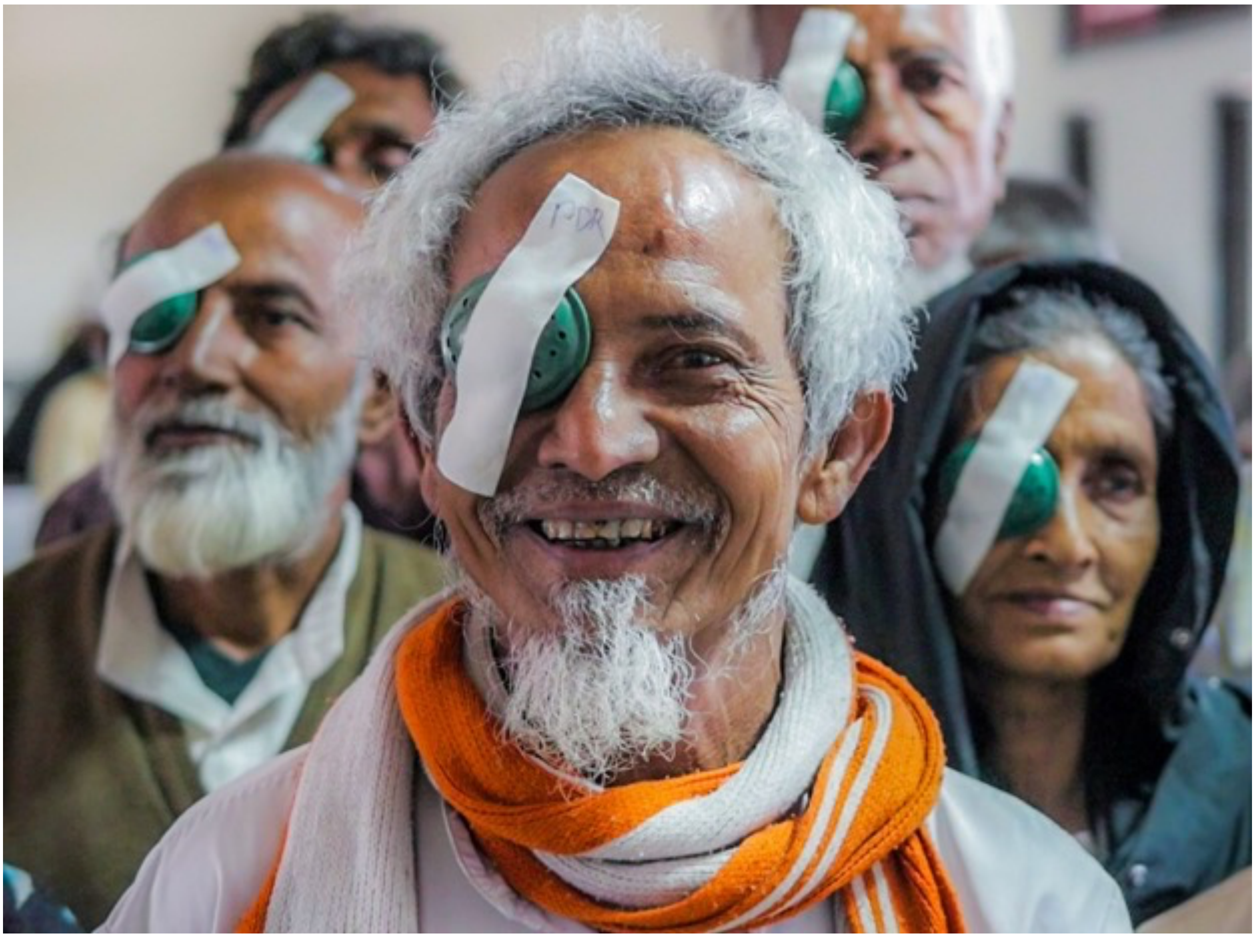

Mr Abdul travelled to the hospital where he received surgery on his left eye. His cataract was removed and an artificial replacement lens was put in its place. “I can see again! I am able to return to my work as a tailor, and can again provide for my family.” But his story doesn’t end there. The hospital’s Director and outreach worker vividly recall Mr Abdul. "Some patients in our community are actually community leaders who are not in any official position, and Mr Abdul was one of them. He was so impressed by the services that he decided to contribute by referring more patients for surgery to our hospital, so that the benefit he received can reach the maximum of his community members.”

He returned home and immediately began to spread the word to friends and neighbours in his village, who also had vision problems. Many didn’t know that sight-saving eye care was available, or they had been nervous about traveling to the hospital for treatment. Mr Abdul took it upon himself to educate anyone he could find. He gathered 18 people from his community who were losing their vision, and brought the entire group back to the eye hospital. While there, Mr Abdul received surgery on his second eye, fully restoring his vision.

As the hospital director recounts: “He himself has given blessings to everyone now.”

Story and Photo credit: Joe Raffanti, Seva

References

1. Horton S, Gelband H, Jamison D, Levin C, Nugent R, Watkins D. Ranking 93 health interventions for low- and middle-income countries by cost-effectiveness. *PLoS One* 2017; **12**(8): e0182951.
2. Polack S, Eusebio C, Fletcher A, Foster A, Kuper H. Visual impairment from cataract and health related quality of life: results from a case-control study in the Philippines. *Ophthalmic Epidemiol* 2010; **17**(3): 152-9.
3. Polack S, Eusebio C, Mathenge W, et al. The impact of cataract surgery on health related quality of life in Kenya, the Philippines, and Bangladesh. *Ophthalmic Epidemiol* 2010; **17**(6): 387-99.
4. Polack S, Eusebio C, Mathenge W, et al. The impact of cataract surgery on activities and time-use: results from a longitudinal study in Kenya, Bangladesh and the Philippines. *PLoS One* 2010; **5**(6): e10913.
5. Polack S, Kuper H, Eusebio C, Mathenge W, Wadud Z, Foster A. The impact of cataract on time-use: results from a population based case-control study in Kenya, the Philippines and Bangladesh. *Ophthalmic Epidemiol* 2008; **15**(6): 372-82.
6. Polack S, Kuper H, Mathenge W, Fletcher A, Foster A. Cataract visual impairment and quality of life in a Kenyan population. *Br J Ophthalmol* 2007; **91**(7): 927-32.
7. Polack S, Kuper H, Wadud Z, Fletcher A, Foster A. Quality of life and visual impairment from cataract in Satkhira district, Bangladesh. *Br J Ophthalmol* 2008; **92**(8): 1026-30.
8. Danquah L, Kuper H, Eusebio C, et al. The long term impact of cataract surgery on quality of life, activities and poverty: results from a six year longitudinal study in Bangladesh and the Philippines. *PLoS One* 2014; **9**(4): e94140.
9. Kuper H, Polack S, Eusebio C, Mathenge W, Wadud Z, Foster A. A case-control study to assess the relationship between poverty and visual impairment from cataract in Kenya, the Philippines, and Bangladesh. *PLoS Med* 2008; **5**(12): e244.
10. Kuper H, Polack S, Mathenge W, et al. Does cataract surgery alleviate poverty? Evidence from a multi-centre intervention study conducted in Kenya, the Philippines and Bangladesh. *PLoS One* 2010; **5**(11): e15431.
11. Syed A, Polack S, Eusebio C, et al. Predictors of attendance and barriers to cataract surgery in Kenya, Bangladesh and the Philippines. *Disability and rehabilitation* 2013; **35**(19): 1660-7.
12. Fink G, Masiye F. Health and agricultural productivity: Evidence from Zambia. *J Health Econ* 2015; **42**: 151-64.
13. Li R, Chen X, Yan H, Deurenberg P, Garby L, Hautvast JG. Functional consequences of iron supplementation in iron-deficient female cotton mill workers in Beijing, China. *Am J Clin Nutr* 1994; **59**(4): 908-13.
14. Wolgemuth JC, Latham MC, Hall A, Chesher A, Crompton DW. Worker productivity and the nutritional status of Kenyan road construction laborers. *Am J Clin Nutr* 1982; **36**(1): 68-78.
15. Basta SS, Soekirman, Karyadi D, Scrimshaw NS. Iron deficiency anemia and the productivity of adult males in Indonesia. *Am J Clin Nutr* 1979; **32**(4): 916-25.
16. Gilgen DD, Mascie-Taylor CG, Rosetta LL. Intestinal helminth infections, anaemia and labour productivity of female tea pluckers in Bangladesh. *Trop Med Int Health* 2001; **6**(6): 449-57.
17. Bourne R. Magnitude, temporal trends, and projections of the global prevalence of blindness and distance and near vision impairment: a systematic review and meta-analysis - VLEG/GBD 2020. *SUBMITTED* 2020.
18. He M, Abdou A, Ellwein LB, et al. Age-related prevalence and met need for correctable and uncorrectable near vision impairment in a multi-country study. *Ophthalmology* 2014; **121**(1): 417-22.
19. He M, Abdou A, Naidoo KS, et al. Prevalence and correction of near vision impairment at seven sites in China, India, Nepal, Niger, South Africa, and the United States. *Am J Ophthalmol* 2012; **154**(1): 107-16 e1.
20. Goertz AD, Stewart WC, Burns WR, Stewart JA, Nelson LA. Review of the impact of presbyopia on quality of life in the developing and developed world. *Acta Ophthalmol* 2014; **92**(6): 497-500.
21. Reddy PA, Congdon N, MacKenzie G, et al. Effect of providing near glasses on productivity among rural Indian tea workers with presbyopia (PROSPER): a randomised trial. *Lancet Glob Health* 2018; **6**(9): e1019-e27.

22. Junta Nacional de Auxilio (JUNAEB) Ministerio de Educación. Junaeb celebra los 25 años de servicios médicos. 2017. https://www.junaeb.cl/archivos/30120 (accessed January 20th 2020.
23. Ministerio de Educación de Chile. Ley de Presupuestos año 2019 N°21.125; 2019.
24. Ministerio de Educación de Chile. Programa Salud Del Estudiante. Normas de Control de Patologías Oftalmológicas. Santiago do Chile; 2017.
25. Ministerio de Salud de Chile. Estrategia Nacional De Salud 2011-2020. Santiago do Chile; 2011.
26. Ministerio de Salud de Chile. Resolución 1232. Programa piloto de control de salud del niño y la niña sano/a en el establecimiento educacional para la población escolar de 5 a 9 año. Santiago do Chile; 2014.
27. Ministerio de Salud de Chile. Norma Técnica para la supervisión de niños y niñas de 0 a 9 años en la Atención Primaria de Salud Programa Nacional de Salud de la Infancia. Santiago do Chile; 2014.
28. Barria F, Conte F, Muñoz S, Leasher JL, Silva JC. Prevalence of refractive error and spectacle coverage in schoolchildren in two urban areas of Chile. *Rev Panam Salud Publica* 2018; **42**: e61.
29. von-Bischhoffshausen FB, Muñoz B, Riquelme A, Ormeño MJ, Silva JC. Spectacle-wear compliance in school children in Concepción Chile. *Ophthalmic Epidemiol* 2014; **21**(6): 362-9.
30. Junta Nacional de Auxilio (JUNAEB) Ministerio de Educación. Informe final "Estudio cualitativo sobre la percepción de los usuarios directos e indirectos respecto de la entrega de los beneficios de los programas del departamento de salud del estudiante de JUNAEB". 2015. https://www.junaeb.cl/wp-content/uploads/2018/09/Estudio-de-percepci%C3%B3n-de-usuarios-Programas-Salud-del-Estudiante-JUNAEB-2015-.pdf. .
31. Jan C, Li L, Keay L, Stafford R, Congdon N, Morgan I. Prevention of myopia, China. *Bull World Health Organ* 2020; **98**: 435-7.
32. Sightsvaers. Understanding the role of lady health workers in improving access to eye health services in Khyber Pakhtunkhwa (KPK) Province of Pakistan:Study Report. Islamabad: Sightsavers, 2020.
33. Malik ANJ, Mafwiri M, Gilbert C. Integrating primary eye care into global child health policies. *Arch Dis Child* 2018; **103**(2): 176-80.
34. World Health Organization. A five year project for the prevention of childhood blindness: Report of a WHO Consultation. Geneva: World Health Organization, 2002.
35. Mafwiri MM, Kisenge R, Gilbert CE. A pilot study to evaluate incorporating eye care for children into reproductive and child health services in Dar-es-Salaam, Tanzania: a historical comparison study. *BMC Nurs* 2014; **13**: 15.
36. United Kingdom Department of Health. National Service Framework for Diabetes: Delivery Strategy. London: Department of Health, 2002.
37. United Kingdom Department of Health. Diabetic eye screening: 2016 to 2017 data. London: Department of Health; 2017.
38. United Kingdom Department of Health. Diabetic eye screening standards valid for data collected from 1 April 2019 London: Public Health England, 2019.
39. Liew G, Michaelides M, Bunce C. A comparison of the causes of blindness certifications in England and Wales in working age adults (16-64 years), 1999-2000 with 2009-2010. *BMJ open* 2014; **4**(2): e004015.
40. Arunga S, Kintoki GM, Gichuhi S, et al. Delay Along the Care Seeking Journey of Patients with Microbial Keratitis in Uganda. *Ophthalmic Epidemiol* 2019; **26**(5): 311-20.
41. Hariharan L, Gilbert CE, Quinn GE, et al. Reducing Blindness from Retinopathy of Prematurity (ROP) in Argentina Through Collaboration, Advocacy and Policy Implementation. *Health Policy Plan* 2018; **33**(5): 654-65.
42. Arnesen L, Durán P, Silva J, Brumana L. A multi-country, cross-sectional observational study of retinopathy of prematurity in Latin America and the Caribbean. *Rev Panam Salud Publica* 2016; **39**(6): 322-9.
43. Ministerio de Salud de Argentina, Grupo ROP. Guía de Práctica Clínica para la prevención, diagnóstico y tratamiento de la Retinopatía del Prematuro (ROP). Buenos Aires; 2016.
44. Silva JC, Zin A, Gilbert C. Retinopathy of prematurity prevention, screening and treatment programmes: Progress in South America. *Semin Perinatol* 2019; **43**(6): 348-51.
45. Alda E, Lomuto CC, Benítez AM, et al. Results of the National Program for the Prevention of Blindness in Childhood by Retinopathy of Prematurity in Argentina (2004-2016). *Arch Argent Pediatr* 2018; **116**(6): 386-93.

46. Farmer J. Developing eye care in papua new Guinea. *Community Eye Health* 2000; **13**(34): 26-7.
47. Newland HS, Harris MF, Walland M, et al. Epidemiology of blindness and visual impairment in Vanuatu. *Bull World Health Organ* 1992; **70**(3): 369-72.
48. Brule J, Tousignant B, Nicholls G, Pearce MG. An in-country model of workforce support for trained mid-level eye care workers in Papua New Guinea and Pacific Islands. *N Z Med J* 2017; **130**(1460): 83-6.
49. Saeedi P, Petersohn I, Salpea P, et al. Global and regional diabetes prevalence estimates for 2019 and projections for 2030 and 2045: Results from the International Diabetes Federation Diabetes Atlas, 9(th) edition. *Diabetes Res Clin Pract* 2019; **157**: 107843.
50. Vujosevic S, Aldington SJ, Silva P, et al. Screening for diabetic retinopathy: new perspectives and challenges. *Lancet Diabetes Endocrinol* 2020; **8**(4): 337-47.
51. Constable IJ, Yogesan K, Eikelboom R, Barry C, Cuypers M. Fred Hollows lecture: digital screening for eye disease. *Clin Exp Ophthalmol* 2000; **28**(3): 129-32.
52. Bastawrous A, Hennig BD. The global inverse care law: a distorted map of blindness. *Br J Ophthalmol* 2012; **96**(10): 1357-8.
53. Yau JW, Rogers SL, Kawasaki R, et al. Global prevalence and major risk factors of diabetic retinopathy. *Diabetes Care* 2012; **35**(3): 556-64.
54. Cheung N, Mitchell P, Wong TY. Diabetic retinopathy. *Lancet* 2010; **376**(9735): 124-36.
55. Tufail A, Kapetanakis VV, Salas-Vega S, et al. An observational study to assess if automated diabetic retinopathy image assessment software can replace one or more steps of manual imaging grading and to determine their cost-effectiveness. *Health Technol Assess* 2016; **20**(92): 1-72.
56. Nguyen HV, Tan GS, Tapp RJ, et al. Cost-effectiveness of a National Telemedicine Diabetic Retinopathy Screening Program in Singapore. *Ophthalmology* 2016; **123**(12): 2571-80.
57. Grzybowski A, Brona P, Lim G, et al. Artificial intelligence for diabetic retinopathy screening: a review. *Eye (Lond)* 2020; **34**(3): 451-60.
58. Ting DSW, Cheung CY, Lim G, et al. Development and Validation of a Deep Learning System for Diabetic Retinopathy and Related Eye Diseases Using Retinal Images From Multiethnic Populations With Diabetes. *JAMA* 2017; **318**(22): 2211-23.
59. Ting DSW, Lee AY, Wong TY. An Ophthalmologist's Guide to Deciphering Studies in Artificial Intelligence. *Ophthalmology* 2019; **126**(11): 1475-9.
60. Huang D, Swanson EA, Lin CP, et al. Optical coherence tomography. *Science* 1991; **254**(5035): 1178-81.
61. Maloca P, Hasler PW, Barthelmes D, et al. Safety and Feasibility of a Novel Sparse Optical Coherence Tomography Device for Patient-Delivered Retina Home Monitoring. *Transl Vis Sci Technol* 2018; **7**(4): 8.
62. Walsh AC. Binocular optical coherence tomography. *Ophthalmic Surg Lasers Imaging* 2011; **42 Suppl**: S95-S105.
63. Ting DSW, Pasquale LR, Peng L, et al. Artificial intelligence and deep learning in ophthalmology. *Br J Ophthalmol* 2019; **103**(2): 167-75.
64. Ting DSW, Peng L, Varadarajan AV, et al. Deep learning in ophthalmology: The technical and clinical considerations. *Prog Retin Eye Res* 2019; **72**: 100759.
65. De Fauw J, Ledsam JR, Romera-Paredes B, et al. Clinically applicable deep learning for diagnosis and referral in retinal disease. *Nat Med* 2018; **24**(9): 1342-50.
66. Liu H, Li L, Wormstone IM, et al. Development and Validation of a Deep Learning System to Detect Glaucomatous Optic Neuropathy Using Fundus Photographs. *JAMA Ophthalmol* 2019.
67. Yim J, Chopra R, Spitz T, et al. Predicting conversion to wet age-related macular degeneration using deep learning. *Nat Med* 2020; **26**(6): 892-9.
68. Varadarajan AV, Bavishi P, Ruamviboonsuk P, et al. Predicting optical coherence tomography-derived diabetic macular edema grades from fundus photographs using deep learning. *Nat Commun* 2020; **11**(1): 130.
69. Medeiros FA, Jammal AA, Thompson AC. From Machine to Machine: An OCT-Trained Deep Learning Algorithm for Objective Quantification of Glaucomatous Damage in Fundus Photographs. *Ophthalmology* 2019; **126**(4): 513-21.
70. INEI. Población afiliada a algún seguro de salud. Lima: Instituto Nacional de Estadística e Informática, 2018.

71. Grupo Banco Mundial. Financiamiento de la salud en Peru. Analisis de la situacion actual y desafios de la politica 2021. Lima: Banco Mundial 2016.
72. Organización Mundial de la Salud. Aplicación del instrumento de evaluacion de los servicios de atencion oftalmica (IESAO) OMS en el sistema de salud de la republica del Peru. Lima: Organización Mundial de la Salud, 2019.
73. Health Financing Strategic Plan 2018-2024. Kigali: Ministry of Health, Republic of Rwanda, 2019.
74. Mathenge W, Nkurikiye J, Limburg H, Kuper H. Rapid assessment of avoidable blindness in Western Rwanda: blindness in a postconflict setting. *PLoS Med* 2007; **4**(7): e217.
75. Spaan E, Mathijssen J, Tromp N, McBain F, ten Have A, Baltussen R. The impact of health insurance in Africa and Asia: a systematic review. *Bull World Health Organ* 2012; **90**(9): 685-92.
76. Centers for Disease Control and Prevention. COVID-19 in Racial and Ethnic Minority Groups. 2020. https://www.cdc.gov/coronavirus/2019-ncov/need-extra-precautions/racial-ethnic-minorities.html (accessed 20/07/2020).
77. Varma R, Vajaranant TS, Burkemper B, et al. Visual Impairment and Blindness in Adults in the United States: Demographic and Geographic Variations From 2015 to 2050. *JAMA Ophthalmol* 2016; **134**(7): 802-9.
78. Munoz B, West SK, Rodriguez J, et al. Blindness, visual impairment and the problem of uncorrected refractive error in a Mexican-American population: Proyecto VER. *Invest Ophthalmol Vis Sci* 2002; **43**(3): 608-14.
79. Zhang X, Cotch MF, Ryskulova A, et al. Vision health disparities in the United States by race/ethnicity, education, and economic status: findings from two nationally representative surveys. *Am J Ophthalmol* 2012; **154**(6 Suppl): S53-62 e1.
80. El-Gasim M, Munoz B, West SK, Scott AW. Discrepancies in the concordance of self-reported vision status and visual acuity in the Salisbury Eye Evaluation Study. *Ophthalmology* 2012; **119**(1): 106-11.
81. Centers for Disease Control and Prevention. Vision and Eye Health Surveillance System (VEHSS). 2020. https://www.cdc.gov/visionhealth/vehss/reports/index.html (accessed 05/08/2020).
82. Rein DB, Wittenborn JS, Phillips EA, Saaddine JB, Vision, Eye Health Surveillance System Study G. Establishing a Vision and Eye Health Surveillance System for the Nation: A Status Update on the Vision and Eye Health Surveillance System. *Ophthalmology* 2018; **125**(4): 471-3.
83. Foreman J, Xie J, Keel S, et al. The Prevalence and Causes of Vision Loss in Indigenous and Non-Indigenous Australians: The National Eye Health Survey. *Ophthalmology* 2017; **124**(12): 1743-52.
84. Australian Institute of Health and Welfare. Indigenous eye health measures 2018. Camberra: Australian Institute of Health and Welfare, 2019.
85. Wang H. [Great Achievements in Health]. *Philatelie Panorama* 2014; (10): 38-9.
86. Zhao J. [Review and outlook of the eye health in China]. *Zhonghua Yan Ke Za Zhi* 2018; **54**(8): 561-4.
87. Weng N. SM. [Changes of China’s Rural Medical Security System]. *Environment and Society* 2020; (1): 53-5.
88. Zhao K, Yang P. [Ophthalmology]. Beijing: People's Medical Publishing House; 2008.
89. Zhang X, Zhang E. [Research And Control of Trachoma in China]. *Chinese Science Bulletin* 1965; **10**(1): 47-55.
90. Jin X. [Progress in Chlamydia Trachomatis Research]. *Ophthalmology in China* 2006; **15**(3): 145-50.
91. Wang N, Deng S, Tian L. A review of trachoma history in China: research, prevention, and control. *Science China Life Sciences* 2016; **59**(6): 541-7.
92. Zhao J. [Development Trend of Ophthalmic Medical Institutions in China]. *Chinese Journal of Ophthalmology* 2003; **39**(5): 260-1.
93. Li M. [Progress of ophthalmology in China in the past 50 years]. *Chinese Journal of Ophthalmology* 2000; **36**(3): 165-70.
94. Zhang S. [50 Years of Ophthalmology in New China]. *Chinese Journal of Ophthalmology* 1999; **35**(5).
95. [Notice on Carrying out Publicity And Education Activities on “National Eye Care Day”]. 1996.
96. Administration NHS. [Statistical Bulletin on National Basic Medical Security Development in 2018]. 2019. http://www.nhsa.gov.cn/art/2019/6/30/art_7_1477.html (accessed March 21 2020).
97. Zhao J. [Vision 2020 And Blindness Prevention And Treatment in China]. *Chinese Journal of Ophthalmology* 2002; **38**(10): 577-9.

98. He X, Zhang, R., Li, Y., Hu, X. [Review And Future Direction of Eliminating Cataract Blindness in China]. *Chinese Journal of Ophthalmology* 2018; **98**(22): 1731-3.
99. Guan H. [Present Status and Development of Prevention of Blindness and Ophthalmic Epidemiologic Studies in China]. *Chinese Journal of Ophthalmology* 2010; **46**(10): 938–43.
100. Wang N, Hu, A. [Enlightenment and thinking of blinding trachoma elimination in China]. *Chinese Journal of Ophthalmology* 2015; **51**(7): 484-6.
101. Zhao J, Mariotti SP, Resnikoff S, et al. Assessment of trachoma in suspected endemic areas within 16 provinces in mainland China. *PLoS Negl Trop Dis* 2019; **13**(1): e0007130.
102. Bai J. [China Will Carry Out Targeted Myopia Interventions]. 2018. http://www.gov.cn/xinwen/2019-04/30/content_5387714.htm (accessed March 21 2020).
103. Wang B, Congdon N, Bourne R, et al. Burden of vision loss associated with eye disease in China 1990-2020: findings from the Global Burden of Disease Study 2015. *Br J Ophthalmol* 2018; **102**(2): 220-4.
104. Society RCoCD. [Expert Consensus on Prevention And Treatment of Diabetic Retinopathy]. *Chinese Journal of Diabetes Mellitus* 2018; **10**(4): 241-7.
105. Blindness NCftPo. [Guidelines for the Prevention and Treatment of Diabetic Retinopathy in China (for Primary Medical Institutions)]. Beijing: People's Medical Publishing House; 2017.
106. Zhao J, Xu X, Ellwein LB, et al. Causes of Visual Impairment and Blindness in the 2006 and 2014 Nine-Province Surveys in Rural China. *Am J Ophthalmol* 2019; **197**: 80-7.
