# Supplementary material for: The Lancet Global Health Commission on Global Eye Health: vision beyond 2020
Source: Lancet Glob Health. 2021 Feb 16;9(4):e489–551. doi: 10.1016/S2214-109X(20)30488-5 (PMC7966694; doi:10.1016/S2214-109X(20)30488-5)
Supplement: Supplementary appendix 3 [file mmc3.pdf]

THE LANCET
Global Health

Supplementary appendix 3

This appendix formed part of the original submission and has been peer reviewed. We post it as supplied by the authors.

Supplement to: Burton MJ, Ramke J, Marques AP, et al. The *Lancet Global Health* Commission on Global Eye Health: vision beyond 2020. *Lancet Glob Health* 2021; published online Feb 16. http://dx.doi.org/10.1016/S2214-109X(20)30488-5.

Figure 1: A timeline of major developments in global eye health

Global developments listed are:

- WHO founded – 1948
- First WHO Trachoma Expert Committee – 1952
- IAPB formed – 1973
- WHA resolution on blindness – 1975
- WHO Prevention of Blindness programme established – 1988
- WHO recommends vitamin A supplementation – early 1990s
- WHA resolution on trachoma – early 2000s
- VISION 2020 launched by WHO and IAPB – 1999
- WHA Elimination of avoidable blindness – late 2000s
- WHA Action Plan for prevention of avoidable blindness – 2009–13
- WHA Universal Eye Health: Global Action Plan – 2014–19
- WHO publishes World report on vision – 2019

Major treatments and programmatic developments are:

- First implanted intraocular lens – early 1950s
- First laser treatment for diabetic retinopathy – early 1960s
- Onchocerciasis control programme launched – late 1970s
- Eye drops for glaucoma – mid-1980s
- First national vision impairment survey – mid-1980s
- Ivermectin donated for onchocerciasis control – early 1990s
- Manual small-incision cataract surgery – mid-1990s
- Affordable intraocular lens produced in India and Nepal – mid-1990s
- Rapid Assessment of Cataract Surgical Services survey method developed – late 1990s
- Azithromycin donated for trachoma control – early 2000s
- Rapid Assessment of Avoidable Blindness survey method developed – late 2000s
- First anti-VEGF treatment for age-related macular degeneration – early 2010s
- Anti-VEGF treatment for diabetic retinopathy – mid-2010s

Figure 3: Multi-part figure portraying a life-course perspective on eye health

The first part of the figure tabulates the biological, social, and environmental determinants of eye disorders at different stages in the life course

- Biological determinants at birth are genetic determinants, maternal nutrition, maternal vaccination, intrauterine infections, and intrauterine growth restriction
- Biological determinants at childbirth are premature birth, low birthweight, infection, and birth injury
- Biological determinants in childhood are genetic determinants, infection, and nutritional deficiencies
- Biological determinants in adolescence are genetic determinants, infections, and trauma
- Biological determinants in adulthood are genetic determinants, diabetes, trauma, and infection
- Biological determinants in older life are ageing, genetic determinants, diabetes, trauma, and infection
- Social and environmental determinants at birth are poverty, access to health care, maternal education, maternal health care, and maternal smoking
- Social and environmental determinants at childbirth are poverty, access to health care, maternal education, maternal health care, and obstetric health care
- Social and environmental determinants in childhood are poverty, access to health care, education, nutrition, environment, and activity
- Social and environmental determinants in adolescence are poverty, access to health care, education, nutrition, environment, and activity
- Social and environmental determinants in adulthood are poverty, access to health care, education, nutrition, and occupational hazards
- Social and environmental determinants in older life are poverty, access to health care, education, and nutrition

The second part of the figure depicts the life stage at which seven ocular disorders typically present

- Cataract in childhood, adulthood, or older life
- Refractive error in childhood, adolescence, or adulthood
- Glaucoma in childhood, adulthood, or older life
- Diabetic retinopathy in adulthood or older life
- Age-related macular degeneration in adulthood or older life
- Trachoma or cornea opacification in childhood, adulthood, or older life
- Congenital conditions before birth, at childbirth, or in childhood

Finally, the figure also shows a schematic plot of the degree of functional vision against these different life stages for both an individual with a condition leading to increased vision impairment and an individual without such a condition. The point is to show that differences in functional vision begin in childhood and widen over the course of the person’s lifetime.

Figure 19: Bar chart showing the approximate proportion of the population in need of cataract surgical services that actually receives the service. The purpose is to show how the proportion gets smaller at each stage in the cascade of care, from initial contact with health services to positive health outcome. The stages in the cascade are:

- The population in need (all adults 50 years and older with vision impairment from cataract)
- Contact with health service
- Contact with an appropriately staffed and equipped cataract surgery service
- Receipt of cataract surgery
- Receipt of cataract surgery and post-operative care according to quality standards
- Receipt of cataract surgery with visual acuity after surgery of 6/12 or better

Figure 23: Schematic illustrating strategies to improve access to eye care for Indigenous and other minority ethnic groups mapped to the patient-centred access framework by Levesque et al.[339] This framework includes perception of need and desire for care, health-care seeking, health-care reaching, health-care use, and health-care consequences.

Strategies to improve approachability for Indigenous populations:

- Supplementing existing diabetes eye screening programmes with regional eye health coordinators to support training of screeners and coordinate the programme improved uptake of screening services (Moynihan, 2017) Australia

Strategies to improve approachability for ethnic minority groups:

- Incorporating vision screening into a soup kitchen programme resulted in substantial detection of and referral for vision-threatening diseases (Shahid, 2012) USA
- Personalised phone calls, in the appropriate language, improved uptake of screening appointments (Bush, 2014) UK
- Educational materials about childhood refractive error and need to see an eye care specialist improved knowledge in parents of children at risk (Frazier, 2012) USA

Strategies to improve acceptability for Indigenous populations:

- Integrating the cataract surgical pathway with the Aboriginal Medical Service at a primary-care level increased the use of cataract services (Penrose, 2018) Australia
- Storage of clinical images was aligned with First Nations Principles of Ownership, Control, Access and Possession (Kim, 2015) Canada
- A teleophthalmology clinic increased patient satisfaction and appointment attendance by recruiting Indigenous nurses, including culturally-sensitive activities, and spiritual ceremonies (Arora, 2013) Canada

Strategies to improve acceptability for ethnic minority groups:

- Community health workers from the same ethnic group facilitating patient to ophthalmologist interactions led to improved glycaemic control, linked to trust (Rovner, 2019) USA
- Having audio-recorded instructions in patient's own language was a feasible option for visual field testing in a multi-cultural context (Nesher, 2001) Israel
- Intensive, personalised follow up, including messages such as "your health matters", improved return rate after vision screening (Anderson, 2003) USA
- Tailored phone calls from culturally-appropriate health educator to build rapport and dispel fears improved completion of dilated fundus exam after screening (Walker, 2000, and Jones, 2011) USA

Strategies to improve availability and accommodation for Indigenous populations:

- Increasing the number of clinic sites, rural locations, and eye care sessions for optometrists at clinics for Indigenous people increased the number of patient attendances and spectacles dispensed (Napper, 2015) Australia
- Nurse-led diabetes eye screening in communities reduced travel costs for patients (Spurr, 2018) Canada

Strategies to improve availability and accommodation for ethnic minority groups:

- Vision screening in community sites (eg, grocery stores, banks, churches) and mobile screening (with well-equipped van) resulted in substantial detection of and referral for vision problems (Al-Aswad, 2017) USA
- Geographic information systems help to target resources for diabetic retinopathy tele-screening more effectively (Jani, 2017) USA
- Behavioural activation (eg, collaborative goal setting to help overcome avoidant tendencies) was more effective compared with general supportive therapy to improve attendance at dilated fundus exam (Winters, 2017) USA
- Removing additional barriers to follow-up, with video information, pre-scheduled appointments within short timeframes and voucher stating value of free eye exam, further improved attendance for full eye exam (compared with base program, both of which include support workers from the same ethnicity group, free exams, and reminders calls) (Zhao, 2018) USA

Strategies to improve affordability for Indigenous populations:

- Elimination of out of pocket cost for optometric consultations and implementing subsidies for spectacles resulted in increased use of the government spectacle scheme (Layland, 2004) Australia
- Introducing a fixed cost spectacle scheme reduced patient contribution required for spectacles and increased numbers of spectacles dispensed (Napper, 2015) Australia

Strategies to improve affordability for ethnic minority groups:

- Covering all examination and transportation costs appeared cost-effective in glaucoma detection, given the health benefits (Pizzi, 2018) USA

Strategies to improve appropriateness and ability to engage for Indigenous populations:

- Care coordinators who ensure patients are guided through the system, including organising transport and accommodation when necessary, were well received and should be further explored (Penrose, 2018) Australia
- Community elders or members were consulted in design of the diabetes eye screening project (Spurling, 2010) Australia

Strategies to improve appropriateness and ability to engage for ethnic minority groups:

- Hiring full time nurses in schools to manage screening through to use of glasses increased provision and use of glasses (Rodriguez, 2018) USA
- Interdisciplinary, longitudinal, coordinated, whole person-based services are effective to reduce vision loss due to diabetic retinopathy (Baker, 1993) USA
- Community health workers leading an educational support group (eg, weekly contact, help with overcoming barriers) improved concordance with recommendation provided at screening (Vaughan, 2017) USA
- Clear information in cartoons, scheduling reminders and a sticker reward system helped improve adherence with patching for children with amblyopia (Tjiam, 2012- and 2013) Netherlands
